# Exploring the under-investigated “microbial dark matter” of drinking water treatment plants

**DOI:** 10.1038/srep44350

**Published:** 2017-03-14

**Authors:** Antonia Bruno, Anna Sandionigi, Ermanno Rizzi, Marzia Bernasconi, Saverio Vicario, Andrea Galimberti, Clementina Cocuzza, Massimo Labra, Maurizio Casiraghi

**Affiliations:** 1 University of Milan-Bicocca, ZooPlantLab, Biotechnology and Biosciences Department, Piazza della Scienza 2, 20126, Milan, Italy; 2 National Research Council (CNR), Institute of Biomedical Technologies (ITB), Via Fratelli Cervi, 93, 20090 Segrate (MI), Italy; 3 Fondazione Telethon Piazza Cavour, 1, 20121, Milan, Italy; 4 Metropolitana Milanese S.p.A., Via Giuseppe Meda 44, 20141, Milan, Italy; 5 Institute of Atmospheric Pollution Research, National ResearchCouncil, C/O Physics Department, University of Bari “Aldo Moro”, Via Giovanni Amendola, 173 70126, Bari, Italy; 6 National Research Council (CNR), Institute of Biomedical and Technologies (ITB), via Giovanni Amendola, 122/D, 70126, Bari, Italy; 7 University of Milan-Bicocca, Medicine and Surgery Department, Via Cadore 48, 20126, Monza, Italy

## Abstract

Scientists recently reported the unexpected detection of unknown or poorly studied bacterial diversity in groundwater. The ability to uncover this neglected biodiversity mainly derives from technical improvements, and the term “microbial dark matter” was used to group taxa poorly investigated and not necessarily monophyletic. We focused on such under-investigated microbial dark matter of drinking water treatment plant from groundwater, across carbon filters, to post-chlorination. We tackled this topic using an integrated approach where the efficacy of stringent water filtration (10000 MWCO) in recovering even the smallest environmental microorganisms was coupled with high-throughput DNA sequencing to depict an informative spectrum of the neglected microbial diversity. Our results revealed that the composition of bacterial communities varies across the plant system: Parcubacteria (OD1) superphylum is found mainly in treated water, while groundwater has the highest heterogeneity, encompassing non-OD1 candidate phyla (Microgenomates, Saccharibacteria, Dependentiae, OP3, OP1, BRC1, WS3). Carbon filters probably act as substrate for microorganism growth and contribute to seeding water downstream, since chlorination does not modify the incoming bacterial community. New questions arise about the role of microbial dark matter in drinking water. Indeed, our results suggest that these bacteria might play a central role in the microbial dynamics of drinking water.

In October 2015, NASA announced the indirect evidence of liquid water on Mars^1^ raising hopes about the existence of the essential medium for life as was hypothesized more than twenty years ago^2^. The possible presence of putative tiny microbial cells in a meteorite from the red planet originated a never-ending debate about the existence of cells that were considered too small to be organisms by part of the scientific community. The quest for extraterrestrial life is fascinating and still unsolved, but the existence of ultra-small biodiversity and/or unknown biodiversity in aquatic environments is much closer to us than previously expected. Adopting the definition of Solden and colleagues^3^, those microorganisms accounting for a large proportion of life and biodiversity but whose basic metabolic and ecological properties are not known are called *microbial dark matter*. Candidate Phyla (CP), that are composed of uncultured organisms, constitute this under-investigated portion of biodiversity and their description represents a key challenge for the scientific community^3^.

For example, ultra-small bacteria (i.e. median cell volume: 0.009 ± 0.002 μm^3^, genome size: less than 1 Mb^4^,^5^) have been surprisingly detected in groundwater on Earth^4^,^6^ and show dimensions under the minimal predicted sizes^7^. They are currently defined as a candidate taxon, including at least 35 phyla with still unsolved phylogenetic relationships and without representatives isolated in culture^6^ with only few exceptions^8^,^9^,^10^.

These findings led researchers to consider drinking water treatment plants (DWTPs) as a source of unexpected biodiversity in terms of environmental microorganisms whose interactions at the community level are still poorly known. The occurrence of Candidate Phyla Radiation (CPR) bacteria, some of which have been shown to have ultrasmall cell sizes, and other Candidate Phyla (CP) of uncultured bacteria is underestimated in many environments, including DWTPs.

Understanding the microbial diversity and ecology of DWTPs is necessary for designing innovative and effective control strategies that will ensure safe and high quality drinking water. Previous studies of microbial communities in drinking water utilized various molecular techniques, such as DGGE, FISH, T-RFLP, clone libraries, and microarrays^11^. Only in a few recent studies have bacterial communities in this particular environment been analysed using High-Throughput DNA Sequencing (HTS) techniques^12^,^13^,^14^. In our opinion, the DWTP’s intrinsic complexity requires an innovative combined strategy, in which new and previously known tools (e.g. a stringent water filtration, HTS and bioinformatics) are used in an integrated analytic environment. In this study, we coupled the stringent filtration conditions with the deep resolution of HTS in order to cope with the significantly lower concentrations of organisms in drinking water when compared to other known cases (such as the Human Microbiome Project^15^). To our knowledge, this is the only option in a case where organisms are scattered, uncultivable, and very small. Moreover, as a post-sequencing analysis we used a phylogenetic entropy approach implemented in PhyloH^16^ to provide a comprehensive view of the microbial diversity in water samples. Differently from conventional phylogenetic methods, this is still an innovative approach in environmental microbial ecology that allows the identification of which lineage, or groups of lineages, give the most significant contribution to the diversity. Compared to conventional phylogenetic methods, the permutation process on all sequences, implemented in PhyloH, prevents any subsampling procedure (i.e. rarefaction): subsampling entails the ability to detect only highly abundant variants, reducing the resolution power, and hiding the signal coming from rare OTUs^17^.

The aim of this work was to describe the emergent microbiological water contaminants that are undetectable using classical approaches (e.g. culture based methods). The critical interpretation of the results will then improve the DWTP monitoring capacities and, consequently, their management strategies. To reach these goals, we analysed the microbial community of water at different steps in the potabilization process: i) raw water from the ground (GW), ii) water after passage across granular activated carbon filters (CF), and iii) water after chlorination (CHL), during an extended monitoring campaign of drinking water treatment plants (DWTPs) located in Milan (Northern Italy). As stated above, groundwater is characterised by low concentrated and often uncultivable microorganisms^11^ and, as has recently been discovered, very small bacteria^4^. To uncover the whole biodiversity of drinking water, we concentrated our samples using a tangential flow filtration system with a nominal pore rating of 10000 MWCO, which is more stringent than the traditional 0.2 μm pore filter. The concentration was then followed by extraction of the environmental DNA and sequencing of the 16 S rDNA V3-V4 regions.

## Results and Discussion

The core of our results is the analysis of the occurrence and diversity of *under-investigated microbial dark matter* along different steps of the DWTP. Unexpectedly, we recorded the presence of bacterial 16 S rDNA sequences even after the end of the potabilization process.

**Figure 1:**
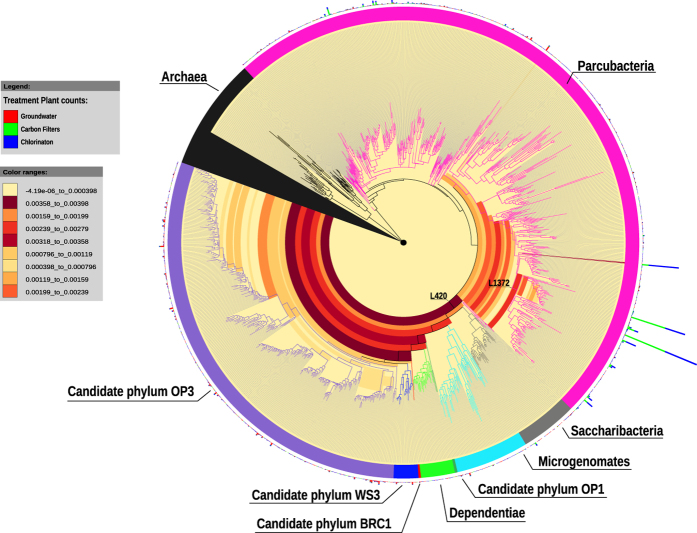
Hairy pacman graphical output from PhyloH analysis. The output couples the phylogenetic information from the RAxML tree and the contribution of the different branches/lineages to the mutual information (i.e. the information shared between each lineage and the grouping at which a given observation belongs expressed as a gradient of colours, where yellow is the null contribution and dark red the maximum). Multiple bars represent the proportion of counts associated with each lineage with respect to the three different sampling points.

**Figure 2:**
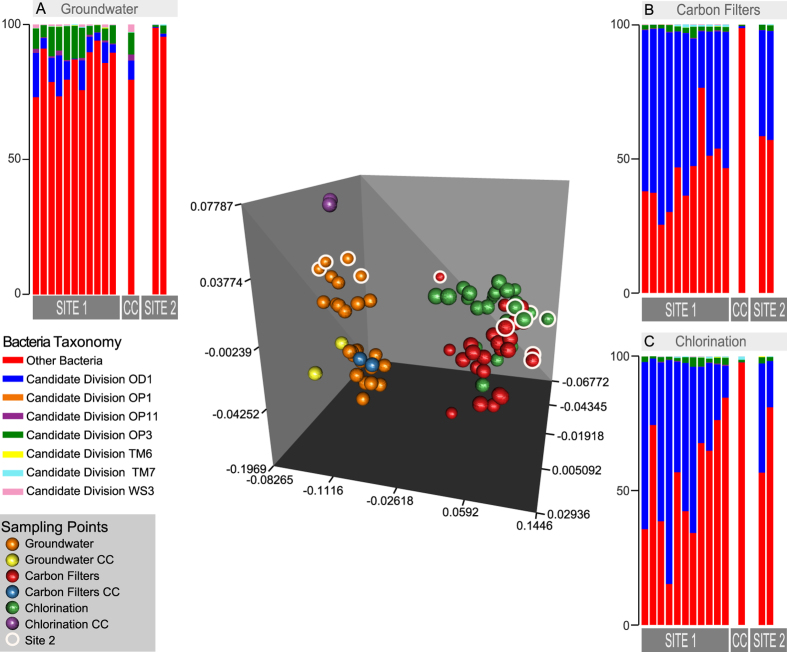
PCoA using phylogenetic turnover as a distance metric. Groundwater (GW) samples are separated by PC1 from Carbon filters (CF) and Chlorination (CHL) samples. Samples belonging to CF and CHL characterised by new (i.e. sterile) carbon filters (CC) are more similar to GW than to CF and CHL samples (see the text for further details). Samples deriving from a different DWTP (Site 2) are circled white. A-B-C barplot describes the phyla distribution of ultra-small bacteria recovered in the different sampling points.

Specifically, we found 36% of sequences belonging to Candidate Phyla and Candidate Phyla Radiation and, among these, sequences of ultra-small bacteria^4^. In total 1123 OTUs have been assigned to Parcubacteria (OD1) superphylum, Microgenomates (OP11) superphylum, Saccharibacteria (TM7), Dependentiae (TM6), OP3, OP1, BRC1, and WS3 candidate phyla (See Fig. 1 and Supplementary Table S2). The Parcubacteria superphylum was the most represented bacteria group in the DWTP (31% of the entire bacterial community, see Table S2 in Supplementary Information for the complete list), whereas all the other CPR and CP contributed only 4% of the total bacterial diversity. The tree of identified bacteria (Fig. 1) would be intended as an entropy-based “map” to estimate the total lineage diversity in our study, and it is not aimed at resolving the bacterial phylogeny. Our results showed that the investigated bacterial community varied among GW, CF, and CHL in DWTP, where groundwater was characterised by the highest diversity (α-diversity values: GW = 2.22, CF = 1.54, CHL = 1.57, for more details see html file in Supplementary Information). The GW samples shared a similar composition throughout the whole survey even when considering the different sampling sites within the whole sampling area (phylogenetic turnover mean across samples of the same group: 5%, Fig. 2 and Supplementary Information). Water samples deriving from CF and CHL shared the same diversity (β-diversity expressed as phylogenetic turnover, CF-CHL: 0.57%) which is also significantly different from GW (CF-GW: 14% and CHL-GW: 12.6%, p < 0.001). Interestingly, there was no evidence for seasonality affecting microbial composition, suggesting that groundwater could be a stable and resilient ecosystem that is not easily affected by external conditions. Noteworthy, all the classical culture methods applied to these samples highlighted the absence of pathogens commonly screened in drinking water (according to the European Directives). All the water samples included in the analysis were labelled as potable after the chlorination step (see Table S3 and S4 in Supplementary Information). Although all the drinking water around the world is treated before human consumption to remove chemical and biological contaminants, relatively little is known about the changes in microorganism composition during the potabilization processes. It is noteworthy that our data suggests that carbon filters could act as a substrate for microorganism growth and could also contribute to seeding water downstream, since chlorination did not greatly modify the incoming bacterial community (Fig. 2). Pinto and co-authors^12^ observed a similar pattern, but they did not focus on the not cultivable and poorly known bacteria diversity that is a large fraction of the total microbial diversity recovered in our results. Our data further support the seeding role of carbon filters, since samples that came from the renewed (i.e. sterile, see Supplementary Information) carbon filters were more similar to GW than to CF or CHL samples in operating conditions (CF renewed - GW: 3.72%; CF renewed - CF before filters renewing: 15.3%; CF renewed - CHL before filters renewing: 9.93%) (Fig. 2 and details in Fig. S1 in Supplementary Information). Water samples at the CHL step (after flowing through renewed filters) showed increased diversity (i.e. mean phylogenetic turnover from 5% to 16.5%) when comparing samples deriving from the CHL basin before and after filter replacement. When we excluded the samples collected during the carbon filter renewal, variation across compartment CF and CHL were similar, and variation within the three compartments across time were comparable to variation across replicates (Fig. S1 in Supplementary Information). A strong point of our approach is the capacity to identify a critical taxon with no or few previous taxonomic information due to the application of the phylogenetic entropy as implemented in PhyloH^16^. Specifically, in our study the use of PhyloH allowed us to investigate the contribution to the total diversity of different lineages instead of summarising the results as a simple check-list of predefined taxa as typically seen in many published works^16^. For instance, in our analysis, the lineage named L1372 belonging to Parcubacteria (OD1) superphylum (Fig. 1 and html in Supplementary Information) characterized treated waters (4%, 68%, and 58% of sequences of GW, CF, and CHL samples, respectively). In carbon filters, the proportion of L1372 increased dramatically reaching about 22% of the total bacterial sequences. On the contrary, lineage L420, that includes all the non-OD1 under-investigated bacteria phyla observed, was typically found in GW samples (58%, 7%, and 10% of GW, CF, and CHL samples, respectively). The Parcubacteria members not belonging to lineage L1372 were spread across the compartments at a low percentage (Fig. 1). L1372 and L420 lineages explained 6.4% of the total turnover across the three compartments. The Parcubacteria (OD1) superphylum was spread across the entire DWTP, but our results indicate that the group L420 (including all the non-OD1 under-investigated bacteria phyla observed) was typical in groundwater. Thus less abundant phyla strongly contributed to α-diversity in groundwater.

## Conclusions

Our results highlight the presence of under-investigated microorganisms across the entire DWTP, even after the potabilization process. But what are the implications of “having a drink” of these mostly unknown microorganisms? According to the parameters provided by international directives (e.g. the European 98/83/CE), drinking water analysed during this survey was clearly “labelled” as potable. Nowadays, these poorly studied bacteria are not (directly or indirectly) linked to any pathogenic condition and are not considered good markers for particular biological activities/status. Consequently, they are not routinely screened. Nevertheless, the detection of this group of uncultivable bacteria in drinking water and their incredible persistence in DWTP open new scenarios. For instance, according to their extremely small genomes, it is likely that ultra-small bacteria depend on other bacteria to survive^4^,^6^,^18^. The common theory is that they are extracellular obligate symbionts. If this is true, a simple but key question is: what are the real interactions in the microbial network characterising drinking water? Is it possible that ultra-small bacteria occurrences and concentrations are indirectly linked to the peculiarities of drinking water through the bacteria symbioses? And lastly, but with important practical consequences, could the removal or controlled maintenance of microbial dark matter affect water plant management? We have more questions than answers, but it is clear that DWTPs should be treated as complex ecosystems rather than inert systems where a tangled network of microbial interactions take place from the source (groundwater, river, lake, and so on) to the tap in our house. Better knowledge of these networks is crucial for improving the management of drinking water facilities.

## Methods

### Sampling

Samples were obtained from two drinking water treatment plants located in Milan, Italy. We collected water samples from different steps of the potabilization processes: i) from groundwater (GW), ii) after passage through granular activated carbon filters (CF), and iii) after chlorination (CHL). The sampling campaign lasted one year, from December 2013 to November 2014. In total we collected 42 samples, listed in Table S1.

### Sample concentration

In order to reduce the volume of the samples and therefore concentrate the bacteria, we used a tangential flow filtration (TFF) system. The system involves a peristaltic pump (Masterflex L/S Economy Drive), Tygon tubing, sterile reservoirs, and filtration modules. The tangential flow filter used was a VivaFlow 200 cassette (Sartorius) composed of polyethersulfone (PES) with a nominal pore rating of 10000 MWCO and a surface area of 200 cm^2^. The system was scaled up with an additional unit connected in parallel to increase the filtration surface area and the flow speed. All tubing, tubing connections, and containers were sterilized with sodium hypochlorite or autoclaved prior to each experiment. Every step was conducted in the laminar flow cabinet. The TFF system was run at a transmembrane pressure of 1.5 bar. TFF experiments were carried out within 24 h after sampling, and samples were always kept at 4 °C. For each sampling point, seven liters of water were concentrated to obtain 100 mL of operative volume.

### DNA extraction and sequencing

Environmental DNA extraction was carried out with an automated nucleic acid extraction (NucliSens EasyMAG system, bioMérieux), based on magnetic beads. Starting from 1 mL of sample, the nucleic acids were eluted into a final volume of 50 μL and stored at −80 °C. Illumina MiSeq 16 S (V3-V4 region of 16 S rRNA gene) libraries were generated following standard protocol (16 S Metagenomic Sequencing Library Preparation, Part # 15044223 Rev. B) with modifications due to the low DNA concentrations. Specifically, DNA extracts were normalized on Ct values of Real Time PCR with the same primer pairs instead of measuring the total amount of microbial DNA with fluorometric/spectrophotometric methods. Amplicon PCR was performed using the primer pairs 5′TCGTCGGCAGCGTCAGATGTGTATAAGAGACAGCCTACGGGNGGCWGCAG3′ 5′GTCTCGTGGGCTCGGAGATGTGTATAAGAGACAGGACTACHVGGGTATCTAAT CC3′ at an initial concentration of [10 μM] with the aim of increasing the volume of DNA in the reaction. The PCR-clean up step after amplicon PCR was modified in the final resuspension volume with a two-fold increase in the sample concentration (as described in Bruno and colleagues^14^). Samples were sequenced using 2 × 300 paired-end chemistry (MiSeq Reagent Kit v3). Technical replicates were included in order to verify the sequencing reproducibility (84 samples in total). 16 S rRNA gene sequence processing and operational taxonomic unit (OTU) selection Illumina reads were paired and pre-processed using USEARCH script^19^. During the Quality filter step reads were filtered out if: 1) ambiguous bases were detected, 2) lengths were outside the bounds of 250 bp and/or 3) average quality scores over a sliding window of 40 bp dropped below 25. Reads were then processed by VSEARCH 1.1.3 software version (https://github.com/torognes/vsearch), which removed noise and chimeras prior to performing de novo clustering into OTUs at 97% sequence identity and discarding those OTUs with <75 sequences.

### Microbial composition and community structure analysis

A representative sequence was selected randomly for each OTU and classified with the RDP (Ribosomal Database Project) classifier v2.2^20^ using the Silva reference set (119 release)^21^. The taxonomic assignment of each sequence was obtained with a confidence score of at least 0.8. Data included in the analyses take into consideration the fraction of the under-investigated microbial community constituted by Candidate Phyla (CP) and Candidate Phyla Radiation (CPR), and among these the occurrence of ultra-small bacteria has been reported^4^. As a consequence, a total of 3,996,876 reads passed the QC step. The OTU representative sequence set was aligned to the Silva dataset using mothur^22^, and OTUs have been assigned to Parcubacteria (OD1) superphylum, Microgenomates (OP11) superphylum, Saccharibacteria (TM7), Dependentiae (TM6), OP3, OP1, BRC1, and WS3 candidate phyla. Based on the alignment of OTU representative sequences, a phylogenetic tree was then built using RAxML version 7.4.2^23^ with the GTRGAMMA model, bootstrapping (1,000 replicates), best maximum likelihood tree inference, and displayed with iTol^24^ (http://itol.embl.de/) representing the output of PhyloH analysis with the ultra-small bacteria OTUs count as multibarplots. Multibarplots are generated with QIIME^25^. 3D PCoA was built using *rgl* R package^26^.

### PhyloH

The measurement of the variety of sequences found in the different samples was done within the overall analysis framework defined in Sandionigi *et al*.^16^, where the ecological concept of gamma diversity *D*_*γ*_ and α-diversity *D*_*α*_ are identified as the exponential of the phylogenetic generalization of Shannon (*H*_*p*_) proposed by Chao *et al*.^27^ and the exponential of the mean *H*_*p*_ per group of observations, respectively (equation (1)):


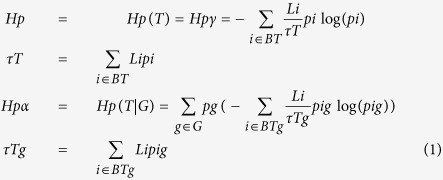


where *p*_i_ is the frequency of observations of organisms descendant of node *i* and L*i* is the length of the branch of the node *i* over the phylogenetic tree *T*. By subdividing observations in groups, it is possible to define *p*_ig_ as the frequency of observations of organisms descendant of node *i* and belonging to group *g*. The ecological concept of β-diversity is identified with the exponential of mutual information between the species observation and the grouping (I(Obs, G) as proposed by Jost (2007)^28^, and we applied this concept to the phylogenetic generalization of Shannon (equation (2))


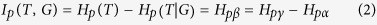


This phylogenetic generalization of mutual information describes the information shared between the lineage and the grouping at which a given observation belongs. By modifying the order of the summation, it is possible to extract the contribution of each branch/lineage to mutual information (*H*_pβi_) (equation (3))


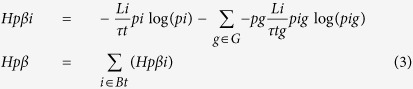


Following Chao^27^, in our work we reported the exponential of *H*_pγ_ such that the unit of measurement is an equivalent number of equi-abundant independent lineages, meaning the number of branches of a star tree in which each terminal taxon is equally abundant and would produce the same level of diversity than in the actually observed sample. As a summary of the differentiation of communities (i.e. the β-diversity), we preferred not to use the exponential of *H*_β_ that would produce estimates in equivalent number of samples, a measure quite ambiguous when samples have different numbers of observations. As a result, we normalized the *H*_β_ by its maximum possible values given the experimental design. Mutual information shared across two variables cannot be bigger than the entropy of the least entropic variable. Given I_p_(T, G), the number of groups is fixed, while *T* is unknown prior to data observation, so the maximum value that mutual information could take is H(G), and therefore, mutual information was normalized between 1 and zero using this value. This measure was defined as *turnover* and, in the case of two groups, is the percentage of observations belonging to a not shared lineage.

Due to the possible effect derived from a different amount of reads assigned to treated water and groundwater, we modified the calculation of *p*_i_ and *p*_ig_ such that each level would contribute equally to those estimates and not proportionally to its number of reads. These changes do not obscure our capacity to correctly estimate if the mutual information is different from zero. Given that, as in Sandionigi *et al*.^16^, significance was obtained by comparing the original dataset with results from a permuted data set in which grouping labels were randomly re-assigned to observations. All output files generated by PhyloH during this study are stored in the “Analysis” folder in the Supplementary Information files.

## Additional Information

**How to cite this article:** Bruno, A. *et al*. Exploring the under-investigated “microbial dark matter” of drinking water treatment plants. *Sci. Rep.* **7**, 44350; doi: 10.1038/srep44350 (2017).

**Publisher's note:** Springer Nature remains neutral with regard to jurisdictional claims in published maps and institutional affiliations.

## Supplementary Material

Supplementary Information
