# Supplementary material for: Exploring the under-investigated “microbial dark matter” of drinking water treatment plants
Source: Sci Rep. 2017 Mar 14;7:44350. doi: 10.1038/srep44350 (PMC5349567; doi:10.1038/srep44350)
Supplement: Supplementary Information [file srep44350-s1.pdf]

Exploring the under-investigated "microbial dark matter" of drinking water treatment plants

Antonia Bruno[1], Anna Sandionigi[1], Ermanno Rizzi[2,3], Marzia Bernasconi[4], Saverio Vicario[5], Andrea Galimberti[1], Clementina Cocuzza[6], Massimo Labra[1], Maurizio Casiraghi[1*]

[1] University of Milan-Bicocca, ZooPlantLab, Dept Biotechnology and Biosciences, Piazza della Scienza 2, 20126, Milan, Italy

[2] National Research Council (CNR), Institute of Biomedical Technologies (ITB), Via Fratelli Cervi, 93 - 20090 Segrate (MI) - Italy

[3] Fondazione Telethon Piazza Cavour, 1, Milano, Italy

[4] Metropolitana Milanese s.p.a., Via Giuseppe Meda 44, Milano, Italy

[5] National Research Council (CNR), Institute of Biomedical and Technologies (ITB), via Giovanni Amendola, 122/D, 70126, Bari, Italy

[6] University of Milan-Bicocca, Dept Medicine and Surgery, Via Cadore 48 , 20126, Monza, Italy

*corresponding author

maurizio.casiraghi@unimib.it

README FILE

#title: Table_S1
Description: Description table of the samples.

#title: Table_S2

Description: Table with relative abundance of the bacteria recovered.

#title: Table_S3
Description: Water samples microbiological analyses

#title: Table_S4
Description: Water samples physico-chemical analyses

#title: Table_S5_DistTurnoverBySample_all_samples
description: Phylogenetic turnover distance matrix by sample. All 84 sample are considered.

#title: Table_S6_DistTurnoverBySample_site1
Description: Phylogenetic turnover distance matrix by sample. Only sample of Site 1 and with identical carbon filter are considered.

#title: Figure_S1
Description: Pie chart of microbial composition of water samples

#title: Figure_S2
Description: Figure reporting change over time and sampling points within a DWTP, expressed as turnover

#title:output_phyloh_site1
Description: PhyloH output that can be viewed with any web browser. Only sample of Site 1 and with identical carbon filter are considered.

#title: phyloh_output_all_sample
Description: PhyloH output that can be viewed with any web browser. All 84 sample are considered.

#SampleID	DWTP	Sampling point	Date	External T (°C)	Weather
3A.s	Site 1	groundwater	Dec-13	7	sunny
3B.s	Site 1	outlet of granular activated carbon filters	Dec-13	7	sunny
3C.s	Site 1	outlet of chlorination basin	Dec-13	7	sunny
4A.s	Site 1	groundwater	Jan-14	1	cloudy
4B.s	Site 1	outlet of granular activated carbon filters	Jan-14	1	cloudy
4C.s	Site 1	outlet of chlorination basin	Jan-14	1	cloudy
5A.s	Site 1	groundwater	Feb-14	8	rain
5B.s	Site 1	outlet of granular activated carbon filters	Feb-14	8	rain
5C.s	Site 1	outlet of chlorination basin	Feb-14	8	rain
6A.s	Site 1	groundwater	Mar-14	14	sunny
6B.s	Site 1	outlet of granular activated carbon filters	Mar-14	14	sunny
6C.s	Site 1	outlet of chlorination basin	Mar-14	14	sunny
7A.s	Site 1	groundwater	Apr-14	18	sunny
7B.s	Site 1	outlet of granular activated carbon filters	Apr-14	18	sunny
7C.s	Site 1	outlet of chlorination basin	Apr-14	18	sunny
8A.s	Site 1	groundwater	May-14	19	sunny
8B.s	Site 1	outlet of granular activated carbon filters	May-14	19	sunny
8C.s	Site 1	outlet of chlorination basin	May-14	19	sunny
9A.s	Site 1	groundwater	Jun-14	16	thunderous
9B.s	Site 1	outlet of granular activated carbon filters	Jun-14	16	thunderous
9C.s	Site 1	outlet of chlorination basin	Jun-14	16	thunderous
10A.s	Site 1	groundwater	Jul-14	28	sunny

10B.s	Site 1	outlet of granular activated carbon filters	Jul-14	28	sunny
10C.s	Site 1	outlet of chlorination basin	Jul-14	28	sunny
11A.s	Site 1	groundwater	Aug-14	25	sunny
11B.s	Site 1	outlet of granular activated carbon filters	Aug-14	25	sunny
11C.s	Site 1	outlet of chlorination basin	Aug-14	25	sunny
12A.s	Site 1	groundwater	Sept-14	21	sunny
12B.s	Site 1	outlet of granular activated carbon filters	Sept-14	21	sunny
12C.s	Site 1	outlet of chlorination basin	Sept-14	21	sunny
13A.s	Site 1	groundwater	Oct-14	16	rain
13B.s	Site 1	outlet of granular activated carbon filters	Oct-14	16	rain
13C.s	Site 1	outlet of chlorination basin	Oct-14	16	rain
14A.s	Site 1	groundwater	Nov-14	10	sunny
14B.s	Site 1	outlet of granular activated carbon filters	Nov-14	10	sunny
14C.s	Site 1	outlet of chlorination basin	Nov-14	10	sunny
1A_cr.s	Site 2	groundwater	Oct-14	16	rain
1B_cr.s	Site 2	outlet of granular activated carbon filters	Oct-14	16	rain
1C_cr.s	Site 2	outlet of chlorination basin	Oct-14	16	rain
2A_cr.s	Site 2	groundwater	Nov-14	10	sunny
2B_cr.s	Site 2	outlet of granular activated carbon filters	Nov-14	10	sunny
2C_cr.s	Site 2	outlet of chlorination basin	Nov-14	10	sunny

Sample	Other Bacteria	Parcubacteria (OD1)	OP1	Microgenomates (OP11)	OP3	Dependentiae (TM6)	Saccharibacteria (TM7)	WS3
1A.cr.s	0.987770935	0.002856371	0	0.000211854	0.007246853	7.31E-06	0.001709439	0.0002411
1B.cr.s	0.584005566	0.394365757	0	0.000812932	0.019377482	0	0.001336648	0.0001329
1C.cr.s	0.567316017	0.406969697	0	0	0.023419913	0.002640693	0	0
2A.cr.s	0.954859375	0.01084375	0	0.001234375	0.0298125	0	0.00325	0
2B.cr.s	0.569904656	0.40557527	0	0.000983368	0.020586601	0.00043824	0.002939416	0
2C.cr.s	0.811387975	0.172538089	0	0	0.015361402	0	0.00182325	0
3A.s	0.734159308	0.164601729	0	0.015255883	0.075664556	0.000295872	0.001257455	0.0128057
3B.s	0.37907161	0.600296444	0	0.002624131	0.016170168	0.000243885	0.000958526	0.0012743
3C.s	0.357099684	0.622341695	0	2.39E-05	0.020693824	0	0	1.59E-05
4A.s	0.911784633	0.038659901	0	0.002945121	0.045103218	0	0.000345672	0.0017284
4B.s	0.373099874	0.610043992	0	0.002504465	0.013328107	0.000152446	0.000675117	0.0006098
4C.s	0.744383425	0.248190215	0	0.000124813	0.007988018	0	0	0
5A.s	0.788097389	0.08901517	0.000131	0.006198583	0.109096961	0.00098911	0.002073327	0.005414
5B.s	0.254136852	0.730980421	0	0.000465862	0.013416816	0.000263988	0.000121124	0.0008137
5C.s	0.386389584	0.591596459	0	0.002766511	0.017245157	0.000338554	0.001097497	0.0011378
6A.s	0.736283277	0.151987594	0	0.018506074	0.087516154	0.001530111	0.001623158	0.0056655
6B.s	0.30172487	0.669697227	0	0.002662362	0.025065851	0.000141615	0.000481491	0.0014728
6C.s	0.152578382	0.834154851	0	0.001621916	0.010677615	0.000190068	0.001081277	2.11E-05
7A.s	0.795978517	0.068272967	0	0.004417065	0.125561905	0.000171992	0.002384433	0.0033851
7B.s	0.467710072	0.505654144	0	0.001089955	0.018146054	0.000485371	0.006548248	0.0004513
7C.s	0.569095066	0.411592659	0	0.002855318	0.011235992	0.000267996	0.004658503	0.0007246
8A.s	0.871273352	0.00269819	0	0.000120995	0.121442745	0.001996419	0.002794986	0
8B.s	0.362591687	0.605145105	0	0.001009886	0.018844123	0.000903582	0.011448921	9.57E-05
8C.s	0.424044103	0.551825559	0	0.000138592	0.021250712	1.54E-05	0.002725635	0
9A.s	0.758343137	0.110189256	0	0.009411182	0.111737147	0.000165109	0.003033868	0.0087507
9B.s	0.472762236	0.474245149	0	0.00291799	0.041425893	0.000583598	0.007338028	0.000928
9C.s	0.342837591	0.618971585	0	0.000130344	0.034801877	0	0.001433785	0.0020203
10A.s	0.898645619	0.056886072	0	0.007913587	0.032519732	0.000093837	0.001126044	0.003889

10B.s	0.764289699	0.210212662	0	0.002816442	0.015089051	0	0.006326789	0.0015647
10C.s	0.677496346	0.283890544	0	0.001350626	0.033025588	0.00096209	0.002368221	0.0012581
11A.s	0.941085415	0.029627566	0.00031	0.001331228	0.026376892	0.000897805	0.000959723	0.0002167
11B.s	0.511375135	0.461767402	0.000137	0.000559531	0.017899023	0.000273813	0.00767271	0.0004524
11C.s	0.649431579	0.326905263	0	0.00151579	0.014484211	0.000336842	0.007073684	0.0005895
12A.s	0.858271115	0.075973633	5.61E-06	0.007656779	0.044253492	0.003323917	0.001378893	0.0097139
12B.s	0.538165721	0.436519898	0	0.004541689	0.013374829	0.00101246	0.0054995	0.0017229
12C.s	0.763000324	0.206852142	0	0.003816485	0.021678444	0.002852249	0.001800356	0
13A.s	0.895913718	0.030433122	0	0.003621423	0.067371637	0	0.000961323	0.0016988
13B.s	0.464865208	0.506943858	0	0.001683146	0.016817375	0.000598608	0.008803065	0.0004859
13C.s	0.846733668	0.118181634	0	0.004265278	0.025662587	0.001195494	0.003991733	1.01E-05
14A.s	0.796144354	0.071024631	0	0.022735074	0.080929343	0.000367749	0.001936813	0.0274831
14B.s	0.987257609	0.007563406	0	0.000519682	0.004585427	0.000267483	0.000323527	0.0004942
14C.s	0.977647538	0.000251054	0	0.000649278	0.009029287	0	0.012033278	0.0003896

**#SampleID**	**Coliforms at 37°C (MPN / 100 mL)**	**Enterococci (MPN / 100mL)**	***Escherichia coli (MPN / 100mL)***	***Pseudomonas aeruginosa(CFU / 250mL)***	***Clostridium perfringens* (and spores)**
3C.s	0	0	0	0	NA
4C.s	0	0	0	NA	0
5C.s	0	0	0	0	NA
6C.s	0	0	0	NA	0
7C.s	0	0	0	0	NA
8C.s	0	0	0	NA	0
9C.s	0	0	0	0	NA
10C.s	0	0	0	NA	0
11C.s	0	0	0	0	NA
12C.s	0	0	0	NA	0
13C.s	0	0	0	0	NA
14C.s	0	0	0	NA	0
1C_cr.s	0	0	0	0	NA
2C_cr.s	0	0	0	NA	0

The methods for microbiological parameters are:

(a) *Escherichia coli* (*E. coli*) and coliform bacteria (EN ISO 9308-1 or EN ISO 9308-2)

(b) *Enterococci* (EN ISO 7899-2)

(c) *Pseudomonas aeruginosa* (EN ISO 16266)

(d) *Clostridium perfringens* including spores (EN ISO 14189).

(a) The Colilert system (IDEXX Laboratories) was used according to the manufacturer's instructions. 100 mL of drinking water was mixed with the substrate and incubated in a 51 well QuantiTray for 24 h at 37 ± 1°C. Total coliform-positive wells displayed a yellow colour, whereas *E. coli*-positive wells were yellow and fluoresced under UV light (365 nm). Counts of the number of positive wells were transferred to a Most Probable Number (MPN) of the target organisms.

(b) The Enterolert system (IDEXX Laboratories) was used according to the manufacturer's instructions. 100 mL of drinking water was mixed with the substrate and incubated in a 51 well QuantiTray for 24 h at 41 ± 0.5°C. Total enterococci-positive wells fluoresced under UV light (365 nm). Counts of the number of positive wells were transferred to a Most Probable Number (MPN) of the target organisms.

(c) Membrane filtration followed by incubation of the membrane on CN agar at (36 ± 2) °C for (44 ± 4) h. Count all colonies that produce blue/green (pyocyanin) colour.

(d) Membrane filtration followed by anaerobic incubation of the membrane on m-CP agar at 44 ± 1 °C for 21 ± 3 hours. Count opaque yellow colonies that turn pink or red after exposure to ammonium hydroxide vapours for 20 to 30 seconds.

#SampleID	Alcalinity (mg/L)	Cl2(mg/L)	Colour (CU)	Conductivity at 20°C (µS/cm)	[H+] (pH)	Total hardness (°F)	Index of aggressivity	Dry residue at 180°C (mg/L)	Temperature (° C)	Turbidity (NTU)
3A.s	200	NA	0	716	7.4	30	12.14	465.4	14.6	0.01
3B.s	NA	NA	NA	NA	NA	NA	NA	NA	NA	NA
3C.s	203	0.02	0.19	704	7.45	28	12.38	457.6	12.9	0.06
4A.s	180.94	NA	<0.01	689.119	7.64	32.31	12.4	447.927	14.3	<0.010
4B.s	NA	NA	NA	NA	NA	NA	NA	NA	NA	NA
4C.s	171.56	0.03	<0.01	617.309	7.65	30.01	12.36	401.251	12.9	<0.010
5A.s	174.28	NA	<0.01	720.201	7.69	28.69	12.34	468.131	13.8	<0.010
5B.s	NA	NA	NA	NA	NA	NA	NA	NA	NA	NA
5C.s	172.05	0.01	<0.01	703.086	7.6	28.57	12.25	457.006	14.5	<0.010
6A.s	224.94	NA	<0.01	717.68	8.04	33	12.26	466.492	15.1	<0.010
6B.s	NA	NA	NA	NA	NA	NA	NA	NA	NA	NA
6C.s	221.74	0.01	<0.01	702.726	7.81	NA	NA	456.772	14.7	<0.010
7A.s	210.58	NA	<0.01	728.947	7.62	29.21	12.41	473.816	15.2	<0.010
7B.s	NA	NA	NA	NA	NA	NA	NA	NA	NA	NA
7C.s	209.66	0.02	<0.01	724.798	7.63	29.15	12.42	471.119	15.6	<0.010
8A.s	207.57	NA	<0.01	702.467	7.55	28.99	12.33	456.604	15.5	NA
8B.s	NA	NA	NA	NA	NA	NA	NA	NA	NA	NA
8C.s	207.59	0.02	<0.01	680.313	7.66	<0.01	<1.00	442.203	16	NA
9A.s	207.34	NA	<0.01	711.002	7.64	26.15	12.37	462.151	17.1	NA
9B.s	NA	NA	NA	NA	NA	NA	NA	NA	NA	NA
9C.s	207.52	0.04	<0.01	700.165	7.53	29.48	12.32	455.107	16.3	NA
10A.s	193.95	NA	<0.01	732.79	7.62	28.34	12.36	476.314	17	NA
10B.s	NA	NA	NA	NA	NA	NA	NA	NA	NA	NA
10C.s	191.08	0.01	<0.01	697.342	7.52	31.87	12.31	453.272	17.7	NA
11A.s	NA	NA	NA	NA	NA	NA	NA	NA	NA	NA
11B.s	NA	NA	NA	NA	NA	NA	NA	NA	NA	NA
11C.s	210.94	0	<0.01	725.479	7.38	33.09	12.23	471.561	18.8	NA
12A.s	206.69	NA	NA	735.464	7.77	27.32	12.53	478.052	NA	NA
12B.s	NA	NA	NA	NA	NA	NA	NA	NA	NA	NA
12C.s	210.21	0.09	NA	715.535	7.61	28.02	12.38	465.098	16.3	NA

13A.s	212.17	NA	NA	730.142	7.81	26.91	12.57	474.592	NA	<0.010
13B.s	NA	NA	NA	NA	NA	NA	NA	NA	NA	NA
13C.s	201.82	0.1	NA	703.133	7.71	30.11	12.5	457.036	15.8	<0.010
14A.s	205.37	NA	NA	715.958	7.74	26.55	12.47	465.373	NA	<0.010
14B.s	NA	NA	NA	NA	NA	NA	NA	NA	NA	NA
14C.s	204.95	0.24	NA	704.303	7.66	32.21	12.48	457.797	14.9	<0.010
1A_cr.s	203.81	NA	NA	727.981	7.77	27.41	12.51	473.188		<0.010
1B_cr.s	NA	NA	NA	NA	NA	NA	NA	NA	NA	NA
1C_cr.s	202.33	0.1	NA	716.098	7.59	31.36	12.39	465.464	15.5	<0.010
2A_cr.s	207.82	NA	NA	729.129	7.64	27.75	12.4	473.934		<0.010
2B_cr.s	NA	NA	NA	NA	NA	NA	NA	NA	NA	NA
2C_cr.s	199.06	0.03	NA	698.868	7.57	32.35	12.38	454.264	14.9	<0.010

	10A.r.s	10A.s	10B.r.s	10B.s	10C.r.s	10C.s	11A.r.s	11A.s	11B.r.s	11B.s	11C.r.s	11C.s	12A.r.s	12A.s	12B.r.s
10A.r.s															
10A.s	0.013333														
10B.r.s	0.134235	0.148236													
10B.s	0.129259	0.143160	0.007575												
10C.r.s	0.146993	0.162163	0.017643	0.017368											
10C.s	0.136735	0.151519	0.018238	0.024589	0.006099										
11A.r.s	0.063784	0.060732	0.147008	0.142266	0.150356	0.138959									
11A.s	0.064863	0.056976	0.154611	0.148788	0.159889	0.149428	0.014060								
11B.r.s	0.169007	0.187949	0.026503	0.019840	0.018173	0.028052	0.177775	0.186713							
11B.s	0.156508	0.174350	0.036214	0.026793	0.025471	0.035527	0.163262	0.172253	0.011414						
11C.r.s	0.150190	0.165677	0.023109	0.026268	0.028589	0.029933	0.155535	0.163134	0.022007	0.027171					
11C.s	0.163682	0.181595	0.019198	0.016008	0.014572	0.023464	0.173238	0.180944	0.009067	0.018375	0.014450				
12A.r.s	0.055456	0.048624	0.142590	0.139118	0.157831	0.146774	0.067461	0.071746	0.182595	0.168858	0.159088	0.177197			
12A.s	0.059913	0.053378	0.149597	0.145653	0.164683	0.153031	0.074504	0.078123	0.187936	0.174160	0.164210	0.181442	0.016025		
12B.r.s	0.135489	0.155108	0.022486	0.026502	0.032913	0.034129	0.156152	0.167094	0.028716	0.033265	0.025249	0.024179	0.150094	0.151513	
12B.s	0.142955	0.161055	0.019255	0.022201	0.030957	0.033859	0.165499	0.175497	0.028773	0.043859	0.036535	0.024350	0.154739	0.157356	0.013167
12C.r.s	0.133540	0.148612	0.022421	0.027261	0.017057	0.015217	0.132612	0.143581	0.035591	0.041035	0.033490	0.027121	0.144115	0.150617	0.032685
12C.s	0.119953	0.134002	0.037807	0.041512	0.035751	0.033465	0.119928	0.131091	0.050592	0.043987	0.037359	0.042317	0.129868	0.137040	0.035909
13A.r.s	0.089861	0.079247	0.219862	0.212412	0.219202	0.204880	0.064764	0.069502	0.251400	0.233744	0.224989	0.247527	0.080065	0.088353	0.227118
13A.s	0.087340	0.075003	0.220962	0.213111	0.221505	0.205824	0.060297	0.051278	0.252599	0.234882	0.226408	0.247861	0.086924	0.092869	0.233062
13B.r.s	0.184558	0.203435	0.029366	0.025144	0.022645	0.034156	0.194854	0.202504	0.016870	0.035565	0.035615	0.015132	0.199382	0.205251	0.032905
13B.s	0.154227	0.172375	0.032442	0.022521	0.032910	0.046207	0.165538	0.168058	0.022078	0.026062	0.034620	0.021355	0.168237	0.173936	0.033354
13C.r.s	0.112465	0.123689	0.040120	0.042464	0.036204	0.030354	0.104568	0.115161	0.057902	0.065901	0.058792	0.049422	0.116753	0.121158	0.049998
13C.s	0.123672	0.136564	0.028972	0.024625	0.023472	0.029038	0.125131	0.133397	0.037691	0.048098	0.046785	0.031290	0.131420	0.136236	0.041091
14A.r.s	0.063734	0.054982	0.189636	0.183578	0.198375	0.185122	0.059369	0.063497	0.228960	0.211256	0.202587	0.223371	0.064465	0.064852	0.195155
14A.s	0.064999	0.054945	0.198710	0.193411	0.210859	0.198358	0.077113	0.069015	0.241231	0.224456	0.213151	0.232614	0.067303	0.070076	0.209567
14B.r.s	0.052292	0.052092	0.130221	0.125165	0.143085	0.136335	0.069684	0.069996	0.167301	0.148637	0.142972	0.157989	0.073006	0.073549	0.130849
14B.s	0.043877	0.037768	0.148955	0.142681	0.163119	0.152340	0.057579	0.055401	0.186962	0.171036	0.160713	0.177469	0.054136	0.056127	0.154044
14C.r.s	0.139507	0.128998	0.216407	0.211322	0.222653	0.216188	0.114616	0.110582	0.246529	0.232179	0.214894	0.241041	0.121390	0.127117	0.230938
14C.s	0.147238	0.134083	0.222465	0.217760	0.229604	0.224404	0.121526	0.117530	0.253607	0.238847	0.221203	0.248047	0.123401	0.130697	0.238426
1A.cr.r.s	0.093445	0.082274	0.183358	0.175702	0.184169	0.172714	0.056861	0.057651	0.210259	0.195995	0.184504	0.207273	0.074643	0.082576	0.195370
1A.cr.s	0.105215	0.093663	0.199957	0.192459	0.202772	0.191690	0.076312	0.074032	0.228981	0.213380	0.198967	0.225020	0.089257	0.095489	0.211456
1B.cr.r.s	0.179484	0.196553	0.035908	0.030241	0.020655	0.031536	0.191976	0.199464	0.027511	0.043045	0.053212	0.024430	0.194649	0.196254	0.054129
1B.cr.s	0.097300	0.098147	0.123421	0.118049	0.125365	0.118974	0.095230	0.100667	0.136712	0.119292	0.116359	0.136513	0.098329	0.092659	0.120244
1C.cr.r.s	0.190150	0.208178	0.040997	0.037394	0.024951	0.035706	0.200388	0.207627	0.033883	0.051181	0.060805	0.029153	0.205604	0.210339	0.062002

12B.s	12C.r.s	12C.s	13A.r.s	13A.s	13B.r.s	13B.s	13C.r.s	13C.s	14A.r.s	14A.s	14B.r.s	14B.s	14C.r.s	14C.s	1A.cr.r.s
0.0338152															
0.0501480	0.0142730														
0.2370833	0.1942990	0.1773300													
0.2423131	0.1983024	0.1833611	0.0215289												
0.0230942	0.0374222	0.0624894	0.2711771	0.2716170											
0.0325151	0.0478480	0.0619674	0.2360672	0.2337100	0.0220364										
0.0442745	0.0279369	0.0464981	0.1569295	0.1601361	0.0589202	0.0587994									
0.0329089	0.0248167	0.0440608	0.1805873	0.1842384	0.0349312	0.0362515	0.0145210								
0.2048705	0.1763462	0.1592540	0.0618110	0.0585793	0.2463258	0.2147717	0.1439711	0.1639480							
0.2171103	0.1890489	0.1714864	0.0819597	0.0647680	0.2584299	0.2224623	0.1596081	0.1743497	0.0289918						
0.1385189	0.1251673	0.1073082	0.0984249	0.0969790	0.1808868	0.1491162	0.1123032	0.1212413	0.0679890	0.0705512					
0.1599944	0.1444700	0.1272820	0.0779129	0.0733042	0.2043219	0.1697474	0.1205340	0.1342758	0.0514984	0.0501668	0.0261187				
0.2382292	0.2036319	0.1896361	0.1069737	0.1077800	0.2587012	0.2290753	0.1775939	0.1849062	0.1166518	0.1251630	0.1384482	0.1222412			
0.2459100	0.2117398	0.1968933	0.1134654	0.1149008	0.2658650	0.2364783	0.1884964	0.1939223	0.1224882	0.1265619	0.1467970	0.1279344	0.0096237		
0.2036180	0.1662760	0.1548361	0.0522650	0.0497129	0.2305704	0.1981138	0.1290688	0.1496314	0.0636839	0.0855390	0.0969402	0.0755920	0.0988780	0.1034555	
0.2205025	0.1828498	0.1693595	0.0611360	0.0611030	0.2468678	0.2147725	0.1505489	0.1655571	0.0772207	0.0966220	0.1073277	0.0850004	0.0843830	0.0872704	0.0184795
0.0382048	0.0390411	0.0713052	0.2610691	0.2614672	0.0181132	0.0362587	0.0567192	0.0367670	0.2385053	0.2505617	0.1777390	0.2001571	0.2554031	0.2618047	0.2231532
0.1384372	0.1161852	0.1008239	0.1164364	0.1232967	0.1631089	0.1364550	0.1102810	0.1226242	0.0999787	0.1194422	0.0861108	0.0918977	0.1473579	0.1514613	0.1006568
0.0438170	0.0424459	0.0768090	0.2747390	0.2725945	0.0194758	0.0399902	0.0571980	0.0383188	0.2505645	0.2611957	0.1903690	0.2107050	0.2637540	0.2703973	0.2352540

1A.cr.s	1B.cr.r.s	1B.cr.s	1C.cr.r.s	1C.cr.s	2A.cr.r.s	2A.cr.s	2B.cr.r.s	2B.cr.s	2C.cr.r.s	2C.cr.s	3A.r.s	3A.s	3B.r.s	3B.s	3C.r.s
0.241923															
0.113970	0.147528														
0.253772	0.008528	0.176001													

3C.s	4A.r.s	4A.s	4B.r.s	4B.s	4C.r.s	4C.s	5A.r.s	5A.s	5B.r.s	5B.s	5C.r.s	5C.s	6A.r.s	6A.s	6B.r.s

6B.s	6C.r.s	6C.s	7A.r.s	7A.s	7B.r.s	7B.s	7C.r.s	7C.s	8A.r.s	8A.s	8B.r.s	8B.s	8C.r.s	8C.s	9A.r.s

9A.s	9B.r.s	9B.s	9C.r.s	9C.s

1C.cr.s	0.179916	0.197379	0.039103	0.035566	0.021572	0.030306	0.189620	0.197716	0.030587	0.045425	0.054980	0.027062	0.197238	0.203140	0.058975
2A.cr.r.s	0.099012	0.087515	0.212013	0.204718	0.215942	0.204205	0.067962	0.069406	0.243987	0.227412	0.213995	0.237515	0.088298	0.096358	0.221149
2A.cr.s	0.105025	0.091559	0.225732	0.218217	0.229317	0.217115	0.070683	0.071761	0.258494	0.241098	0.226670	0.252477	0.088814	0.096501	0.236393
2B.cr.r.s	0.174433	0.191549	0.034571	0.027506	0.021758	0.033580	0.184297	0.192168	0.028805	0.041640	0.054492	0.025368	0.189134	0.195356	0.052896
2B.cr.s	0.173412	0.191517	0.032906	0.026909	0.020562	0.031602	0.186197	0.195014	0.024068	0.036306	0.050092	0.022053	0.189552	0.188344	0.047359
2C.cr.r.s	0.169102	0.182737	0.036159	0.037052	0.020783	0.026630	0.172701	0.179272	0.033491	0.046766	0.051261	0.029475	0.179569	0.178077	0.057917
2C.cr.s	0.175681	0.190348	0.036512	0.037502	0.021666	0.028649	0.182109	0.188673	0.032837	0.047233	0.052172	0.028941	0.187394	0.185491	0.057839
3A.r.s	0.040213	0.040515	0.127406	0.122748	0.144321	0.133604	0.065315	0.072184	0.165780	0.152100	0.145956	0.160306	0.049354	0.052282	0.128284
3A.s	0.040335	0.041040	0.128077	0.123349	0.140431	0.129591	0.060065	0.067141	0.162801	0.149172	0.143735	0.158149	0.046013	0.051998	0.127867
3B.r.s	0.149941	0.171141	0.059033	0.045471	0.056956	0.070459	0.175694	0.185415	0.043954	0.044253	0.066603	0.048074	0.158164	0.161313	0.057025
3B.s	0.124560	0.139345	0.121972	0.118440	0.140602	0.138550	0.152255	0.160347	0.129838	0.124769	0.122748	0.133202	0.122336	0.119812	0.112203
3C.r.s	0.178020	0.196009	0.033258	0.035872	0.022123	0.023531	0.190303	0.199362	0.022883	0.037611	0.038935	0.025147	0.189272	0.195512	0.050933
3C.s	0.134025	0.148601	0.082416	0.077993	0.070301	0.066380	0.144262	0.154080	0.060702	0.046730	0.060705	0.072831	0.138183	0.144956	0.074644
4A.r.s	0.047118	0.043704	0.165438	0.158962	0.175616	0.162873	0.050441	0.047665	0.203188	0.187510	0.179317	0.196480	0.063671	0.069382	0.174161
4A.s	0.047964	0.044215	0.165677	0.159284	0.175825	0.162917	0.049511	0.046839	0.203657	0.188003	0.179024	0.196848	0.058456	0.064876	0.173292
4B.r.s	0.159776	0.179824	0.022986	0.026647	0.026778	0.028994	0.180127	0.187759	0.030030	0.049944	0.044871	0.027241	0.176013	0.180588	0.040136
4B.s	0.162144	0.182500	0.026453	0.029906	0.030111	0.032570	0.183076	0.190899	0.032989	0.051736	0.048098	0.029225	0.176191	0.181353	0.044520
4C.r.s	0.185653	0.205011	0.035827	0.033482	0.019271	0.026015	0.199240	0.207971	0.020929	0.035595	0.043389	0.022125	0.201591	0.208056	0.053374
4C.s	0.142895	0.158037	0.082806	0.078825	0.070776	0.066347	0.150764	0.159642	0.062697	0.047638	0.058386	0.072903	0.150706	0.157787	0.076768
5A.r.s	0.047559	0.042193	0.169595	0.164051	0.180365	0.166213	0.045867	0.050965	0.207686	0.190711	0.183657	0.202965	0.045240	0.045056	0.171156
5A.s	0.045201	0.039626	0.177007	0.170553	0.184842	0.170007	0.042373	0.047623	0.214517	0.196277	0.190055	0.209523	0.046257	0.048383	0.179409
5B.r.s	0.187945	0.209167	0.033300	0.028223	0.021750	0.032302	0.205065	0.214911	0.018934	0.035001	0.045335	0.019359	0.202772	0.208438	0.046729
5B.s	0.189375	0.210462	0.037899	0.032636	0.024472	0.035870	0.207450	0.217077	0.023355	0.040496	0.054527	0.024353	0.205513	0.211777	0.052528
5C.r.s	0.144093	0.163224	0.042283	0.029948	0.033659	0.043669	0.164048	0.173315	0.021852	0.018516	0.038766	0.028332	0.157895	0.161673	0.041797
5C.s	0.112456	0.128036	0.061464	0.058165	0.066985	0.064418	0.132863	0.141018	0.055689	0.043038	0.048042	0.062265	0.120477	0.121625	0.051374
6A.r.s	0.042490	0.040708	0.148340	0.144513	0.161786	0.151174	0.060516	0.055817	0.185308	0.171391	0.165204	0.180058	0.056086	0.059084	0.153866
6A.s	0.039123	0.038955	0.153445	0.149170	0.170303	0.158665	0.066376	0.060889	0.191484	0.176959	0.171883	0.187113	0.047463	0.053347	0.157519
6B.r.s	0.127582	0.144614	0.039395	0.026196	0.032956	0.043833	0.145064	0.153009	0.026025	0.024211	0.045398	0.030193	0.139394	0.144499	0.041315
6B.s	0.085226	0.096308	0.070725	0.067873	0.080806	0.075567	0.097701	0.109743	0.077701	0.068527	0.066715	0.081188	0.087164	0.090408	0.061030
6C.r.s	0.171509	0.190757	0.056316	0.044662	0.037065	0.046785	0.185706	0.194633	0.023022	0.015570	0.040462	0.033227	0.183644	0.190464	0.052792
6C.s	0.201359	0.221744	0.037535	0.035806	0.022186	0.031192	0.217297	0.226054	0.021564	0.037875	0.046124	0.021932	0.217247	0.223473	0.053139
7A.r.s	0.067170	0.057651	0.178074	0.170315	0.182555	0.170096	0.041570	0.032352	0.210642	0.193969	0.186391	0.205078	0.075335	0.081941	0.189662
7A.s	0.069622	0.059830	0.186986	0.179127	0.189837	0.176044	0.040530	0.034424	0.220033	0.202594	0.194314	0.214138	0.076896	0.083268	0.199858
7B.r.s	0.157639	0.176314	0.016855	0.019972	0.019153	0.022160	0.170530	0.177538	0.023021	0.036657	0.030801	0.018729	0.173631	0.176557	0.032809
7B.s	0.169631	0.189095	0.026183	0.020499	0.019592	0.031233	0.185425	0.191176	0.020457	0.033024	0.042936	0.016751	0.188004	0.191997	0.040356
7C.r.s	0.162579	0.181846	0.027463	0.020906	0.021154	0.030801	0.179339	0.189545	0.010877	0.015469	0.030307	0.015628	0.174816	0.180038	0.031894

0.046180	0.038299	0.070644	0.261644	0.260262	0.022212	0.043530	0.055432	0.039098	0.237310	0.249303	0.182185	0.200178	0.254841	0.261366	0.222422
0.230555	0.194111	0.179828	0.062921	0.061176	0.261341	0.228857	0.156945	0.177747	0.063089	0.080162	0.096430	0.076051	0.097563	0.101687	0.060736
0.245841	0.206911	0.190644	0.056682	0.056969	0.276369	0.243070	0.166496	0.188071	0.065055	0.080991	0.104734	0.082337	0.088355	0.091583	0.059049
0.034800	0.039119	0.070533	0.255908	0.255087	0.016247	0.030988	0.053785	0.031746	0.233287	0.245227	0.168825	0.192797	0.248634	0.255979	0.217391
0.033575	0.038913	0.068593	0.258505	0.259024	0.017296	0.032198	0.054601	0.033935	0.232425	0.245473	0.168355	0.193290	0.252994	0.261042	0.220040
0.046623	0.035271	0.064209	0.239916	0.237805	0.029519	0.049327	0.048141	0.034337	0.216482	0.228273	0.166772	0.184346	0.231269	0.239067	0.197816
0.045422	0.037469	0.067362	0.251404	0.249119	0.027350	0.048394	0.051814	0.036260	0.226661	0.237612	0.173220	0.192555	0.240153	0.247521	0.209508
0.135702	0.130224	0.116126	0.097512	0.100672	0.182999	0.154458	0.110225	0.122259	0.060880	0.068316	0.047713	0.039477	0.145916	0.150557	0.093577
0.135884	0.127921	0.114807	0.088744	0.093215	0.179432	0.151475	0.106550	0.122065	0.057227	0.074588	0.052178	0.040259	0.136880	0.141448	0.085169
0.050061	0.077268	0.090696	0.239692	0.246383	0.052822	0.032657	0.077666	0.060641	0.215740	0.227999	0.157122	0.173812	0.250053	0.257085	0.203530
0.111954	0.142986	0.140280	0.189285	0.198530	0.151570	0.116746	0.125332	0.134515	0.171884	0.184252	0.139180	0.141444	0.209763	0.215518	0.163603
0.040293	0.036547	0.063405	0.262850	0.263745	0.028792	0.051182	0.057360	0.049330	0.240621	0.251906	0.181195	0.201024	0.260225	0.267635	0.223704
0.092019	0.077427	0.070868	0.199328	0.203413	0.097702	0.086281	0.091364	0.095206	0.181216	0.196793	0.137813	0.149888	0.216801	0.222163	0.173176
0.181920	0.156448	0.142761	0.063921	0.052617	0.221310	0.187365	0.125264	0.144998	0.045201	0.048446	0.051093	0.036520	0.125201	0.132624	0.065092
0.181471	0.156000	0.140512	0.059059	0.052065	0.222434	0.187342	0.125318	0.144527	0.046228	0.049648	0.054101	0.038997	0.125414	0.131890	0.062198
0.022631	0.038386	0.068923	0.253452	0.253459	0.026526	0.035820	0.045381	0.037851	0.227744	0.237170	0.165955	0.184531	0.256631	0.264927	0.215275
0.024234	0.041839	0.072715	0.255353	0.255520	0.028194	0.037970	0.048287	0.040572	0.231766	0.240457	0.170022	0.188136	0.258333	0.266829	0.217414
0.041848	0.036373	0.065332	0.271980	0.271588	0.021544	0.045375	0.060833	0.045625	0.247312	0.258584	0.187416	0.209021	0.266384	0.273762	0.233862
0.098114	0.073406	0.066897	0.205731	0.206541	0.096992	0.087646	0.094549	0.097234	0.182980	0.199639	0.143969	0.156534	0.220168	0.225527	0.178756
0.178445	0.163242	0.148894	0.054066	0.057427	0.228921	0.192453	0.124921	0.148637	0.038703	0.058259	0.060176	0.039432	0.115874	0.123944	0.054623
0.187737	0.165609	0.149415	0.048395	0.047520	0.235785	0.200250	0.126225	0.150419	0.034261	0.053835	0.058144	0.039070	0.114284	0.122525	0.049878
0.033798	0.042485	0.071724	0.280622	0.282581	0.016473	0.032071	0.061777	0.043940	0.255855	0.267942	0.190828	0.212664	0.276720	0.284025	0.240995
0.035067	0.046368	0.076939	0.282020	0.283816	0.018131	0.037825	0.064084	0.044919	0.258780	0.269127	0.192851	0.214235	0.277758	0.285272	0.244714
0.046298	0.051764	0.060200	0.232258	0.234746	0.041421	0.028352	0.068047	0.051751	0.204797	0.216041	0.146591	0.164402	0.241657	0.248976	0.198348
0.069269	0.069633	0.061241	0.189713	0.192116	0.090244	0.065720	0.081617	0.082790	0.161723	0.174822	0.115812	0.128154	0.209286	0.216193	0.161150
0.158105	0.148891	0.136909	0.085083	0.076835	0.203456	0.168466	0.123526	0.140427	0.058277	0.062627	0.052119	0.044346	0.135908	0.141903	0.083129
0.160966	0.154790	0.139521	0.085344	0.075999	0.210238	0.171881	0.131341	0.145068	0.059569	0.053480	0.053693	0.042484	0.136026	0.140910	0.088434
0.041257	0.049242	0.060127	0.210556	0.212142	0.040059	0.023768	0.056867	0.040197	0.185345	0.197090	0.130708	0.147364	0.223667	0.231738	0.178662
0.074074	0.077801	0.070119	0.146340	0.154743	0.108818	0.080786	0.073435	0.082433	0.119812	0.137266	0.087245	0.094299	0.178155	0.186422	0.124584
0.063910	0.056270	0.060069	0.255164	0.256921	0.047353	0.044338	0.085381	0.066890	0.230615	0.243173	0.172127	0.192702	0.254667	0.260872	0.220039
0.041703	0.041098	0.070205	0.292806	0.292877	0.019652	0.044870	0.068584	0.050022	0.268046	0.278352	0.202624	0.225932	0.282397	0.289298	0.254399
0.198868	0.163479	0.152690	0.050617	0.031655	0.228294	0.193102	0.130429	0.151620	0.048260	0.056942	0.074206	0.055713	0.109143	0.116374	0.049796
0.208676	0.170289	0.158189	0.049401	0.030735	0.237599	0.203220	0.135303	0.157972	0.049466	0.057641	0.077203	0.058767	0.107782	0.114668	0.051263
0.023188	0.027821	0.053157	0.243299	0.241971	0.023203	0.030283	0.041208	0.031760	0.217510	0.227273	0.156929	0.176547	0.237999	0.245838	0.204810
0.027021	0.035038	0.061938	0.258243	0.257070	0.016054	0.021822	0.048382	0.028650	0.233202	0.242341	0.169324	0.189398	0.251387	0.258633	0.220311
0.029588	0.038267	0.051457	0.252289	0.256436	0.026498	0.028767	0.061881	0.044442	0.223783	0.237427	0.159662	0.181396	0.250987	0.258763	0.212185

0.241556	0.008720	0.156058	0.006473												
0.070163	0.251530	0.106428	0.263180	0.243512											
0.066461	0.268241	0.120262	0.278800	0.265526	0.010058										
0.235009	0.007436	0.157170	0.009335	0.011076	0.242813	0.260945									
0.238736	0.005634	0.145091	0.012961	0.012061	0.246466	0.264142	0.005341								
0.216796	0.013324	0.134964	0.016591	0.013055	0.226629	0.240858	0.019803	0.014139							
0.228211	0.011367	0.141644	0.014746	0.012319	0.237475	0.252038	0.018007	0.012004	0.000897						
0.108013	0.176359	0.086300	0.189466	0.176348	0.089636	0.102858	0.172116	0.170047	0.166804	0.173505					
0.099627	0.173429	0.084467	0.185482	0.172903	0.085879	0.098090	0.168248	0.168021	0.164807	0.171716	0.014819				
0.227764	0.052209	0.143158	0.057796	0.060396	0.237096	0.251273	0.050597	0.046083	0.069311	0.067397	0.143652	0.143050			
0.187047	0.154583	0.128065	0.165558	0.162461	0.190446	0.198804	0.153145	0.144137	0.157626	0.158315	0.118534	0.116539	0.064524		
0.243957	0.020602	0.148335	0.025466	0.020872	0.253067	0.268180	0.026748	0.022771	0.022358	0.021406	0.174974	0.171338	0.061280	0.140752	
0.191388	0.097238	0.092095	0.108425	0.093755	0.195649	0.207473	0.103271	0.094270	0.088937	0.092483	0.127273	0.123718	0.083661	0.110049	0.058154
0.078168	0.213109	0.093158	0.226171	0.210526	0.062843	0.072165	0.206027	0.206366	0.195430	0.204471	0.049221	0.049991	0.190924	0.155080	0.213445
0.075405	0.214036	0.093380	0.227465	0.212060	0.061759	0.069530	0.207677	0.208185	0.197154	0.206208	0.047990	0.048951	0.189259	0.152110	0.214279
0.237592	0.022030	0.153616	0.023598	0.027463	0.248812	0.264091	0.023018	0.021943	0.032552	0.030341	0.158098	0.156099	0.042215	0.112255	0.020602
0.239592	0.023124	0.157970	0.025172	0.029162	0.250972	0.265782	0.023064	0.022686	0.033377	0.031022	0.161116	0.158715	0.040760	0.108465	0.020099
0.253094	0.013343	0.157262	0.016739	0.013585	0.262642	0.278734	0.018651	0.016208	0.019414	0.017868	0.184697	0.180724	0.059323	0.154123	0.006423
0.195439	0.097673	0.095255	0.106991	0.092497	0.200551	0.213580	0.104154	0.096282	0.089831	0.093517	0.138959	0.133535	0.096975	0.132486	0.064843
0.070366	0.219923	0.087617	0.235493	0.219385	0.062476	0.069389	0.214908	0.212085	0.200356	0.210479	0.038914	0.036902	0.181793	0.130381	0.218685
0.065150	0.227552	0.093330	0.240825	0.228663	0.060463	0.060117	0.221894	0.220123	0.205620	0.215980	0.044941	0.042300	0.193314	0.145250	0.225501
0.262110	0.012118	0.166793	0.013304	0.015328	0.273344	0.289173	0.015308	0.012985	0.026307	0.023416	0.184162	0.180894	0.038826	0.135875	0.014735
0.265013	0.010883	0.172569	0.013336	0.015084	0.274283	0.289821	0.012872	0.011233	0.023786	0.020716	0.187612	0.184580	0.044343	0.145331	0.016448
0.220120	0.041552	0.122332	0.048859	0.044709	0.228083	0.241754	0.044560	0.037252	0.051211	0.050935	0.138803	0.137946	0.022024	0.097095	0.037632
0.181542	0.096029	0.089431	0.107862	0.097572	0.190000	0.201281	0.099991	0.088807	0.093598	0.095900	0.104474	0.104231	0.056498	0.064806	0.068895
0.096876	0.195863	0.095216	0.208479	0.197848	0.092809	0.097247	0.192756	0.190226	0.180309	0.188223	0.045073	0.044244	0.165559	0.129660	0.194216
0.101345	0.204237	0.100102	0.216717	0.207583	0.094741	0.097521	0.199768	0.198005	0.191015	0.198513	0.042489	0.045476	0.164246	0.121876	0.202369
0.200588	0.040026	0.118069	0.047764	0.043814	0.209052	0.224557	0.039952	0.034994	0.049642	0.050091	0.123660	0.122353	0.017954	0.093878	0.046290
0.146624	0.115267	0.076591	0.129076	0.115566	0.145401	0.160035	0.116060	0.106308	0.108624	0.112978	0.071912	0.072196	0.067120	0.054182	0.095400
0.238084	0.050220	0.132710	0.055974	0.049784	0.248036	0.261299	0.054735	0.048088	0.055881	0.055939	0.167954	0.164906	0.050862	0.138865	0.036666
0.273012	0.013610	0.173785	0.014383	0.014939	0.283254	0.298509	0.019132	0.016451	0.023266	0.020413	0.199904	0.196538	0.060394	0.162385	0.010453
0.065002	0.220985	0.102235	0.233015	0.217318	0.048109	0.053698	0.212030	0.215262	0.201540	0.211658	0.072556	0.066847	0.205947	0.169883	0.222274
0.066340	0.230733	0.110313	0.241381	0.227900	0.052513	0.050617	0.222462	0.225894	0.208906	0.219568	0.077695	0.071977	0.217180	0.179670	0.231632
0.223529	0.021157	0.137869	0.025634	0.026472	0.236732	0.250748	0.022405	0.019655	0.026496	0.025168	0.155859	0.153354	0.050379	0.121133	0.019807
0.238443	0.012832	0.156622	0.013392	0.018404	0.250647	0.265309	0.011259	0.012105	0.025463	0.023151	0.168656	0.166112	0.040865	0.136162	0.026741
0.232336	0.028441	0.131973	0.036362	0.032975	0.243345	0.258361	0.030945	0.024348	0.036704	0.035447	0.155996	0.153827	0.035899	0.114554	0.020628

0.1610289															
0.1605729	0.0070872														
0.0918509	0.1985195	0.1986830													
0.0924250	0.2017218	0.2013843	0.0031410												
0.0710623	0.2218890	0.2233407	0.0233349	0.0231439											
0.0135743	0.1675982	0.1681800	0.0971763	0.1001274	0.0679017										
0.1560302	0.0349323	0.0333747	0.2011273	0.2027833	0.2303219	0.1680327									
0.1603495	0.0321947	0.0325941	0.2102520	0.2123390	0.2369544	0.1720987	0.0122590								
0.0841898	0.2289062	0.2294403	0.0145725	0.0151302	0.0101448	0.0879834	0.2327021	0.2406163							
0.0923990	0.2297410	0.2303849	0.0167780	0.0158622	0.0106100	0.0982838	0.2347014	0.2424997	0.0051617						
0.0491088	0.1816438	0.1812994	0.0391264	0.0406042	0.0363127	0.0544239	0.1797813	0.1868664	0.0267005	0.0341135					
0.0296268	0.1441680	0.1425278	0.0722163	0.0737909	0.0788864	0.0354950	0.1342901	0.1427997	0.0751902	0.0900422	0.0319514				
0.1435250	0.0396540	0.0392989	0.1764132	0.1782743	0.2041662	0.1549490	0.0397475	0.0417937	0.2060158	0.2078023	0.1590809	0.1202499			
0.1484284	0.0439599	0.0415713	0.1816393	0.1822595	0.2119344	0.1608411	0.0415070	0.0425238	0.2121168	0.2133941	0.1627300	0.1208478	0.0155154		
0.0622670	0.1600628	0.1590538	0.0392510	0.0402907	0.0436170	0.0704813	0.1575609	0.1662757	0.0322341	0.0379024	0.0121175	0.0445841	0.1377971	0.1419121	
0.0548247	0.1022727	0.1005074	0.0873937	0.0891521	0.1069123	0.0670433	0.0869655	0.0982655	0.1021605	0.1127877	0.0596625	0.0281978	0.0822995	0.0859505	0.0478070
0.0367831	0.2083393	0.2082531	0.0596339	0.0625794	0.0337471	0.0354393	0.2128780	0.2166800	0.0370637	0.0443102	0.0174590	0.0448630	0.1894262	0.1955789	0.0318207
0.0840031	0.2422084	0.2430775	0.0222638	0.0233501	0.0053061	0.0837080	0.2510115	0.2569057	0.0064710	0.0085452	0.0379068	0.0853870	0.2216077	0.2287157	0.0471014
0.1714890	0.0336528	0.0336867	0.2108069	0.2136100	0.2306160	0.1752717	0.0424723	0.0373750	0.2390404	0.2405601	0.1935493	0.1567098	0.0552602	0.0595810	0.1713595
0.1783598	0.0380752	0.0387032	0.2219323	0.2243085	0.2400834	0.1830743	0.0462114	0.0375261	0.2493884	0.2506640	0.2028289	0.1652173	0.0569472	0.0633299	0.1820637
0.0812420	0.1914114	0.1921913	0.0093120	0.0108649	0.0211790	0.0841058	0.1958260	0.2028002	0.0163154	0.0184693	0.0362725	0.0652020	0.1714380	0.1775735	0.0372094
0.0946645	0.2055413	0.2064234	0.0144640	0.0159029	0.0199752	0.0960844	0.2131913	0.2196944	0.0107985	0.0113257	0.0321897	0.0808982	0.1862323	0.1924939	0.0309811
0.0564785	0.2007915	0.2013361	0.0256327	0.0272810	0.0198630	0.0610405	0.1995438	0.2075171	0.0158044	0.0198818	0.0158330	0.0445630	0.1772018	0.1820521	0.0233608

0.082064						
0.117861	0.033775					
0.118832	0.219194	0.251406				
0.128749	0.227151	0.260503	0.008323			
0.081485	0.050581	0.020545	0.200024	0.209569		
0.100143	0.045380	0.016880	0.214375	0.223809	0.009898	
0.069581	0.021411	0.019512	0.212261	0.222082	0.020825	0.020355

7C.s	0.1379949	0.1559938	0.0362630	0.0244910	0.0398854	0.0518673	0.1584649	0.1674550	0.0286073	0.0274040	0.0443685	0.0306204	0.1514401	0.1553474	0.0299530
8A.r.s	0.1128878	0.0996982	0.2363778	0.2273878	0.2327073	0.2180983	0.0641990	0.0662909	0.2655314	0.2477219	0.2382440	0.2624077	0.0937664	0.0994190	0.2473669
8A.s	0.1149770	0.1016348	0.2471737	0.2384418	0.2440983	0.2293043	0.0693465	0.0698990	0.2773000	0.2584450	0.2491140	0.2741614	0.0969898	0.1029785	0.2573992
8B.r.s	0.1807360	0.1985673	0.0275243	0.0221002	0.0157297	0.0278711	0.1911224	0.1975858	0.0132543	0.0260485	0.0339480	0.0132952	0.1971484	0.2018623	0.0427792
8B.s	0.1584999	0.1783352	0.0370510	0.0266530	0.0293373	0.0405683	0.1699245	0.1803461	0.0222899	0.0253329	0.0419898	0.0242113	0.1719087	0.1768564	0.0356308
8C.r.s	0.1284571	0.1424759	0.0581417	0.0549041	0.0675253	0.0649301	0.1310071	0.1375760	0.0639817	0.0553500	0.0534574	0.0619059	0.1383889	0.1469801	0.0441437
8C.s	0.1654749	0.1842375	0.0466058	0.0395795	0.0300751	0.0375431	0.1737622	0.1834565	0.0182672	0.0113680	0.0333721	0.0258289	0.1787765	0.1862388	0.0450563
9A.r.s	0.0453305	0.0422540	0.1666567	0.1606554	0.1765355	0.1640454	0.0527123	0.0569223	0.2053657	0.1895714	0.1816133	0.2003082	0.0504970	0.0559590	0.1722163
9A.s	0.0512200	0.0394202	0.1647831	0.1598645	0.1748653	0.1627632	0.0535712	0.0565903	0.2038350	0.1883759	0.1799534	0.1980078	0.0478310	0.0515065	0.1712043
9B.r.s	0.1214814	0.1367927	0.0269774	0.0195350	0.0232582	0.0299321	0.1325730	0.1428890	0.0300629	0.0317404	0.0387791	0.0264229	0.1324585	0.1384878	0.0336471
9B.s	0.1415457	0.1587397	0.0182170	0.0138150	0.0114140	0.0179532	0.1544794	0.1650831	0.0186465	0.0268630	0.0344833	0.0163773	0.1539601	0.1597772	0.0330380
9C.r.s	0.1473114	0.1626100	0.0337157	0.0299433	0.0148604	0.0177802	0.1498598	0.1599330	0.0187623	0.0194240	0.0284005	0.0234298	0.1556080	0.1623925	0.0414584
9C.s	0.1739035	0.1906961	0.0258712	0.0258514	0.0096439	0.0151998	0.1817470	0.1907320	0.0165803	0.0283671	0.0343472	0.0159614	0.1867905	0.1928582	0.0429158

0.0290556	0.0522780	0.0597835	0.2281750	0.2336260	0.0380915	0.0186974	0.0634400	0.0438489	0.1957168	0.2103285	0.1334807	0.1527495	0.2321384	0.2391671	0.1933571
0.2577668	0.2080275	0.1941458	0.0469470	0.0437990	0.2852123	0.2522551	0.1617638	0.1894174	0.0665190	0.0873101	0.1154870	0.0924493	0.0882651	0.0958950	0.0523047
0.2680610	0.2173155	0.2016607	0.0468730	0.0439318	0.2967697	0.2630400	0.1718170	0.1999250	0.0668299	0.0872660	0.1179057	0.0945927	0.0919861	0.0989924	0.0545972
0.0337119	0.0339959	0.0605665	0.2657250	0.2635802	0.0112725	0.0263547	0.0598767	0.0354504	0.2412350	0.2506998	0.1768440	0.1995648	0.2522850	0.2587290	0.2253371
0.0346849	0.0451447	0.0608844	0.2395282	0.2432698	0.0276130	0.0171824	0.0552430	0.0387048	0.2178881	0.2324651	0.1584454	0.1787927	0.2461850	0.2538910	0.2044040
0.0608371	0.0577170	0.0528465	0.1877461	0.1891325	0.0787381	0.0548921	0.0591517	0.0597677	0.1716091	0.1876560	0.1253471	0.1378327	0.1991144	0.2042295	0.1544367
0.0545002	0.0446030	0.0479968	0.2426287	0.2435690	0.0426082	0.0424884	0.0699644	0.0552397	0.2226900	0.2357613	0.1639204	0.1843985	0.2430901	0.2499580	0.2053044
0.1803700	0.1579110	0.1435072	0.0581605	0.0596723	0.2242297	0.1924567	0.1277904	0.1465904	0.0349797	0.0529920	0.0559745	0.0410890	0.1203848	0.1277683	0.0591858
0.1772420	0.1584118	0.1452910	0.0663347	0.0645148	0.2209569	0.1913269	0.1304729	0.1484080	0.0383543	0.0545691	0.0591899	0.0404194	0.1172728	0.1227033	0.0639241
0.0327300	0.0348164	0.0482015	0.1979351	0.2004410	0.0347229	0.0222620	0.0390185	0.0275437	0.1702910	0.1851450	0.1183970	0.1369525	0.2133029	0.2217391	0.1636147
0.0254223	0.0270161	0.0479072	0.2242645	0.2264620	0.0228891	0.0260473	0.0381667	0.0259472	0.1976294	0.2110161	0.1408271	0.1602989	0.2329450	0.2415472	0.1877252
0.0491621	0.0300674	0.0406153	0.2153783	0.2174133	0.0376484	0.0384515	0.0488623	0.0404035	0.1951611	0.2088533	0.1461058	0.1615600	0.2241125	0.2318784	0.1787065
0.0370080	0.0259303	0.0510424	0.2540308	0.2541547	0.0192007	0.0379151	0.0485682	0.0344240	0.2312063	0.2425222	0.1732887	0.1924792	0.2502724	0.2570975	0.2157647

0.2115020	0.0478965	0.1252998	0.0537284	0.0561100	0.2197317	0.2344033	0.0433537	0.0415573	0.0634425	0.0624790	0.1289914	0.1279720	0.0241328	0.0956608	0.0604535
0.0665102	0.2764927	0.1348338	0.2854840	0.2718975	0.0526482	0.0443840	0.2703360	0.2738541	0.2475381	0.2595590	0.1144217	0.1061771	0.2616194	0.2084314	0.2765751
0.0662549	0.2889623	0.1412802	0.2970720	0.2840241	0.0548808	0.0457344	0.2814871	0.2855193	0.2588680	0.2709284	0.1195578	0.1100017	0.2713610	0.2135441	0.2887127
0.2413264	0.0109162	0.1550255	0.0145110	0.0147487	0.2551620	0.2700274	0.0122928	0.0112653	0.0194497	0.0177048	0.1792590	0.1759884	0.0516440	0.1520989	0.0178892
0.2256590	0.0332402	0.1417765	0.0343678	0.0377043	0.2375187	0.2515145	0.0311130	0.0305073	0.0489267	0.0480715	0.1548678	0.1513809	0.0273367	0.1170349	0.0474463
0.1686784	0.0965300	0.1102559	0.1010065	0.0969191	0.1805009	0.1900258	0.0897639	0.0915901	0.0981530	0.1008563	0.1247940	0.1217388	0.0881299	0.1217350	0.0961218
0.2234104	0.0488087	0.1292054	0.0556697	0.0474545	0.2355071	0.2484209	0.0502077	0.0448924	0.0491428	0.0496091	0.1638350	0.1604689	0.0585334	0.1358987	0.0332771
0.0720521	0.2154245	0.0900889	0.2296232	0.2131863	0.0612269	0.0721117	0.2092227	0.2091122	0.1968989	0.2068083	0.0392738	0.0382583	0.1885397	0.1490314	0.2162333
0.0768970	0.2105811	0.0899992	0.2246047	0.2086802	0.0674590	0.0785204	0.2058824	0.2053287	0.1920942	0.2016720	0.0398902	0.0397899	0.1882250	0.1514857	0.2107865
0.1860555	0.0376873	0.1165025	0.0411760	0.0403964	0.1919474	0.2086208	0.0323993	0.0333211	0.0465415	0.0475204	0.1147999	0.1125693	0.0317035	0.1043889	0.0490477
0.2099799	0.0186408	0.1281717	0.0233585	0.0216510	0.2173794	0.2336051	0.0184511	0.0165870	0.0265078	0.0263932	0.1360081	0.1337930	0.0356332	0.1164730	0.0244930
0.1999040	0.0367320	0.1149600	0.0408814	0.0335450	0.2108194	0.2239502	0.0399271	0.0358841	0.0326629	0.0348340	0.1421101	0.1378163	0.0556654	0.1321897	0.0258442
0.2351462	0.0142721	0.1461694	0.0160944	0.0129112	0.2465152	0.2610601	0.0180962	0.0163022	0.0155217	0.0151072	0.1713214	0.1673265	0.0599291	0.1522400	0.0111575

0.081281	0.175029	0.175409	0.039215	0.043039	0.055906	0.084694	0.170324	0.177953	0.038538	0.044022	0.021040	0.052123	0.151438	0.154356	0.018834
0.214471	0.073240	0.072462	0.274443	0.276047	0.285353	0.220575	0.065538	0.054436	0.297388	0.299383	0.250170	0.209863	0.102470	0.106118	0.229303
0.223755	0.074320	0.073585	0.285512	0.287194	0.297395	0.228202	0.068221	0.055420	0.309590	0.311642	0.260976	0.217681	0.105673	0.107906	0.239732
0.084398	0.215147	0.216289	0.024127	0.025123	0.011004	0.083103	0.224508	0.230109	0.010725	0.012007	0.031576	0.080794	0.197599	0.205512	0.033596
0.082258	0.193586	0.193728	0.032628	0.036647	0.040779	0.080507	0.194539	0.201079	0.026254	0.031569	0.019489	0.064287	0.175201	0.180928	0.019514
0.085270	0.149962	0.148071	0.081406	0.086095	0.099060	0.078164	0.153052	0.154618	0.092580	0.102158	0.070734	0.061315	0.142151	0.145866	0.068456
0.039235	0.197852	0.197942	0.057414	0.057183	0.031378	0.038540	0.204695	0.208374	0.040108	0.043692	0.026964	0.047886	0.183626	0.189544	0.036673
0.159993	0.030661	0.032537	0.201195	0.204144	0.224852	0.167300	0.021746	0.022324	0.230286	0.231705	0.181488	0.141122	0.041736	0.045737	0.160766
0.154711	0.034165	0.037556	0.198964	0.202109	0.220909	0.163632	0.023803	0.026564	0.226730	0.228695	0.179461	0.141320	0.041003	0.048414	0.158364
0.078811	0.146587	0.147950	0.033519	0.038355	0.046466	0.081309	0.147143	0.154062	0.034824	0.041816	0.026116	0.058266	0.134891	0.141683	0.018747
0.075425	0.172707	0.173695	0.016883	0.020052	0.022436	0.079124	0.174127	0.181510	0.015127	0.017850	0.022088	0.061363	0.156580	0.162896	0.020868
0.043380	0.173444	0.173862	0.041949	0.046068	0.025721	0.042988	0.176565	0.182002	0.031794	0.036877	0.024635	0.050000	0.162376	0.168940	0.031450
0.069964	0.207093	0.208135	0.023570	0.026175	0.007609	0.068494	0.214379	0.220455	0.013294	0.015078	0.033604	0.073690	0.192247	0.199780	0.039815

0.061038	0.041417	0.054794	0.190100	0.199683	0.037144	0.029198	0.022529								
0.163494	0.268578	0.306568	0.050634	0.045043	0.259056	0.274158	0.268752	0.248551							
0.170351	0.279783	0.319153	0.052252	0.048895	0.271063	0.286027	0.280426	0.258465	0.007905						
0.104415	0.034678	0.010226	0.221343	0.230064	0.016305	0.010979	0.018213	0.039123	0.277743	0.289821					
0.079243	0.034499	0.039535	0.202344	0.211614	0.031346	0.020004	0.025627	0.014828	0.257772	0.268742	0.026236				
0.068843	0.076412	0.101341	0.154373	0.160563	0.067746	0.073823	0.072878	0.051513	0.199448	0.208749	0.083124	0.055738			
0.078508	0.013208	0.036131	0.204471	0.211819	0.044984	0.042093	0.020944	0.045285	0.253047	0.264894	0.031420	0.038284	0.061228		
0.097163	0.209459	0.245265	0.044233	0.049838	0.195149	0.210608	0.199312	0.171071	0.072835	0.074409	0.219303	0.194974	0.154496	0.202301	
0.097305	0.205665	0.240400	0.048590	0.052601	0.193376	0.207919	0.197290	0.170454	0.076328	0.079844	0.215029	0.193034	0.157273	0.200944	0.013317
0.059733	0.047598	0.050040	0.159351	0.169161	0.034415	0.028229	0.030725	0.019942	0.213777	0.224431	0.034616	0.016259	0.055455	0.045709	0.146583
0.071342	0.039429	0.024117	0.183828	0.194277	0.016337	0.014049	0.016039	0.025893	0.240256	0.251877	0.017504	0.018110	0.068627	0.036457	0.172756
0.068809	0.021669	0.031978	0.179961	0.187863	0.033553	0.035666	0.022427	0.043516	0.226463	0.237843	0.026811	0.031010	0.066026	0.018727	0.175957
0.097860	0.032177	0.009079	0.214355	0.222593	0.018927	0.017464	0.019448	0.048450	0.266144	0.278406	0.010443	0.033003	0.081649	0.026993	0.210331

0.144678				
0.170550	0.011158			
0.174527	0.028177	0.020774		
0.206588	0.034460	0.015699	0.014257	

	10A.r.s	10A.s	10B.r.s	10B.s	10C.r.s	10C.s	11A.r.s	11A.s	11B.r.s	11B.s	11C.r.s	11C.s	12A.r.s	12A.s	12B.r.s
10A.r.s															
10A.s	0.0133330														
10B.r.s	0.1342350	0.1482364													
10B.s	0.1292599	0.1431600	0.0075750												
10C.r.s	0.1469933	0.1621633	0.0176439	0.0173684											
10C.s	0.1367350	0.1515191	0.0182387	0.0245893	0.0060990										
11A.r.s	0.0637840	0.0607329	0.1470089	0.1422662	0.1503560	0.1389594									
11A.s	0.0648637	0.0569762	0.1546112	0.1487889	0.1598893	0.1494289	0.0140600								
11B.r.s	0.1690074	0.1879494	0.0265033	0.0198409	0.0181735	0.0280521	0.1777757	0.1867137							
11B.s	0.1565082	0.1743509	0.0362147	0.0267932	0.0254710	0.0355274	0.1632625	0.1722531	0.0114142						
11C.r.s	0.1501907	0.1656771	0.0231093	0.0262680	0.0285895	0.0299339	0.1555358	0.1631347	0.0220075	0.0271719					
11C.s	0.1636821	0.1815954	0.0191981	0.0160084	0.0145721	0.0234649	0.1732385	0.1809440	0.0090675	0.0183758	0.0144500				
12A.r.s	0.0554569	0.0486241	0.1425901	0.1391189	0.1578311	0.1467748	0.0674612	0.0717468	0.1825954	0.1688583	0.1590880	0.1771970			
12A.s	0.0599134	0.0533378	0.1495979	0.1456531	0.1646834	0.1530311	0.0745040	0.0781231	0.1879365	0.1741602	0.1642100	0.1814429	0.0160254		
12B.r.s	0.1354897	0.1551082	0.0224867	0.0265021	0.0329131	0.0341295	0.1561528	0.1670940	0.0287167	0.0332657	0.0252490	0.0241791	0.1500941	0.1515131	
12B.s	0.1429558	0.1610559	0.0192550	0.0222010	0.0309579	0.0338599	0.1654994	0.1754978	0.0287739	0.0438590	0.0365350	0.0243501	0.1547391	0.1573560	0.0131671
12C.r.s	0.1335409	0.1486128	0.0224214	0.0272610	0.0170571	0.0152170	0.1326121	0.1435815	0.0355910	0.0410357	0.0334901	0.0271211	0.1441159	0.1506174	0.0326858
12C.s	0.1199534	0.1340027	0.0378071	0.0415125	0.0357511	0.0334657	0.1199288	0.1310914	0.0505928	0.0439870	0.0373598	0.0423171	0.1298689	0.1370401	0.0359091
13A.r.s	0.0898614	0.0792477	0.2198627	0.2124128	0.2192025	0.2048808	0.0647642	0.0695020	0.2514001	0.2337445	0.2249890	0.2475270	0.0800651	0.0883538	0.2271185
13A.s	0.0873408	0.0750034	0.2209624	0.2131110	0.2215058	0.2058240	0.0602979	0.0512785	0.2525999	0.2348821	0.2264081	0.2478610	0.0869247	0.0928694	0.2330620
13B.r.s	0.1845587	0.2034357	0.0293663	0.0251448	0.0226458	0.0341565	0.1948548	0.2025040	0.0168705	0.0355655	0.0356157	0.0151327	0.1993825	0.2052514	0.0329054
13B.s	0.1542277	0.1723757	0.0324424	0.0225210	0.0329108	0.0462070	0.1655381	0.1680587	0.0220781	0.0260621	0.0346200	0.0213551	0.1682377	0.1739361	0.0333548
13C.r.s	0.1124654	0.1236892	0.0401209	0.0424640	0.0362041	0.0303549	0.1045688	0.1151615	0.0579029	0.0659010	0.0587920	0.0494221	0.1167537	0.1211581	0.0499982
13C.s	0.1236727	0.1365640	0.0289720	0.0246251	0.0234721	0.0290387	0.1251315	0.1333975	0.0376910	0.0480980	0.0467851	0.0312907	0.1314209	0.1362360	0.0410910
14A.r.s	0.0637342	0.0549821	0.1896361	0.1835780	0.1983757	0.1851220	0.0593691	0.0634970	0.2289601	0.2112560	0.2025870	0.2233711	0.0644659	0.0648520	0.1951559
14A.s	0.0649995	0.0549450	0.1987109	0.1934111	0.2108590	0.1983589	0.0771130	0.0690159	0.2412314	0.2244565	0.2131517	0.2326148	0.0673030	0.0700764	0.2095671
3A.r.s	0.0402138	0.0405154	0.1274067	0.1227484	0.1443210	0.1336043	0.0653151	0.0721845	0.1657809	0.1521008	0.1459568	0.1603060	0.0493545	0.0522824	0.1282840
3A.s	0.0403358	0.0410402	0.1280779	0.1233492	0.1404310	0.1295914	0.0600650	0.0671414	0.1628011	0.1491724	0.1437354	0.1581490	0.0460138	0.0519980	0.1278672
3B.r.s	0.1499411	0.1711411	0.0590331	0.0454718	0.0569562	0.0704595	0.1756948	0.1854157	0.0439545	0.0442530	0.0666031	0.0480740	0.1581643	0.1613130	0.0570250
3B.s	0.1245605	0.1393451	0.1219725	0.1184409	0.1406021	0.1385501	0.1522557	0.1603475	0.1293887	0.1247693	0.1227488	0.1332021	0.1223369	0.1198125	0.1122034
3C.r.s	0.1780203	0.1960099	0.0332589	0.0358721	0.0221230	0.0235319	0.1903034	0.1993625	0.0228838	0.0376111	0.0389357	0.0251474	0.1892721	0.1955127	0.0509339
3C.s	0.1340257	0.1486015	0.0824169	0.0779938	0.0703015	0.0663808	0.1442629	0.1540807	0.0607024	0.0467309	0.0607059	0.0728319	0.1381838	0.1449564	0.0746445
4A.r.s	0.0471184	0.0437040	0.1654387	0.1589621	0.1756164	0.1628730	0.0504410	0.0476659	0.2031884	0.1875101	0.1793177	0.1964800	0.0636717	0.0693824	0.1741618
4A.s	0.0479649	0.0442155	0.1656778	0.1592844	0.1758250	0.1629171	0.0495118	0.0468399	0.2036574	0.1880034	0.1790245	0.1968481	0.0584567	0.0648765	0.1732925
4B.r.s	0.1597762	0.1798240	0.0229860	0.0266470	0.0267787	0.0289943	0.1801279	0.1877591	0.0300300	0.0499444	0.0448719	0.0272418	0.1760131	0.1805880	0.0401369

12B.s	12C.r.s	12C.s	13A.r.s	13A.s	13B.r.s	13B.s	13C.r.s	13C.s	14A.r.s	14A.s	3A.r.s	3A.s	3B.r.s	3B.s	3C.r.s
0.033815															
0.050148	0.014273														
0.237083	0.194299	0.177330													
0.242313	0.198302	0.183361	0.021528												
0.023094	0.037422	0.062489	0.271177	0.271617											
0.032515	0.047848	0.061967	0.236067	0.233710	0.022036										
0.044274	0.027936	0.046498	0.156929	0.160136	0.058920	0.058799									
0.032908	0.024816	0.044060	0.180587	0.184238	0.034931	0.036251	0.014521								
0.204870	0.176346	0.159254	0.061811	0.058579	0.246325	0.214771	0.143971	0.163948							
0.217110	0.189048	0.171486	0.081959	0.064768	0.258429	0.222462	0.159608	0.174349	0.028991						
0.135702	0.130224	0.116126	0.097512	0.100672	0.182999	0.154458	0.110225	0.122259	0.060880	0.068316					
0.135884	0.127921	0.114807	0.088744	0.093215	0.179432	0.151475	0.106550	0.122065	0.057227	0.074588	0.014819				
0.050061	0.077268	0.090696	0.239692	0.246383	0.052822	0.032657	0.077666	0.060641	0.215740	0.227999	0.143652	0.143050			
0.111954	0.142986	0.140280	0.189285	0.198530	0.151570	0.116746	0.125332	0.134515	0.171884	0.184252	0.118534	0.116539	0.064524		
0.040293	0.036547	0.063405	0.262850	0.263745	0.028792	0.051182	0.057360	0.049330	0.240621	0.251906	0.174974	0.171338	0.061280	0.140752	
0.092019	0.077427	0.070868	0.199328	0.203413	0.097702	0.086281	0.091364	0.095206	0.181216	0.196793	0.127273	0.123718	0.083661	0.110049	0.058154
0.181920	0.156448	0.142761	0.063921	0.052617	0.221310	0.187365	0.125264	0.144998	0.045201	0.048446	0.049221	0.049991	0.190924	0.155080	0.213445
0.181471	0.156000	0.140512	0.059059	0.052065	0.222434	0.187342	0.125318	0.144527	0.046228	0.049648	0.047990	0.048951	0.189259	0.152110	0.214279
0.022631	0.038386	0.068923	0.253452	0.253459	0.026526	0.035820	0.045381	0.037851	0.227744	0.237170	0.158098	0.156099	0.042215	0.112255	0.020602

3C.s	4A.r.s	4A.s	4B.r.s	4B.s	4C.r.s	4C.s	5A.r.s	5A.s	5B.r.s	5B.s	5C.r.s	5C.s	6A.r.s	6A.s	6B.r.s
0.1610289															
0.1605729	0.007087[illegible]														
0.0918509	0.1985195	0.1986836													

6B.s	6C.r.s	6C.s	7A.r.s	7A.s	7B.r.s	7B.s	7C.r.s	7C.s	8A.r.s	8A.s	8B.r.s	8B.s	8C.r.s	8C.s	9A.r.s

9A.s	9B.r.s	9B.s	9C.r.s	9C.s

4B.s	0.162144	0.182500	0.026453	0.029906	0.030111	0.032570	0.183076	0.190899	0.032989	0.051736	0.048098	0.029225	0.176191	0.181353	0.044520
4C.r.s	0.185653	0.205011	0.035827	0.033482	0.019271	0.026015	0.199240	0.207971	0.020929	0.035595	0.043389	0.022125	0.201591	0.208056	0.053374
4C.s	0.142895	0.158037	0.082806	0.078825	0.070776	0.066347	0.150764	0.159642	0.062697	0.047638	0.058386	0.072903	0.150706	0.157787	0.076768
5A.r.s	0.047559	0.042193	0.169595	0.164051	0.180365	0.166213	0.045867	0.050965	0.207686	0.190711	0.183657	0.202965	0.045240	0.045056	0.171156
5A.s	0.045201	0.039626	0.177007	0.170553	0.184842	0.170007	0.042373	0.047623	0.214517	0.196277	0.190055	0.209523	0.046257	0.048383	0.179409
5B.r.s	0.187945	0.209167	0.033300	0.028223	0.021750	0.032302	0.205065	0.214911	0.018934	0.035001	0.045335	0.019359	0.202772	0.208438	0.046729
5B.s	0.189375	0.210462	0.037899	0.032636	0.024472	0.035870	0.207450	0.217077	0.023355	0.040496	0.054527	0.024353	0.205513	0.211777	0.052528
5C.r.s	0.144093	0.163224	0.042283	0.029948	0.033659	0.043669	0.164048	0.173315	0.021852	0.018516	0.038766	0.028332	0.157895	0.161673	0.041797
5C.s	0.112456	0.128036	0.061464	0.058165	0.066985	0.064418	0.132863	0.141018	0.055689	0.043038	0.048042	0.062265	0.120477	0.121625	0.051374
6A.r.s	0.042490	0.040708	0.148340	0.144513	0.161786	0.151174	0.060516	0.055817	0.185308	0.171391	0.165204	0.180058	0.056086	0.059084	0.153866
6A.s	0.039123	0.038955	0.153445	0.149170	0.170303	0.158665	0.066376	0.060889	0.191484	0.176959	0.171883	0.187113	0.047463	0.053347	0.157519
6B.r.s	0.127582	0.144614	0.039395	0.026196	0.032956	0.043833	0.145064	0.153009	0.026025	0.024211	0.045398	0.030193	0.139394	0.144499	0.041315
6B.s	0.085226	0.096308	0.070725	0.067873	0.080806	0.075567	0.097701	0.109743	0.077701	0.068527	0.066715	0.081188	0.087164	0.090408	0.061030
6C.r.s	0.171509	0.190757	0.056316	0.044662	0.037065	0.046785	0.185706	0.194633	0.023022	0.015570	0.040462	0.033227	0.183644	0.190464	0.052792
6C.s	0.201359	0.221744	0.037535	0.035806	0.022186	0.031192	0.217297	0.226054	0.021564	0.037875	0.046124	0.021932	0.217247	0.223473	0.053139
7A.r.s	0.067170	0.057651	0.178074	0.170315	0.182555	0.170096	0.041570	0.032352	0.210642	0.193969	0.186391	0.205078	0.075335	0.081941	0.189662
7A.s	0.069622	0.059830	0.186986	0.179127	0.189837	0.176044	0.040530	0.034424	0.220033	0.202594	0.194314	0.214138	0.076896	0.083268	0.199858
7B.r.s	0.157639	0.176314	0.016855	0.019972	0.019153	0.022160	0.170530	0.177538	0.023021	0.036657	0.030801	0.018729	0.173631	0.176557	0.032809
7B.s	0.169631	0.189095	0.026183	0.020499	0.019592	0.031233	0.185425	0.191176	0.020457	0.033024	0.042936	0.016751	0.188004	0.191997	0.040356
7C.r.s	0.162579	0.181846	0.027463	0.020906	0.021154	0.030801	0.179339	0.189545	0.010877	0.015469	0.030307	0.015628	0.174816	0.180038	0.031894
7C.s	0.137994	0.155993	0.036263	0.024491	0.039885	0.051867	0.158464	0.167455	0.028607	0.027404	0.044368	0.030620	0.151440	0.155347	0.029953
8A.r.s	0.112887	0.099698	0.236377	0.227387	0.232707	0.218098	0.064199	0.066290	0.265531	0.247721	0.238244	0.262407	0.093766	0.099419	0.247366
8A.s	0.114977	0.101634	0.247173	0.238441	0.244098	0.229304	0.069346	0.069899	0.277300	0.258445	0.249114	0.274161	0.096989	0.102978	0.257399
8B.r.s	0.180736	0.198567	0.027524	0.022100	0.015729	0.027871	0.191122	0.197585	0.013254	0.026048	0.033948	0.013295	0.197148	0.201862	0.042779
8B.s	0.158499	0.178335	0.037051	0.026653	0.029337	0.040568	0.169924	0.180346	0.022289	0.025332	0.041989	0.024211	0.171908	0.176856	0.035630
8C.r.s	0.128457	0.142475	0.058141	0.054904	0.067525	0.064930	0.131007	0.137576	0.063981	0.055350	0.053457	0.061905	0.138388	0.146980	0.044143
8C.s	0.165474	0.184237	0.046605	0.039579	0.030075	0.037543	0.173762	0.183456	0.018267	0.011368	0.033372	0.025828	0.178776	0.186238	0.045056
9A.r.s	0.045330	0.042254	0.166656	0.160655	0.176535	0.164045	0.052712	0.056922	0.205365	0.189571	0.181613	0.200308	0.050497	0.055959	0.172216
9A.s	0.051220	0.039420	0.164783	0.159864	0.174865	0.162763	0.053571	0.056590	0.203835	0.188375	0.179953	0.198007	0.047831	0.051506	0.171204
9B.r.s	0.121481	0.136792	0.026977	0.019535	0.023258	0.029932	0.132573	0.142889	0.030062	0.031740	0.038779	0.026422	0.132458	0.138487	0.033647
9B.s	0.141545	0.158739	0.018217	0.013815	0.011414	0.017953	0.154479	0.165083	0.018646	0.026863	0.034483	0.016377	0.153960	0.159777	0.033038
9C.r.s	0.147311	0.162610	0.033715	0.029943	0.014860	0.017780	0.149859	0.159933	0.018762	0.019424	0.028400	0.023429	0.155608	0.162392	0.041458
9C.s	0.173903	0.190696	0.025871	0.025851	0.009643	0.015199	0.181747	0.190732	0.016580	0.028367	0.034347	0.015961	0.186790	0.192858	0.042915

0.024234	0.041839	0.072715	0.255353	0.255520	0.028194	0.037970	0.048287	0.040572	0.231766	0.240457	0.161116	0.158715	0.040760	0.108465	0.020099
0.041848	0.036373	0.065332	0.271980	0.271588	0.021544	0.045375	0.060833	0.045625	0.247312	0.258584	0.184697	0.180724	0.059323	0.154123	0.006423
0.098114	0.073406	0.066897	0.205731	0.206541	0.096992	0.087646	0.094549	0.097234	0.182980	0.199639	0.138959	0.133535	0.096975	0.132486	0.064843
0.178445	0.163242	0.148894	0.054066	0.057427	0.228921	0.192453	0.124921	0.148637	0.038703	0.058259	0.038914	0.036902	0.181793	0.130381	0.218685
0.187737	0.165609	0.149415	0.048395	0.047520	0.235785	0.200250	0.126225	0.150419	0.034261	0.053835	0.044941	0.042300	0.193314	0.145250	0.225501
0.033798	0.042485	0.071724	0.280622	0.282581	0.016473	0.032071	0.061777	0.043940	0.255855	0.267942	0.184162	0.180894	0.038826	0.135875	0.014735
0.035067	0.046368	0.076939	0.282020	0.283816	0.018131	0.037825	0.064084	0.044919	0.258780	0.269127	0.187612	0.184580	0.044343	0.145331	0.016448
0.046298	0.051764	0.060200	0.232258	0.234746	0.041421	0.028352	0.068047	0.051751	0.204797	0.216041	0.138803	0.137946	0.022024	0.097095	0.037632
0.069269	0.069633	0.061241	0.189713	0.192116	0.090244	0.065720	0.081617	0.082790	0.161723	0.174822	0.104474	0.104231	0.056498	0.064806	0.068895
0.158105	0.148891	0.136909	0.085083	0.076835	0.203456	0.168466	0.123526	0.140427	0.058277	0.062627	0.045073	0.044244	0.165559	0.129660	0.194216
0.160966	0.154790	0.139521	0.085344	0.075999	0.210238	0.171881	0.131341	0.145068	0.059569	0.053480	0.042489	0.045476	0.164246	0.121876	0.202369
0.041257	0.049242	0.060127	0.210556	0.212142	0.040059	0.023768	0.056867	0.040197	0.185345	0.197090	0.123660	0.122353	0.017954	0.093878	0.046290
0.074074	0.077801	0.070119	0.146340	0.154743	0.108818	0.080786	0.073435	0.082433	0.119812	0.137266	0.071912	0.072196	0.067120	0.054182	0.095400
0.063910	0.056270	0.060069	0.255164	0.256921	0.047353	0.044338	0.085381	0.066890	0.230615	0.243173	0.167954	0.164906	0.050862	0.138865	0.036666
0.041703	0.041098	0.070205	0.292806	0.292877	0.019652	0.044870	0.068584	0.050022	0.268046	0.278352	0.199904	0.196538	0.060394	0.162385	0.010453
0.198868	0.163479	0.152690	0.050617	0.031655	0.228294	0.193102	0.130429	0.151620	0.048260	0.056942	0.072556	0.066847	0.205947	0.169883	0.222274
0.208676	0.170289	0.158189	0.049401	0.030735	0.237599	0.203220	0.135303	0.157972	0.049466	0.057641	0.077695	0.071977	0.217180	0.179670	0.231632
0.023188	0.027821	0.053157	0.243299	0.241971	0.023203	0.030283	0.041208	0.031760	0.217510	0.227273	0.155859	0.153354	0.050379	0.121133	0.019807
0.027021	0.035038	0.061938	0.258243	0.257070	0.016054	0.021822	0.048382	0.028650	0.233202	0.242341	0.168656	0.166112	0.040865	0.136162	0.026741
0.029588	0.038267	0.051457	0.252289	0.256436	0.026498	0.028767	0.061881	0.044442	0.223783	0.237427	0.155996	0.153827	0.035899	0.114554	0.020628
0.029055	0.052278	0.059783	0.228175	0.233626	0.038091	0.018697	0.063440	0.043848	0.195716	0.210328	0.128991	0.127972	0.024132	0.095660	0.060453
0.257766	0.208027	0.194145	0.046947	0.043799	0.285212	0.252255	0.161763	0.189417	0.066519	0.087310	0.114421	0.106177	0.261619	0.208431	0.276575
0.268061	0.217315	0.201660	0.046873	0.043931	0.296769	0.263040	0.171817	0.199925	0.066829	0.087266	0.119557	0.110001	0.271361	0.213544	0.288712
0.033711	0.033995	0.060566	0.265725	0.263580	0.011272	0.026354	0.059876	0.035450	0.241235	0.250699	0.179259	0.175988	0.051644	0.152098	0.017889
0.034684	0.045144	0.060884	0.239528	0.243269	0.027613	0.017182	0.055243	0.038704	0.217888	0.232465	0.154867	0.151380	0.027336	0.117034	0.047446
0.060837	0.057717	0.052846	0.187746	0.189132	0.078738	0.054892	0.059151	0.059767	0.171609	0.187656	0.124794	0.121738	0.088129	0.121735	0.096121
0.054500	0.044603	0.047996	0.242628	0.243569	0.042608	0.042488	0.069964	0.055239	0.222690	0.235761	0.163835	0.160468	0.058533	0.135898	0.033277
0.180370	0.157911	0.143507	0.058160	0.059672	0.224229	0.192456	0.127790	0.146590	0.034979	0.052992	0.039273	0.038258	0.188539	0.149031	0.216233
0.177242	0.158411	0.145291	0.066334	0.064514	0.220956	0.191326	0.130472	0.148408	0.038354	0.054569	0.039890	0.039789	0.188225	0.151485	0.210786
0.032730	0.034816	0.048201	0.197935	0.200441	0.034722	0.022262	0.039018	0.027543	0.170291	0.185145	0.114799	0.112569	0.031703	0.104388	0.049047
0.025422	0.027016	0.047907	0.224264	0.226462	0.022889	0.026047	0.038166	0.025947	0.197629	0.211016	0.136008	0.133793	0.035633	0.116473	0.024493
0.049162	0.030067	0.040615	0.215378	0.217413	0.037648	0.038451	0.048862	0.040403	0.195161	0.208853	0.142110	0.137816	0.055665	0.132189	0.025844
0.037008	0.025930	0.051042	0.254030	0.254154	0.019200	0.037915	0.048568	0.034424	0.231206	0.242522	0.171321	0.167326	0.059929	0.152240	0.011157

0.092425	0.201721	0.201384	0.003141												
0.071062	0.221889	0.223340	0.023334	0.023143											
0.013574	0.167598	0.168180	0.097176	0.100127	0.067901										
0.156030	0.034932	0.033374	0.201127	0.202783	0.230321	0.168032									
0.160349	0.032194	0.032594	0.210252	0.212339	0.236954	0.172098	0.012259								
0.084189	0.228906	0.229440	0.014572	0.015130	0.010144	0.087983	0.232702	0.240616							
0.092399	0.229741	0.230384	0.016778	0.015862	0.010610	0.098283	0.234701	0.242499	0.005161						
0.049108	0.181643	0.181299	0.039126	0.040604	0.036312	0.054423	0.179781	0.186866	0.026700	0.034113					
0.029626	0.144168	0.142527	0.072216	0.073790	0.078886	0.035495	0.134290	0.142799	0.075190	0.090042	0.031951				
0.143525	0.039654	0.039298	0.176413	0.178274	0.204166	0.154949	0.039747	0.041793	0.206015	0.207802	0.159080	0.120249			
0.148428	0.043959	0.041571	0.181639	0.182259	0.211934	0.160841	0.041507	0.042523	0.212116	0.213394	0.162730	0.120847	0.015515		
0.062267	0.160062	0.159053	0.039251	0.040290	0.043617	0.070481	0.157560	0.166275	0.032234	0.037902	0.012117	0.044584	0.137797	0.141912	
0.054824	0.102272	0.100507	0.087393	0.089152	0.106912	0.067043	0.086965	0.098265	0.102160	0.112787	0.059662	0.028197	0.082299	0.085950	0.047807
0.036783	0.208339	0.208253	0.059633	0.062579	0.033747	0.035439	0.212878	0.216680	0.037063	0.044310	0.017459	0.044863	0.189426	0.195578	0.031820
0.084003	0.242208	0.243077	0.022263	0.023350	0.005306	0.083708	0.251011	0.256905	0.006471	0.008545	0.037906	0.085387	0.221607	0.228715	0.047101
0.171489	0.033652	0.033686	0.210806	0.213610	0.230616	0.175271	0.042472	0.037375	0.239040	0.240560	0.193549	0.156709	0.055260	0.059581	0.171359
0.178359	0.038075	0.038703	0.221932	0.224308	0.240083	0.183074	0.046211	0.037526	0.249388	0.250664	0.202828	0.165217	0.056947	0.063329	0.182063
0.081242	0.191411	0.192191	0.009312	0.010864	0.021179	0.084105	0.195826	0.202800	0.016315	0.018469	0.036272	0.065202	0.171438	0.177573	0.037209
0.094664	0.205541	0.206423	0.014464	0.015902	0.019975	0.096084	0.213191	0.219694	0.010798	0.011325	0.032189	0.080898	0.186232	0.192493	0.030981
0.056478	0.200791	0.201336	0.025632	0.027281	0.019863	0.061040	0.199543	0.207517	0.015804	0.019881	0.015833	0.044563	0.177201	0.182052	0.023360
0.081281	0.175029	0.175409	0.039215	0.043039	0.055906	0.084694	0.170324	0.177953	0.038538	0.044022	0.021040	0.052123	0.151438	0.154356	0.018834
0.214471	0.073240	0.072462	0.274443	0.276047	0.285353	0.220575	0.065538	0.054436	0.297388	0.299383	0.250170	0.209863	0.102470	0.106118	0.229303
0.223755	0.074320	0.073585	0.285512	0.287194	0.297395	0.228202	0.068221	0.055420	0.309590	0.311642	0.260976	0.217681	0.105673	0.107906	0.239732
0.084398	0.215147	0.216289	0.024127	0.025123	0.011004	0.083103	0.224508	0.230109	0.010725	0.012007	0.031576	0.080794	0.197599	0.205512	0.033596
0.082258	0.193586	0.193728	0.032628	0.036647	0.040779	0.080507	0.194539	0.201079	0.026254	0.031569	0.019489	0.064287	0.175201	0.180928	0.019514
0.085270	0.149962	0.148071	0.081406	0.086095	0.099060	0.078164	0.153052	0.154618	0.092580	0.102158	0.070734	0.061315	0.142151	0.145866	0.068456
0.039235	0.197852	0.197942	0.057414	0.057183	0.031378	0.038540	0.204695	0.208374	0.040108	0.043692	0.026964	0.047886	0.183626	0.189544	0.036673
0.159993	0.030661	0.032537	0.201195	0.204144	0.224852	0.167300	0.021746	0.022324	0.230286	0.231705	0.181488	0.141122	0.041736	0.045737	0.160766
0.154711	0.034165	0.037556	0.198964	0.202109	0.220909	0.163632	0.023803	0.026564	0.226730	0.228695	0.179461	0.141320	0.041003	0.048414	0.158364
0.078811	0.146587	0.147950	0.033519	0.038355	0.046466	0.081309	0.147143	0.154062	0.034824	0.041816	0.026116	0.058266	0.134891	0.141683	0.018747
0.075425	0.172707	0.173695	0.016883	0.020052	0.022436	0.079124	0.174127	0.181510	0.015127	0.017850	0.022088	0.061363	0.156580	0.162896	0.020868
0.043380	0.173444	0.173862	0.041949	0.046068	0.025721	0.042988	0.176565	0.182002	0.031794	0.036877	0.024635	0.050000	0.162376	0.168940	0.031450
0.069964	0.207093	0.208135	0.023570	0.026175	0.007609	0.068494	0.214379	0.220455	0.013294	0.015078	0.033604	0.073690	0.192247	0.199780	0.039815

0.0820640															
0.1178610	0.0337750														
0.1188327	0.2191949	0.2514062													
0.1287490	0.2271514	0.2605030	0.0083235												
0.0814859	0.0505819	0.0205453	0.2000242	0.2095698											
0.1001435	0.0453802	0.0168804	0.2143750	0.2238091	0.0098984										
0.0695810	0.0214111	0.0195129	0.2122613	0.2220827	0.0208253	0.0203552									
0.0610383	0.0414177	0.0547949	0.1901000	0.1996831	0.0371447	0.0291988	0.0225297								
0.1634941	0.2685789	0.3065687	0.0506349	0.0450439	0.2590565	0.2741587	0.2687529	0.2485515							
0.1703517	0.2797837	0.3191531	0.0522528	0.0488950	0.2710635	0.2860272	0.2804260	0.2584655	0.0079059						
0.1044157	0.0346788	0.0102263	0.2213438	0.2300647	0.0163057	0.0109797	0.0182131	0.0391235	0.2777431	0.2898214					
0.0792438	0.0344994	0.0395355	0.2023440	0.2116143	0.0313469	0.0200042	0.0256277	0.0148280	0.2577725	0.2687429	0.0262361				
0.0688432	0.0764125	0.1013419	0.1543738	0.160563	0.0677467	0.0738232	0.0728780	0.0515135	0.1994484	0.2087498	0.0831241	0.0557383			
0.0785082	0.0132089	0.0361314	0.2044717	0.2118199	0.0449843	0.0420934	0.0209448	0.0452859	0.2530479	0.2648942	0.0314202	0.0382843	0.0612280		
0.0971637	0.2094594	0.2452655	0.0442332	0.0498382	0.1951491	0.2106082	0.1993122	0.1710710	0.0728352	0.0744093	0.2193034	0.1949740	0.1544960	0.2023010	
0.0973052	0.2056658	0.2404005	0.0485909	0.0526013	0.1933769	0.2079190	0.1972900	0.1704549	0.0763281	0.0798448	0.2150290	0.1930345	0.1572730	0.2009448	0.0133178
0.0597331	0.0475984	0.0500404	0.1593514	0.1691618	0.0344150	0.0282293	0.0307253	0.0199429	0.2137770	0.2244314	0.0346160	0.0162592	0.0554550	0.0457090	0.1465833
0.0713422	0.0394290	0.0241175	0.1838288	0.1942771	0.0163371	0.0140491	0.0160392	0.0258931	0.2402560	0.2518771	0.0175042	0.0181105	0.0686275	0.0364572	0.1727565
0.0688097	0.0216698	0.0319783	0.1799615	0.1878637	0.0335530	0.0356663	0.0224272	0.0435162	0.2264639	0.2378439	0.0268113	0.0310107	0.0660262	0.0187273	0.1759575
0.0978607	0.0321775	0.0090791	0.2143552	0.2225938	0.0189279	0.0174640	0.0194484	0.0484500	0.2661442	0.2784069	0.0104430	0.0330037	0.0816491	0.0269931	0.2103310

0.144678[illegible]				
0.170550[illegible]	0.011158[illegible]			
0.174527[illegible]	0.028177[illegible]	0.020774[illegible]		
0.206588[illegible]	0.034460[illegible]	0.015699[illegible]	0.014257[illegible]	

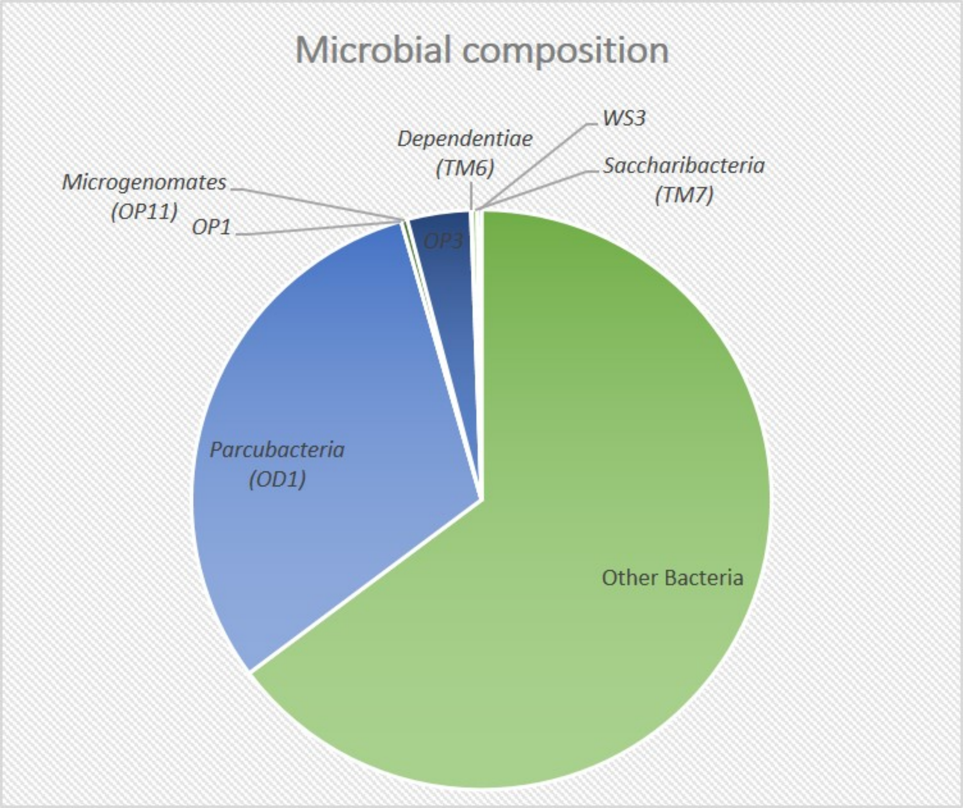
Microbial composition
WS3
Dependentiae
(TM6)
Saccharibacteria
(TM7)
Microgenomates
(OP11)
OP1
OP3
Parcubacteria
(OD1)
Other Bacteria

Fig. S1

Bacterial composition of water samples. See Table S2 reporting the relative abundance of the bacteria recovered.

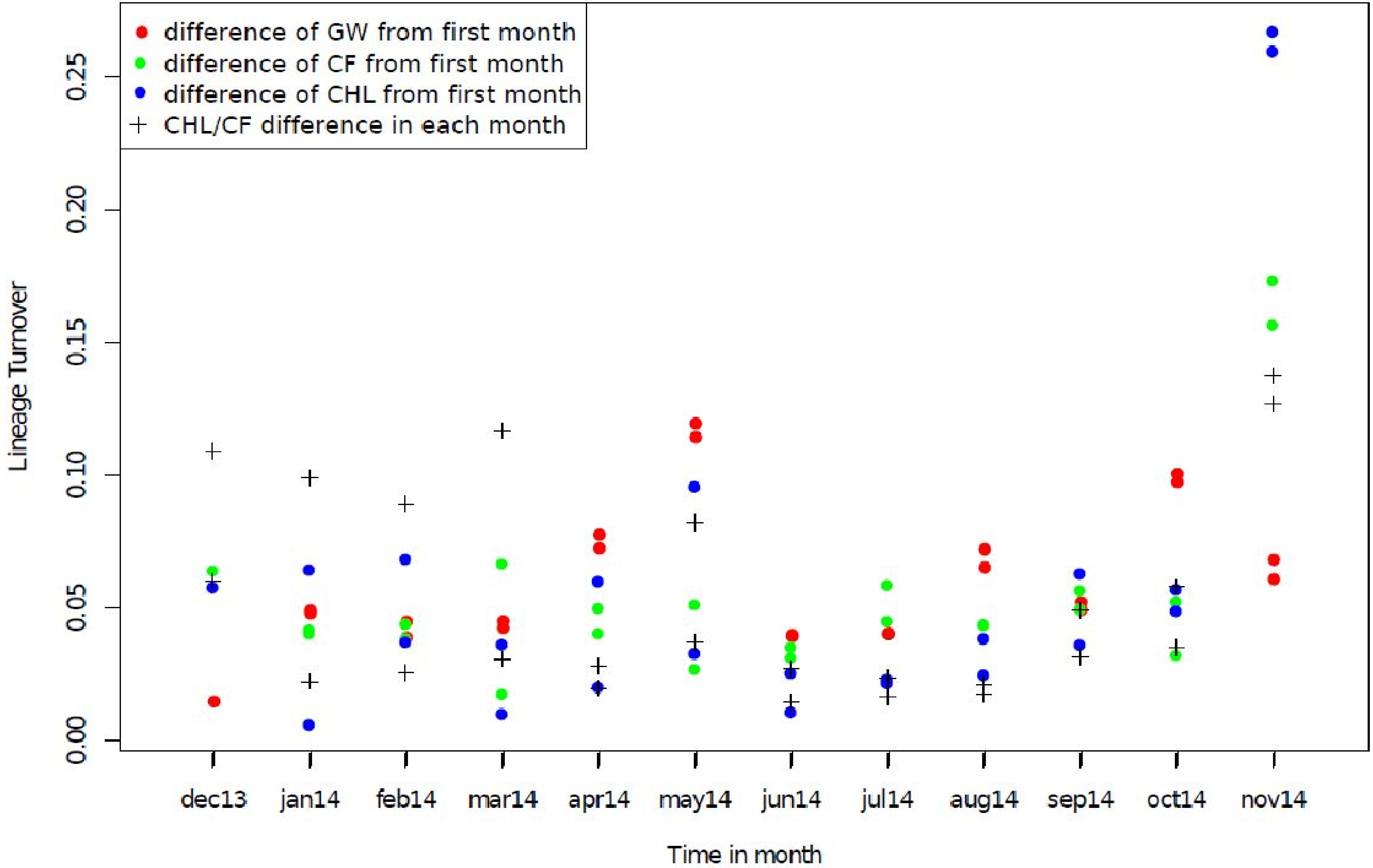
difference of GW from first month
difference of CF from first month
difference of CHL from first month
CHL/CF difference in each month
Lineage Turnover
0.00
0.05
0.10
0.15
0.20
0.25
dec13
jan14
feb14
mar14
apr14
may14
jun14
jul14
aug14
sep14
oct14
nov14
Time in month

Fig. S2

Change over time and sampling points within a DWTP (Site 1), expressed as *turnover*. The three series of circles show the change with respect to the first sample in December 2013 of their respective technical replica and the other sampling time points. Red, green, and blue circles indicate GW, CF, and CHL compartments, respectively. The cross indicates a comparison for each replica and each time between compartments CF and CHL. Notice that the samples from November 2014 are different from others in CF and CHL compartments (green and blue circles), due to the renewal of carbon filters in the DWTP. Notice that, excluding November 2014, variation across compartments CF and CHL and variation within the three compartments across time are similar and comparable to variation across replicas, indicating a seeding effect of CF on CHL and a lack of a strong temporal trend. This seeding effect is present in the last sample given that differences are smaller across compartment than across time in CHL (blue circle); in November 2014, CHL sample is more different from CF than in the other sampling time points (see also Fig. 1).

output_phyloh_site1

Partitioning information in /home/anna/Scrivania/openw/progetto_acqua/16S_merge_work/phyloH.raxml/

Table of Contents

The run call was:

esecutorePhyloHPandas.py -s /home/anna/Scrivania/openw/progetto_acqua/16S_merge_work/phyloH.raxml/paper.phylo.20.11/otu_paper.sample.txt -t /home/anna/Scrivania/openw/progetto_acqua/16S_merge_work/phyloH.raxml/paper.phylo.20.11/nanoarchea_taxa_mod.txt -f /home/anna/Scrivania/openw/progetto_acqua/16S_merge_work/phyloH.raxml/paper.phylo.20.11/nanoarchea.lb.rm.reroot_newick.txt -g /home/anna/Scrivania/openw/progetto_acqua/16S_merge_work/phyloH.raxml/paper.phylo.20.11/map_h2o_M_cre.fil.rm.txt -r 999 -h 1 -q 1 -e 1 -k 1 -o /home/anna/Scrivania/openw/progetto_acqua/16S_merge_work/phyloH.raxml/paper.phylo.20.11/nano_arche.lb.cre.fil.rm.samP.999.e1.k1

Experimental Design:

Counts of observations across groups and samples within groups

Total Counts	Group Name	Group Counts	Sample Name	Sample Counts
4041318	Aquifer	534149	10A.r.s	62411
			10A.s	27577
			11A.r.s	1795
			11A.s	14254
			12A.r.s	87148
			12A.s	8342
			13A.r.s	51516
			13A.s	20891
			14A.r.s	14924
			14A.s	7225
			3A.r.s	251395
			3A.s	7622
			4A.r.s	21877
			4A.s	84514
			5A.r.s	283518
			5A.s	5704
			6A.r.s	73243
			6A.s	22480
			7A.r.s	27784
			7A.s	12747
			8A.r.s	8572
			8A.s	202073
			9A.r.s	10728
			9A.s	17584
	Cfilters	1862664	10B.r.s	33918
			10B.s	88003
			11B.r.s	5133
			11B.s	8683
			12B.r.s	12796
			12B.s	196761
			13B.r.s	80259
			13B.s	43550
			3B.r.s	151853
			3B.s	101531
			4B.r.s	14119
			4B.s	25605
			5B.r.s	64882
			5B.s	58469
			6B.r.s	102777
			6B.s	2654
			7B.r.s	19608
			7B.s	85755
			8B.r.s	14488
			8B.s	20192
			9B.r.s	3972
			9B.s	18927
	Chlor	1644505	10C.r.s	1106

			**10C.s**	**9801**
			**11C.r.s**	**20449**
			**11C.s**	**56553**
			**12C.r.s**	**22187**
			**12C.s**	**48275**
			**13C.r.s**	**13944**
			**13C.s**	**228900**
			**3C.r.s**	**100190**
			**3C.s**	**21846**
			**4C.r.s**	**7657**
			**4C.s**	**2580**
			**5C.r.s**	**53103**
			**5C.s**	**22565**
			**6C.r.s**	**612596**
			**6C.s**	**212195**
			**7C.r.s**	**46101**
			**7C.s**	**34101**
			**8C.r.s**	**4789**
			**8C.s**	**14021**
			**9C.r.s**	**18105**
			**9C.s**	**10395**

Entropy across samples, groups and samples within groups

MaxDiversity gives the values for a maximally balanced experimental design

	nats	Diversity	MaxDiversity
**H(G)**	1.1	3.0	3.0
**H(S)**	4.22	68.0	68.0
**H(S|G)**	3.12	22.7	22.7

Gamma diversities:

Total entropy and diversity within each group and overall data

Unit measure for Diversity is equivalent number of independent equi-abundant linneages

	nats	Diversity
**Group**		
**Aquifer**	0.798	2.22
**Cfilters**	0.435	1.54
**Chlor**	0.451	1.57
**Overall**	0.677	1.97

Alpha diversities:

Mean entropy and diversity within sample or group

Unit measure for Diversity is equivalent number of independent equi-abundant linneages

	nats	Diversity
**H(T|S)**	0.496	1.64
**H(T|G)**	0.568	1.77

Beta diversity:

Information shared across Tree and Sample or Group vector expressed as nats and turnover of linneage.

Turnover is the percentage of observations not shared across groups

Pvalue is computed with Permutation procedure

	nats	TurnOver	pvalue
**I(T,G)**	0.109	9.96%	0.0
**I(T,S|G)**	0.0718	2.3%	0.0

Difference of each group from total:

phylogenetic Kullback-Leiber distance between each group and the overall data

	KullBack-Lieber(PG(i)||Ptot(i)
**Group**	
**Aquifer**	0.194
**Cfilters**	0.0582
**Chlor**	0.0573

Pairwise TurnOver between groups

	Aquifer	Cfilters	Chlor
**Aquifer**			
**Cfilters**	14.0%		
**Chlor**	12.6%	0.573%	

Per Node Statistics mapped on the phylogeny

Three types of data are shown on the tree:

1. The color of the branches cyan indicates a contribution to I(T,E) higher than the null distribution, while branches are black otherwise.
2. The background of each branch is a gradient from yellow to red for increased contribution to I(T,E). For details look at the legend on the side
3. Bar plot on each tips indicates the relative frequencies in each group

Look at the tree find an relevant branches and text search the label of the branch to access the correct row on the by node statistics table.

Go itol using the link to modify the tree, or use the itol table and the labelled tree to add further data set (i.e. taxonomic name)

[Click here to modify image]

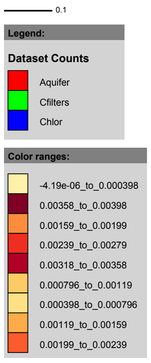

Per Node Statistics in tabular format:

		Metric	I(Ti,G)				By Group Relative Frequency			I(Ti,S|G)	
		Stat	nats	TurnOver	pvalue	MultTest	Aquifer	Cfilters	Chlor	nats	TurnOver
Name	Is_Leaf	Taxonomy									
L575	False	Bacteria	0.00437	0.398%	0.0	1	0.518	0.0543	0.0812	0.00069	0.0221%
L420	False	Bacteria	0.00394	0.359%	0.0	1	0.586	0.0738	0.103	0.000581	0.0186%
OTU_6998	True	ZB2	0.00375	0.341%	0.0	1	0.000644	0.0416	0.186	0.00298	0.0955%
L526	False	Bacteria	0.00375	0.341%	0.0	1	0.526	0.055	0.0844	0.000611	0.0196%
L1372	False	OD1	0.00305	0.278%	0.0	1	0.0426	0.683	0.576	0.000622	0.0199%
L422	False	Bacteria	0.00301	0.274%	0.0	1	0.568	0.0611	0.0919	0.000435	0.0139%
L1326	False	OD1	0.00292	0.266%	0.0	1	0.0929	0.741	0.777	0.000342	0.0109%
L527	False	Bacteria	0.00265	0.241%	0.0	1	0.525	0.055	0.0844	0.000432	0.0139%
L1324	False	OD1	0.00261	0.237%	0.0	1	0.13	0.748	0.78	0.000335	0.0107%
L1379	False	ZB2	0.0026	0.237%	0.0	1	0.0147	0.599	0.456	0.000866	0.0277%
L1375	False	OD1	0.00257	0.234%	0.0	1	0.0159	0.599	0.456	0.00087	0.0279%
L1331	False	OD1	0.00252	0.23%	0.0	1	0.0917	0.741	0.777	0.00029	0.00928%
L1363	False	OD1	0.00236	0.215%	0.0	1	0.0709	0.73	0.766	0.000258	0.00825%
L1325	False	OD1	0.00208	0.19%	0.0	1	0.112	0.745	0.779	0.000252	0.00808%
L421	False	Bacteria	0.00196	0.179%	0.0	1	0.579	0.0733	0.102	0.000303	0.00971%
L921	False	koll11	0.00179	0.163%	0.0	1	0.226	0.0249	0.0375	0.000367	0.0117%
L1328	False	OD1	0.00176	0.16%	0.0	1	0.0919	0.741	0.777	0.000203	0.00649%
L1391	False	ZB2	0.0014	0.127%	0.0	1	0.013	0.369	0.323	0.00127	0.0408%
L1315	False	OD1	0.00138	0.126%	0.0	1	0.235	0.762	0.789	0.000314	0.0101%
L612	False	OP3	0.00125	0.113%	0.0	1	0.478	0.0515	0.0792	0.000246	0.00787%
L1350	False	OD1	0.00122	0.111%	0.0	1	0.0861	0.74	0.775	0.000139	0.00445%
L1330	False	OD1	0.00114	0.104%	0.0	1	0.0918	0.741	0.777	0.000131	0.00421%
L1323	False	OD1	0.00114	0.103%	0.0	1	0.224	0.761	0.789	0.000244	0.00781%
L920	False	koll11	0.00111	0.101%	0.0	1	0.239	0.0263	0.0394	0.000187	0.00598%
L576	False	Bacteria	0.0011	0.1%	0.0	1	0.514	0.0543	0.0809	0.000173	0.00553%
L1373	False	OD1	0.00106	0.0966%	0.0	1	0.0163	0.599	0.456	0.00036	0.0115%
L914	False	OP3	0.00106	0.0963%	0.0	1	0.24	0.0264	0.0394	0.000174	0.00557%
L1381	False	ZB2	0.00104	0.0949%	0.0	1	0.0147	0.599	0.455	0.000347	0.0111%
L1332	False	OD1	0.00102	0.0933%	0.0	1	0.0905	0.741	0.776	0.000117	0.00376%
L973	False	koll11	0.000971	0.0885%	0.0	1	0.169	0.0197	0.0267	0.000207	0.00663%
L983	False	koll11	0.000905	0.0825%	0.0	1	0.159	0.0183	0.0258	0.000196	0.00629%
L1386	False	ZB2	0.00089	0.0811%	0.0	1	0.00169	0.222	0.129	0.0012	0.0385%
L1587	False	ZB2	0.00073	0.0665%	0.0	1	0.000808	0.0416	0.186	0.000585	0.0187%
L1407	False	ZB2	0.000705	0.0642%	0.0	1	0.00479	0.303	0.291	0.000817	0.0262%
L1768	False	OD1	0.000639	0.0582%	0.0	1	0.0323	0.00459	0.00215	0.000843	0.027%
L1383	False	ZB2	0.000626	0.057%	0.0	1	0.0147	0.594	0.454	0.000217	0.00695%
L840	False	OP3	0.000602	0.0548%	0.0	1	0.0655	0.00615	0.00876	0.00025	0.008%
L620	False	OP3	0.000571	0.052%	0.0	1	0.478	0.0515	0.0792	0.000114	0.00364%
L1401	False	ZB2	0.00055	0.0501%	0.0	1	0.00777	0.366	0.321	0.000471	0.0151%
L1393	False	ZB2	0.000536	0.0488%	0.0	1	0.0123	0.369	0.323	0.000483	0.0155%
L1370	False	OD1	0.000517	0.0471%	0.0	1	0.0701	0.687	0.578	0.000124	0.00398%
L706	False	OP3	0.000513	0.0467%	0.0	1	0.115	0.0118	0.0212	0.000253	0.00811%
OTU_7148	True	OD1	0.000505	0.046%	0.0	1	0.03	0.00436	0.00202	0.000781	0.025%
L861	False	koll11	0.000485	0.0442%	0.0	1	0.0263	0.00313	0.00442	0.000176	0.00564%
L622	False	OP3	0.000454	0.0414%	0.0	1	0.145	0.0159	0.0266	0.000165	0.00529%
L990	False	koll11	0.000449	0.0409%	0.0	1	0.141	0.0157	0.0246	8.54e-05	0.00274%
L938	False	koll11	0.000447	0.0407%	0.0	1	0.221	0.0247	0.037	9.11e-05	0.00292%
L1371	False	OD1	0.000439	0.04%	0.0	1	0.0694	0.687	0.578	0.000106	0.00339%
L925	False	koll11	0.000433	0.0395%	0.0	1	0.225	0.0249	0.0375	8.96e-05	0.00287%
L443	False	OP11	0.000429	0.0391%	0.0	1	0.039	0.00528	0.0051	0.00021	0.00672%
L458	False	OP11-4	0.000426	0.0388%	0.0	1	0.0345	0.00504	0.00409	0.000225	0.00721%
L621	False	OP3	0.000418	0.0381%	0.0	1	0.237	0.0252	0.0398	0.000119	0.0038%
L1388	False	ZB2	0.000398	0.0363%	0.0	1	0.00168	0.221	0.128	0.000549	0.0176%

**OTU_6187**	**True**	**ZB2**	0.00037	0.0337%	0.0	1	0.000158	0.015	0.0245	0.000729	0.0234%
**OTU_65**	**True**	**OD1**	0.000365	0.0332%	0.0	1	0.00607	0.000764	0.000195	0.000437	0.014%
**L838**	**False**	**OP3**	0.000341	0.031%	0.0	1	0.0658	0.00615	0.00876	0.000141	0.00452%
**L644**	**False**	**OP3**	0.000331	0.0302%	0.0	1	0.134	0.0142	0.0261	0.000137	0.0044%
**L927**	**False**	**koll11**	0.000328	0.0299%	0.0	1	0.224	0.0249	0.0375	6.84e-05	0.00219%
**L783**	**False**	**OP3**	0.000326	0.0297%	0.0	1	0.0504	0.00501	0.0112	0.000236	0.00757%
**L1403**	**False**	**ZB2**	0.00032	0.0291%	0.0	1	0.000597	0.0635	0.0306	0.000553	0.0177%
**L929**	**False**	**koll11**	0.000312	0.0285%	0.0	1	0.224	0.0248	0.037	6.38e-05	0.00204%
**L707**	**False**	**PBS-25**	0.000309	0.0281%	0.0	1	0.051	0.00565	0.00829	0.000217	0.00694%
**L1384**	**False**	**ZB2**	0.000308	0.0281%	0.0	1	0.00172	0.225	0.13	0.000398	0.0128%
**L577**	**False**	**PRR-12**	0.000306	0.0279%	0.0	1	0.0354	0.00278	0.00165	0.000164	0.00526%
**L1680**	**False**	**OD1**	0.000304	0.0277%	0.0	1	0.0178	0.00273	0.00139	0.000132	0.00422%
**L1431**	**False**	**ZB2**	0.000302	0.0275%	0.0	1	0.00589	0.0509	0.079	0.000263	0.00842%
**OTU_5414**	**True**	**ZB2**	0.000298	0.0272%	0.0	1	0.00356	0.267	0.211	0.000539	0.0173%
**L173**	**False**	**SM2F11**	0.000292	0.0266%	0.0	1	0.0117	0.00198	0.000548	0.000173	0.00555%
**L1767**	**False**	**OD1**	0.000289	0.0263%	0.0	1	0.0754	0.0103	0.00573	0.000116	0.00371%
**L39**	**False**	**OD1**	0.00028	0.0255%	0.0	1	0.0143	0.000514	0.00232	0.000172	0.00551%
**OTU_185**	**True**	**PRR-12**	0.00027	0.0246%	0.0	1	0.0141	0.00099	0.000555	0.000456	0.0146%
**L940**	**False**	**koll11**	0.000265	0.0242%	0.0	1	0.221	0.0247	0.0369	5.4e-05	0.00173%
**L1427**	**False**	**ZB2**	0.000264	0.0241%	0.0	1	0.0127	0.0812	0.112	0.000309	0.0099%
**L968**	**False**	**koll11**	0.000258	0.0235%	0.0	1	0.18	0.0214	0.0304	5.65e-05	0.00181%
**L950**	**False**	**koll11**	0.000256	0.0233%	0.0	1	0.21	0.0237	0.0354	5.78e-05	0.00185%
**L1317**	**False**	**OD1**	0.000253	0.023%	0.0	1	0.00904	0.000614	0.0005	0.00017	0.00544%
**L931**	**False**	**koll11**	0.000247	0.0225%	0.0	1	0.224	0.0248	0.037	5.04e-05	0.00161%
**L1410**	**False**	**ZB2**	0.00024	0.0219%	0.0	1	0.00467	0.298	0.282	0.0003	0.00961%
**L1782**	**False**	**OD1**	0.000229	0.0209%	0.0	1	0.0335	0.00442	0.00306	0.000139	0.00446%
**OTU_81**	**True**	**OD1**	0.000228	0.0208%	0.0	1	0.00702	0.00205	0.000547	0.000403	0.0129%
**L1741**	**False**	**OD1**	0.000223	0.0203%	0.0	1	0.0944	0.0131	0.00826	7.93e-05	0.00254%
**OTU_4624**	**True**	**PRR-11**	0.000218	0.0199%	0.0	1	0.00377	8.73e-06	0.000276	0.000526	0.0169%
**L52**	**False**	**ABY1**	0.000202	0.0184%	0.0	1	0.00736	0.0506	0.0222	0.000512	0.0164%
**L987**	**False**	**koll11**	0.000199	0.0181%	0.0	1	0.153	0.0176	0.0256	4.18e-05	0.00134%
**L457**	**False**	**OP11**	0.000194	0.0177%	0.0	1	0.0375	0.00518	0.00417	8.77e-05	0.00281%
**L1408**	**False**	**ZB2**	0.000194	0.0176%	0.0	1	0.00468	0.299	0.283	0.000241	0.00772%
**L423**	**False**	**Bacteria**	0.000192	0.0175%	0.0	1	0.0417	0.00603	0.00747	0.000116	0.00371%
**L1809**	**False**	**OD1**	0.000191	0.0174%	0.0	1	0.00972	0.0011	0.000192	0.000156	0.00501%
**L1428**	**False**	**ZB2**	0.000184	0.0168%	0.0	1	0.0111	0.0521	0.0801	0.000209	0.00671%
**L1429**	**False**	**ZB2**	0.000181	0.0165%	0.0	1	0.0062	0.0509	0.079	0.000159	0.00508%
**L645**	**False**	**koll11**	0.00018	0.0164%	0.0	1	0.0186	0.00244	0.0049	0.0001	0.00321%
**L744**	**False**	**OP3**	0.000174	0.0158%	0.0	1	0.0641	0.00611	0.0129	8.39e-05	0.00269%
**L90**	**False**	**ABY1**	0.000172	0.0156%	0.0	1	0.0241	0.00513	0.00116	9.31e-05	0.00298%
**L583**	**False**	**PRR-12**	0.000159	0.0145%	0.0	1	0.014	0.00144	0.000858	0.000142	0.00454%
**L1797**	**False**	**OD1**	0.000157	0.0143%	0.0	1	0.0288	0.0036	0.00255	0.000105	0.00335%
**L1742**	**False**	**OD1**	0.000155	0.0141%	0.0	1	0.019	0.00281	0.00252	0.000115	0.00367%
**L969**	**False**	**koll11**	0.000154	0.014%	0.0	1	0.177	0.0208	0.0282	3.15e-05	0.00101%
**L977**	**False**	**koll11**	0.000146	0.0133%	0.0	1	0.163	0.0189	0.0262	3.03e-05	0.000971%
**L467**	**False**	**OP11-4**	0.000143	0.013%	0.0	1	0.0297	0.00439	0.00327	9.88e-05	0.00317%
**L1829**	**False**	**OD1**	0.000142	0.0129%	0.0	1	0.013	0.00207	0.00192	0.00014	0.0045%
**L711**	**False**	**PBS-25**	0.00014	0.0128%	0.0	1	0.0507	0.00565	0.00807	9.97e-05	0.00319%
**L441**	**False**	**Bacteria**	0.000139	0.0127%	0.0	1	0.039	0.00541	0.00539	6.98e-05	0.00223%
**L1539**	**False**	**ZB2**	0.000137	0.0125%	0.0	1	0.0268	0.00367	0.00277	0.000108	0.00345%
**L934**	**False**	**koll11**	0.000137	0.0125%	0.0	1	0.222	0.0247	0.037	2.79e-05	0.000893%
**L1415**	**False**	**ZB2**	0.000137	0.0124%	0.0	1	0.000574	0.031	0.0699	0.000263	0.00844%
**L1452**	**False**	**ZB2**	0.000136	0.0124%	0.0	1	0.000524	0.024	0.0397	0.000135	0.00434%
**L1446**	**False**	**ZB2**	0.000136	0.0124%	0.0	1	0.000112	0.0207	0.0288	0.000103	0.00329%
**L847**	**False**	**koll11**	0.000135	0.0123%	0.0	1	0.0315	0.0034	0.00361	8.81e-05	0.00282%
**L837**	**False**	**OP3**	0.000134	0.0122%	0.0	1	0.0921	0.00928	0.0132	3.91e-05	0.00125%
**OTU_8000**	**True**	**OD1**	0.000132	0.012%	0.0	1	0.00865	0.000614	0.000483	9.11e-05	0.00292%
**L578**	**False**	**PRR-12**	0.000129	0.0117%	0.0	1	0.0334	0.00278	0.00165	8e-05	0.00256%
**L709**	**False**	**PBS-25**	0.000129	0.0117%	0.0	1	0.0507	0.00565	0.00829	9.31e-05	0.00298%
**L171**	**False**	**OD1**	0.000126	0.0114%	0.0	1	0.0338	0.00679	0.00442	7.42e-05	0.00238%
**L1497**	**False**	**ZB2**	0.000125	0.0114%	0.0	1	0.00159	0.0291	0.0323	0.000379	0.0122%
**L1783**	**False**	**OD1**	0.000124	0.0113%	0.0	1	0.0332	0.00442	0.00298	7.5e-05	0.0024%
**L1013**	**False**	**koll11**	0.000123	0.0112%	0.0	1	0.0223	0.00245	0.00313	5.35e-05	0.00171%
**L495**	**False**	**OP11-4**	0.000123	0.0112%	0.0	1	0.0155	0.00222	0.00216	0.000222	0.0071%
**L1166**	**False**	**koll11**	0.000122	0.0111%	0.0	1	0.0129	0.00142	0.00194	8.39e-05	0.00269%

**L581**	**False**	**PRR-12**	0.00012	0.011%	0.0	1	0.0148	0.00144	0.000858	9.84e-05	0.00315%
**OTU_7453**	**True**	**ABY1**	0.000118	0.0107%	0.0	1	0.00408	0.000353	0.000265	0.000135	0.00433%
**L1841**	**False**	**OD1**	0.000117	0.0107%	0.0	1	0.00936	0.0013	0.000828	9.15e-05	0.00293%
**L1307**	**False**	**OD1**	0.000117	0.0106%	0.0	1	0.0064	0.000399	0.000845	0.000147	0.00472%
**L785**	**False**	**OP3**	0.000116	0.0105%	0.0	1	0.0503	0.00501	0.0112	8.4e-05	0.00269%
**L31**	**False**	**OD1**	0.000115	0.0105%	0.0	1	0.0158	0.000543	0.00234	7.19e-05	0.0023%
**L51**	**False**	**OD1**	0.000111	0.0101%	0.0	1	0.0491	0.109	0.0581	0.000539	0.0173%
**L1743**	**False**	**OD1**	0.00011	0.0101%	0.0	1	0.0174	0.0019	0.000175	3.98e-05	0.00128%
**L61**	**False**	**ABY1**	0.00011	0.01%	0.0	1	0.00124	0.0496	0.0222	0.000145	0.00463%
**L459**	**False**	**OP11-4**	0.00011	0.01%	0.0	1	0.0305	0.00451	0.00338	6.87e-05	0.0022%
**L1807**	**False**	**OD1**	0.000106	0.00969%	0.0	1	0.0128	0.00126	0.000386	7.04e-05	0.00225%
**L1010**	**False**	**koll11**	0.000105	0.00953%	0.0	1	0.0824	0.00944	0.0155	2.1e-05	0.000673%
**OTU_6636**	**True**	**ABY1**	0.000103	0.00939%	0.0	1	0.00696	0.00231	0.000915	0.00031	0.00994%
**L713**	**False**	**PBS-25**	0.000101	0.00916%	0.0	1	0.0502	0.00565	0.00804	7.27e-05	0.00233%
**L1449**	**False**	**ZB2**	0.0001	0.00911%	0.0	1	0.00142	0.0292	0.0489	8.42e-05	0.0027%
**L390**	**False**	**ABY1**	9.85e-05	0.00898%	0.0	1	0.0052	0.000224	0.000332	5.62e-05	0.0018%
**L594**	**False**	**PRR-12**	9.84e-05	0.00896%	0.0	1	0.0186	0.0013	0.000671	0.000101	0.00323%
**L790**	**False**	**OP3**	9.78e-05	0.0089%	0.0	1	0.0493	0.0049	0.0108	7.13e-05	0.00228%
**L1450**	**False**	**ZB2**	9.72e-05	0.00886%	0.0	1	0.000562	0.0243	0.0397	9.7e-05	0.00311%
**L988**	**False**	**koll11**	9.71e-05	0.00885%	0.0	1	0.143	0.0158	0.0248	1.79e-05	0.000574%
**L1316**	**False**	**OD1**	9.7e-05	0.00884%	0.0	1	0.0107	0.000653	0.000507	4.53e-05	0.00145%
**L932**	**False**	**koll11**	9.7e-05	0.00883%	0.0	1	0.222	0.0247	0.037	1.97e-05	0.000632%
**L1656**	**False**	**OD1**	9.67e-05	0.00881%	0.0	1	0.00788	0.00229	0.000805	0.000168	0.00538%
**L232**	**False**	**ABY1**	9.67e-05	0.00881%	0.0	1	0.0133	0.00172	0.00155	8.66e-05	0.00277%
**L1777**	**False**	**OD1**	9.58e-05	0.00873%	0.0	1	0.0431	0.00575	0.00358	3.91e-05	0.00125%
**L1443**	**False**	**ZB2**	9.51e-05	0.00866%	0.0	1	0.00328	0.0505	0.0787	6.79e-05	0.00217%
**L1781**	**False**	**OD1**	9.5e-05	0.00865%	0.0	1	0.0421	0.00574	0.00358	4.16e-05	0.00133%
**OTU_7957**	**True**	**OD1**	9.48e-05	0.00864%	0.0	1	0.00255	0.000179	1.82e-05	0.000105	0.00337%
**OTU_5981**	**True**	**ZB2**	9.3e-05	0.00847%	0.0	1	0.000505	0.0248	0.0649	0.000189	0.00607%
**OTU_7115**	**True**	**ABY1**	9.28e-05	0.00845%	0.0	1	0.000112	0.015	0.0133	0.000167	0.00536%
**L1028**	**False**	**koll11**	9.27e-05	0.00844%	0.0	1	0.029	0.00339	0.00527	3.23e-05	0.00104%
**L1009**	**False**	**koll11**	8.98e-05	0.00818%	0.0	1	0.0848	0.00956	0.016	1.82e-05	0.000583%
**L1246**	**False**	**TM7-1**	8.87e-05	0.00808%	0.0	1	2.86e-05	0.0112	0.0087	0.000169	0.0054%
**OTU_6581**	**True**	**ZB2**	8.87e-05	0.00808%	0.0	1	0.000284	0.024	0.0397	8.39e-05	0.00269%
**L410**	**False**	**ABY1**	8.77e-05	0.00799%	0.0	1	0.000125	0.0059	0.00827	0.000132	0.00424%
**L72**	**False**	**ABY1**	7.68e-05	0.007%	0.0	1	0.000622	0.0389	0.0113	0.000131	0.00419%
**L360**	**False**	**ABY1**	7.66e-05	0.00697%	0.0	1	0.00616	0.000751	0.00027	0.0001	0.00321%
**L528**	**False**	**TM6**	7.6e-05	0.00692%	0.0	1	0.00747	0.000667	0.00325	0.000249	0.00798%
**L123**	**False**	**ABY1**	7.58e-05	0.00691%	0.0	1	4.16e-05	0.0108	0.00791	0.000117	0.00374%
**L843**	**False**	**koll11**	7.58e-05	0.0069%	0.0	1	0.0409	0.00391	0.00539	4.61e-05	0.00148%
**L1808**	**False**	**OD1**	7.48e-05	0.00681%	0.0	1	0.0104	0.00112	0.000203	5.48e-05	0.00176%
**L54**	**False**	**ABY1**	7.3e-05	0.00665%	0.0	1	0.00736	0.0506	0.0222	0.000185	0.00593%
**L68**	**False**	**ABY1**	7.23e-05	0.00659%	0.0	1	0.000752	0.0472	0.0183	9.94e-05	0.00318%
**L989**	**False**	**koll11**	7.18e-05	0.00654%	0.0	1	0.143	0.0158	0.0248	1.35e-05	0.000434%
**OTU_5941**	**True**	**OD1**	7.13e-05	0.00649%	0.0	1	0.00135	0.00031	8.04e-05	0.000202	0.00646%
**L148**	**False**	**OD1**	7.04e-05	0.00642%	0.0	1	0.00355	0.00061	0.000174	9.79e-05	0.00314%
**OTU_314**	**True**	**koll11**	6.93e-05	0.00631%	0.0	1	0.0104	0.000474	0.000705	4.72e-05	0.00151%
**OTU_2703**	**True**	**koll11**	6.9e-05	0.00628%	0.0	1	0.0141	0.00172	0.000941	6.91e-05	0.00221%
**L1853**	**False**	**OD1**	6.86e-05	0.00625%	0.0	1	0.00858	0.00132	0.000514	2.93e-05	0.000937%
**OTU_8447**	**True**	**ABY1**	6.72e-05	0.00612%	0.0	1	0.000726	0.00753	0.0137	0.00016	0.00513%
**L1655**	**False**	**OD1**	6.72e-05	0.00612%	0.0	1	0.0188	0.00413	0.00234	4.84e-05	0.00155%
**L942**	**False**	**koll11**	6.72e-05	0.00612%	0.0	1	0.221	0.0247	0.0368	1.37e-05	0.000438%
**L792**	**False**	**OP3**	6.61e-05	0.00602%	0.0	1	0.0478	0.00474	0.0107	4.87e-05	0.00156%
**OTU_6197**	**True**	**OD1**	6.59e-05	0.006%	0.0	1	0.00398	0.00022	0.00058	0.000113	0.00361%
**L766**	**False**	**OP3**	6.58e-05	0.006%	0.0	1	0.0548	0.00533	0.0117	4.12e-05	0.00132%
**L1011**	**False**	**koll11**	6.55e-05	0.00597%	0.0	1	0.0703	0.0079	0.0116	1.39e-05	0.000445%
**L1167**	**False**	**koll11**	6.52e-05	0.00594%	0.0	1	0.0124	0.00122	0.00139	3.99e-05	0.00128%
**OTU_8254**	**True**	**OD1**	6.51e-05	0.00593%	0.0	1	0.000906	4.94e-05	1.93e-05	9.51e-05	0.00305%
**L733**	**False**	**PBS-25**	6.48e-05	0.0059%	0.0	1	0.0234	0.00227	0.00269	6.58e-05	0.00211%
**L389**	**False**	**ABY1**	6.42e-05	0.00585%	0.0	1	0.0133	0.00266	0.00139	7.37e-05	0.00236%
**OTU_10802**	**True**	**OP11-4**	6.28e-05	0.00572%	0.0	1	0.00197	0.000126	1.69e-05	6.96e-05	0.00223%
**L271**	**False**	**OD1**	6.23e-05	0.00567%	0.0	1	0.00549	0.00299	0.000973	0.000223	0.00714%
**OTU_7960**	**True**	**OD1**	6.18e-05	0.00563%	0.0	1	0.00101	1.33e-05	2.05e-05	9.61e-05	0.00308%
**L260**	**False**	**OD1**	6.17e-05	0.00562%	0.0	1	0.0148	0.0102	0.00477	0.000319	0.0102%
**L81**	**False**	**ABY1**	6.16e-05	0.00561%	0.0	1	0.0261	0.00817	0.00278	4.36e-05	0.0014%

**OTU_8880**	**True**	**ABY1**	6.15e-05	0.0056%	0.0	1	0.00277	0.000153	1.24e-06	8.16e-05	0.00261%
**L1309**	**False**	**OD1**	6.14e-05	0.00559%	0.0	1	0.00599	0.000399	0.000842	8.38e-05	0.00268%
**OTU_7795**	**True**	**ABY1**	6.07e-05	0.00553%	0.0	1	1.33e-05	0.00531	0.00631	9.97e-05	0.00319%
**L512**	**False**	**OP11-3**	6.04e-05	0.0055%	0.0	1	0.00202	8.71e-05	7.23e-05	0.000131	0.00418%
**L28**	**False**	**OD1**	6.03e-05	0.00549%	0.0	1	0.154	0.155	0.0975	0.000684	0.0219%
**OTU_8200**	**True**	**ABY1**	6e-05	0.00546%	0.0	1	0.00189	6.43e-05	6.92e-06	7.08e-05	0.00227%
**OTU_6128**	**True**	**ZB2**	5.95e-05	0.00542%	0.0	1	0.00496	0.0006	0.000297	9.09e-05	0.00291%
**L481**	**False**	**OP11-4**	5.93e-05	0.0054%	0.0	1	0.0261	0.00383	0.0031	5.3e-05	0.0017%
**L951**	**False**	**koll11**	5.89e-05	0.00537%	0.0	1	0.0304	0.00225	0.00504	3.7e-05	0.00119%
**L184**	**False**	**SM2F11**	5.77e-05	0.00526%	0.0	1	0.00897	0.00166	0.000263	3.74e-05	0.0012%
**OTU_260**	**True**	**koll11**	5.61e-05	0.00511%	0.0	1	0.02	0.00139	0.00111	3.83e-05	0.00123%
**L63**	**False**	**ABY1**	5.61e-05	0.00511%	0.0	1	0.00124	0.0493	0.022	7.35e-05	0.00235%
**L789**	**False**	**OP3**	5.57e-05	0.00507%	0.0	1	0.0495	0.0049	0.0108	4.02e-05	0.00129%
**L47**	**False**	**OD1**	5.55e-05	0.00506%	0.0	1	0.0496	0.11	0.0581	0.00027	0.00866%
**L480**	**False**	**OP11-4**	5.55e-05	0.00506%	0.0	1	0.0267	0.00388	0.00315	4.66e-05	0.00149%
**OTU_7384**	**True**	**ABY1**	5.52e-05	0.00503%	0.0	1	4.16e-05	0.0104	0.0076	9.18e-05	0.00294%
**L1842**	**False**	**OD1**	5.49e-05	0.005%	0.0	1	0.00328	0.000538	0.000632	6.4e-05	0.00205%
**OTU_6068**	**True**	**OD1**	5.48e-05	0.00499%	0.0	1	0.00166	3.93e-05	7.45e-06	7.57e-05	0.00242%
**L623**	**False**	**PBS-25**	5.46e-05	0.00498%	0.0	1	0.0115	0.00166	0.000552	2.48e-05	0.000796%
**L767**	**False**	**OP3**	5.46e-05	0.00497%	0.0	1	0.0546	0.00533	0.0117	3.43e-05	0.0011%
**L1594**	**False**	**OD1**	5.32e-05	0.00484%	0.0	1	0.000249	0.00642	0.00413	0.000125	0.00401%
**OTU_6646**	**True**	**ZB2**	5.31e-05	0.00483%	0.0	1	2.51e-05	0.00534	0.00602	9.11e-05	0.00292%
**L366**	**False**	**ABY1**	5.3e-05	0.00482%	0.0	1	0.00569	0.00046	0.000102	3.63e-05	0.00116%
**L320**	**False**	**OD1**	5.27e-05	0.0048%	0.0	1	0.0128	0.00125	0.00107	3.42e-05	0.0011%
**OTU_6705**	**True**	**ZB2**	5.25e-05	0.00478%	0.0	1	1.36e-05	0.00179	0.00585	4.52e-05	0.00145%
**L1543**	**False**	**ZB2**	5.22e-05	0.00475%	0.0	1	0.0264	0.00367	0.00277	4.24e-05	0.00136%
**L1402**	**False**	**ZB2**	5.21e-05	0.00474%	0.0	1	0.00297	0.0635	0.0306	0.000113	0.00363%
**L1412**	**False**	**ZB2**	5.2e-05	0.00474%	0.0	1	0.00111	0.031	0.071	0.000101	0.00323%
**OTU_7221**	**True**	**ABY1**	5.18e-05	0.00472%	0.0	1	0.00598	0.000237	9.9e-05	3.67e-05	0.00118%
**L1855**	**False**	**OD1**	5.09e-05	0.00464%	0.0	1	0.00794	0.00129	0.000514	2.6e-05	0.000832%
**L1426**	**False**	**OD1**	5.08e-05	0.00463%	0.0	1	0.0263	0.0839	0.12	9.2e-05	0.00295%
**L579**	**False**	**PRR-12**	5.02e-05	0.00457%	0.0	1	0.0148	0.00148	0.000984	4.2e-05	0.00135%
**L511**	**False**	**OP11**	5e-05	0.00456%	0.0	1	0.00298	0.000143	8.33e-05	7.35e-05	0.00236%
**L89**	**False**	**ABY1**	4.98e-05	0.00454%	0.0	1	0.0241	0.00802	0.00276	3.86e-05	0.00124%
**L1500**	**False**	**ZB2**	4.91e-05	0.00448%	0.0	1	0.000318	0.0141	0.00774	0.00025	0.00799%
**L1005**	**False**	**koll11**	4.91e-05	0.00447%	0.0	1	0.09	0.00992	0.0165	9.16e-06	0.000293%
**L1444**	**False**	**ZB2**	4.86e-05	0.00443%	0.0	1	0.00186	0.0213	0.0297	5.59e-05	0.00179%
**L46**	**False**	**OD1**	4.77e-05	0.00435%	0.0	1	0.0523	0.11	0.0582	0.000245	0.00785%
**L952**	**False**	**koll11**	4.75e-05	0.00433%	0.0	1	0.0298	0.00192	0.00494	3.09e-05	0.000991%
**L760**	**False**	**OP3**	4.75e-05	0.00432%	0.0	1	0.0573	0.00544	0.0118	3.03e-05	0.000969%
**L1459**	**False**	**ZB2**	4.6e-05	0.00419%	0.0	1	0.0	0.000294	0.00226	0.000104	0.00332%
**L809**	**False**	**OP3**	4.6e-05	0.00419%	0.0	1	0.0353	0.00348	0.00705	3.78e-05	0.00121%
**L12**	**False**	**OD1**	4.58e-05	0.00417%	0.0	1	0.00181	0.000129	6.93e-06	5.87e-05	0.00188%
**L605**	**False**	**PRR-12**	4.55e-05	0.00414%	0.0	1	0.002	3.67e-06	0.0	5.23e-05	0.00168%
**L93**	**False**	**ABY1**	4.54e-05	0.00414%	0.0	1	0.0222	0.00478	0.00115	2.42e-05	0.000776%
**L1791**	**False**	**OD1**	4.49e-05	0.00409%	0.0	1	0.00296	0.000564	0.00035	4.81e-05	0.00154%
**L1661**	**False**	**OD1**	4.48e-05	0.00408%	0.0	1	0.011	0.00184	0.00153	4.61e-05	0.00148%
**OTU_5604**	**True**	**OD1**	4.42e-05	0.00403%	0.0	1	0.0151	0.00162	0.000144	1.76e-05	0.000563%
**L800**	**False**	**OP3**	4.39e-05	0.004%	0.0	1	0.0463	0.0046	0.0106	3.34e-05	0.00107%
**L1092**	**False**	**koll11**	4.38e-05	0.00399%	0.0	1	0.0104	0.00104	0.00237	4.96e-05	0.00159%
**OTU_6570**	**True**	**SM2F11**	4.34e-05	0.00395%	0.0	1	0.00188	2.64e-05	1.33e-06	4.19e-05	0.00134%
**L1567**	**False**	**ZB2**	4.32e-05	0.00394%	0.0	1	0.00312	0.000439	0.000749	5.84e-05	0.00187%
**L233**	**False**	**ABY1**	4.3e-05	0.00392%	0.0	1	0.00568	0.000301	0.000313	5.21e-05	0.00167%
**L359**	**False**	**OD1**	4.3e-05	0.00391%	0.0	1	0.00717	0.000772	0.000271	4.66e-05	0.00149%
**L488**	**False**	**OP11-4**	4.27e-05	0.00389%	0.0	1	0.0084	0.0014	0.000917	3.94e-05	0.00126%
**L300**	**False**	**ABY1**	4.27e-05	0.00389%	0.0	1	0.000808	0.0112	0.00746	9.1e-05	0.00292%
**L597**	**False**	**PRR-12**	4.26e-05	0.00388%	0.0	1	0.00427	0.000308	0.000116	3.82e-05	0.00123%
**L1885**	**False**	**OD1**	4.25e-05	0.00387%	0.0	1	0.00434	0.000686	0.000229	3.29e-05	0.00105%
**OTU_10771**	**True**	**OP11-3**	4.24e-05	0.00386%	0.0	1	0.00158	0.0	0.0	9.3e-05	0.00298%
**L648**	**False**	**koll11**	4.23e-05	0.00386%	0.0	1	0.017	0.00244	0.00477	2.69e-05	0.000861%
**L984**	**False**	**koll11**	4.22e-05	0.00384%	0.0	1	0.00636	0.0007	0.000206	6.12e-05	0.00196%
**OTU_172**	**True**	**PBS-25**	4.19e-05	0.00382%	0.0	1	0.0146	0.00203	0.00355	6.85e-05	0.00219%
**L1596**	**False**	**OD1**	4.16e-05	0.00379%	0.0	1	0.000249	0.00641	0.00347	8.76e-05	0.00281%
**L278**	**False**	**ABY1**	4.15e-05	0.00378%	0.0	1	0.00357	0.000513	9.59e-05	4.89e-05	0.00157%

**L1831**	**False**	**OD1**	4.12e-05	0.00375%	0.0	1	0.0117	0.00176	0.00184	3.95e-05	0.00126%
**OTU_4910**	**True**	**OD1**	4.07e-05	0.00371%	0.0	1	0.000581	4.26e-05	0.000136	0.000144	0.00461%
**L140**	**False**	**ABY1**	4.07e-05	0.00371%	0.0	1	0.0049	0.0203	0.00712	0.000258	0.00825%
**L1112**	**False**	**koll11**	4.07e-05	0.00371%	0.0	1	0.0276	0.00347	0.00297	1.86e-05	0.000596%
**L585**	**False**	**PRR-12**	4.03e-05	0.00367%	0.0	1	0.0138	0.00144	0.000827	3.72e-05	0.00119%
**L64**	**False**	**ABY1**	3.97e-05	0.00361%	0.0	1	0.00123	0.0472	0.0184	5.84e-05	0.00187%
**OTU_7053**	**True**	**ABY1**	3.95e-05	0.0036%	0.0	1	0.00566	0.000751	0.000239	6.22e-05	0.00199%
**L1546**	**False**	**ZB2**	3.94e-05	0.00359%	0.0	1	0.0242	0.00344	0.00226	3.31e-05	0.00106%
**L624**	**False**	**PBS-25**	3.94e-05	0.00358%	0.0	1	0.00711	0.00151	0.000451	3.12e-05	0.001%
**OTU_7302**	**True**	**ABY1**	3.93e-05	0.00358%	0.0	1	0.00487	0.00125	0.00013	7.35e-05	0.00236%
**L1677**	**False**	**OD1**	3.91e-05	0.00356%	0.0	1	0.00388	0.000373	0.000367	6.68e-05	0.00214%
**L1827**	**False**	**OD1**	3.89e-05	0.00355%	0.0	1	0.0131	0.00207	0.00192	3.81e-05	0.00122%
**L794**	**False**	**OP3**	3.87e-05	0.00352%	0.0	1	0.047	0.00464	0.0106	2.86e-05	0.000915%
**L192**	**False**	**ABY1**	3.86e-05	0.00352%	0.0	1	0.0207	0.00479	0.00385	4.32e-05	0.00138%
**OTU_6344**	**True**	**OD1**	3.85e-05	0.00351%	0.0	1	0.00274	0.000474	6.59e-05	4.66e-05	0.00149%
**L628**	**False**	**PBS-25**	3.84e-05	0.0035%	0.0	1	0.00341	0.000157	0.000101	5.55e-05	0.00178%
**OTU_7971**	**True**	**OD1**	3.83e-05	0.00349%	0.0	1	0.00105	0.000137	4.09e-05	7.55e-05	0.00242%
**L807**	**False**	**OP3**	3.82e-05	0.00348%	0.0	1	0.0435	0.00379	0.00904	2.97e-05	0.000951%
**L1230**	**False**	**[Acetothermia]**	3.8e-05	0.00347%	0.0	1	0.000813	3.22e-05	0.0	8.29e-05	0.00266%
**L1689**	**False**	**OD1**	3.8e-05	0.00346%	0.0	1	0.00947	0.00118	0.000352	1.76e-05	0.000563%
**L1843**	**False**	**OD1**	3.72e-05	0.00339%	0.0	1	0.00315	0.000535	0.000629	4.58e-05	0.00147%
**L1027**	**False**	**koll11**	3.7e-05	0.00337%	0.0	1	0.0407	0.00445	0.00763	1.21e-05	0.000389%
**OTU_186**	**True**	**koll11**	3.67e-05	0.00334%	0.0	1	0.0236	0.00329	0.00244	2.01e-05	0.000645%
**L1806**	**False**	**OD1**	3.64e-05	0.00331%	0.0	1	0.0142	0.00151	0.000588	2.68e-05	0.00086%
**L321**	**False**	**OD1**	3.62e-05	0.0033%	0.0	1	0.0122	0.00122	0.00107	2.42e-05	0.000774%
**L97**	**False**	**ABY1**	3.61e-05	0.00329%	0.0	1	0.0133	0.00241	0.00043	2.52e-05	0.000809%
**L1177**	**False**	**koll11**	3.6e-05	0.00328%	0.0	1	0.0101	0.000799	0.00129	2.25e-05	0.000722%
**OTU_242**	**True**	**PBS-25**	3.59e-05	0.00327%	0.0	1	0.0147	0.00127	0.00106	5.14e-05	0.00165%
**OTU_8054**	**True**	**ABY1**	3.58e-05	0.00326%	0.0	1	0.0015	5.64e-05	2.4e-05	6.29e-05	0.00201%
**OTU_281**	**True**	**koll11**	3.56e-05	0.00324%	0.0	1	0.00378	0.000365	0.000508	5.72e-05	0.00183%
**OTU_7992**	**True**	**TM7-1**	3.46e-05	0.00315%	0.0	1	2.86e-05	0.0112	0.0087	6.57e-05	0.00211%
**OTU_183**	**True**	**koll11**	3.45e-05	0.00314%	0.0	1	0.00884	0.00165	0.000718	6.61e-05	0.00212%
**OTU_6978**	**True**	**ZB2**	3.44e-05	0.00314%	0.0	1	0.00253	0.000261	2.26e-05	4.09e-05	0.00131%
**OTU_83**	**True**	**Mb-NB09**	3.4e-05	0.00309%	0.0	1	0.000655	0.000133	4.67e-05	7.96e-05	0.00255%
**L1716**	**False**	**Mb-NB09**	3.38e-05	0.00308%	0.0	1	0.00271	0.000279	0.000114	6.01e-05	0.00192%
**L261**	**False**	**OD1**	3.36e-05	0.00307%	0.0	1	0.0145	0.008	0.00345	0.000117	0.00375%
**L1335**	**False**	**ZB2**	3.32e-05	0.00302%	0.0	1	0.00371	0.000763	0.00034	4.05e-05	0.0013%
**OTU_7002**	**True**	**ZB2**	3.3e-05	0.00301%	0.0	1	0.000898	0.0	0.0	6.83e-05	0.00219%
**L333**	**False**	**OD1**	3.3e-05	0.003%	0.0	1	0.00169	0.000178	6.94e-05	4.7e-05	0.0015%
**L58**	**False**	**ABY1**	3.29e-05	0.003%	0.0	1	0.00395	0.0505	0.0222	5.77e-05	0.00185%
**L1110**	**False**	**koll11**	3.28e-05	0.00299%	0.0	1	0.0382	0.00418	0.00461	1.08e-05	0.000347%
**L803**	**False**	**OP3**	3.28e-05	0.00299%	0.0	1	0.0435	0.00379	0.0094	2.64e-05	0.000844%
**OTU_303**	**True**	**koll11**	3.25e-05	0.00296%	0.0	1	0.00975	0.00092	0.00203	3.93e-05	0.00126%
**L1697**	**False**	**Mb-NB09**	3.23e-05	0.00295%	0.0	1	0.00103	0.000183	5.56e-05	5.07e-05	0.00163%
**L1561**	**False**	**ZB2**	3.22e-05	0.00294%	0.0	1	0.0057	0.000665	0.000297	4.85e-05	0.00155%
**L845**	**False**	**koll11**	3.2e-05	0.00292%	0.0	1	0.0352	0.0037	0.00461	2.05e-05	0.000656%
**OTU_5994**	**True**	**ZB2**	3.17e-05	0.00289%	0.0	1	0.000434	0.0468	0.0235	6.74e-05	0.00216%
**L660**	**False**	**koll11**	3.16e-05	0.00288%	0.0	1	0.00373	0.000239	0.000608	4.24e-05	0.00136%
**L1536**	**False**	**OD1**	3.13e-05	0.00285%	0.0	1	0.00458	0.000321	0.000696	4.52e-05	0.00145%
**L186**	**False**	**SM2F11**	3.13e-05	0.00285%	0.0	1	0.00602	0.00116	0.000232	3.88e-05	0.00124%
**L45**	**False**	**OD1**	3.08e-05	0.0028%	0.0	1	0.113	0.151	0.0935	0.000436	0.014%
**OTU_6780**	**True**	**ZB2**	3.08e-05	0.0028%	0.0	1	0.0049	0.000601	0.00401	0.000104	0.00334%
**L1544**	**False**	**ZB2**	3.07e-05	0.0028%	0.0	1	0.0247	0.00345	0.00227	2.48e-05	0.000795%
**OTU_5705**	**True**	**OD1**	3.07e-05	0.0028%	0.0	1	0.00949	0.000329	0.00123	2.96e-05	0.000948%
**L391**	**False**	**ABY1**	3.04e-05	0.00277%	0.0	1	0.00404	0.000194	0.000173	2.64e-05	0.000845%
**L842**	**False**	**OP3**	3.04e-05	0.00277%	0.0	1	0.0618	0.00593	0.0078	1.41e-05	0.000451%
**OTU_689**	**True**	**koll11**	3.02e-05	0.00276%	0.0	1	0.00412	0.00044	0.00104	3.17e-05	0.00102%
**L811**	**False**	**OP3**	3.01e-05	0.00274%	0.0	1	0.0352	0.00345	0.00705	2.48e-05	0.000793%
**L1413**	**False**	**ZB2**	2.99e-05	0.00273%	0.0	1	0.000574	0.031	0.0701	5.75e-05	0.00184%
**L1712**	**False**	**Mb-NB09**	2.99e-05	0.00272%	0.0	1	0.00388	0.000522	0.000178	4.31e-05	0.00138%
**L646**	**False**	**koll11**	2.98e-05	0.00272%	0.0	1	0.017	0.00244	0.0049	1.9e-05	0.000609%
**OTU_62**	**True**	**ZB2**	2.96e-05	0.0027%	0.0	1	0.000105	0.014	0.0077	0.000136	0.00436%
**L32**	**False**	**ABY1**	2.96e-05	0.0027%	0.0	1	0.00151	2.88e-05	2.26e-05	3.95e-05	0.00127%
**OTU_180**	**True**	**koll11**	2.96e-05	0.00269%	0.0	1	0.00631	0.000815	0.000432	4.37e-05	0.0014%
**OTU_10861**	**True**	**OP11-4**	2.95e-05	0.00268%	0.0	1	0.00378	0.000517	0.000706	4.34e-05	0.00139%

**L972**	**False**	**koll11**	2.9e-05	0.00265%	0.0	1	0.174	0.0201	0.028	6.08e-06	0.000195%
**L1004**	**False**	**koll11**	2.88e-05	0.00263%	0.0	1	0.128	0.0141	0.0211	4.87e-06	0.000156%
**L59**	**False**	**ABY1**	2.87e-05	0.00261%	0.0	1	0.00169	0.0496	0.0222	3.97e-05	0.00127%
**L347**	**False**	**ABY1**	2.81e-05	0.00256%	0.0	1	0.00421	0.0004	0.000282	3.12e-05	0.000998%
**L613**	**False**	**BD4-9**	2.8e-05	0.00255%	0.0	1	0.00075	1.39e-05	0.0	5.01e-05	0.0016%
**L92**	**False**	**ABY1**	2.78e-05	0.00253%	0.0	1	0.023	0.00489	0.00116	1.5e-05	0.00048%
**OTU_2037**	**True**	**koll11**	2.77e-05	0.00253%	0.0	1	0.0104	0.00114	0.00116	3.29e-05	0.00106%
**L874**	**False**	**koll11**	2.75e-05	0.00251%	0.0	1	0.0183	0.00211	0.00369	1.57e-05	0.000502%
**L1550**	**False**	**ZB2**	2.75e-05	0.0025%	0.0	1	0.0213	0.00308	0.00213	2.61e-05	0.000836%
**L523**	**False**	**OP11**	2.73e-05	0.00248%	0.0	1	0.000965	5.63e-05	1.1e-05	4.83e-05	0.00155%
**OTU_85**	**True**	**OD1**	2.7e-05	0.00246%	0.0	1	0.0003	3.8e-05	0.0	7.99e-05	0.00256%
**OTU_7179**	**True**	**OD1**	2.7e-05	0.00246%	0.0	1	0.000436	0.0	0.0	5.14e-05	0.00165%
**OTU_3932**	**True**	**SJA-4**	2.69e-05	0.00245%	0.0	1	0.00169	0.0	0.0	6.31e-05	0.00202%
**L1593**	**False**	**OD1**	2.69e-05	0.00245%	0.0	1	0.000249	0.00655	0.00413	5.86e-05	0.00188%
**OTU_9009**	**True**	**Mb-NB09**	2.68e-05	0.00245%	0.0	1	0.000989	7.16e-06	2.68e-05	6.86e-05	0.0022%
**L730**	**False**	**PBS-25**	2.68e-05	0.00245%	0.0	1	0.00829	0.000654	0.000908	3.9e-05	0.00125%
**OTU_6723**	**True**	**OD1**	2.65e-05	0.00242%	0.0	1	7.37e-07	0.00132	0.000319	8.13e-05	0.0026%
**L1097**	**False**	**koll11**	2.65e-05	0.00241%	0.0	1	0.0104	0.00142	0.00324	4.09e-05	0.00131%
**OTU_8031**	**True**	**ABY1**	2.64e-05	0.00241%	0.0	1	0.00474	0.00156	0.000421	4e-05	0.00128%
**OTU_214**	**True**	**PBS-25**	2.64e-05	0.00241%	0.0	1	0.00681	0.000276	0.0018	3.71e-05	0.00119%
**L1872**	**False**	**OD1**	2.64e-05	0.0024%	0.0	1	0.00126	0.000384	6.74e-05	5.21e-05	0.00167%
**OTU_6683**	**True**	**ZB2**	2.63e-05	0.0024%	0.0	1	0.00112	2.28e-05	7.83e-05	4.38e-05	0.0014%
**L816**	**False**	**PBS-25**	2.63e-05	0.00239%	0.0	1	0.0205	0.00192	0.00236	2.18e-05	0.000698%
**OTU_258**	**True**	**koll11**	2.61e-05	0.00238%	0.0	1	0.0088	0.00153	0.00102	3.08e-05	0.000987%
**L725**	**False**	**PBS-25**	2.58e-05	0.00235%	0.0	1	0.0324	0.0032	0.00375	2.44e-05	0.000782%
**L1456**	**False**	**ZB2**	2.56e-05	0.00233%	0.0	1	0.000383	0.00491	0.00923	3.76e-05	0.0012%
**OTU_8251**	**True**	**Mb-NB09**	2.56e-05	0.00233%	0.0	1	0.00271	7.87e-05	0.000102	3.59e-05	0.00115%
**OTU_9036**	**True**	**Mb-NB09**	2.55e-05	0.00232%	0.0	1	0.000691	0.0	0.0	5.59e-05	0.00179%
**L193**	**False**	**ABY1**	2.54e-05	0.00232%	0.0	1	0.00733	0.00307	0.0023	9.43e-05	0.00302%
**L323**	**False**	**OD1**	2.54e-05	0.00231%	0.0	1	0.00556	0.000399	0.000181	1.66e-05	0.000531%
**L322**	**False**	**OD1**	2.53e-05	0.00231%	0.0	1	0.00564	0.000399	0.00019	1.62e-05	0.000517%
**L1658**	**False**	**OD1**	2.53e-05	0.0023%	0.0	1	0.00773	0.00226	0.000804	4.53e-05	0.00145%
**OTU_8240**	**True**	**ABY1**	2.53e-05	0.0023%	0.0	1	0.00177	0.000307	0.000581	9.91e-05	0.00317%
**OTU_8751**	**True**	**OD1**	2.51e-05	0.00229%	0.0	1	0.00359	0.00022	0.000749	4.31e-05	0.00138%
**OTU_2281**	**True**	**koll11**	2.51e-05	0.00228%	0.0	1	0.00295	5.65e-05	0.000354	3.91e-05	0.00125%
**L141**	**False**	**ABY1**	2.5e-05	0.00228%	0.0	1	0.00204	0.0188	0.00683	9.58e-05	0.00307%
**L1512**	**False**	**OD1**	2.49e-05	0.00227%	0.0	1	0.00567	0.000801	0.0053	8.02e-05	0.00257%
**L1312**	**False**	**OD1**	2.47e-05	0.00225%	0.0	1	0.000644	0.000136	0.0	6.04e-05	0.00193%
**OTU_2093**	**True**	**GIF10**	2.47e-05	0.00225%	0.0	1	0.00305	0.000166	0.000141	3.92e-05	0.00126%
**L248**	**False**	**ABY1**	2.46e-05	0.00224%	0.0	1	0.00658	0.00139	0.000179	2.73e-05	0.000875%
**L1197**	**False**	**koll11**	2.46e-05	0.00224%	0.0	1	0.00229	0.000142	0.000235	2.02e-05	0.000648%
**L822**	**False**	**PBS-25**	2.45e-05	0.00223%	0.0	1	0.0192	0.0018	0.00236	2.09e-05	0.00067%
**L403**	**False**	**ABY1**	2.44e-05	0.00222%	0.0	1	0.00805	0.0024	0.00106	5.82e-05	0.00186%
**L1278**	**False**	**TM7-3**	2.43e-05	0.00221%	0.0	1	0.00461	0.00027	0.000893	7.4e-05	0.00237%
**L424**	**False**	**OP11**	2.43e-05	0.00221%	0.0	1	0.00269	0.000622	0.00209	0.000112	0.00357%
**L4**	**False**	**ABY1**	2.42e-05	0.00221%	0.0	1	0.00262	0.000604	0.000162	3.37e-05	0.00108%
**L1365**	**False**	**ZB2**	2.42e-05	0.0022%	0.0	1	2.36e-06	0.00169	0.00166	6.63e-05	0.00212%
**OTU_8002**	**True**	**ABY1**	2.41e-05	0.0022%	0.0	1	0.00139	2.16e-05	1.89e-05	5.29e-05	0.00169%
**L868**	**False**	**koll11**	2.41e-05	0.0022%	0.0	1	0.0191	0.00216	0.00371	1.25e-05	0.0004%
**L1683**	**False**	**Mb-NB09**	2.41e-05	0.00219%	0.0	1	0.00307	0.0008	6.87e-05	4.56e-05	0.00146%
**OTU_7054**	**True**	**ABY1**	2.41e-05	0.00219%	0.0	1	0.0038	0.000396	9.49e-05	2.58e-05	0.000827%
**OTU_10762**	**True**	**OP11-4**	2.4e-05	0.00218%	0.0	1	0.00117	0.000201	1.83e-05	2.82e-05	0.000904%
**L1663**	**False**	**OD1**	2.39e-05	0.00218%	0.0	1	0.00332	0.000489	6.59e-05	1.93e-05	0.000619%
**OTU_49**	**True**	**OD1**	2.39e-05	0.00217%	0.0	1	0.000305	1.77e-05	0.0	4.96e-05	0.00159%
**OTU_4160**	**True**	**GIF10**	2.38e-05	0.00217%	0.0	1	0.000808	4.54e-07	0.0	5.02e-05	0.00161%
**L864**	**False**	**koll11**	2.38e-05	0.00217%	0.0	1	0.0198	0.00222	0.00389	1.17e-05	0.000376%
**L1523**	**False**	**ZB2**	2.38e-05	0.00217%	0.0	1	0.00223	0.000274	0.000102	2.77e-05	0.000887%
**L137**	**False**	**OD1**	2.38e-05	0.00217%	0.0	1	0.00933	0.0362	0.0206	0.000108	0.00345%
**OTU_6129**	**True**	**SM2F11**	2.37e-05	0.00216%	0.0	1	0.00295	0.000492	3.11e-05	4.11e-05	0.00132%
**L1771**	**False**	**OD1**	2.37e-05	0.00216%	0.0	1	0.00155	0.000112	7.25e-05	4.38e-05	0.0014%
**L1690**	**False**	**OD1**	2.36e-05	0.00215%	0.0	1	0.00545	0.000635	0.000174	1.02e-05	0.000327%
**OTU_7898**	**True**	**ABY1**	2.36e-05	0.00215%	0.0	1	1.62e-05	0.00262	0.00258	6.71e-05	0.00215%
**L1784**	**False**	**OD1**	2.35e-05	0.00214%	0.0	1	0.00442	0.000814	0.00043	2.24e-05	0.000716%
**L427**	**False**	**OP11**	2.34e-05	0.00213%	0.0	1	0.00128	5.88e-05	0.000189	4.08e-05	0.00131%
**L535**	**False**	**SBRH58**	2.34e-05	0.00213%	0.0	1	0.00161	6.35e-05	0.000116	5.64e-05	0.00181%

**L1798**	**False**	**OD1**	2.33e-05	0.00213%	0.0	1	0.0156	0.00154	0.000626	1.56e-05	0.0005%
**L297**	**False**	**ABY1**	2.32e-05	0.00212%	0.0	1	0.00104	0.0113	0.00876	4.92e-05	0.00158%
**OTU_6014**	**True**	**SM2F11**	2.32e-05	0.00211%	0.0	1	0.00546	0.00116	0.000232	3.09e-05	0.000991%
**L1558**	**False**	**ZB2**	2.31e-05	0.0021%	0.0	1	0.015	0.00175	0.00143	2.05e-05	0.000657%
**OTU_633**	**True**	**koll11**	2.3e-05	0.0021%	0.0	1	0.0183	0.00164	0.00218	1.37e-05	0.000439%
**L815**	**False**	**OP3**	2.3e-05	0.00209%	0.0	1	0.0218	0.00196	0.00236	1.68e-05	0.000538%
**L1509**	**False**	**OD1**	2.3e-05	0.00209%	0.0	1	0.00804	0.0014	0.00549	6.07e-05	0.00194%
**L94**	**False**	**ABY1**	2.28e-05	0.00207%	0.0	1	0.0176	0.0041	0.00072	1.69e-05	0.000542%
**L507**	**False**	**OP11-4**	2.28e-05	0.00207%	0.0	1	0.00379	0.000519	0.000706	3.35e-05	0.00107%
**L649**	**False**	**koll11**	2.27e-05	0.00207%	0.0	1	0.0167	0.00236	0.00459	1.44e-05	0.000461%
**OTU_423**	**True**	**PBS-25**	2.26e-05	0.00206%	0.0	1	0.00673	0.00149	0.000451	1.99e-05	0.000639%
**L1511**	**False**	**OD1**	2.25e-05	0.00205%	0.0	1	0.00804	0.00107	0.0054	5.08e-05	0.00163%
**L485**	**False**	**OP11-4**	2.24e-05	0.00204%	0.0	1	0.00197	0.000126	1.69e-05	2.48e-05	0.000795%
**L175**	**False**	**SM2F11**	2.23e-05	0.00203%	0.0	1	0.00188	0.000239	1.63e-05	3.19e-05	0.00102%
**OTU_5431**	**True**	**OD1**	2.18e-05	0.00199%	0.0	1	0.000642	3.06e-05	0.0	4.1e-05	0.00131%
**L974**	**False**	**koll11**	2.18e-05	0.00198%	0.0	1	0.00633	0.000815	0.000432	3.2e-05	0.00103%
**OTU_3387**	**True**	**PBS-25**	2.17e-05	0.00198%	0.0	1	0.0019	0.0	0.0	6.15e-05	0.00197%
**L1336**	**False**	**ZB2**	2.17e-05	0.00198%	0.0	1	0.00211	0.000168	0.000119	2.2e-05	0.000704%
**OTU_7967**	**True**	**ABY1**	2.17e-05	0.00197%	0.0	1	0.00289	5.32e-05	3.63e-05	2.11e-05	0.000675%
**L69**	**False**	**ABY1**	2.16e-05	0.00196%	0.0	1	0.00013	0.00837	0.00694	3.9e-05	0.00125%
**OTU_6576**	**True**	**ZB2**	2.13e-05	0.00194%	0.0	1	0.000578	1.47e-05	0.0	3.06e-05	0.00098%
**OTU_8044**	**True**	**WCHB1**	2.12e-05	0.00193%	0.0	1	0.000619	1.94e-05	0.0	2.9e-05	0.00093%
**L520**	**False**	**OP11-3**	2.11e-05	0.00192%	0.0	1	0.00165	0.0	0.0	4.36e-05	0.0014%
**L1720**	**False**	**Mb-NB09**	2.11e-05	0.00192%	0.0	1	0.00455	0.000754	0.000968	3.28e-05	0.00105%
**L370**	**False**	**OD1**	2.1e-05	0.00191%	0.0	1	0.00216	0.000332	2.69e-05	2.23e-05	0.000714%
**L1790**	**False**	**OD1**	2.1e-05	0.00191%	0.0	1	0.0034	0.000564	0.000357	1.79e-05	0.000573%
**L1714**	**False**	**Mb-NB09**	2.09e-05	0.0019%	0.0	1	0.00327	0.000287	0.000137	2.95e-05	0.000945%
**OTU_6061**	**True**	**ZB2**	2.09e-05	0.0019%	0.0	1	2.97e-05	0.00196	0.000123	4.1e-05	0.00131%
**L309**	**False**	**ABY1**	2.09e-05	0.0019%	0.0	1	3.62e-05	0.00458	0.00397	2.31e-05	0.000741%
**OTU_949**	**True**	**koll11**	2.09e-05	0.0019%	0.0	1	0.00544	0.000751	0.000179	2.96e-05	0.000949%
**L1836**	**False**	**OD1**	2.08e-05	0.0019%	0.0	1	0.00225	0.000449	0.000857	0.000133	0.00427%
**L1533**	**False**	**OD1**	2.07e-05	0.00189%	0.0	1	3.99e-06	0.000457	0.00111	5.37e-05	0.00172%
**L812**	**False**	**PBS-25**	2.07e-05	0.00189%	0.0	1	0.0134	0.00148	0.00469	3.52e-05	0.00113%
**OTU_9039**	**True**	**ABY1**	2.05e-05	0.00187%	0.0	1	0.0011	9.53e-06	3.57e-06	3.62e-05	0.00116%
**OTU_36**	**True**	**PBS-25**	2.05e-05	0.00187%	0.0	1	0.00332	0.000179	0.000241	4.88e-05	0.00156%
**OTU_8035**	**True**	**OD1**	2.05e-05	0.00186%	0.0	1	0.00168	0.00021	0.000483	9.5e-05	0.00304%
**OTU_927**	**True**	**PRR-12**	2.03e-05	0.00185%	0.0	1	0.00196	5.01e-05	1.6e-05	2.52e-05	0.000807%
**OTU_7950**	**True**	**ABY1**	2.02e-05	0.00184%	0.0	1	0.00131	0.000243	0.000169	6.15e-05	0.00197%
**L1572**	**False**	**ZB2**	2.01e-05	0.00183%	0.0	1	0.0019	0.000256	0.000611	4.66e-05	0.00149%
**OTU_7450**	**True**	**ABY1**	2.01e-05	0.00183%	0.0	1	0.00343	0.000608	0.000174	3.03e-05	0.000972%
**L158**	**False**	**ABY1**	2e-05	0.00182%	0.0	1	0.00278	0.000334	0.000101	2.08e-05	0.000668%
**L393**	**False**	**ABY1**	2e-05	0.00182%	0.0	1	0.00376	0.00017	0.000173	1.73e-05	0.000554%
**L1793**	**False**	**OD1**	2e-05	0.00182%	0.0	1	0.00205	0.000515	0.000331	3.88e-05	0.00124%
**OTU_380**	**True**	**koll11**	1.99e-05	0.00182%	0.0	1	0.00221	0.000322	4.11e-05	3.21e-05	0.00103%
**OTU_6571**	**True**	**ZB2**	1.99e-05	0.00181%	0.0	1	0.00237	0.0	3.51e-05	2.58e-05	0.000826%
**OTU_216**	**True**	**koll11**	1.99e-05	0.00181%	0.0	1	0.00921	0.00121	0.00295	3.73e-05	0.00119%
**OTU_7951**	**True**	**ABY1**	1.99e-05	0.00181%	0.0	1	0.00132	5.2e-05	0.0	3.22e-05	0.00103%
**L595**	**False**	**PRR-12**	1.98e-05	0.0018%	0.0	1	0.00451	0.000308	0.000116	1.66e-05	0.00053%
**L5**	**False**	**ABY1**	1.98e-05	0.0018%	0.0	1	0.00101	5.28e-05	0.000152	2.68e-05	0.00086%
**L99**	**False**	**ABY1**	1.98e-05	0.0018%	0.0	1	0.0113	0.00152	0.000309	1.74e-05	0.000556%
**L1178**	**False**	**koll11**	1.98e-05	0.0018%	0.0	1	0.00674	0.000452	0.000463	1.38e-05	0.000441%
**L1352**	**False**	**OD1**	1.97e-05	0.0018%	0.0	1	0.00323	0.00065	0.00118	8.54e-05	0.00273%
**L482**	**False**	**OP11-4**	1.97e-05	0.0018%	0.0	1	0.0106	0.00161	0.000937	1.21e-05	0.000387%
**L405**	**False**	**ABY1**	1.97e-05	0.0018%	0.0	1	0.00728	0.00232	0.000928	5.38e-05	0.00172%
**L308**	**False**	**ABY1**	1.97e-05	0.0018%	0.0	1	4.13e-05	0.00486	0.00425	2.27e-05	0.000726%
**OTU_2374**	**True**	**koll11**	1.95e-05	0.00178%	0.0	1	0.00104	2.62e-06	6.21e-07	3.76e-05	0.0012%
**OTU_8134**	**True**	**ZB2**	1.94e-05	0.00177%	0.0	1	0.000565	0.0	0.0	4.11e-05	0.00132%
**L162**	**False**	**ABY1**	1.94e-05	0.00177%	0.0	1	0.00208	0.000204	6.51e-05	2.34e-05	0.000751%
**L174**	**False**	**SM2F11**	1.93e-05	0.00176%	0.0	1	0.00246	0.000322	0.000252	2.38e-05	0.000761%
**OTU_10749**	**True**	**OP11-4**	1.92e-05	0.00175%	0.0	1	0.0148	0.00217	0.00208	3.8e-05	0.00122%
**L419**	**False**	**Bacteria**	1.92e-05	0.00175%	0.0	1	0.821	0.835	0.892	0.000182	0.00582%
**OTU_7995**	**True**	**ABY1**	1.92e-05	0.00174%	0.0	1	7.37e-07	0.000415	0.00149	6.04e-05	0.00194%
**OTU_1400**	**True**	**PBS-25**	1.91e-05	0.00174%	0.0	1	0.0103	0.000807	0.00129	2.24e-05	0.000717%
**OTU_2490**	**True**	**koll11**	1.91e-05	0.00174%	0.0	1	0.00605	0.000285	0.00139	4.9e-05	0.00157%
**L992**	**False**	**koll11**	1.91e-05	0.00174%	0.0	1	0.0108	0.00128	0.00254	3.53e-05	0.00113%

**OTU_7263**	**True**	**ABY1**	1.9e-05	0.00173%	0.0	1	0.00408	0.000576	0.000198	1.26e-05	0.000404%
**L1111**	**False**	**koll11**	1.89e-05	0.00172%	0.0	1	0.0332	0.0038	0.00417	6.56e-06	0.00021%
**OTU_8072**	**True**	**ABY1**	1.87e-05	0.00171%	0.0	1	0.000767	8.77e-05	0.00013	4.21e-05	0.00135%
**L30**	**False**	**OD1**	1.87e-05	0.00171%	0.0	1	0.129	0.152	0.0961	0.00028	0.00896%
**OTU_163**	**True**	**koll11**	1.86e-05	0.0017%	0.0	1	0.00822	0.00023	0.00376	3.19e-05	0.00102%
**L1463**	**False**	**ZB2**	1.85e-05	0.00168%	0.0	1	0.00031	0.00446	0.00687	2.93e-05	0.000938%
**OTU_8038**	**True**	**ABY1**	1.85e-05	0.00168%	0.0	1	0.000963	0.0	1.33e-06	3.53e-05	0.00113%
**L444**	**False**	**OP11-4**	1.85e-05	0.00168%	0.0	1	0.00153	9.98e-05	0.000932	7.74e-05	0.00248%
**L1786**	**False**	**OD1**	1.84e-05	0.00168%	0.0	1	0.0042	0.000564	0.000357	1.51e-05	0.000483%
**L76**	**False**	**ABY1**	1.84e-05	0.00168%	0.0	1	0.00226	0.000876	7.94e-05	5.77e-05	0.00185%
**OTU_7120**	**True**	**ABY1**	1.84e-05	0.00167%	0.0	1	0.00264	0.00113	0.000122	5.12e-05	0.00164%
**L1356**	**False**	**ZB2**	1.83e-05	0.00167%	0.0	1	0.00135	6.32e-05	9.89e-06	2.66e-05	0.000851%
**OTU_8074**	**True**	**ABY1**	1.83e-05	0.00167%	0.0	1	0.00013	0.003	0.00269	3.92e-05	0.00125%
**L129**	**False**	**ABY1**	1.81e-05	0.00165%	0.0	1	0.0016	5.64e-05	2.4e-05	2.87e-05	0.000919%
**OTU_8776**	**True**	**Mb-NB09**	1.8e-05	0.00164%	0.0	1	0.00282	0.000674	3e-05	3.55e-05	0.00114%
**L1862**	**False**	**OD1**	1.8e-05	0.00164%	0.0	1	0.00299	0.000578	0.000261	1.56e-05	0.000501%
**L856**	**False**	**OP3**	1.79e-05	0.00163%	0.0	1	0.0188	0.0018	0.0023	1.06e-05	0.000341%
**OTU_261**	**True**	**koll11**	1.79e-05	0.00163%	0.0	1	0.00158	0.000138	0.0	3.82e-05	0.00122%
**L1629**	**False**	**ZB2**	1.78e-05	0.00162%	0.0	1	0.00421	0.000863	0.00177	7.07e-05	0.00227%
**OTU_400**	**True**	**koll11**	1.77e-05	0.00162%	0.0	1	0.00169	0.000149	0.000315	5.42e-05	0.00174%
**L1457**	**False**	**ZB2**	1.77e-05	0.00162%	0.0	1	0.0	0.000298	0.00235	3.72e-05	0.00119%
**L96**	**False**	**ABY1**	1.77e-05	0.00161%	0.0	1	0.0144	0.00273	0.000524	1.15e-05	0.000369%
**L183**	**False**	**SM2F11**	1.77e-05	0.00161%	0.0	1	0.00924	0.00166	0.000295	1.17e-05	0.000376%
**L116**	**False**	**OD1**	1.76e-05	0.00161%	0.0	1	0.0156	0.0481	0.0305	6.71e-05	0.00215%
**L262**	**False**	**OD1**	1.76e-05	0.0016%	0.0	1	0.00905	0.00368	0.00216	4.97e-05	0.00159%
**L1056**	**False**	**koll11**	1.76e-05	0.0016%	0.0	1	0.00422	0.000431	0.000751	2.76e-05	0.000885%
**L326**	**False**	**ABY1**	1.76e-05	0.0016%	0.0	1	0.00111	6.83e-05	0.00011	3.05e-05	0.000976%
**L211**	**False**	**ABY1**	1.74e-05	0.00159%	0.0	1	0.00121	1.04e-05	2.25e-05	2.78e-05	0.000891%
**L851**	**False**	**koll11**	1.74e-05	0.00159%	0.0	1	0.0245	0.00286	0.00211	9.38e-06	0.000301%
**L483**	**False**	**OP11-4**	1.74e-05	0.00159%	0.0	1	0.00221	0.000213	2e-05	2e-05	0.00064%
**L1856**	**False**	**OD1**	1.73e-05	0.00157%	0.0	1	0.0036	0.000604	0.000285	1.11e-05	0.000357%
**L538**	**False**	**SJA-4**	1.72e-05	0.00156%	0.0	1	0.00567	0.000458	0.00297	7.83e-05	0.00251%
**L994**	**False**	**koll11**	1.71e-05	0.00156%	0.0	1	0.0107	0.00127	0.00253	3.2e-05	0.00102%
**OTU_6084**	**True**	**ZB2**	1.7e-05	0.00155%	0.0	1	3.81e-05	0.00431	0.00169	3.41e-05	0.00109%
**L1865**	**False**	**OD1**	1.7e-05	0.00155%	0.0	1	0.00164	0.000194	0.000194	2.23e-05	0.000715%
**L1528**	**False**	**OD1**	1.7e-05	0.00155%	0.0	1	0.00556	0.00121	0.002	4.06e-05	0.0013%
**OTU_32**	**True**	**Mb-NB09**	1.68e-05	0.00153%	0.0	1	0.000377	4.95e-05	8.9e-06	3.63e-05	0.00116%
**OTU_6635**	**True**	**OD1**	1.68e-05	0.00153%	0.0	1	0.000874	5.64e-06	3.78e-06	1.87e-05	0.000599%
**L1306**	**False**	**OD1**	1.68e-05	0.00153%	0.0	1	0.00704	0.000535	0.000845	2.13e-05	0.000681%
**OTU_358**	**True**	**koll11**	1.68e-05	0.00153%	0.0	1	0.00144	8.12e-05	0.000643	6.64e-05	0.00213%
**L1692**	**False**	**OD1**	1.68e-05	0.00153%	0.0	1	0.00375	0.000482	0.000105	1.1e-05	0.000352%
**L1778**	**False**	**OD1**	1.67e-05	0.00152%	0.0	1	0.00101	5.64e-06	3.78e-06	1.47e-05	0.00047%
**OTU_6663**	**True**	**ZB2**	1.67e-05	0.00152%	0.0	1	0.00199	0.000479	8.11e-05	3.2e-05	0.00103%
**OTU_6502**	**True**	**WCHB1-64**	1.66e-05	0.00151%	0.0	1	3.84e-05	0.000403	0.00123	4.82e-05	0.00154%
**L489**	**False**	**OP11-4**	1.66e-05	0.00151%	0.0	1	0.00688	0.00113	0.000826	2.19e-05	0.000702%
**OTU_1080**	**True**	**koll11**	1.65e-05	0.0015%	0.0	1	0.00567	0.000205	0.000775	2.95e-05	0.000946%
**OTU_6232**	**True**	**ZB2**	1.65e-05	0.0015%	0.0	1	5.79e-05	0.00123	0.00528	4.67e-05	0.00149%
**L1684**	**False**	**Mb-NB09**	1.65e-05	0.0015%	0.0	1	0.00292	0.000674	3.75e-05	3.07e-05	0.000985%
**OTU_7045**	**True**	**ABY1**	1.63e-05	0.00149%	0.0	1	7.16e-05	0.0184	0.00659	3.45e-05	0.00111%
**OTU_6203**	**True**	**OD1**	1.61e-05	0.00146%	0.0	1	5.6e-05	0.00615	0.00292	2.89e-05	0.000925%
**L380**	**False**	**OD1**	1.6e-05	0.00146%	0.0	1	0.000985	4.5e-05	5.3e-06	2.28e-05	0.000732%
**OTU_7022**	**True**	**ABY1**	1.6e-05	0.00146%	0.0	1	0.00013	0.00831	0.00692	2.94e-05	0.000943%
**OTU_217**	**True**	**koll11**	1.6e-05	0.00146%	0.0	1	0.00727	0.000675	0.00183	2.82e-05	0.000902%
**L251**	**False**	**ABY1**	1.6e-05	0.00146%	0.0	1	0.00556	0.00139	0.000179	2.39e-05	0.000765%
**L1044**	**False**	**koll11**	1.6e-05	0.00146%	0.0	1	0.0133	0.00166	0.00173	8.23e-06	0.000264%
**L101**	**False**	**ABY1**	1.6e-05	0.00146%	0.0	1	0.0107	0.00119	0.000302	1.34e-05	0.000429%
**L714**	**False**	**PBS-25**	1.59e-05	0.00145%	0.0	1	0.0149	0.00203	0.00355	2.57e-05	0.000825%
**L1175**	**False**	**koll11**	1.59e-05	0.00145%	0.0	1	0.0112	0.000971	0.00134	1.04e-05	0.000334%
**L1277**	**False**	**TM7**	1.59e-05	0.00144%	0.0	1	0.0054	0.000412	0.00107	3.91e-05	0.00125%
**OTU_7952**	**True**	**ABY1**	1.59e-05	0.00144%	0.0	1	0.000773	0.0	9.04e-07	2.51e-05	0.000804%
**L1117**	**False**	**koll11**	1.58e-05	0.00144%	0.0	1	0.0056	0.000328	0.0012	1.69e-05	0.00054%
**L737**	**False**	**PBS-25**	1.58e-05	0.00144%	0.0	1	0.0172	0.00146	0.0023	2.46e-05	0.000789%
**L226**	**False**	**ABY1**	1.58e-05	0.00144%	0.0	1	0.00122	9.87e-05	0.0	3.27e-05	0.00105%
**L877**	**False**	**koll11**	1.58e-05	0.00144%	0.0	1	0.0181	0.00204	0.00368	8.98e-06	0.000288%

**OTU_994**	**True**	**[Acetothermia]**	1.57e-05	0.00143%	0.0	1	0.000404	0.0	0.0	4.1e-05	0.00131%
**OTU_9054**	**True**	**ABY1**	1.55e-05	0.00141%	0.0	1	0.000228	0.0	0.000897	6.9e-05	0.00221%
**OTU_347**	**True**	**koll11**	1.55e-05	0.00141%	0.0	1	0.00245	0.000186	0.00104	5.11e-05	0.00164%
**OTU_567**	**True**	**koll11**	1.54e-05	0.0014%	0.0	1	0.00119	3.33e-05	0.0	3.18e-05	0.00102%
**OTU_419**	**True**	**koll11**	1.52e-05	0.00139%	0.0	1	0.00131	1.91e-05	0.0	2.83e-05	0.000907%
**L1857**	**False**	**OD1**	1.52e-05	0.00138%	0.0	1	0.000613	2.61e-05	2.36e-05	2.22e-05	0.000711%
**L1892**	**False**	**OD1**	1.51e-05	0.00138%	0.0	1	0.00242	0.000516	0.000191	2.72e-05	0.000871%
**L1125**	**False**	**koll11**	1.51e-05	0.00138%	0.0	1	0.00335	0.000233	0.000126	2.77e-05	0.000887%
**L136**	**False**	**OD1**	1.51e-05	0.00138%	0.0	1	0.00941	0.0362	0.0206	6.91e-05	0.00221%
**OTU_6177**	**True**	**OD1**	1.5e-05	0.00137%	0.0	1	0.000672	0.000184	0.0	2.98e-05	0.000955%
**L1039**	**False**	**koll11**	1.5e-05	0.00136%	0.0	1	0.00782	0.000692	0.00215	2.54e-05	0.000813%
**L1049**	**False**	**koll11**	1.49e-05	0.00136%	0.0	1	0.00236	0.000322	4.11e-05	2.24e-05	0.000717%
**OTU_6298**	**True**	**OD1**	1.49e-05	0.00136%	0.0	1	0.00242	0.000175	0.000667	3.9e-05	0.00125%
**OTU_10764**	**True**	**OP11-4**	1.49e-05	0.00135%	0.0	1	0.00525	0.001	0.000599	2.7e-05	0.000864%
**L514**	**False**	**OP11-3**	1.48e-05	0.00135%	0.0	1	0.00185	8.71e-05	7.23e-05	3.58e-05	0.00115%
**OTU_7223**	**True**	**ABY1**	1.47e-05	0.00134%	0.0	1	0.000754	0.000436	9.68e-06	3.64e-05	0.00117%
**OTU_8250**	**True**	**ABY1**	1.47e-05	0.00134%	0.0	1	0.00174	0.000876	7.94e-05	5.67e-05	0.00182%
**OTU_2352**	**True**	**SJA-4**	1.47e-05	0.00134%	0.0	1	0.000817	0.0	0.0	3.03e-05	0.000972%
**L1037**	**False**	**koll11**	1.47e-05	0.00133%	0.0	1	0.00853	0.000714	0.00216	2.1e-05	0.000673%
**L1894**	**False**	**OD1**	1.46e-05	0.00133%	0.0	1	0.00224	0.000452	0.000174	2.61e-05	0.000836%
**OTU_247**	**True**	**koll11**	1.46e-05	0.00133%	0.0	1	0.00407	0.000771	0.000677	4.26e-05	0.00137%
**L29**	**False**	**OD1**	1.46e-05	0.00133%	0.0	1	0.149	0.154	0.0974	0.00018	0.00578%
**OTU_6086**	**True**	**ZB2**	1.44e-05	0.00131%	0.0	1	0.000689	1.13e-05	2e-05	3.18e-05	0.00102%
**OTU_8007**	**True**	**ABY1**	1.42e-05	0.00129%	0.0	1	0.00099	1.29e-05	0.0	3.12e-05	0.000999%
**L1472**	**False**	**ZB2**	1.42e-05	0.00129%	0.0	1	0.00493	0.00119	0.00114	2.63e-05	0.000841%
**OTU_5425**	**True**	**OD1**	1.41e-05	0.00128%	0.0	1	0.00229	0.000277	3.15e-05	1.75e-05	0.000559%
**OTU_6667**	**True**	**ABY1**	1.41e-05	0.00128%	0.0	1	0.000229	0.0387	0.0113	2.25e-05	0.000722%
**L346**	**False**	**ABY1**	1.4e-05	0.00128%	0.0	1	0.00597	0.000708	0.000863	2.13e-05	0.000681%
**L274**	**False**	**ABY1**	1.4e-05	0.00127%	0.0	1	0.000213	0.00217	0.00132	4.59e-05	0.00147%
**OTU_2335**	**True**	**SBRH58**	1.39e-05	0.00127%	0.0	1	0.00126	6.35e-05	0.000116	4.65e-05	0.00149%
**OTU_1554**	**True**	**PBS-25**	1.38e-05	0.00126%	0.0	1	0.000976	0.0	0.0	1.78e-05	0.000569%
**L1722**	**False**	**Mb-NB09**	1.38e-05	0.00126%	0.0	1	0.00451	0.000732	0.000967	2.16e-05	0.000693%
**L862**	**False**	**koll11**	1.37e-05	0.00125%	0.0	1	0.0253	0.00297	0.00407	5.24e-06	0.000168%
**L1753**	**False**	**OD1**	1.37e-05	0.00125%	0.0	1	0.000542	0.000699	0.00192	0.000143	0.0046%
**OTU_4950**	**True**	**[Acetothermia]**	1.36e-05	0.00124%	0.0	1	0.000409	3.22e-05	0.0	2.92e-05	0.000937%
**L1696**	**False**	**Mb-NB09**	1.36e-05	0.00124%	0.0	1	0.00111	0.000184	5.92e-05	2.02e-05	0.000647%
**OTU_7990**	**True**	**ABY1**	1.36e-05	0.00124%	0.0	1	0.000857	0.000115	0.0	2.75e-05	0.00088%
**L991**	**False**	**koll11**	1.36e-05	0.00123%	0.0	1	0.0127	0.00165	0.00357	2.16e-05	0.000692%
**L995**	**False**	**koll11**	1.35e-05	0.00123%	0.0	1	0.00844	0.00122	0.00251	3.02e-05	0.000969%
**OTU_327**	**True**	**koll11**	1.35e-05	0.00123%	0.0	1	0.00135	0.0	0.0	2.19e-05	0.000701%
**L1045**	**False**	**koll11**	1.34e-05	0.00122%	0.0	1	0.0118	0.00161	0.00153	8.07e-06	0.000258%
**OTU_323**	**True**	**koll11**	1.33e-05	0.00121%	0.0	1	0.00172	0.000137	4.14e-05	2.28e-05	0.00073%
**L264**	**False**	**OD1**	1.32e-05	0.0012%	0.0	1	0.00431	0.00212	0.00174	0.000124	0.00397%
**OTU_292**	**True**	**PBS-25**	1.32e-05	0.0012%	0.0	1	0.00766	0.000603	0.000832	2.22e-05	0.000711%
**L1262**	**False**	**TM7-1**	1.32e-05	0.0012%	0.0	1	0.00239	0.000263	7.09e-05	1.66e-05	0.000531%
**L1611**	**False**	**ZB2**	1.31e-05	0.0012%	0.0	1	0.00197	0.000116	0.000108	1.69e-05	0.000541%
**OTU_6073**	**True**	**ZB2**	1.31e-05	0.0012%	0.0	1	6.88e-05	0.00613	0.00508	6.09e-05	0.00195%
**OTU_6914**	**True**	**ZB2**	1.31e-05	0.0012%	0.0	1	0.000983	9.71e-05	0.000104	2.98e-05	0.000953%
**L1282**	**False**	**TM7-3**	1.31e-05	0.00119%	0.0	1	0.00454	0.000237	9.33e-05	2.45e-05	0.000785%
**L1824**	**False**	**OD1**	1.31e-05	0.00119%	0.0	1	0.00141	0.000249	0.000202	4.55e-05	0.00146%
**L328**	**False**	**ABY1**	1.3e-05	0.00118%	0.0	1	0.00111	6.42e-05	0.000103	2.21e-05	0.000709%
**L14**	**False**	**OD1**	1.29e-05	0.00118%	0.0	1	0.00178	9.46e-05	6.93e-06	1.67e-05	0.000536%
**L119**	**False**	**ABY1**	1.29e-05	0.00117%	0.0	1	0.00115	0.0108	0.00791	4.1e-05	0.00131%
**L126**	**False**	**OD1**	1.27e-05	0.00116%	0.0	1	0.0144	0.0368	0.0211	0.000101	0.00322%
**L1838**	**False**	**OD1**	1.27e-05	0.00115%	0.0	1	0.00214	0.000426	0.000351	5.55e-05	0.00178%
**OTU_6064**	**True**	**ZB2**	1.26e-05	0.00115%	0.0	1	4.86e-05	0.00217	0.00235	3.91e-05	0.00125%
**L1195**	**False**	**koll11**	1.26e-05	0.00115%	0.0	1	0.00339	0.000347	0.000824	1.7e-05	0.000546%
**L979**	**False**	**koll11**	1.25e-05	0.00114%	0.0	1	0.00389	0.000578	0.000458	2.09e-05	0.000668%
**OTU_10801**	**True**	**OP11-4**	1.25e-05	0.00114%	0.0	1	0.00163	0.000124	0.000227	1.61e-05	0.000515%
**L589**	**False**	**PRR-12**	1.25e-05	0.00114%	0.0	1	0.0117	0.00137	0.000811	1.34e-05	0.000428%
**L84**	**False**	**ABY1**	1.25e-05	0.00114%	0.0	1	0.00168	7.15e-05	0.0	1.66e-05	0.000531%
**OTU_6170**	**True**	**ZB2**	1.25e-05	0.00113%	0.0	1	0.000748	6.49e-05	0.0	2.1e-05	0.000674%
**OTU_1087**	**True**	**OP3**	1.24e-05	0.00113%	0.0	1	0.000539	0.0	4.95e-06	2.41e-05	0.000771%
**L1905**	**False**	**OD1**	1.24e-05	0.00113%	0.0	1	0.0014	6.82e-05	1.12e-05	1.32e-05	0.000423%
**L1244**	**False**	**TM7-1**	1.24e-05	0.00113%	0.0	1	0.00175	0.0114	0.00877	4.98e-05	0.0016%

**L82**	**False**	**ABY1**	1.23e-05	0.00112%	0.0	1	0.00203	0.000154	1.57e-05	1.46e-05	0.000467%
**L1048**	**False**	**koll11**	1.22e-05	0.00111%	0.0	1	0.00643	0.00109	0.000718	1.85e-05	0.000593%
**L1608**	**False**	**ZB2**	1.22e-05	0.00111%	0.0	1	0.00271	0.000144	0.000247	1.36e-05	0.000434%
**OTU_7373**	**True**	**OD1**	1.22e-05	0.00111%	0.0	1	0.001	0.000378	0.00029	6.72e-05	0.00215%
**L1245**	**False**	**TM7-1**	1.2e-05	0.0011%	0.0	1	0.00105	0.0113	0.00871	3.85e-05	0.00123%
**OTU_2622**	**True**	**SJA-4**	1.2e-05	0.00109%	0.0	1	0.0	0.0	0.000969	2.78e-05	0.000891%
**L1199**	**False**	**koll11**	1.2e-05	0.00109%	0.0	1	0.00218	0.000119	0.000231	1.12e-05	0.000358%
**OTU_8016**	**True**	**TM7-3**	1.19e-05	0.00108%	0.0	1	0.0	3.27e-05	0.000799	3.2e-05	0.00103%
**L1816**	**False**	**OD1**	1.19e-05	0.00108%	0.0	1	0.000476	4.44e-05	1.05e-05	2.91e-05	0.000933%
**L1012**	**False**	**koll11**	1.19e-05	0.00108%	0.0	1	0.0296	0.00345	0.00396	4.29e-06	0.000137%
**OTU_7164**	**True**	**ABY1**	1.17e-05	0.00107%	0.0	1	0.000292	0.0	0.0	3.06e-05	0.000979%
**OTU_8135**	**True**	**EW055**	1.17e-05	0.00106%	0.0	1	0.00236	0.000236	0.0	3.65e-05	0.00117%
**OTU_4911**	**True**	**OD1**	1.15e-05	0.00105%	0.0	1	0.000876	4.76e-05	2.33e-06	2.23e-05	0.000714%
**L534**	**False**	**TM6**	1.15e-05	0.00104%	0.0	1	0.00728	0.000522	0.00308	3.76e-05	0.00121%
**OTU_8948**	**True**	**TM7**	1.14e-05	0.00104%	0.0	1	0.000641	8.55e-05	3.62e-05	3.5e-05	0.00112%
**L113**	**False**	**ABY1**	1.14e-05	0.00104%	0.0	1	5.77e-06	0.00289	0.0016	1.78e-05	0.000571%
**L1711**	**False**	**OD1**	1.14e-05	0.00104%	0.0	1	0.00402	0.00054	0.000178	1.48e-05	0.000474%
**L283**	**False**	**ABY1**	1.14e-05	0.00104%	0.0	1	0.00821	0.0232	0.026	5.42e-05	0.00174%
**OTU_201**	**True**	**OD1**	1.14e-05	0.00104%	0.0	1	0.000268	0.0	8.8e-06	2.66e-05	0.000851%
**OTU_5635**	**True**	**OD1**	1.14e-05	0.00104%	0.0	1	0.000386	0.0	4.9e-06	2.45e-05	0.000786%
**OTU_409**	**True**	**koll11**	1.14e-05	0.00103%	0.0	1	0.00134	3.16e-05	0.0	2.71e-05	0.000868%
**L437**	**False**	**OP11**	1.13e-05	0.00103%	0.0	1	0.000934	2.01e-05	0.0	1.21e-05	0.000387%
**L1131**	**False**	**koll11**	1.12e-05	0.00102%	0.0	1	0.0021	2.68e-05	0.000162	1.57e-05	0.000503%
**OTU_7442**	**True**	**TM7-1**	1.12e-05	0.00102%	0.0	1	0.00107	0.000263	7.09e-05	2.5e-05	0.000801%
**OTU_8916**	**True**	**MJK10**	1.11e-05	0.00101%	0.0	1	0.000863	6.49e-05	0.000213	2.44e-05	0.000781%
**L802**	**False**	**OP3**	1.11e-05	0.00101%	0.0	1	0.046	0.00448	0.0104	8.51e-06	0.000273%
**L978**	**False**	**koll11**	1.09e-05	0.000997%	0.0	1	0.00426	0.000578	0.000458	1.73e-05	0.000553%
**OTU_7941**	**True**	**ABY1**	1.09e-05	0.000996%	0.0	1	0.000991	1.41e-05	3.7e-05	1.6e-05	0.000513%
**OTU_205**	**True**	**koll11**	1.09e-05	0.000989%	0.0	1	0.00562	0.000522	0.00036	9.86e-06	0.000316%
**OTU_7982**	**True**	**ABY1**	1.08e-05	0.000985%	0.0	1	0.00318	0.000488	9.59e-05	1.38e-05	0.000441%
**OTU_7840**	**True**	**ABY1**	1.08e-05	0.000982%	0.0	1	0.00251	0.000226	0.000147	1.83e-05	0.000586%
**L1597**	**False**	**OD1**	1.07e-05	0.000979%	0.0	1	0.000227	0.00627	0.00345	2.28e-05	0.00073%
**L1832**	**False**	**OD1**	1.07e-05	0.000978%	0.0	1	0.00233	0.000453	0.00102	6.75e-05	0.00216%
**L1770**	**False**	**OD1**	1.07e-05	0.000974%	0.0	1	0.00228	0.000228	0.000132	1.94e-05	0.00062%
**OTU_6586**	**True**	**OD1**	1.07e-05	0.000973%	0.0	1	0.00187	0.000426	0.000319	5.95e-05	0.00191%
**L80**	**False**	**OD1**	1.07e-05	0.000973%	0.0	1	0.0417	0.0562	0.0333	0.000131	0.00421%
**L1486**	**False**	**ZB2**	1.06e-05	0.00097%	0.0	1	0.00303	0.000464	0.000627	2.22e-05	0.000712%
**OTU_276**	**True**	**koll11**	1.06e-05	0.000969%	0.0	1	0.00258	0.000128	8.97e-05	1.88e-05	0.000602%
**OTU_8127**	**True**	**ABY1**	1.06e-05	0.000968%	0.0	1	0.0	4.23e-05	0.000748	1.59e-05	0.000511%
**L188**	**False**	**SM2F11**	1.06e-05	0.000968%	0.0	1	0.00575	0.00116	0.000232	1.36e-05	0.000434%
**L726**	**False**	**PBS-25**	1.06e-05	0.000964%	0.0	1	0.00898	0.000932	0.00106	1.42e-05	0.000455%
**L417**	**False**	**Bacteria**	1.04e-05	0.00095%	0.0	1	0.821	0.835	0.892	9.93e-05	0.00318%
**L1662**	**False**	**OD1**	1.03e-05	0.000941%	0.0	1	0.00671	0.00138	0.000778	1.07e-05	0.000343%
**OTU_7166**	**True**	**ABY1**	1.03e-05	0.000938%	0.0	1	0.00079	0.0	0.0	2.22e-05	0.00071%
**OTU_8910**	**True**	**TM7-1**	1.03e-05	0.000937%	0.0	1	0.000491	0.0	0.0	1.9e-05	0.000608%
**L1610**	**False**	**ZB2**	1.03e-05	0.000936%	0.0	1	0.00255	0.000139	0.000167	1.27e-05	0.000407%
**OTU_8010**	**True**	**ABY1**	1.03e-05	0.000935%	0.0	1	0.000404	0.0	2.02e-06	2.75e-05	0.000881%
**L243**	**False**	**ABY1**	1.02e-05	0.000929%	0.0	1	0.001	2.79e-05	0.00106	6.19e-05	0.00198%
**L284**	**False**	**ABY1**	1.02e-05	0.000927%	0.0	1	0.00717	0.0118	0.0172	9.54e-05	0.00306%
**L1799**	**False**	**OD1**	1.02e-05	0.000927%	0.0	1	0.00141	2.46e-05	3.82e-05	9e-06	0.000288%
**L1096**	**False**	**koll11**	1.02e-05	0.000926%	0.0	1	0.0121	0.00154	0.00392	1.32e-05	0.000422%
**OTU_6987**	**True**	**OD1**	1.01e-05	0.000923%	0.0	1	0.00542	0.00124	0.000358	1.79e-05	0.000575%
**L79**	**False**	**OD1**	1.01e-05	0.000922%	0.0	1	0.0417	0.0589	0.0359	0.000125	0.004%
**L1018**	**False**	**koll11**	1.01e-05	0.00092%	0.0	1	0.00641	0.000809	0.00079	8.65e-06	0.000277%
**L1559**	**False**	**ZB2**	1.01e-05	0.00092%	0.0	1	0.00958	0.000825	0.000482	1.28e-05	0.000409%
**OTU_8013**	**True**	**ABY1**	1.01e-05	0.000919%	0.0	1	5.72e-06	0.000554	0.00196	3.08e-05	0.000988%
**L1249**	**False**	**TM7-1**	1.01e-05	0.000918%	0.0	1	0.00102	8.05e-05	4e-06	9.82e-06	0.000315%
**L1179**	**False**	**GIF10**	1e-05	0.000914%	0.0	1	0.00535	0.000437	0.00042	9.49e-06	0.000304%
**L269**	**False**	**OD1**	1e-05	0.000913%	0.0	1	0.00549	0.00432	0.00129	5.57e-05	0.00178%
**L1886**	**False**	**OD1**	9.98e-06	0.000909%	0.0	1	0.00294	0.000617	0.000218	1.41e-05	0.000453%
**L1290**	**False**	**TM7-3**	9.98e-06	0.000909%	0.0	1	0.00412	0.000237	9.33e-05	1.98e-05	0.000635%
**L540**	**False**	**SJA-4**	9.98e-06	0.000909%	0.0	1	0.00398	0.000458	0.00297	6.47e-05	0.00207%
**L8**	**False**	**ABY1**	9.96e-06	0.000907%	0.0	1	0.00161	0.000551	9.68e-06	2.01e-05	0.000643%
**OTU_1149**	**True**	**koll11**	9.94e-06	0.000906%	0.0	1	0.000663	0.000148	6.99e-05	2.75e-05	0.00088%
**L334**	**False**	**OD1**	9.94e-06	0.000905%	0.0	1	0.000903	0.00012	5.8e-05	1.94e-05	0.000622%

**OTU_9845**	**True**	**SC3**	9.9e-06	0.000902%	0.0	1	0.000495	0.0	0.0	1.9e-05	0.000608%
**L773**	**False**	**PBS-25**	9.87e-06	0.000899%	0.0	1	0.00299	0.000273	0.000408	3.04e-05	0.000975%
**L1055**	**False**	**koll11**	9.83e-06	0.000895%	0.0	1	0.0047	0.000475	0.000778	1.41e-05	0.000452%
**L1532**	**False**	**OD1**	9.82e-06	0.000895%	0.0	1	0.00459	0.000778	0.00181	2.61e-05	0.000836%
**L876**	**False**	**koll11**	9.74e-06	0.000887%	0.0	1	0.0183	0.00209	0.00369	5.53e-06	0.000177%
**OTU_6179**	**True**	**ZB2**	9.72e-06	0.000885%	0.0	1	0.00053	1.5e-05	9.55e-06	1.22e-05	0.000389%
**OTU_270**	**True**	**koll11**	9.69e-06	0.000883%	0.0	1	0.00071	2.21e-05	9.5e-06	2.09e-05	0.000669%
**L128**	**False**	**OD1**	9.67e-06	0.000881%	0.0	1	0.0144	0.0367	0.0211	7.8e-05	0.0025%
**L41**	**False**	**OD1**	9.65e-06	0.000879%	0.0	1	0.0048	0.000185	0.00109	1.01e-05	0.000322%
**L1606**	**False**	**OD1**	9.64e-06	0.000878%	0.0	1	0.00978	0.00254	0.00295	1.86e-05	0.000595%
**OTU_5954**	**True**	**OD1**	9.61e-06	0.000875%	0.0	1	0.000655	0.0	0.0	2.02e-05	0.000646%
**L961**	**False**	**koll11**	9.58e-06	0.000873%	0.0	1	0.02	0.00161	0.00111	6.84e-06	0.000219%
**OTU_6699**	**True**	**ZB2**	9.56e-06	0.000871%	0.0	1	0.000345	2.69e-06	0.0	1.87e-05	0.0006%
**OTU_7050**	**True**	**ABY1**	9.48e-06	0.000864%	0.0	1	0.000763	2.97e-05	0.000157	2.24e-05	0.000719%
**OTU_229**	**True**	**koll11**	9.34e-06	0.00085%	0.0	1	0.00317	0.00058	0.000482	1.11e-05	0.000354%
**OTU_6990**	**True**	**ZB2**	9.32e-06	0.000849%	0.0	1	0.000128	0.00264	0.00103	3.6e-05	0.00115%
**OTU_6178**	**True**	**ZB2**	9.25e-06	0.000842%	0.0	1	0.00388	0.00016	0.000185	1.52e-05	0.000485%
**L1702**	**False**	**OD1**	9.23e-06	0.000841%	0.0	1	0.00249	0.000189	4.59e-05	9.67e-06	0.00031%
**OTU_5362**	**True**	**ZB2**	9.22e-06	0.00084%	0.0	1	0.000764	2.12e-05	3.57e-06	1.56e-05	0.000501%
**OTU_9171**	**True**	**Mb-NB09**	9.19e-06	0.000837%	0.0	1	0.000912	6.76e-05	0.0	1.33e-05	0.000427%
**L395**	**False**	**ABY1**	9.13e-06	0.000832%	0.0	1	0.00361	0.00017	0.000155	7.95e-06	0.000255%
**L1190**	**False**	**koll11**	9.08e-06	0.000827%	0.0	1	0.00139	1.48e-05	4.3e-05	1.25e-05	0.000399%
**L1551**	**False**	**ZB2**	9.06e-06	0.000826%	0.0	1	0.00631	0.00132	0.000704	1.52e-05	0.000488%
**L492**	**False**	**OP11-4**	9.02e-06	0.000821%	0.0	1	0.00152	0.000274	9.09e-05	1.08e-05	0.000347%
**OTU_2377**	**True**	**SJA-4**	9.01e-06	0.000821%	0.0	1	0.000643	0.0	0.0	1.87e-05	0.000598%
**L776**	**False**	**PBS-25**	9e-06	0.00082%	0.0	1	0.00209	0.000216	0.00011	2.57e-05	0.000822%
**L1821**	**False**	**OD1**	9e-06	0.00082%	0.0	1	0.000951	4.76e-05	2.4e-06	1.55e-05	0.000498%
**L1547**	**False**	**ZB2**	8.99e-06	0.000819%	0.0	1	0.0029	0.00036	0.00013	1.06e-05	0.000339%
**OTU_8003**	**True**	**d153**	8.98e-06	0.000818%	0.0	1	0.000315	6.41e-07	0.0	1.85e-05	0.000594%
**OTU_7940**	**True**	**ABY1**	8.94e-06	0.000814%	0.0	1	0.00279	0.000147	0.000136	9.22e-06	0.000295%
**L277**	**False**	**ABY1**	8.91e-06	0.000812%	0.0	1	0.0118	0.0237	0.0261	8.32e-05	0.00266%
**L607**	**False**	**PRR-12**	8.9e-06	0.000811%	0.0	1	0.002	3.67e-06	0.0	1.02e-05	0.000328%
**OTU_6039**	**True**	**OD1**	8.89e-06	0.00081%	0.0	1	0.000299	1.43e-05	4.16e-05	3.21e-05	0.00103%
**L1694**	**False**	**OD1**	8.89e-06	0.00081%	0.0	1	0.0036	0.000482	0.000105	6.71e-06	0.000215%
**OTU_5496**	**True**	**ZB2**	8.89e-06	0.000809%	0.0	1	0.00188	0.000586	0.00117	8.69e-05	0.00278%
**OTU_10750**	**True**	**OP11-4**	8.88e-06	0.000809%	0.0	1	0.000425	0.0	0.0	1.91e-05	0.000611%
**OTU_8764**	**True**	**ABY1**	8.87e-06	0.000808%	0.0	1	0.000694	9.54e-05	7.42e-08	1.67e-05	0.000535%
**L1724**	**False**	**Mb-NB09**	8.83e-06	0.000804%	0.0	1	0.00399	0.000603	0.000939	1.49e-05	0.000477%
**OTU_8147**	**True**	**ABY1**	8.78e-06	0.0008%	0.0	1	0.000529	4.81e-06	3.16e-05	1.15e-05	0.000369%
**L139**	**False**	**OD1**	8.77e-06	0.000799%	0.0	1	0.00922	0.0212	0.00734	7.69e-05	0.00246%
**L294**	**False**	**ABY1**	8.76e-06	0.000798%	0.0	1	0.0067	0.00777	0.0138	0.000124	0.00398%
**L1607**	**False**	**OD1**	8.73e-06	0.000795%	0.0	1	0.005	0.00168	0.00118	2.27e-05	0.000728%
**OTU_7946**	**True**	**ABY1**	8.72e-06	0.000794%	0.0	1	0.000243	0.0	0.0	1.62e-05	0.000519%
**L854**	**False**	**OP3**	8.61e-06	0.000784%	0.0	1	0.0209	0.00202	0.00241	4.65e-06	0.000149%
**L55**	**False**	**ABY1**	8.6e-06	0.000783%	0.0	1	0.00341	0.000106	0.0	1.8e-05	0.000575%
**OTU_8823**	**True**	**OD1**	8.58e-06	0.000781%	0.0	1	0.000881	0.0	0.0	1.79e-05	0.000572%
**OTU_6986**	**True**	**ZB2**	8.56e-06	0.00078%	0.0	1	0.000655	0.000275	0.000161	3.85e-05	0.00123%
**OTU_4488**	**True**	**PRR-12**	8.54e-06	0.000778%	0.0	1	0.000245	0.0	0.0	1.82e-05	0.000584%
**L147**	**False**	**OD1**	8.53e-06	0.000777%	0.0	1	0.00432	0.00093	0.000217	9.64e-06	0.000309%
**OTU_9143**	**True**	**OD1**	8.52e-06	0.000776%	0.0	1	0.0	0.000131	0.000283	4.72e-05	0.00151%
**OTU_2351**	**True**	**SJA-4**	8.5e-06	0.000774%	0.0	1	0.000437	0.0	0.0	1.76e-05	0.000563%
**OTU_9178**	**True**	**ABY1**	8.48e-06	0.000773%	0.0	1	0.000521	0.0	7.42e-08	1.02e-05	0.000325%
**L1813**	**False**	**OD1**	8.48e-06	0.000772%	0.0	1	0.00237	0.000145	0.000183	1.38e-05	0.000442%
**OTU_8334**	**True**	**ABY1**	8.42e-06	0.000767%	0.0	1	0.000308	0.0	0.0	1.82e-05	0.000583%
**L107**	**False**	**ABY1**	8.34e-06	0.00076%	0.0	1	0.00462	0.000686	0.000429	6e-06	0.000192%
**OTU_2076**	**True**	**PRR-12**	8.33e-06	0.000759%	0.0	1	0.000747	0.0	7.42e-08	1.24e-05	0.000397%
**L165**	**False**	**ABY1**	8.32e-06	0.000758%	0.0	1	0.00193	0.000161	5.72e-05	9.81e-06	0.000314%
**L356**	**False**	**ABY1**	8.31e-06	0.000757%	0.0	1	0.000561	2.96e-05	4.68e-06	1.06e-05	0.00034%
**OTU_7350**	**True**	**ABY1**	8.3e-06	0.000756%	0.0	1	5.77e-06	0.0012	0.000451	1.52e-05	0.000487%
**L425**	**False**	**OP11**	8.29e-06	0.000756%	0.0	1	0.00269	0.000622	0.00209	3.81e-05	0.00122%
**L376**	**False**	**OD1**	8.14e-06	0.000742%	0.0	1	0.00136	4.5e-05	5.38e-06	7.36e-06	0.000236%
**OTU_895**	**True**	**PRR-12**	8.14e-06	0.000742%	0.0	1	0.00131	0.000104	2.33e-05	1.22e-05	0.000389%
**L1022**	**False**	**koll11**	8.12e-06	0.00074%	0.0	1	0.00293	0.000193	0.000308	1.13e-05	0.000363%
**L827**	**False**	**PBS-25**	8.12e-06	0.000739%	0.0	1	0.00375	0.000411	0.000376	1.95e-05	0.000625%
**OTU_6183**	**True**	**WCHB1**	8.07e-06	0.000735%	0.0	1	0.00075	5.1e-05	0.000189	2.84e-05	0.000908%

**OTU_349**	**True**	**koll11**	8.06e-06	0.000735%	0.0	1	0.00214	0.000222	0.000113	1.66e-05	0.000532%
**OTU_7850**	**True**	**ABY1**	8.02e-06	0.000731%	0.0	1	0.0002	0.0	1.4e-06	1.77e-05	0.000567%
**L172**	**False**	**OD1**	7.96e-06	0.000725%	0.0	1	0.0324	0.00677	0.0044	5.02e-06	0.000161%
**L1058**	**False**	**koll11**	7.96e-06	0.000725%	0.0	1	0.00303	0.000398	0.000751	1.97e-05	0.000632%
**OTU_8768**	**True**	**Mb-NB09**	7.94e-06	0.000723%	0.0	1	0.00171	0.000153	6.89e-05	6.2e-06	0.000199%
**L768**	**False**	**PBS-25**	7.91e-06	0.000721%	0.0	1	0.00416	0.000323	0.000443	1.51e-05	0.000484%
**L1462**	**False**	**ZB2**	7.91e-06	0.00072%	0.0	1	0.000383	0.00461	0.00688	1.24e-05	0.000397%
**OTU_6669**	**True**	**ZB2**	7.91e-06	0.00072%	0.0	1	1.06e-05	0.0	0.000365	2.11e-05	0.000677%
**OTU_325**	**True**	**koll11**	7.86e-06	0.000716%	0.0	1	0.00134	1.16e-05	0.0	1.36e-05	0.000436%
**OTU_415**	**True**	**koll11**	7.84e-06	0.000714%	0.0	1	0.000675	0.0	0.0	2.14e-05	0.000686%
**L234**	**False**	**ABY1**	7.82e-06	0.000713%	0.0	1	0.00418	0.000265	0.000281	1.09e-05	0.000348%
**OTU_8008**	**True**	**TM7-1**	7.78e-06	0.000709%	0.0	1	0.000534	0.0	0.0	2.04e-05	0.000653%
**OTU_8060**	**True**	**OD1**	7.75e-06	0.000706%	0.0	1	0.000159	1.04e-05	0.0	1.64e-05	0.000525%
**OTU_8221**	**True**	**OD1**	7.7e-06	0.000702%	0.0	1	0.000139	0.0	0.0	1.38e-05	0.000443%
**L209**	**False**	**ABY1**	7.69e-06	0.0007%	0.0	1	0.00136	2.23e-05	2.26e-05	1.03e-05	0.000328%
**OTU_7028**	**True**	**ABY1**	7.67e-06	0.000699%	0.0	1	0.0	0.00168	0.00115	1.77e-05	0.000566%
**L434**	**False**	**WCHB1-64**	7.65e-06	0.000697%	0.0	1	0.000447	0.00014	0.000672	6.62e-05	0.00212%
**L1146**	**False**	**koll11**	7.64e-06	0.000696%	0.0	1	0.00412	0.000314	0.00133	1.57e-05	0.000501%
**OTU_7962**	**True**	**ABY1**	7.61e-06	0.000693%	0.0	1	0.000894	0.00015	0.000274	3.21e-05	0.00103%
**OTU_8318**	**True**	**OD1**	7.61e-06	0.000693%	0.0	1	0.00042	8.91e-05	0.0	2.58e-05	0.000826%
**OTU_7143**	**True**	**OP11-4**	7.61e-06	0.000693%	0.0	1	0.00102	4.99e-05	0.00075	4.63e-05	0.00148%
**L599**	**False**	**PRR-12**	7.6e-06	0.000692%	0.0	1	0.00296	0.000204	9.29e-05	9.5e-06	0.000304%
**L945**	**False**	**koll11**	7.58e-06	0.00069%	0.0	1	0.0044	0.000492	0.00106	7.29e-06	0.000234%
**OTU_82**	**True**	**ZB2**	7.57e-06	0.00069%	0.0	1	0.000212	2.47e-05	0.000208	7.69e-05	0.00246%
**L345**	**False**	**OD1**	7.51e-06	0.000684%	0.0	1	0.00656	0.000821	0.000879	1.05e-05	0.000336%
**L285**	**False**	**ABY1**	7.48e-06	0.000682%	0.0	1	0.000463	0.00405	0.00336	2.12e-05	0.000681%
**OTU_359**	**True**	**koll11**	7.48e-06	0.000681%	0.0	1	0.000653	0.0	0.0	1.4e-05	0.000447%
**OTU_2334**	**True**	**SJA-4**	7.47e-06	0.000681%	0.0	1	3.25e-06	0.00023	0.00101	2.78e-05	0.000889%
**OTU_7978**	**True**	**ABY1**	7.46e-06	0.000679%	0.0	1	0.00107	0.000361	3.07e-05	1.61e-05	0.000515%
**OTU_8141**	**True**	**ABY1**	7.46e-06	0.000679%	0.0	1	2.74e-05	0.00622	0.00317	1.64e-05	0.000524%
**L412**	**False**	**ABY1**	7.44e-06	0.000678%	0.0	1	0.000112	0.000583	0.00196	3.17e-05	0.00102%
**OTU_8120**	**True**	**ABY1**	7.44e-06	0.000678%	0.0	1	1.07e-05	0.00206	0.00362	1.12e-05	0.000357%
**L601**	**False**	**PRR-12**	7.44e-06	0.000678%	0.0	1	0.00193	0.000148	7.07e-05	1.34e-05	0.000431%
**OTU_428**	**True**	**koll11**	7.44e-06	0.000678%	0.0	1	0.000109	6.34e-06	0.000951	2.44e-05	0.000782%
**OTU_250**	**True**	**koll11**	7.43e-06	0.000677%	0.0	1	0.00141	0.000287	0.000315	2.69e-05	0.00086%
**OTU_455**	**True**	**PBS-25**	7.42e-06	0.000676%	0.0	1	0.00194	0.000194	2.25e-06	1.8e-05	0.000577%
**L752**	**False**	**PBS-25**	7.4e-06	0.000674%	0.0	1	0.00467	0.000446	0.00103	1.24e-05	0.000399%
**OTU_6584**	**True**	**OD1**	7.37e-06	0.000671%	0.0	1	0.0	0.000204	0.000877	2.54e-05	0.000812%
**L120**	**False**	**ABY1**	7.37e-06	0.000671%	0.0	1	0.00111	2.24e-05	0.0	1.44e-05	0.00046%
**OTU_604**	**True**	**koll11**	7.33e-06	0.000668%	0.0	1	0.00328	0.000298	6.8e-05	1.25e-05	0.000401%
**L1800**	**False**	**OD1**	7.32e-06	0.000667%	0.0	1	0.000691	2.46e-05	3.82e-05	1.05e-05	0.000338%
**L499**	**False**	**OP11-4**	7.31e-06	0.000666%	0.0	1	0.000679	5.38e-05	0.0	7.98e-06	0.000256%
**L469**	**False**	**OP11-4**	7.29e-06	0.000664%	0.0	1	0.00255	0.000508	0.000127	6.32e-06	0.000202%
**L1029**	**False**	**koll11**	7.27e-06	0.000662%	0.0	1	0.00958	0.000872	0.00314	1.07e-05	0.000343%
**OTU_1019**	**True**	**PBS-25**	7.25e-06	0.000661%	0.0	1	0.000962	1.87e-05	1.33e-05	1.61e-05	0.000514%
**L1107**	**False**	**koll11**	7.24e-06	0.00066%	0.0	1	0.00157	4.05e-05	4.36e-05	7.69e-06	0.000246%
**L1514**	**False**	**OD1**	7.19e-06	0.000655%	0.0	1	0.000774	0.0002	0.0013	4.64e-05	0.00149%
**L1238**	**False**	**TM7**	7.18e-06	0.000654%	0.0	1	0.00506	0.0117	0.00915	0.000125	0.00401%
**OTU_8113**	**True**	**ABY1**	7.16e-06	0.000652%	0.0	1	0.000348	8.28e-05	1.57e-05	2.31e-05	0.000739%
**L654**	**False**	**koll11**	7.16e-06	0.000652%	0.0	1	0.0157	0.00222	0.00438	4.85e-06	0.000155%
**L745**	**False**	**PBS-25**	7.15e-06	0.000652%	0.0	1	0.0068	0.000675	0.00108	9.32e-06	0.000299%
**L1163**	**False**	**koll11**	7.11e-06	0.000648%	0.0	1	0.00167	4.77e-05	3.45e-05	1.45e-05	0.000463%
**L1016**	**False**	**koll11**	7.11e-06	0.000647%	0.0	1	0.00728	0.001	0.000836	6.18e-06	0.000198%
**L363**	**False**	**OD1**	7.1e-06	0.000647%	0.0	1	0.00101	2.09e-05	9.04e-07	9.64e-06	0.000309%
**L1319**	**False**	**OD1**	7.08e-06	0.000645%	0.0	1	0.000385	0.0	1.65e-05	7.87e-06	0.000252%
**OTU_318**	**True**	**koll11**	7.07e-06	0.000644%	0.0	1	0.0029	0.000139	0.000437	1.55e-05	0.000495%
**L1908**	**False**	**OD1**	7.07e-06	0.000644%	0.0	1	0.00077	5.34e-05	7.6e-06	1.02e-05	0.000327%
**OTU_6761**	**True**	**ZB2**	7.02e-06	0.000639%	0.0	1	0.0	2.75e-05	0.000667	1.97e-05	0.000631%
**L470**	**False**	**OP11-4**	7e-06	0.000637%	0.0	1	0.00155	0.000281	7.67e-05	7.78e-06	0.000249%
**L1474**	**False**	**ZB2**	6.9e-06	0.000629%	0.0	1	0.0048	0.00119	0.00114	1.34e-05	0.000429%
**OTU_7298**	**True**	**ABY1**	6.9e-06	0.000628%	0.0	1	0.000134	0.00209	0.00129	1.97e-05	0.000631%
**L1592**	**False**	**OD1**	6.89e-06	0.000628%	0.0	1	0.00194	0.00666	0.00471	6.29e-05	0.00201%
**OTU_8909**	**True**	**ABY1**	6.89e-06	0.000627%	0.0	1	0.000994	1.13e-05	0.0	1.38e-05	0.000441%
**L194**	**False**	**ABY1**	6.88e-06	0.000627%	0.0	1	0.0055	0.000801	0.000814	7.35e-06	0.000235%

**L336**	**False**	**OD1**	6.83e-06	0.000622%	0.0	1	0.000781	7.02e-05	5.76e-05	1.25e-05	0.0004%
**OTU_4142**	**True**	**koll11**	6.8e-06	0.00062%	0.0	1	0.000633	0.0	0.0	1.49e-05	0.000476%
**L1141**	**False**	**koll11**	6.8e-06	0.00062%	0.0	1	0.00907	0.00182	0.000752	1.25e-05	0.000399%
**OTU_6494**	**True**	**ZB2**	6.78e-06	0.000617%	0.0	1	0.0016	0.00022	0.000269	1.54e-05	0.000492%
**L195**	**False**	**ABY1**	6.75e-06	0.000615%	0.0	1	0.00261	0.000704	4.2e-05	7.87e-06	0.000252%
**L301**	**False**	**ABY1**	6.74e-06	0.000614%	0.0	1	0.000767	0.00632	0.00321	2.97e-05	0.000951%
**OTU_8770**	**True**	**OD1**	6.73e-06	0.000613%	0.0	1	0.000182	9.86e-06	0.0	1.47e-05	0.000472%
**OTU_6559**	**True**	**ZB2**	6.69e-06	0.000609%	0.0	1	0.0	5.78e-05	0.00147	1.5e-05	0.000481%
**OTU_170**	**True**	**koll11**	6.68e-06	0.000608%	0.0	1	0.00248	0.00029	0.000143	1.15e-05	0.00037%
**OTU_6309**	**True**	**ZB2**	6.67e-06	0.000608%	0.0	1	0.000692	1.04e-06	4.48e-07	8.1e-06	0.00026%
**L702**	**False**	**koll11**	6.67e-06	0.000607%	0.0	1	0.00139	0.0	0.0	8.79e-06	0.000282%
**OTU_10859**	**True**	**OP11**	6.66e-06	0.000607%	0.0	1	0.000649	1.59e-05	5.3e-07	1.28e-05	0.00041%
**L474**	**False**	**OP11-4**	6.65e-06	0.000606%	0.0	1	0.00141	0.000278	7.06e-05	7.67e-06	0.000246%
**OTU_8327**	**True**	**ZB2**	6.63e-06	0.000604%	0.0	1	0.0	3.3e-05	0.000517	1.75e-05	0.000562%
**OTU_5589**	**True**	**OD1**	6.63e-06	0.000604%	0.0	1	0.000361	1.08e-05	3.42e-05	1.59e-05	0.000509%
**L658**	**False**	**koll11**	6.59e-06	0.000601%	0.0	1	0.0046	0.000298	0.000982	8.9e-06	0.000285%
**L287**	**False**	**ABY1**	6.59e-06	0.0006%	0.0	1	0.000463	0.00302	0.00271	2.6e-05	0.000832%
**L701**	**False**	**koll11**	6.56e-06	0.000598%	0.0	1	0.00159	2.25e-07	0.0	7.32e-06	0.000234%
**OTU_6056**	**True**	**ABY1**	6.55e-06	0.000596%	0.0	1	0.00131	6.96e-05	1.85e-05	6.94e-06	0.000222%
**L109**	**False**	**ABY1**	6.53e-06	0.000595%	0.0	1	0.00422	0.000686	0.000409	5.25e-06	0.000168%
**L1333**	**False**	**ZB2**	6.51e-06	0.000593%	0.0	1	0.00437	0.00104	0.0005	6.89e-06	0.000221%
**L1073**	**False**	**koll11**	6.5e-06	0.000592%	0.0	1	0.00252	0.00016	0.0	7.72e-06	0.000247%
**OTU_1389**	**True**	**GIF10**	6.45e-06	0.000588%	0.0	1	0.00104	0.000172	5.83e-05	1.56e-05	0.000499%
**OTU_60**	**True**	**OD1**	6.44e-06	0.000587%	0.0	1	0.000105	2.36e-05	0.000506	2.94e-05	0.000943%
**L717**	**False**	**PBS-25**	6.35e-06	0.000578%	0.0	1	0.0353	0.00362	0.00449	5.21e-06	0.000167%
**L1292**	**False**	**TM7-3**	6.33e-06	0.000577%	0.0	1	0.00162	5.22e-07	3.05e-05	9.88e-06	0.000316%
**L1887**	**False**	**OD1**	6.3e-06	0.000574%	0.0	1	0.000521	0.000101	2.77e-05	1.69e-05	0.00054%
**OTU_361**	**True**	**koll11**	6.29e-06	0.000573%	0.0	1	0.00123	0.000269	6.62e-05	1.16e-05	0.000371%
**L627**	**False**	**PBS-25**	6.23e-06	0.000567%	0.0	1	0.00438	0.000157	0.000101	6.01e-06	0.000192%
**OTU_7480**	**True**	**ABY1**	6.22e-06	0.000567%	0.0	1	0.0	0.00103	0.000653	1.41e-05	0.000452%
**OTU_6096**	**True**	**ZB2**	6.2e-06	0.000564%	0.0	1	0.00484	0.00128	0.000673	1.58e-05	0.000506%
**OTU_162**	**True**	**OD1**	6.19e-06	0.000564%	0.0	1	0.000106	0.0	0.0	1.56e-05	0.0005%
**L1583**	**False**	**ZB2**	6.12e-06	0.000557%	0.0	1	0.00161	0.00022	0.000499	1.8e-05	0.000577%
**OTU_6221**	**True**	**ZB2**	6.11e-06	0.000557%	0.0	1	0.000893	5.38e-07	0.0	1.16e-05	0.000371%
**OTU_7837**	**True**	**ZB2**	6.08e-06	0.000554%	0.0	1	0.000318	0.0	0.0	1.14e-05	0.000366%
**L330**	**False**	**ABY1**	6.08e-06	0.000554%	0.0	1	0.00111	1.41e-05	3.7e-05	7.33e-06	0.000235%
**L858**	**False**	**OP3**	6.06e-06	0.000552%	0.0	1	0.0187	0.00169	0.00219	3.5e-06	0.000112%
**L1422**	**False**	**ZB2**	6.03e-06	0.000549%	0.0	1	5.35e-05	0.00269	0.00275	1.64e-05	0.000524%
**L1688**	**False**	**OD1**	6.02e-06	0.000548%	0.0	1	0.014	0.00193	0.00132	2.24e-06	7.18e-05%
**OTU_6269**	**True**	**OD1**	6e-06	0.000546%	0.0	1	0.000163	0.0	0.000256	3.68e-05	0.00118%
**L1727**	**False**	**Mb-NB09**	5.99e-06	0.000546%	0.0	1	0.00356	0.000545	0.000774	1.12e-05	0.000359%
**L1046**	**False**	**koll11**	5.98e-06	0.000545%	0.0	1	0.00661	0.00109	0.000718	8.74e-06	0.00028%
**OTU_7948**	**True**	**ABY1**	5.98e-06	0.000545%	0.0	1	0.000427	2.41e-05	3.24e-05	1.5e-05	0.000481%
**L1059**	**False**	**koll11**	5.97e-06	0.000544%	0.0	1	0.000799	9.09e-05	7.3e-05	1.04e-05	0.000333%
**OTU_2333**	**True**	**PBS-25**	5.93e-06	0.00054%	0.0	1	0.00137	3.8e-05	0.00018	1.55e-05	0.000497%
**OTU_10907**	**True**	**OP11-4**	5.93e-06	0.00054%	0.0	1	0.000182	3.44e-06	0.0	1.29e-05	0.000412%
**OTU_4604**	**True**	**ZB2**	5.92e-06	0.00054%	0.0	1	0.000165	0.0	0.0	1.12e-05	0.000358%
**L1554**	**False**	**ZB2**	5.92e-06	0.000539%	0.0	1	0.00504	0.00132	0.000704	1.48e-05	0.000473%
**OTU_166**	**True**	**OP3**	5.91e-06	0.000538%	0.0	1	0.000274	0.0	0.0	1.04e-05	0.000332%
**OTU_10120**	**True**	**OD1**	5.9e-06	0.000538%	0.0	1	0.000403	0.000136	0.0	1.86e-05	0.000594%
**L1157**	**False**	**koll11**	5.9e-06	0.000537%	0.0	1	0.00278	0.000604	0.00211	4.5e-05	0.00144%
**OTU_8867**	**True**	**Mb-NB09**	5.83e-06	0.000531%	0.0	1	0.000846	5.7e-05	0.00068	2.61e-05	0.000837%
**OTU_8043**	**True**	**ABY1**	5.83e-06	0.000531%	0.0	1	0.000466	0.0	0.0	1.01e-05	0.000322%
**OTU_6041**	**True**	**ZB2**	5.79e-06	0.000527%	0.0	1	0.00175	0.000296	0.000935	3.35e-05	0.00107%
**L558**	**False**	**SJA-4**	5.75e-06	0.000524%	0.0	1	0.00217	0.000134	0.000764	2.38e-05	0.000762%
**OTU_240**	**True**	**koll11**	5.74e-06	0.000523%	0.0	1	0.00224	5.27e-05	1.37e-05	1.06e-05	0.00034%
**L305**	**False**	**ABY1**	5.72e-06	0.000521%	0.0	1	0.000418	0.00631	0.00321	1.93e-05	0.000617%
**L824**	**False**	**PBS-25**	5.72e-06	0.000521%	0.0	1	0.0159	0.00134	0.00182	5.53e-06	0.000177%
**L1874**	**False**	**OD1**	5.71e-06	0.00052%	0.0	1	0.00126	0.000384	6.74e-05	1.13e-05	0.000362%
**L1259**	**False**	**TM7-1**	5.63e-06	0.000513%	0.0	1	0.00239	0.000317	0.000368	1.14e-05	0.000367%
**L1208**	**False**	**koll11**	5.63e-06	0.000513%	0.0	1	0.000584	0.0	0.0	1.06e-05	0.000339%
**OTU_5822**	**True**	**OD1**	5.61e-06	0.000511%	0.0	1	0.000235	2.09e-05	0.0	7.98e-06	0.000256%
**OTU_10794**	**True**	**OP11-3**	5.59e-06	0.000509%	0.0	1	0.000173	0.0	0.0	1.19e-05	0.000381%
**L954**	**False**	**koll11**	5.57e-06	0.000507%	0.0	1	0.00158	8.34e-05	8.05e-05	5.18e-06	0.000166%

**OTU_12**	**True**	**ZB2**	5.56e-06	0.000507%	0.0	1	0.000989	0.000174	0.000138	7.5e-06	0.00024%
**L1569**	**False**	**ZB2**	5.52e-06	0.000502%	0.0	1	0.00295	0.00043	0.000749	7.82e-06	0.00025%
**OTU_2350**	**True**	**SJA-4**	5.51e-06	0.000502%	0.0	1	0.000685	0.0	0.0	1.14e-05	0.000364%
**OTU_10803**	**True**	**OP11-4**	5.5e-06	0.000501%	0.0	1	0.000278	5.21e-06	0.0	1.14e-05	0.000364%
**OTU_7000**	**True**	**ZB2**	5.49e-06	0.0005%	0.0	1	1.63e-06	0.000768	0.000341	1.41e-05	0.000452%
**L159**	**False**	**ABY1**	5.49e-06	0.0005%	0.0	1	0.000698	0.00013	3.57e-05	1.77e-05	0.000568%
**OTU_4915**	**True**	**GIF10**	5.49e-06	0.0005%	0.0	1	0.000795	0.000183	7.01e-05	1.57e-05	0.000502%
**L1864**	**False**	**OD1**	5.47e-06	0.000498%	0.0	1	0.0029	0.000578	0.000261	4.92e-06	0.000158%
**OTU_8645**	**True**	**Mb-NB09**	5.46e-06	0.000498%	0.0	1	0.000485	5.35e-05	4.23e-06	7.29e-06	0.000234%
**OTU_6393**	**True**	**ZB2**	5.45e-06	0.000497%	0.0	1	0.000606	0.000101	0.000116	1.64e-05	0.000524%
**OTU_6604**	**True**	**ZB2**	5.44e-06	0.000496%	0.0	1	0.0	7.7e-06	0.000664	1.63e-05	0.000522%
**OTU_5239**	**True**	**OD1**	5.44e-06	0.000495%	0.0	1	0.000726	1.68e-05	7.6e-06	7.41e-06	0.000237%
**OTU_6051**	**True**	**OD1**	5.41e-06	0.000493%	0.0	1	0.000145	0.0	0.0	1.09e-05	0.000349%
**OTU_6230**	**True**	**ZB2**	5.39e-06	0.000491%	0.0	1	0.000163	0.0167	0.00712	1.29e-05	0.000413%
**L956**	**False**	**koll11**	5.36e-06	0.000488%	0.0	1	0.00153	4.84e-05	6.87e-05	4.8e-06	0.000154%
**OTU_348**	**True**	**koll11**	5.36e-06	0.000488%	0.0	1	0.000551	1.71e-05	0.000324	2.69e-05	0.000861%
**OTU_9347**	**True**	**Mb-NB09**	5.29e-06	0.000482%	0.0	1	0.000455	4.16e-05	1.81e-07	9.56e-06	0.000306%
**OTU_7046**	**True**	**ABY1**	5.28e-06	0.000481%	0.0	1	0.00101	7.15e-05	0.0	1.26e-05	0.000405%
**OTU_297**	**True**	**koll11**	5.27e-06	0.00048%	0.0	1	0.00251	0.000453	0.000274	1.01e-05	0.000323%
**OTU_7970**	**True**	**ABY1**	5.25e-06	0.000478%	0.0	1	1.61e-05	0.000838	0.000431	2.31e-05	0.000741%
**L1196**	**False**	**koll11**	5.25e-06	0.000478%	0.0	1	0.00314	0.000325	0.000773	8.11e-06	0.00026%
**OTU_8826**	**True**	**TM7-1**	5.24e-06	0.000477%	0.0	1	0.00053	0.0	0.0	1.07e-05	0.000343%
**OTU_7217**	**True**	**ZB2**	5.23e-06	0.000477%	0.0	1	0.000282	0.0	0.0	9.74e-06	0.000312%
**L398**	**False**	**ABY1**	5.23e-06	0.000476%	0.0	1	0.00117	2.97e-05	0.000159	1e-05	0.000321%
**OTU_7379**	**True**	**ZB2**	5.21e-06	0.000474%	0.0	1	0.000311	0.0	0.0	9.76e-06	0.000313%
**L1757**	**False**	**OD1**	5.2e-06	0.000474%	0.0	1	0.000542	0.0	0.000272	1.66e-05	0.000532%
**L1181**	**False**	**GIF10**	5.2e-06	0.000473%	0.0	1	0.0023	0.000271	0.000279	8.21e-06	0.000263%
**OTU_288**	**True**	**PBS-25**	5.19e-06	0.000473%	0.0	1	0.00213	0.000314	0.000621	1.47e-05	0.00047%
**OTU_6306**	**True**	**ZB2**	5.19e-06	0.000473%	0.0	1	0.00169	0.000102	0.000572	1.81e-05	0.00058%
**L426**	**False**	**OP11**	5.18e-06	0.000472%	0.0	1	0.00131	0.000462	0.00142	4.6e-05	0.00147%
**L407**	**False**	**ABY1**	5.16e-06	0.00047%	0.0	1	0.000323	9.47e-06	1.3e-05	1.07e-05	0.000343%
**L299**	**False**	**ABY1**	5.15e-06	0.000469%	0.0	1	0.000813	0.0113	0.00786	1.05e-05	0.000335%
**OTU_8139**	**True**	**EW055**	5.15e-06	0.000469%	0.0	1	0.00161	5.22e-07	3.05e-05	8.06e-06	0.000258%
**L1438**	**False**	**ZB2**	5.14e-06	0.000468%	0.0	1	0.00203	0.000357	0.000279	1.38e-05	0.000443%
**OTU_8913**	**True**	**Mb-NB09**	5.13e-06	0.000468%	0.0	1	0.000242	0.0	0.0	9.24e-06	0.000296%
**L1730**	**False**	**Mb-NB09**	5.11e-06	0.000466%	0.0	1	0.00274	0.000478	0.000769	1.68e-05	0.00054%
**OTU_6094**	**True**	**ZB2**	5.11e-06	0.000465%	0.0	1	3.06e-05	0.00235	0.00186	1.25e-05	0.000401%
**OTU_8036**	**True**	**TM7-1**	5.1e-06	0.000465%	0.0	1	0.000657	3.61e-05	4e-06	5.81e-06	0.000186%
**OTU_6185**	**True**	**ABY1**	5.1e-06	0.000464%	0.0	1	0.000379	0.000107	1.45e-05	1.85e-05	0.000592%
**L311**	**False**	**ABY1**	5.04e-06	0.000459%	0.0	1	2.73e-05	0.00157	0.0013	9.54e-06	0.000305%
**L1080**	**False**	**koll11**	5.03e-06	0.000458%	0.0	1	0.0036	0.000697	0.000403	8.9e-06	0.000285%
**OTU_8793**	**True**	**OD1**	5.03e-06	0.000458%	0.0	1	3.99e-06	0.000424	0.000597	1.86e-05	0.000596%
**L1631**	**False**	**ZB2**	4.97e-06	0.000453%	0.0	1	0.00345	0.000846	0.00158	2.7e-05	0.000865%
**OTU_1425**	**True**	**OP3**	4.97e-06	0.000453%	0.0	1	0.000395	8.72e-05	0.0	1.12e-05	0.00036%
**L1673**	**False**	**OD1**	4.97e-06	0.000453%	0.0	1	0.00424	0.000457	0.000757	9.2e-06	0.000295%
**L1845**	**False**	**OD1**	4.94e-06	0.00045%	0.0	1	0.00302	0.000504	0.00056	6.14e-06	0.000197%
**L1706**	**False**	**Mb-NB09**	4.92e-06	0.000449%	0.0	1	0.00144	0.00013	4.59e-05	5.76e-06	0.000184%
**OTU_5847**	**True**	**OD1**	4.91e-06	0.000447%	0.0	1	0.00039	1.27e-05	3.69e-05	1.26e-05	0.000402%
**OTU_6123**	**True**	**SM2F11**	4.9e-06	0.000447%	0.0	1	0.000268	0.0	0.0	8.56e-06	0.000274%
**OTU_5575**	**True**	**ZB2**	4.9e-06	0.000446%	0.0	1	0.000139	0.0	7.01e-07	1.03e-05	0.00033%
**OTU_5424**	**True**	**ZB2**	4.88e-06	0.000444%	0.0	1	0.000552	9.6e-06	7.61e-05	9.21e-06	0.000295%
**L132**	**False**	**OD1**	4.87e-06	0.000444%	0.0	1	0.0128	0.0366	0.021	3.21e-05	0.00103%
**L1133**	**False**	**koll11**	4.87e-06	0.000444%	0.0	1	0.0016	2.68e-05	3.97e-05	6.9e-06	0.000221%
**L1351**	**False**	**OD1**	4.86e-06	0.000443%	0.0	1	0.00352	0.00065	0.00154	2.2e-05	0.000706%
**OTU_67**	**True**	**ZB2**	4.86e-06	0.000443%	0.0	1	8.69e-05	0.0154	0.0228	4e-06	0.000128%
**L1895**	**False**	**OD1**	4.86e-06	0.000442%	0.0	1	0.00194	0.000394	0.000148	9.77e-06	0.000313%
**OTU_3671**	**True**	**kpj58rc**	4.85e-06	0.000442%	0.0	1	0.000452	3.54e-05	0.0001	8.44e-06	0.00027%
**OTU_8317**	**True**	**Mb-NB09**	4.85e-06	0.000442%	0.0	1	0.000619	0.000234	4.13e-05	2.04e-05	0.000652%
**OTU_3763**	**True**	**OP3**	4.85e-06	0.000442%	0.0	1	0.000223	0.0	0.0	1.2e-05	0.000384%
**L1565**	**False**	**ZB2**	4.85e-06	0.000442%	0.0	1	0.00523	0.000929	0.000939	5.63e-06	0.00018%
**L207**	**False**	**ABY1**	4.83e-06	0.00044%	0.0	1	0.00167	2.23e-05	2.26e-05	5.21e-06	0.000167%
**OTU_6679**	**True**	**OD1**	4.82e-06	0.000439%	0.0	1	0.00123	0.000242	0.000156	1.99e-05	0.000637%
**L429**	**False**	**OP11**	4.81e-06	0.000438%	0.0	1	0.00098	5.1e-05	0.000189	1.2e-05	0.000385%
**OTU_6095**	**True**	**ZB2**	4.8e-06	0.000437%	0.0	1	0.00167	0.000255	0.000161	1.51e-05	0.000483%

**L565**	**False**	**SJA-4**	4.78e-06	0.000435%	0.0	1	0.00154	4.42e-07	0.000581	1.99e-05	0.000638%
**OTU_6343**	**True**	**ZB2**	4.73e-06	0.000431%	0.0	1	0.00251	0.000905	0.00112	5.55e-05	0.00178%
**OTU_7969**	**True**	**ABY1**	4.72e-06	0.00043%	0.0	1	0.000621	0.000246	1.12e-05	9.73e-06	0.000312%
**L498**	**False**	**OP11-4**	4.71e-06	0.000429%	0.0	1	0.000679	5.38e-05	4.93e-05	7.59e-06	0.000243%
**L631**	**False**	**PBS-25**	4.7e-06	0.000429%	0.0	1	0.00215	8.59e-05	9.04e-05	1.09e-05	0.00035%
**OTU_4064**	**True**	**koll11**	4.7e-06	0.000428%	0.0	1	0.000369	3.86e-05	8.09e-06	1.51e-05	0.000484%
**L253**	**False**	**ABY1**	4.69e-06	0.000427%	0.0	1	0.000695	0.000146	2.46e-05	9.04e-06	0.00029%
**L544**	**False**	**SJA-4**	4.68e-06	0.000427%	0.0	1	0.00108	0.00023	0.00213	3.47e-05	0.00111%
**L1553**	**False**	**ZB2**	4.68e-06	0.000426%	0.0	1	0.00542	0.00132	0.000704	9.67e-06	0.00031%
**OTU_499**	**True**	**PBS-25**	4.67e-06	0.000426%	0.0	1	0.000366	0.0	0.0	5.8e-06	0.000186%
**L1182**	**False**	**GIF10**	4.67e-06	0.000425%	0.0	1	0.00117	8.83e-05	0.000209	1.61e-05	0.000517%
**OTU_436**	**True**	**koll11**	4.67e-06	0.000425%	0.0	1	0.000451	0.0	2.25e-06	9.98e-06	0.00032%
**OTU_165**	**True**	**koll11**	4.66e-06	0.000424%	0.0	1	0.0135	0.000917	0.0021	3.37e-06	0.000108%
**OTU_9264**	**True**	**ABY1**	4.66e-06	0.000424%	0.0	1	0.00029	2.45e-05	3.24e-06	1.18e-05	0.000378%
**L917**	**False**	**OP3**	4.65e-06	0.000424%	0.0	1	0.000619	8.72e-05	0.0	8.45e-06	0.000271%
**L133**	**False**	**ABY1**	4.65e-06	0.000423%	0.0	1	0.00341	0.000376	0.000421	7.52e-06	0.000241%
**L1888**	**False**	**OD1**	4.62e-06	0.000421%	0.0	1	0.000379	7.48e-05	1.05e-05	1.31e-05	0.000419%
**L653**	**False**	**koll11**	4.61e-06	0.00042%	0.0	1	0.0162	0.00231	0.00455	2.96e-06	9.48e-05%
**OTU_4014**	**True**	**GIF10**	4.6e-06	0.000419%	0.0	1	0.0	3.39e-05	0.000469	1.73e-05	0.000554%
**L144**	**False**	**ABY1**	4.6e-06	0.000419%	0.0	1	0.00286	0.00141	0.000297	2.22e-05	0.000711%
**OTU_6328**	**True**	**ZB2**	4.59e-06	0.000418%	0.0	1	0.0	0.00102	0.000785	1.49e-05	0.000477%
**OTU_7670**	**True**	**ABY1**	4.59e-06	0.000418%	0.0	1	0.000398	1.99e-07	1.96e-05	9.08e-06	0.000291%
**OTU_4797**	**True**	**koll11**	4.59e-06	0.000418%	0.0	1	1.89e-06	1.22e-05	0.000394	1.24e-05	0.000397%
**OTU_571**	**True**	**koll11**	4.59e-06	0.000418%	0.0	1	0.000261	0.0	0.0	8.36e-06	0.000268%
**OTU_173**	**True**	**PBS-25**	4.58e-06	0.000418%	0.0	1	0.00118	8.44e-05	0.000843	3.49e-05	0.00112%
**OTU_6544**	**True**	**ZB2**	4.58e-06	0.000418%	0.0	1	0.0	0.0	0.00084	1.28e-05	0.00041%
**L1726**	**False**	**Mb-NB09**	4.58e-06	0.000417%	0.0	1	0.00374	0.000603	0.000939	8.36e-06	0.000268%
**L204**	**False**	**ABY1**	4.55e-06	0.000415%	0.0	1	0.00192	9.63e-05	0.00077	1.17e-05	0.000374%
**L826**	**False**	**PBS-25**	4.55e-06	0.000414%	0.0	1	0.00559	0.000528	0.00053	8.74e-06	0.00028%
**L1900**	**False**	**OD1**	4.53e-06	0.000413%	0.0	1	0.000524	0.000172	7.38e-05	2.25e-05	0.000721%
**OTU_8067**	**True**	**OD1**	4.53e-06	0.000412%	0.0	1	0.000313	8.44e-05	0.0	1.43e-05	0.000459%
**L663**	**False**	**koll11**	4.52e-06	0.000411%	0.0	1	0.00317	0.000101	0.000354	6.9e-06	0.000221%
**L1243**	**False**	**TM7-1**	4.5e-06	0.00041%	0.0	1	0.00413	0.0117	0.00913	4.8e-05	0.00154%
**OTU_753**	**True**	**koll11**	4.5e-06	0.00041%	0.0	1	0.000497	5.38e-06	0.0	9.63e-06	0.000308%
**OTU_6437**	**True**	**ZB2**	4.45e-06	0.000405%	0.0	1	0.000473	1.12e-05	8.97e-06	5.61e-06	0.00018%
**L1192**	**False**	**koll11**	4.41e-06	0.000402%	0.0	1	0.000582	1.44e-05	4.3e-05	7.04e-06	0.000226%
**L1591**	**False**	**OD1**	4.4e-06	0.000401%	0.0	1	0.00194	0.00666	0.00501	3.88e-05	0.00124%
**OTU_6**	**True**	**OD1**	4.37e-06	0.000398%	0.0	1	0.000111	0.0	0.0	1.07e-05	0.000342%
**OTU_6013**	**True**	**ABY1**	4.36e-06	0.000397%	0.0	1	0.000297	0.0	4.68e-06	6.34e-06	0.000203%
**OTU_6055**	**True**	**ABY1**	4.35e-06	0.000396%	0.0	1	0.000264	2.96e-05	0.0	9.07e-06	0.000291%
**OTU_4583**	**True**	**ZB2**	4.34e-06	0.000395%	0.0	1	0.0011	0.000106	0.000112	1.03e-05	0.000331%
**L1484**	**False**	**ZB2**	4.34e-06	0.000395%	0.0	1	0.00401	0.000561	0.000731	6.88e-06	0.00022%
**OTU_6572**	**True**	**ZB2**	4.33e-06	0.000395%	0.0	1	0.00172	0.000189	5.1e-05	6.57e-06	0.000211%
**OTU_495**	**True**	**koll11**	4.33e-06	0.000395%	0.0	1	0.000899	1.89e-05	0.0	7.29e-06	0.000234%
**L477**	**False**	**OP11-4**	4.33e-06	0.000395%	0.0	1	0.000998	0.000227	5.01e-05	7.22e-06	0.000231%
**L770**	**False**	**PBS-25**	4.32e-06	0.000393%	0.0	1	0.00411	0.00031	0.000443	8.49e-06	0.000272%
**L636**	**False**	**PBS-25**	4.31e-06	0.000393%	0.0	1	0.00106	3.56e-05	1.06e-05	6.21e-06	0.000199%
**OTU_8782**	**True**	**ABY1**	4.3e-06	0.000392%	0.0	1	0.00242	9.51e-05	0.0	8.95e-06	0.000287%
**L1866**	**False**	**OD1**	4.29e-06	0.00039%	0.0	1	0.00149	0.000146	0.000181	6.69e-06	0.000214%
**L15**	**False**	**ABY1**	4.27e-06	0.000389%	0.0	1	0.00146	5.3e-05	0.0	6.49e-06	0.000208%
**OTU_861**	**True**	**PBS-25**	4.26e-06	0.000388%	0.0	1	0.000814	0.0	0.0	1.24e-05	0.000396%
**L848**	**False**	**koll11**	4.24e-06	0.000387%	0.0	1	0.00697	0.000533	0.0015	1e-05	0.000321%
**L377**	**False**	**ABY1**	4.24e-06	0.000386%	0.0	1	0.000378	0.0	7.42e-08	5.99e-06	0.000192%
**OTU_10862**	**True**	**OP11-4**	4.21e-06	0.000384%	0.0	1	0.000846	0.000227	5.01e-05	8.99e-06	0.000288%
**OTU_8108**	**True**	**ABY1**	4.2e-06	0.000382%	0.0	1	0.000234	1.43e-05	1.81e-07	7.35e-06	0.000235%
**OTU_8163**	**True**	**ABY1**	4.19e-06	0.000382%	0.0	1	0.000231	4.54e-07	0.0	7.94e-06	0.000254%
**L1001**	**False**	**koll11**	4.19e-06	0.000382%	0.0	1	0.00191	0.000373	0.00102	2.43e-05	0.000778%
**L1062**	**False**	**koll11**	4.18e-06	0.000381%	0.0	1	0.00223	0.000307	0.000678	1.44e-05	0.000462%
**L1147**	**False**	**koll11**	4.18e-06	0.000381%	0.0	1	0.00329	0.000274	0.00115	1.08e-05	0.000345%
**L506**	**False**	**OP11-4**	4.17e-06	0.00038%	0.0	1	0.00397	0.000523	0.000706	5.66e-06	0.000181%
**L554**	**False**	**SJA-4**	4.17e-06	0.00038%	0.0	1	0.000694	6.99e-05	5.85e-05	1.42e-05	0.000456%
**L1833**	**False**	**OD1**	4.16e-06	0.000379%	0.0	1	8.42e-05	3.91e-06	0.000159	2.41e-05	0.000771%
**L1019**	**False**	**koll11**	4.13e-06	0.000376%	0.0	1	0.00348	0.000616	0.000482	4.67e-06	0.00015%
**OTU_8815**	**True**	**ABY1**	4.11e-06	0.000375%	0.0	1	0.000404	1.26e-05	0.0	1.2e-05	0.000386%

**OTU_7853**	**True**	**ABY1**	4.11e-06	0.000375%	0.0	1	0.000816	2.24e-05	1.94e-05	8.3e-06	0.000266%
**L1151**	**False**	**koll11**	4.09e-06	0.000372%	0.0	1	0.00218	0.000351	0.000165	8.93e-06	0.000286%
**OTU_8527**	**True**	**ABY1**	4.08e-06	0.000371%	0.0	1	5.04e-06	0.000159	0.000401	1.51e-05	0.000483%
**L1293**	**False**	**TM7-3**	4.06e-06	0.00037%	0.0	1	0.00162	5.22e-07	3.05e-05	6.33e-06	0.000203%
**OTU_4028**	**True**	**koll11**	4.06e-06	0.00037%	0.0	1	0.000761	0.0	0.0	6.59e-06	0.000211%
**OTU_8299**	**True**	**ABY1**	4.06e-06	0.00037%	0.0	1	1.66e-05	0.00157	0.0013	7.28e-06	0.000233%
**OTU_335**	**True**	**koll11**	4.05e-06	0.000369%	0.0	1	0.00308	0.000402	0.000138	7.47e-06	0.000239%
**OTU_2521**	**True**	**koll11**	4.04e-06	0.000368%	0.0	1	0.000283	1.8e-06	3.57e-06	9.56e-06	0.000306%
**OTU_1088**	**True**	**koll11**	4.03e-06	0.000368%	0.0	1	0.000632	0.0	3.57e-06	7.53e-06	0.000241%
**L1540**	**False**	**ZB2**	4.03e-06	0.000367%	0.0	1	0.000439	0.0	0.0	6.24e-06	0.0002%
**OTU_7961**	**True**	**ZB2**	4.03e-06	0.000367%	0.0	1	0.000279	0.000183	1.08e-05	1.48e-05	0.000475%
**OTU_1247**	**True**	**PRR-12**	4.03e-06	0.000367%	0.0	1	0.00103	5.69e-05	2.22e-05	8.27e-06	0.000265%
**L718**	**False**	**PBS-25**	4.03e-06	0.000367%	0.0	1	0.00295	0.000418	0.000744	8.35e-06	0.000267%
**OTU_1590**	**True**	**PBS-25**	4.02e-06	0.000366%	0.0	1	0.000377	1.25e-05	0.0	8.05e-06	0.000258%
**L433**	**False**	**OP11**	4.01e-06	0.000365%	0.0	1	0.00138	0.000161	0.000672	1.5e-05	0.000481%
**L1263**	**False**	**TM7-1**	3.97e-06	0.000362%	0.0	1	0.00102	0.0	0.0	5.52e-06	0.000177%
**L198**	**False**	**ABY1**	3.96e-06	0.000361%	0.0	1	0.00131	0.000342	1.12e-05	6.42e-06	0.000206%
**OTU_5893**	**True**	**OD1**	3.93e-06	0.000358%	0.0	1	0.000167	0.0	0.0	8.35e-06	0.000267%
**OTU_4041**	**True**	**koll11**	3.93e-06	0.000358%	0.0	1	0.000553	0.0	0.0	8.03e-06	0.000257%
**OTU_9156**	**True**	**Mb-NB09**	3.92e-06	0.000357%	0.0	1	0.000251	0.0	0.0	8.38e-06	0.000268%
**OTU_7122**	**True**	**ZB2**	3.92e-06	0.000357%	0.0	1	0.000288	1.89e-05	2e-05	1.12e-05	0.000359%
**OTU_672**	**True**	**koll11**	3.89e-06	0.000355%	0.0	1	0.000828	4.04e-05	0.00019	1.14e-05	0.000365%
**L1261**	**False**	**TM7-1**	3.89e-06	0.000354%	0.0	1	0.00239	0.000263	7.09e-05	4.9e-06	0.000157%
**L1598**	**False**	**OD1**	3.87e-06	0.000353%	0.0	1	5.78e-05	0.00627	0.00345	6.64e-06	0.000213%
**OTU_8744**	**True**	**TM7-1**	3.85e-06	0.000351%	0.0	1	0.000134	0.0	0.0	9.33e-06	0.000299%
**L1091**	**False**	**koll11**	3.84e-06	0.00035%	0.0	1	0.0117	0.00106	0.00237	3.98e-06	0.000128%
**L748**	**False**	**PBS-25**	3.84e-06	0.00035%	0.0	1	0.00526	0.000519	0.00103	5.86e-06	0.000188%
**OTU_7233**	**True**	**ABY1**	3.84e-06	0.00035%	0.0	1	0.00367	4.32e-05	0.000258	5.41e-06	0.000173%
**OTU_10795**	**True**	**OP11-3**	3.84e-06	0.00035%	0.0	1	7.49e-05	0.0	0.0	1.01e-05	0.000323%
**OTU_6709**	**True**	**OD1**	3.83e-06	0.000349%	0.0	1	0.000225	0.0	0.0	5.23e-06	0.000168%
**OTU_6089**	**True**	**SM2F11**	3.82e-06	0.000348%	0.0	1	0.000295	0.0	0.0	8.99e-06	0.000288%
**OTU_8039**	**True**	**ABY1**	3.82e-06	0.000348%	0.0	1	0.000448	0.0	0.0	6.99e-06	0.000224%
**OTU_140**	**True**	**OD1**	3.82e-06	0.000348%	0.0	1	4.46e-05	4.37e-05	0.00037	2.41e-05	0.000771%
**L1666**	**False**	**OD1**	3.82e-06	0.000348%	0.0	1	0.00339	0.000892	0.000712	9.63e-06	0.000308%
**L1239**	**False**	**TM7**	3.82e-06	0.000348%	0.0	1	0.00467	0.0117	0.00913	5.45e-05	0.00174%
**L943**	**False**	**koll11**	3.82e-06	0.000348%	0.0	1	0.0102	0.00101	0.00142	2.13e-06	6.82e-05%
**L118**	**False**	**OD1**	3.81e-06	0.000347%	0.0	1	0.0156	0.0477	0.029	1.53e-05	0.000491%
**OTU_8224**	**True**	**ABY1**	3.81e-06	0.000347%	0.0	1	0.000114	0.0	0.0	6.79e-06	0.000218%
**L206**	**False**	**ABY1**	3.81e-06	0.000347%	0.0	1	0.00192	5.4e-05	2.26e-05	4.27e-06	0.000137%
**OTU_449**	**True**	**koll11**	3.79e-06	0.000345%	0.0	1	0.000405	3.96e-05	0.000321	2.3e-05	0.000736%
**OTU_5236**	**True**	**OD1**	3.78e-06	0.000345%	0.0	1	0.000517	1.48e-05	3.57e-06	4.33e-06	0.000139%
**OTU_315**	**True**	**PBS-25**	3.78e-06	0.000344%	0.0	1	0.00479	0.000534	9.75e-05	3.93e-06	0.000126%
**OTU_57**	**True**	**Mb-NB09**	3.77e-06	0.000343%	0.0	1	5.86e-05	6.69e-06	0.0	1.31e-05	0.00042%
**OTU_343**	**True**	**koll11**	3.77e-06	0.000343%	0.0	1	0.000861	7.32e-05	0.000402	1.71e-05	0.000547%
**OTU_3125**	**True**	**koll11**	3.76e-06	0.000343%	0.0	1	0.000412	2.04e-05	2e-05	9.48e-06	0.000304%
**L1748**	**False**	**OD1**	3.75e-06	0.000342%	0.0	1	0.00156	0.000795	0.00234	4.69e-05	0.0015%
**OTU_837**	**True**	**koll11**	3.73e-06	0.00034%	0.0	1	0.000918	0.000248	0.000109	1.89e-05	0.000607%
**L634**	**False**	**PBS-25**	3.73e-06	0.00034%	0.0	1	0.00112	4.56e-05	1.06e-05	5.4e-06	0.000173%
**OTU_7973**	**True**	**ABY1**	3.72e-06	0.000339%	0.0	1	0.0	0.000812	0.000582	1.58e-05	0.000505%
**L1819**	**False**	**OD1**	3.72e-06	0.000339%	0.0	1	0.00131	5.83e-05	3.66e-05	6.2e-06	0.000199%
**L1241**	**False**	**TM7**	3.71e-06	0.000338%	0.0	1	0.00414	0.0117	0.00913	3.96e-05	0.00127%
**L102**	**False**	**ABY1**	3.69e-06	0.000337%	0.0	1	0.00153	5.83e-05	1.55e-05	4.89e-06	0.000157%
**OTU_9026**	**True**	**ABY1**	3.67e-06	0.000335%	0.0	1	0.000478	4.8e-05	0.00012	8.4e-06	0.000269%
**L1667**	**False**	**OD1**	3.67e-06	0.000335%	0.0	1	0.000734	0.000184	0.0	6.42e-06	0.000206%
**L1521**	**False**	**ZB2**	3.67e-06	0.000334%	0.0	1	0.00237	0.000274	0.000102	3.64e-06	0.000117%
**L958**	**False**	**koll11**	3.67e-06	0.000334%	0.0	1	0.000845	2.78e-05	6.27e-05	6.29e-06	0.000201%
**OTU_8643**	**True**	**ABY1**	3.65e-06	0.000332%	0.0	1	0.000349	6.74e-06	7e-06	6.42e-06	0.000206%
**L196**	**False**	**ABY1**	3.64e-06	0.000332%	0.0	1	0.00154	0.000343	1.12e-05	5.64e-06	0.000181%
**OTU_8146**	**True**	**ABY1**	3.64e-06	0.000331%	0.0	1	0.000186	0.0	0.0	7.63e-06	0.000245%
**OTU_6315**	**True**	**K2-4-19**	3.63e-06	0.00033%	0.0	1	0.00023	0.0	0.0	6.8e-06	0.000218%
**L460**	**False**	**OP11-4**	3.61e-06	0.000329%	0.0	1	0.000804	0.00012	0.000108	8.02e-06	0.000257%
**OTU_8122**	**True**	**ABY1**	3.61e-06	0.000329%	0.0	1	0.000251	1.57e-05	0.0	7.38e-06	0.000237%
**L86**	**False**	**ABY1**	3.6e-06	0.000328%	0.0	1	0.00111	7.15e-05	0.0	7.32e-06	0.000234%
**L500**	**False**	**OP11-4**	3.58e-06	0.000326%	0.0	1	0.000391	5.21e-06	0.0	5.36e-06	0.000172%
**OTU_310**	**True**	**koll11**	3.58e-06	0.000326%	0.0	1	0.000891	2.68e-05	3.97e-05	9.08e-06	0.000291%

**L353**	**False**	**OD1**	3.58e-06	0.000326%	0.0	1	0.000431	9.16e-05	1.53e-05	1.15e-05	0.000369%
**OTU_1020**	**True**	**PBS-25**	3.57e-06	0.000325%	0.0	1	0.000454	0.0	3.57e-05	8.42e-06	0.00027%
**OTU_6634**	**True**	**K2-4-19**	3.56e-06	0.000325%	0.0	1	0.000295	7.75e-06	0.0	4.17e-06	0.000133%
**OTU_6567**	**True**	**ZB2**	3.56e-06	0.000324%	0.0	1	0.00175	0.000246	0.000489	1.42e-05	0.000453%
**L1144**	**False**	**koll11**	3.54e-06	0.000323%	0.0	1	0.00412	0.000451	0.00133	8.21e-06	0.000263%
**OTU_6088**	**True**	**ABY1**	3.54e-06	0.000323%	0.0	1	0.000626	9.19e-05	3.87e-05	1.13e-05	0.000362%
**L252**	**False**	**ABY1**	3.54e-06	0.000323%	0.0	1	0.000695	0.000146	4.93e-05	7.85e-06	0.000251%
**L1504**	**False**	**ZB2**	3.53e-06	0.000322%	0.0	1	0.000213	9.09e-07	0.0	9.46e-06	0.000303%
**L242**	**False**	**ABY1**	3.53e-06	0.000321%	0.0	1	0.00759	0.00142	0.00124	6.24e-06	0.0002%
**L463**	**False**	**OP11-4**	3.53e-06	0.000321%	0.0	1	0.000358	0.000103	5.43e-06	1.11e-05	0.000355%
**L1219**	**False**	**koll11**	3.51e-06	0.00032%	0.0	1	0.000181	0.0	0.0	8.07e-06	0.000258%
**OTU_5443**	**True**	**ZB2**	3.5e-06	0.000319%	0.0	1	0.000774	0.000385	0.000185	2.22e-05	0.000713%
**L1186**	**False**	**GIF10**	3.49e-06	0.000318%	0.0	1	0.000343	0.0	0.0	5.01e-06	0.000161%
**OTU_6922**	**True**	**OD1**	3.48e-06	0.000317%	0.0	1	0.000272	0.0	3.24e-05	6.97e-06	0.000223%
**OTU_1101**	**True**	**koll11**	3.47e-06	0.000316%	0.0	1	0.00157	0.000157	0.000413	1.29e-05	0.000412%
**L1763**	**False**	**OD1**	3.45e-06	0.000315%	0.0	1	0.000313	4.37e-05	0.000379	3.33e-05	0.00107%
**L935**	**False**	**koll11**	3.45e-06	0.000314%	0.0	1	0.00101	2.41e-05	0.0	5.07e-06	0.000162%
**L1529**	**False**	**ZB2**	3.45e-06	0.000314%	0.0	1	0.000972	0.000437	0.000194	1.59e-05	0.000509%
**L1703**	**False**	**OD1**	3.45e-06	0.000314%	0.0	1	0.00104	5.9e-05	0.0	5.65e-06	0.000181%
**OTU_29**	**True**	**ZB2**	3.42e-06	0.000311%	0.0	1	0.00176	0.000247	0.000588	8.72e-06	0.000279%
**OTU_5495**	**True**	**ZB2**	3.42e-06	0.000311%	0.0	1	0.000687	6.32e-05	9.89e-06	7.17e-06	0.00023%
**OTU_9271**	**True**	**ABY1**	3.41e-06	0.000311%	0.0	1	0.000126	0.0	0.0	6.97e-06	0.000223%
**OTU_907**	**True**	**koll11**	3.41e-06	0.00031%	0.0	1	0.00369	0.000222	0.000955	5.71e-06	0.000183%
**L1564**	**False**	**ZB2**	3.4e-06	0.00031%	0.0	1	0.00542	0.000929	0.000943	3.71e-06	0.000119%
**L586**	**False**	**PRR-12**	3.39e-06	0.000309%	0.0	1	0.00217	7.07e-05	1.6e-05	3.6e-06	0.000115%
**OTU_7009**	**True**	**ZB2**	3.39e-06	0.000308%	0.0	1	0.000419	0.0	0.0	6.9e-06	0.000221%
**OTU_371**	**True**	**koll11**	3.39e-06	0.000308%	0.0	1	0.000655	0.000122	0.000339	2.06e-05	0.00066%
**OTU_451**	**True**	**PRR-10**	3.38e-06	0.000308%	0.0	1	0.00962	0.00117	0.000595	3.9e-06	0.000125%
**L1628**	**False**	**ZB2**	3.36e-06	0.000306%	0.0	1	0.00478	0.000863	0.00177	1.01e-05	0.000325%
**L1623**	**False**	**OD1**	3.36e-06	0.000306%	0.0	1	0.000303	3.78e-05	6.17e-05	1.14e-05	0.000365%
**OTU_608**	**True**	**koll11**	3.35e-06	0.000306%	0.0	1	0.00377	0.000309	0.00101	5.81e-06	0.000186%
**OTU_9291**	**True**	**ABY1**	3.35e-06	0.000305%	0.0	1	0.000277	2.57e-05	0.0	9.03e-06	0.000289%
**L886**	**False**	**koll11**	3.33e-06	0.000304%	0.0	1	0.00168	0.000428	0.000324	9.71e-06	0.000311%
**L1200**	**False**	**koll11**	3.33e-06	0.000303%	0.0	1	0.00173	8.37e-05	0.000131	4.04e-06	0.000129%
**OTU_437**	**True**	**PBS-25**	3.32e-06	0.000303%	0.0	1	0.000361	0.0	1.78e-05	7.08e-06	0.000227%
**OTU_3064**	**True**	**koll11**	3.32e-06	0.000302%	0.0	1	0.000734	7.07e-05	4.24e-05	5.82e-06	0.000187%
**OTU_8664**	**True**	**OD1**	3.29e-06	0.0003%	0.0	1	0.000117	1.34e-06	0.0	8.92e-06	0.000286%
**L339**	**False**	**OD1**	3.28e-06	0.000299%	0.0	1	0.000787	5.76e-05	1.15e-05	7.3e-06	0.000234%
**OTU_5449**	**True**	**OD1**	3.28e-06	0.000299%	0.0	1	0.000764	0.000117	4.34e-05	6.19e-06	0.000198%
**L1201**	**False**	**koll11**	3.27e-06	0.000298%	0.0	1	0.00132	6.33e-05	0.000111	4.76e-06	0.000152%
**L1525**	**False**	**ZB2**	3.26e-06	0.000297%	0.0	1	0.000509	8.49e-05	5.06e-05	7.82e-06	0.000251%
**L1670**	**False**	**OD1**	3.25e-06	0.000296%	0.0	1	0.00266	0.000708	0.000712	1.44e-05	0.000461%
**OTU_4757**	**True**	**ZB2**	3.25e-06	0.000296%	0.0	1	0.000397	0.000348	2.52e-05	2.23e-05	0.000714%
**L550**	**False**	**SJA-4**	3.24e-06	0.000295%	0.0	1	0.000643	0.0	0.000153	1.22e-05	0.00039%
**OTU_8832**	**True**	**Mb-NB09**	3.23e-06	0.000295%	0.0	1	0.00056	8.14e-06	2.3e-05	8.24e-06	0.000264%
**OTU_5902**	**True**	**OD1**	3.23e-06	0.000294%	0.0	1	0.000443	2.25e-07	6.97e-06	4.65e-06	0.000149%
**OTU_9077**	**True**	**OD1**	3.23e-06	0.000294%	0.0	1	0.000404	0.0	3.57e-06	9.05e-06	0.00029%
**L178**	**False**	**SM2F11**	3.23e-06	0.000294%	0.0	1	0.000582	8.3e-05	0.000236	1.16e-05	0.000371%
**OTU_996**	**True**	**PBS-25**	3.23e-06	0.000294%	0.0	1	0.000643	2.03e-05	0.0	6.34e-06	0.000203%
**OTU_7174**	**True**	**OD1**	3.21e-06	0.000293%	0.0	1	0.00226	0.000219	0.000371	4.18e-06	0.000134%
**OTU_7138**	**True**	**ABY1**	3.21e-06	0.000292%	0.0	1	6.69e-05	1.34e-05	0.000995	9.78e-06	0.000313%
**L1762**	**False**	**OD1**	3.2e-06	0.000292%	0.0	1	0.000419	4.37e-05	0.000379	2.57e-05	0.000823%
**OTU_2347**	**True**	**SJA-4**	3.2e-06	0.000292%	0.0	1	0.000694	6.99e-05	5.71e-05	1.09e-05	0.000349%
**L1084**	**False**	**koll11**	3.2e-06	0.000291%	0.0	1	0.00318	0.000635	0.000361	5.79e-06	0.000185%
**OTU_268**	**True**	**koll11**	3.2e-06	0.000291%	0.0	1	0.000873	0.000191	4.62e-05	7.29e-06	0.000233%
**OTU_395**	**True**	**koll11**	3.18e-06	0.00029%	0.0	1	0.00109	3.68e-05	8.83e-05	4.6e-06	0.000147%
**OTU_552**	**True**	**koll11**	3.18e-06	0.00029%	0.0	1	0.00063	8.13e-05	2.05e-05	1.18e-05	0.000377%
**OTU_600**	**True**	**PBS-25**	3.18e-06	0.00029%	0.0	1	0.000521	4.1e-06	3.14e-05	7.44e-06	0.000238%
**OTU_430**	**True**	**PBS-25**	3.17e-06	0.000289%	0.0	1	0.00307	0.000298	0.000941	8.41e-06	0.000269%
**OTU_35**	**True**	**GIF10**	3.15e-06	0.000287%	0.0	1	0.000849	0.0	1.48e-07	5.04e-06	0.000161%
**L1272**	**False**	**TM7**	3.14e-06	0.000286%	0.0	1	0.000385	6.43e-07	2.07e-05	5.62e-06	0.00018%
**OTU_6662**	**True**	**ZB2**	3.14e-06	0.000286%	0.0	1	7.37e-07	0.000503	0.00132	1.01e-05	0.000325%
**OTU_833**	**True**	**koll11**	3.13e-06	0.000285%	0.0	1	0.00117	1.09e-05	1.87e-05	3.29e-06	0.000105%
**L1134**	**False**	**koll11**	3.12e-06	0.000285%	0.0	1	0.00115	2.68e-05	3.97e-05	5.94e-06	0.00019%
**OTU_9016**	**True**	**ABY1**	3.11e-06	0.000284%	0.0	1	0.00024	1.93e-05	7.42e-08	6.52e-06	0.000209%

**L1354**	**False**	**ZB2**	3.11e-06	0.000283%	0.0	1	0.00323	0.00065	0.00118	1.34e-05	0.00043%
**OTU_1263**	**True**	**PBS-25**	3.1e-06	0.000283%	0.0	1	0.000452	0.0	0.0	6.64e-06	0.000213%
**OTU_6568**	**True**	**ZB2**	3.1e-06	0.000282%	0.0	1	0.000243	0.0	0.0	5.72e-06	0.000183%
**OTU_2207**	**True**	**PBS-25**	3.08e-06	0.000281%	0.0	1	0.00184	0.000117	0.000154	1.08e-05	0.000345%
**OTU_7944**	**True**	**ABY1**	3.07e-06	0.00028%	0.0	1	0.000502	0.0	3.1e-05	7.12e-06	0.000228%
**OTU_7945**	**True**	**ABY1**	3.06e-06	0.000279%	0.0	1	0.000427	1.04e-05	2.25e-05	6.66e-06	0.000213%
**L1896**	**False**	**OD1**	3.05e-06	0.000278%	0.0	1	0.00106	0.000176	3.49e-05	5.35e-06	0.000171%
**OTU_5654**	**True**	**OD1**	3.05e-06	0.000278%	0.0	1	0.000119	0.0	0.0	6.55e-06	0.00021%
**OTU_7327**	**True**	**ABY1**	3.05e-06	0.000278%	0.0	1	4.73e-07	0.000657	9.38e-05	5.46e-06	0.000175%
**L1516**	**False**	**OD1**	3.04e-06	0.000277%	0.0	1	0.000618	0.000173	0.00129	1.86e-05	0.000596%
**L1612**	**False**	**ZB2**	3.03e-06	0.000276%	0.0	1	0.00142	0.000106	3.2e-05	4.07e-06	0.00013%
**OTU_8249**	**True**	**ABY1**	3.03e-06	0.000276%	0.0	1	0.00197	0.000404	0.000239	1.03e-05	0.000329%
**OTU_299**	**True**	**PBS-25**	3.03e-06	0.000276%	0.0	1	0.00139	0.000268	0.000286	8.22e-06	0.000263%
**OTU_9728**	**True**	**ZB2**	3.02e-06	0.000275%	0.0	1	0.000159	1.1e-05	0.0	8.02e-06	0.000257%
**L657**	**False**	**koll11**	3.02e-06	0.000275%	0.0	1	0.00496	0.000334	0.00101	3.95e-06	0.000127%
**OTU_7005**	**True**	**ZB2**	3.01e-06	0.000274%	0.0	1	0.000312	0.0	0.0	6.51e-06	0.000208%
**L1077**	**False**	**koll11**	2.99e-06	0.000272%	0.0	1	0.00208	0.000143	0.0	4.86e-06	0.000156%
**OTU_2705**	**True**	**SJA-4**	2.99e-06	0.000272%	0.0	1	0.000173	0.0	2.15e-05	8.17e-06	0.000262%
**OTU_5493**	**True**	**ZB2**	2.99e-06	0.000272%	0.0	1	0.00066	0.0	0.0	3.64e-06	0.000116%
**OTU_7212**	**True**	**ZB2**	2.98e-06	0.000272%	0.0	1	0.000173	5.08e-06	2.99e-07	6.76e-06	0.000217%
**OTU_5588**	**True**	**OD1**	2.96e-06	0.00027%	0.0	1	0.000722	1.68e-05	1.1e-05	5.31e-06	0.00017%
**L1517**	**False**	**OD1**	2.95e-06	0.000269%	0.0	1	0.000618	0.000173	0.00129	1.81e-05	0.00058%
**L1643**	**False**	**ZB2**	2.94e-06	0.000267%	0.0	1	0.00117	0.000307	0.00104	2.17e-05	0.000696%
**L1858**	**False**	**OD1**	2.94e-06	0.000267%	0.0	1	0.000302	1.04e-06	1.96e-05	5.18e-06	0.000166%
**OTU_5240**	**True**	**ZB2**	2.93e-06	0.000267%	0.0	1	0.000409	0.000141	9.16e-06	9.19e-06	0.000295%
**OTU_8868**	**True**	**ABY1**	2.92e-06	0.000266%	0.0	1	0.000152	2.15e-05	8.39e-07	7.03e-06	0.000225%
**OTU_7383**	**True**	**ABY1**	2.92e-06	0.000266%	0.0	1	0.000279	2.42e-05	0.0	6.79e-06	0.000217%
**OTU_7983**	**True**	**ABY1**	2.91e-06	0.000265%	0.0	1	0.0	0.000454	0.000443	1.44e-05	0.00046%
**L1695**	**False**	**Mb-NB09**	2.9e-06	0.000265%	0.0	1	0.00111	0.000293	5.92e-05	5.12e-06	0.000164%
**OTU_4894**	**True**	**GIF10**	2.9e-06	0.000265%	0.0	1	0.000222	0.0	0.0	6.19e-06	0.000198%
**L238**	**False**	**ABY1**	2.89e-06	0.000263%	0.0	1	0.000864	2.25e-06	2.87e-05	5.59e-06	0.000179%
**OTU_2634**	**True**	**PBS-25**	2.88e-06	0.000263%	0.0	1	0.00332	0.000468	0.000545	7.2e-06	0.000231%
**L662**	**False**	**koll11**	2.88e-06	0.000262%	0.0	1	0.00341	0.000153	0.000515	4.2e-06	0.000135%
**OTU_6057**	**True**	**ABY1**	2.87e-06	0.000262%	0.0	1	0.000377	8.8e-05	0.0	1.34e-05	0.000428%
**OTU_7975**	**True**	**ABY1**	2.87e-06	0.000262%	0.0	1	0.00109	0.000246	7.16e-06	6.72e-06	0.000215%
**OTU_463**	**True**	**koll11**	2.87e-06	0.000261%	0.0	1	0.00044	3.85e-05	3.45e-05	7.06e-06	0.000226%
**OTU_10779**	**True**	**OP11-4**	2.86e-06	0.000261%	0.0	1	0.000922	0.00016	5.12e-05	4.66e-06	0.000149%
**OTU_5515**	**True**	**ZB2**	2.86e-06	0.00026%	0.0	1	0.000356	2.04e-06	7.01e-07	6.35e-06	0.000203%
**L878**	**False**	**koll11**	2.85e-06	0.00026%	0.0	1	0.00774	0.00156	0.00297	4.29e-06	0.000137%
**OTU_6469**	**True**	**OD1**	2.85e-06	0.000259%	0.0	1	0.00312	0.000256	0.000324	5.69e-06	0.000182%
**L1850**	**False**	**OD1**	2.82e-06	0.000257%	0.0	1	0.000309	9.86e-06	8.21e-05	1.08e-05	0.000346%
**OTU_6591**	**True**	**ZB2**	2.81e-06	0.000256%	0.0	1	0.000333	0.0	0.0	5.74e-06	0.000184%
**OTU_4491**	**True**	**koll11**	2.81e-06	0.000256%	0.0	1	0.000263	0.0	0.0	6e-06	0.000192%
**L911**	**False**	**koll11**	2.8e-06	0.000255%	0.0	1	0.00101	0.000157	0.000352	1.03e-05	0.000331%
**L1100**	**False**	**koll11**	2.79e-06	0.000254%	0.0	1	0.00171	0.000125	0.000683	9.67e-06	0.00031%
**OTU_5931**	**True**	**OD1**	2.78e-06	0.000254%	0.0	1	0.000912	0.000146	8.84e-05	7.19e-06	0.00023%
**L1803**	**False**	**OD1**	2.78e-06	0.000253%	0.0	1	0.000716	0.0	0.0	3.67e-06	0.000117%
**L1339**	**False**	**ZB2**	2.78e-06	0.000253%	0.0	1	0.00161	0.000595	0.000221	8.14e-06	0.000261%
**L734**	**False**	**PBS-25**	2.78e-06	0.000253%	0.0	1	0.00618	0.000802	0.000384	2.67e-06	8.55e-05%
**OTU_7843**	**True**	**ABY1**	2.75e-06	0.00025%	0.0	1	0.00324	0.00136	0.000196	9.76e-06	0.000313%
**OTU_6966**	**True**	**OD1**	2.74e-06	0.00025%	0.0	1	0.000273	0.0	0.0	5.56e-06	0.000178%
**OTU_8786**	**True**	**ABY1**	2.74e-06	0.000249%	0.0	1	7.36e-05	0.000316	5.86e-06	1.4e-05	0.000449%
**OTU_52**	**True**	**ZB2**	2.72e-06	0.000248%	0.0	1	0.000167	9.04e-06	0.0	6.61e-06	0.000212%
**L754**	**False**	**PBS-25**	2.72e-06	0.000247%	0.0	1	0.00161	0.000149	9.12e-05	4.77e-06	0.000153%
**L1773**	**False**	**OD1**	2.71e-06	0.000247%	0.0	1	0.000668	0.000112	7.25e-05	6.48e-06	0.000207%
**OTU_6779**	**True**	**ZB2**	2.7e-06	0.000246%	0.0	1	4.67e-06	0.0	0.00023	6.66e-06	0.000213%
**OTU_5995**	**True**	**SM2F11**	2.7e-06	0.000246%	0.0	1	0.000244	4.01e-05	0.000206	1.86e-05	0.000595%
**OTU_7343**	**True**	**ABY1**	2.69e-06	0.000245%	0.0	1	4.73e-07	0.000781	0.00113	1.12e-05	0.000358%
**L740**	**False**	**PBS-25**	2.68e-06	0.000244%	0.0	1	0.00137	9.48e-05	0.00119	1.81e-05	0.000581%
**OTU_7592**	**True**	**ZB2**	2.67e-06	0.000243%	0.0	1	0.0	0.0	0.000306	6.08e-06	0.000195%
**L1746**	**False**	**OD1**	2.67e-06	0.000243%	0.0	1	0.00162	0.000914	0.00235	4.03e-05	0.00129%
**OTU_328**	**True**	**koll11**	2.67e-06	0.000243%	0.0	1	0.000398	0.0	0.0	5.53e-06	0.000177%
**L1114**	**False**	**koll11**	2.67e-06	0.000243%	0.0	1	0.00399	0.000176	0.000525	3.59e-06	0.000115%
**L1340**	**False**	**ZB2**	2.65e-06	0.000242%	0.0	1	0.000973	0.000508	4.41e-05	8.29e-06	0.000265%
**OTU_5343**	**True**	**ZB2**	2.65e-06	0.000241%	0.0	1	0.000655	8.49e-05	2.84e-05	5.65e-06	0.000181%

**L1616**	**False**	**ZB2**	2.64e-06	0.00024%	0.0	1	0.000574	2.36e-05	5.9e-05	6.66e-06	0.000213%
**OTU_7231**	**True**	**ABY1**	2.64e-06	0.00024%	0.0	1	0.000274	0.0	0.0	4.03e-06	0.000129%
**L1075**	**False**	**koll11**	2.63e-06	0.00024%	0.0	1	0.00232	0.000143	0.0	3.66e-06	0.000117%
**OTU_227**	**True**	**koll11**	2.62e-06	0.000239%	0.0	1	0.00183	0.000267	0.000357	9.19e-06	0.000295%
**OTU_1449**	**True**	**PBS-25**	2.62e-06	0.000239%	0.0	1	0.0133	0.00147	0.00466	4.46e-06	0.000143%
**OTU_1583**	**True**	**koll11**	2.61e-06	0.000238%	0.0	1	0.00026	4.06e-06	1.57e-05	6.09e-06	0.000195%
**L1202**	**False**	**koll11**	2.61e-06	0.000238%	0.0	1	0.00112	5.12e-05	9.29e-05	4.49e-06	0.000144%
**L772**	**False**	**PBS-25**	2.6e-06	0.000237%	0.0	1	0.00365	0.00031	0.000408	5.65e-06	0.000181%
**OTU_5490**	**True**	**Mb-NB09**	2.6e-06	0.000237%	0.0	1	0.000726	5.77e-05	0.0	6.19e-06	0.000198%
**OTU_8208**	**True**	**ABY1**	2.59e-06	0.000236%	0.0	1	7.58e-05	0.0	8.63e-06	7.45e-06	0.000239%
**L655**	**False**	**koll11**	2.58e-06	0.000235%	0.0	1	0.00784	0.000607	0.00198	2.14e-06	6.86e-05%
**OTU_5881**	**True**	**OD1**	2.58e-06	0.000235%	0.0	1	0.000706	0.000209	0.000257	1.2e-05	0.000384%
**OTU_6598**	**True**	**ZB2**	2.58e-06	0.000235%	0.0	1	0.0	0.000236	0.000791	7.5e-06	0.00024%
**OTU_7959**	**True**	**ABY1**	2.57e-06	0.000234%	0.0	1	0.000574	0.0	0.0	4.24e-06	0.000136%
**OTU_7052**	**True**	**ABY1**	2.56e-06	0.000233%	0.0	1	0.00105	9.87e-05	0.0	6.56e-06	0.00021%
**L1121**	**False**	**koll11**	2.55e-06	0.000232%	0.0	1	0.00155	0.000101	0.000116	3.98e-06	0.000128%
**OTU_1282**	**True**	**PRR-12**	2.53e-06	0.000231%	0.0	1	0.000665	4.65e-05	2e-06	5.49e-06	0.000176%
**OTU_9070**	**True**	**ABY1**	2.53e-06	0.000231%	0.0	1	5.1e-06	0.000283	0.00028	1.23e-05	0.000394%
**L202**	**False**	**ABY1**	2.53e-06	0.00023%	0.0	1	0.00289	9.63e-05	0.000772	4.24e-06	0.000136%
**L1344**	**False**	**ZB2**	2.52e-06	0.00023%	0.0	1	0.000563	0.000352	2.52e-05	1.1e-05	0.000353%
**OTU_5531**	**True**	**OD1**	2.52e-06	0.00023%	0.0	1	0.000316	1.3e-06	0.0	4.51e-06	0.000145%
**L674**	**False**	**koll11**	2.51e-06	0.000228%	0.0	1	0.00786	0.00161	0.0024	4.75e-06	0.000152%
**L944**	**False**	**koll11**	2.5e-06	0.000227%	0.0	1	0.0046	0.000492	0.00106	2.4e-06	7.68e-05%
**L1185**	**False**	**GIF10**	2.49e-06	0.000227%	0.0	1	0.00114	0.000183	7.01e-05	4.14e-06	0.000133%
**OTU_307**	**True**	**koll11**	2.49e-06	0.000227%	0.0	1	0.00397	0.000227	0.00108	4.83e-06	0.000155%
**OTU_2626**	**True**	**SJA-4**	2.49e-06	0.000227%	0.0	1	3.49e-05	4.42e-07	0.000581	7.39e-06	0.000237%
**L1648**	**False**	**ZB2**	2.49e-06	0.000227%	0.0	1	0.000438	0.000194	1.08e-05	6.9e-06	0.000221%
**OTU_5528**	**True**	**ZB2**	2.49e-06	0.000227%	0.0	1	0.000198	5.2e-05	8.5e-06	7.48e-06	0.00024%
**OTU_418**	**True**	**koll11**	2.48e-06	0.000226%	0.0	1	0.00122	3.74e-05	5.05e-06	3.08e-06	9.86e-05%
**OTU_4887**	**True**	**koll11**	2.47e-06	0.000225%	0.0	1	0.000475	4.71e-06	4.3e-05	4.76e-06	0.000153%
**OTU_7966**	**True**	**ZB2**	2.47e-06	0.000225%	0.0	1	0.0	0.000328	8.13e-05	8.4e-06	0.000269%
**OTU_9808**	**True**	**TM7-1**	2.47e-06	0.000225%	0.0	1	0.000284	0.0	0.0	5.06e-06	0.000162%
**L245**	**False**	**ABY1**	2.46e-06	0.000224%	0.0	1	0.000104	2.69e-05	0.00103	8.22e-06	0.000263%
**L1168**	**False**	**GIF10**	2.46e-06	0.000224%	0.0	1	0.00123	0.00025	4.74e-05	4.74e-06	0.000152%
**L1469**	**False**	**ZB2**	2.46e-06	0.000224%	0.0	1	0.000473	1.12e-05	1.58e-05	3.28e-06	0.000105%
**OTU_7974**	**True**	**ABY1**	2.45e-06	0.000223%	0.0	1	0.000122	0.00018	7.06e-06	1.23e-05	0.000395%
**L684**	**False**	**koll11**	2.44e-06	0.000222%	0.0	1	0.000979	3.69e-05	3.08e-05	5.59e-06	0.000179%
**OTU_6672**	**True**	**ABY1**	2.44e-06	0.000222%	0.0	1	7.07e-05	0.000626	0.000225	1.51e-05	0.000483%
**OTU_278**	**True**	**koll11**	2.43e-06	0.000222%	0.0	1	0.000842	8.81e-05	0.00011	6.96e-06	0.000223%
**OTU_414**	**True**	**PRR-12**	2.42e-06	0.000221%	0.0	1	0.0012	3.67e-06	0.0	3.81e-06	0.000122%
**OTU_998**	**True**	**PBS-25**	2.42e-06	0.000221%	0.0	1	0.00043	0.0	0.0	5.06e-06	0.000162%
**OTU_8222**	**True**	**ABY1**	2.42e-06	0.000221%	0.0	1	0.000104	0.0	7.42e-08	4.43e-06	0.000142%
**L1087**	**False**	**koll11**	2.41e-06	0.00022%	0.0	1	0.000667	0.000182	8.69e-05	9.19e-06	0.000294%
**L1067**	**False**	**koll11**	2.41e-06	0.000219%	0.0	1	0.000746	4.81e-05	0.000196	7.53e-06	0.000241%
**OTU_8932**	**True**	**Mb-NB09**	2.4e-06	0.000219%	0.0	1	0.000518	0.000129	2.82e-05	7.42e-06	0.000238%
**OTU_8198**	**True**	**OD1**	2.38e-06	0.000217%	0.0	1	0.000157	2.61e-05	3.39e-06	8.09e-06	0.000259%
**OTU_8201**	**True**	**ABY1**	2.38e-06	0.000217%	0.0	1	0.000146	1.19e-05	7.42e-08	4.63e-06	0.000148%
**OTU_5423**	**True**	**OD1**	2.38e-06	0.000217%	0.0	1	9.16e-05	0.0	0.0	4.86e-06	0.000156%
**OTU_8267**	**True**	**ABY1**	2.37e-06	0.000216%	0.0	1	8.85e-06	0.00301	0.00266	5.31e-06	0.00017%
**OTU_2571**	**True**	**PBS-25**	2.35e-06	0.000214%	0.0	1	0.000248	1.53e-05	5.22e-06	6.05e-06	0.000194%
**L747**	**False**	**PBS-25**	2.35e-06	0.000214%	0.0	1	0.00584	0.000656	0.00107	3.07e-06	9.84e-05%
**L387**	**False**	**Bacteria**	2.35e-06	0.000214%	0.0	1	0.835	0.844	0.902	2.3e-05	0.000737%
**OTU_8928**	**True**	**TM7-1**	2.34e-06	0.000213%	0.0	1	0.000344	8.86e-05	2.3e-05	9.68e-06	0.00031%
**OTU_6953**	**True**	**ZB2**	2.32e-06	0.000212%	0.0	1	0.0	0.000421	0.0	2.86e-06	9.16e-05%
**OTU_1094**	**True**	**PBS-25**	2.32e-06	0.000211%	0.0	1	0.00135	0.000149	7.05e-05	4.92e-06	0.000158%
**L1070**	**False**	**koll11**	2.31e-06	0.000211%	0.0	1	0.000782	7.12e-06	3.8e-06	5.53e-06	0.000177%
**OTU_5997**	**True**	**OD1**	2.3e-06	0.00021%	0.0	1	6.28e-05	3.91e-06	0.000159	1.29e-05	0.000414%
**L1434**	**False**	**ZB2**	2.3e-06	0.000209%	0.0	1	0.00231	0.000376	0.000299	4.87e-06	0.000156%
**L1303**	**False**	**TM7**	2.3e-06	0.000209%	0.0	1	0.000786	0.000142	0.000177	1.03e-05	0.00033%
**OTU_10765**	**True**	**OP11-4**	2.3e-06	0.000209%	0.0	1	0.000692	4.89e-05	7.7e-05	3.03e-06	9.72e-05%
**L1118**	**False**	**koll11**	2.29e-06	0.000209%	0.0	1	0.00405	0.000227	0.00108	4.28e-06	0.000137%
**OTU_306**	**True**	**koll11**	2.29e-06	0.000209%	0.0	1	0.00108	7.42e-05	0.000283	5.61e-06	0.00018%
**L1481**	**False**	**ZB2**	2.29e-06	0.000209%	0.0	1	0.000474	0.0	0.0	4.19e-06	0.000134%
**L462**	**False**	**OP11-4**	2.28e-06	0.000208%	0.0	1	0.000745	0.000116	0.000108	5.72e-06	0.000183%
**OTU_259**	**True**	**PBS-25**	2.28e-06	0.000208%	0.0	1	0.00247	0.000692	0.000949	8.31e-06	0.000266%

**OTU_178**	**True**	**koll11**	2.27e-06	0.000207%	0.0	1	0.00119	0.000209	0.000291	9.68e-06	0.00031%
**OTU_3998**	**True**	**koll11**	2.27e-06	0.000207%	0.0	1	0.000251	2.29e-05	5.03e-05	6.89e-06	0.000221%
**OTU_5951**	**True**	**OD1**	2.27e-06	0.000207%	0.0	1	0.000166	1.82e-05	6.98e-06	6.3e-06	0.000202%
**OTU_1825**	**True**	**koll11**	2.26e-06	0.000206%	0.0	1	0.000754	0.000147	0.000352	1.31e-05	0.00042%
**L340**	**False**	**OD1**	2.26e-06	0.000206%	0.0	1	0.000521	2.42e-05	5.77e-06	5.21e-06	0.000167%
**L351**	**False**	**OD1**	2.25e-06	0.000205%	0.0	1	0.000584	0.000113	1.61e-05	5.05e-06	0.000162%
**OTU_7219**	**True**	**ZB2**	2.24e-06	0.000204%	0.0	1	3.85e-05	0.000342	0.0	6.72e-06	0.000215%
**OTU_858**	**True**	**koll11**	2.24e-06	0.000204%	0.0	1	0.000395	2.67e-05	0.0	7.91e-06	0.000253%
**OTU_4008**	**True**	**koll11**	2.24e-06	0.000204%	0.0	1	0.00288	0.000273	0.000972	6.16e-06	0.000197%
**OTU_8749**	**True**	**OD1**	2.23e-06	0.000203%	0.0	1	0.000432	0.000102	4.54e-05	6.52e-06	0.000209%
**L1487**	**False**	**ZB2**	2.22e-06	0.000202%	0.0	1	0.00219	0.000311	0.000605	6.91e-06	0.000221%
**L1394**	**False**	**ZB2**	2.22e-06	0.000202%	0.0	1	0.00453	0.00316	0.00218	3.07e-05	0.000983%
**L761**	**False**	**PBS-25**	2.22e-06	0.000202%	0.0	1	0.00248	0.000107	0.000147	6.76e-06	0.000217%
**L609**	**False**	**PRR-12**	2.21e-06	0.000201%	0.0	1	0.000797	0.0	0.0	3.05e-06	9.75e-05%
**OTU_811**	**True**	**koll11**	2.21e-06	0.000201%	0.0	1	0.000402	0.0	1.42e-05	3.2e-06	0.000103%
**L1570**	**False**	**ZB2**	2.21e-06	0.000201%	0.0	1	0.00196	0.000256	0.000611	4.94e-06	0.000158%
**L373**	**False**	**ABY1**	2.2e-06	0.000201%	0.0	1	0.000501	0.000287	2.15e-05	8.93e-06	0.000286%
**L1492**	**False**	**ZB2**	2.2e-06	0.0002%	0.0	1	0.000839	0.000154	2.15e-05	3.02e-06	9.68e-05%
**L915**	**False**	**OP3**	2.2e-06	0.0002%	0.0	1	0.00116	8.72e-05	4.95e-06	2.83e-06	9.06e-05%
**OTU_891**	**True**	**PRR-12**	2.19e-06	0.0002%	0.0	1	0.00117	0.000147	7.07e-05	6.03e-06	0.000193%
**OTU_345**	**True**	**koll11**	2.18e-06	0.000199%	0.0	1	3.65e-05	6.01e-05	0.000456	1.3e-05	0.000417%
**L1502**	**False**	**ZB2**	2.18e-06	0.000198%	0.0	1	0.000213	3.82e-05	4.16e-05	1.48e-05	0.000475%
**L1172**	**False**	**GIF10**	2.17e-06	0.000197%	0.0	1	0.00107	0.00025	4.74e-05	4.82e-06	0.000155%
**OTU_6161**	**True**	**ABY1**	2.16e-06	0.000197%	0.0	1	0.000145	0.0	0.0	4.66e-06	0.000149%
**L237**	**False**	**ABY1**	2.16e-06	0.000196%	0.0	1	0.0015	3.57e-05	3.19e-05	3.1e-06	9.92e-05%
**OTU_2529**	**True**	**GIF10**	2.15e-06	0.000196%	0.0	1	0.000506	1.54e-05	1.41e-05	4.59e-06	0.000147%
**OTU_6108**	**True**	**ZB2**	2.14e-06	0.000195%	0.0	1	0.0	0.000137	2.25e-06	4.49e-06	0.000144%
**OTU_10850**	**True**	**OP11-4**	2.14e-06	0.000195%	0.0	1	0.000113	0.0	0.0	3.88e-06	0.000124%
**OTU_5514**	**True**	**OD1**	2.13e-06	0.000194%	0.0	1	0.000136	1.87e-05	0.0	5.86e-06	0.000188%
**OTU_7207**	**True**	**ZB2**	2.13e-06	0.000194%	0.0	1	9.83e-05	2.2e-06	2.14e-06	4.66e-06	0.000149%
**OTU_904**	**True**	**koll11**	2.13e-06	0.000194%	0.0	1	0.000433	1.01e-05	0.0	3.5e-06	0.000112%
**OTU_549**	**True**	**koll11**	2.13e-06	0.000194%	0.0	1	0.00023	0.0	2.72e-05	5.2e-06	0.000167%
**L280**	**False**	**ABY1**	2.12e-06	0.000193%	0.0	1	0.000395	2.57e-05	0.0	4.13e-06	0.000132%
**OTU_2412**	**True**	**SJA-4**	2.11e-06	0.000192%	0.0	1	0.000167	8.8e-06	0.0	5.1e-06	0.000163%
**L1707**	**False**	**Mb-NB09**	2.11e-06	0.000192%	0.0	1	0.000989	8.87e-05	4.57e-05	3.4e-06	0.000109%
**OTU_7938**	**True**	**ABY1**	2.1e-06	0.000191%	0.0	1	0.00028	2.25e-06	2.87e-05	4.49e-06	0.000144%
**OTU_10809**	**True**	**OP11-4**	2.1e-06	0.000191%	0.0	1	0.000289	4.86e-05	0.0	4.38e-06	0.00014%
**OTU_8239**	**True**	**ABY1**	2.09e-06	0.000191%	0.0	1	0.000228	1.66e-06	0.0	4.13e-06	0.000132%
**OTU_6680**	**True**	**OD1**	2.09e-06	0.000191%	0.0	1	0.000257	0.0	1.65e-05	3.26e-06	0.000104%
**OTU_8990**	**True**	**ABY1**	2.08e-06	0.000189%	0.0	1	0.0	0.000338	0.000168	9.2e-06	0.000295%
**L1170**	**False**	**GIF10**	2.08e-06	0.000189%	0.0	1	0.00107	0.00025	4.74e-05	4.62e-06	0.000148%
**L34**	**False**	**ABY1**	2.08e-06	0.000189%	0.0	1	0.000413	1.93e-05	1.91e-05	3.57e-06	0.000114%
**L445**	**False**	**OP11-4**	2.07e-06	0.000189%	0.0	1	0.00139	7.45e-05	0.000909	9.12e-06	0.000292%
**OTU_6976**	**True**	**ZB2**	2.07e-06	0.000189%	0.0	1	0.000301	2.76e-05	2.43e-05	7.87e-06	0.000252%
**OTU_892**	**True**	**BD4-9**	2.07e-06	0.000188%	0.0	1	0.000293	0.0	0.0	5.11e-06	0.000164%
**OTU_7929**	**True**	**OD1**	2.06e-06	0.000188%	0.0	1	9.59e-05	0.0	8.29e-07	4.34e-06	0.000139%
**L1880**	**False**	**OD1**	2.05e-06	0.000187%	0.0	1	0.00031	4.8e-05	1.68e-05	5.04e-06	0.000161%
**OTU_4010**	**True**	**koll11**	2.03e-06	0.000185%	0.0	1	0.000545	0.000255	7.35e-06	6.65e-06	0.000213%
**L1291**	**False**	**TM7-3**	2.02e-06	0.000184%	0.0	1	0.00162	5.22e-07	3.05e-05	3.15e-06	0.000101%
**L1620**	**False**	**ZB2**	2.01e-06	0.000183%	0.0	1	0.00199	0.00149	0.000866	2.87e-05	0.000919%
**OTU_821**	**True**	**koll11**	2.01e-06	0.000183%	0.0	1	0.000186	0.0	0.0	4.29e-06	0.000137%
**OTU_817**	**True**	**BD4-9**	2.01e-06	0.000183%	0.0	1	0.000119	0.0	0.0	3.66e-06	0.000117%
**OTU_8021**	**True**	**ABY1**	1.99e-06	0.000181%	0.0	1	0.000637	3.35e-05	3.19e-06	4.38e-06	0.00014%
**L1651**	**False**	**ZB2**	1.99e-06	0.000181%	0.0	1	0.000178	8.52e-05	0.000818	1.02e-05	0.000328%
**L720**	**False**	**PBS-25**	1.97e-06	0.000179%	0.0	1	0.00217	0.000314	0.000626	5.44e-06	0.000174%
**OTU_1539**	**True**	**PBS-25**	1.97e-06	0.000179%	0.0	1	0.000141	0.0	0.0	4.19e-06	0.000134%
**L906**	**False**	**koll11**	1.96e-06	0.000179%	0.0	1	0.00115	0.00025	0.00105	1.65e-05	0.000528%
**OTU_8006**	**True**	**TM7-1**	1.96e-06	0.000178%	0.0	1	0.00036	4.44e-05	0.0	5.24e-06	0.000168%
**OTU_559**	**True**	**koll11**	1.95e-06	0.000178%	0.0	1	0.000339	1.61e-05	3.45e-05	4.93e-06	0.000158%
**OTU_6189**	**True**	**ZB2**	1.95e-06	0.000178%	0.0	1	0.0	0.000409	8.73e-05	3.16e-06	0.000101%
**OTU_6069**	**True**	**ABY1**	1.95e-06	0.000178%	0.0	1	0.000321	4.16e-05	3.57e-05	6.11e-06	0.000196%
**L1266**	**False**	**TM7-1**	1.94e-06	0.000177%	0.0	1	0.00136	0.000263	7.09e-05	4.19e-06	0.000134%
**OTU_262**	**True**	**koll11**	1.94e-06	0.000177%	0.0	1	0.00749	0.00107	0.00214	5.15e-06	0.000165%
**OTU_8012**	**True**	**ZB2**	1.93e-06	0.000176%	0.0	1	0.000759	1.77e-05	0.000199	5.7e-06	0.000183%
**L1877**	**False**	**OD1**	1.93e-06	0.000176%	0.0	1	0.000755	0.000188	4.05e-05	4.78e-06	0.000153%

**OTU_8050**	**True**	**ABY1**	1.92e-06	0.000175%	0.0	1	0.000161	4.45e-05	1.81e-07	6e-06	0.000192%
**OTU_6569**	**True**	**ZB2**	1.92e-06	0.000175%	0.0	1	0.000332	3.01e-05	1.64e-05	4.48e-06	0.000144%
**OTU_466**	**True**	**koll11**	1.92e-06	0.000175%	0.0	1	0.000202	6.78e-06	0.000104	1.05e-05	0.000335%
**L665**	**False**	**koll11**	1.91e-06	0.000174%	0.0	1	0.00305	8.14e-05	0.000354	2.93e-06	9.37e-05%
**OTU_8936**	**True**	**Mb-NB09**	1.9e-06	0.000173%	0.0	1	0.0	0.0002	1.24e-05	4.84e-06	0.000155%
**L214**	**False**	**ABY1**	1.89e-06	0.000172%	0.0	1	0.000256	3.17e-05	0.0	4.75e-06	0.000152%
**L696**	**False**	**koll11**	1.89e-06	0.000172%	0.0	1	0.00193	0.000563	5.65e-05	2.71e-06	8.67e-05%
**OTU_8219**	**True**	**ABY1**	1.88e-06	0.000171%	0.0	1	0.000137	9.47e-06	1.3e-05	6.71e-06	0.000215%
**OTU_8247**	**True**	**ZB2**	1.87e-06	0.000171%	0.0	1	0.000413	1.14e-05	2.97e-06	3.4e-06	0.000109%
**OTU_2577**	**True**	**PBS-25**	1.87e-06	0.000171%	0.0	1	0.000835	9.94e-05	3.42e-05	5.33e-06	0.000171%
**OTU_293**	**True**	**koll11**	1.87e-06	0.00017%	0.0	1	0.000462	0.000214	0.000124	2.2e-05	0.000706%
**OTU_10770**	**True**	**OP11-4**	1.86e-06	0.00017%	0.0	1	0.000246	8.77e-05	3.05e-06	5.39e-06	0.000173%
**OTU_3769**	**True**	**koll11**	1.86e-06	0.00017%	0.0	1	0.00138	0.000307	4.91e-05	3.5e-06	0.000112%
**OTU_509**	**True**	**PBS-25**	1.86e-06	0.000169%	0.0	1	0.00114	9.98e-05	5.34e-05	5.05e-06	0.000162%
**L1875**	**False**	**OD1**	1.86e-06	0.000169%	0.0	1	0.000954	0.000336	5.06e-05	5.42e-06	0.000174%
**L451**	**False**	**OP11-4**	1.85e-06	0.000168%	0.0	1	0.00117	5.38e-05	0.000775	9.64e-06	0.000309%
**OTU_8227**	**True**	**OD1**	1.85e-06	0.000168%	0.0	1	0.000118	7.22e-06	1.53e-05	5.66e-06	0.000181%
**OTU_8164**	**True**	**Mb-NB09**	1.85e-06	0.000168%	0.0	1	0.000668	0.000413	6.22e-05	8.14e-06	0.000261%
**OTU_9341**	**True**	**TM7-1**	1.84e-06	0.000168%	0.0	1	0.000224	3.22e-06	3.68e-05	4.77e-06	0.000153%
**L65**	**False**	**ABY1**	1.84e-06	0.000168%	0.0	1	0.00048	1.02e-06	0.00011	5.93e-06	0.00019%
**OTU_2714**	**True**	**koll11**	1.84e-06	0.000168%	0.0	1	0.00119	0.000244	0.000824	1.37e-05	0.000438%
**L739**	**False**	**PBS-25**	1.84e-06	0.000167%	0.0	1	0.00251	0.000195	0.00124	8.43e-06	0.00027%
**OTU_2279**	**True**	**OP3**	1.83e-06	0.000167%	0.0	1	0.00125	4e-05	0.0	2.44e-06	7.8e-05%
**OTU_3356**	**True**	**SBRH58**	1.82e-06	0.000166%	0.0	1	0.000349	0.0	7.42e-08	2.98e-06	9.56e-05%
**L728**	**False**	**PBS-25**	1.82e-06	0.000166%	0.0	1	0.00842	0.000857	0.000909	2.66e-06	8.53e-05%
**OTU_6219**	**True**	**ZB2**	1.82e-06	0.000166%	0.0	1	0.000296	2.4e-05	0.0	4.96e-06	0.000159%
**OTU_190**	**True**	**koll11**	1.82e-06	0.000165%	0.0	1	0.00252	0.000588	0.00208	1.57e-05	0.000503%
**L1734**	**False**	**Mb-NB09**	1.81e-06	0.000165%	0.0	1	0.000909	0.000413	6.22e-05	5.21e-06	0.000167%
**OTU_2202**	**True**	**PBS-25**	1.8e-06	0.000164%	0.0	1	0.000221	0.0	0.0	4.36e-06	0.00014%
**OTU_367**	**True**	**koll11**	1.79e-06	0.000163%	0.0	1	0.000515	4.81e-05	0.00019	7e-06	0.000224%
**OTU_860**	**True**	**PBS-25**	1.79e-06	0.000163%	0.0	1	0.000217	0.0	0.0	5.24e-06	0.000168%
**OTU_1432**	**True**	**koll11**	1.79e-06	0.000163%	0.0	1	0.000482	5.14e-06	0.0	1.98e-06	6.36e-05%
**L1086**	**False**	**koll11**	1.79e-06	0.000163%	0.0	1	0.00318	0.000635	0.000361	3.23e-06	0.000104%
**OTU_445**	**True**	**koll11**	1.78e-06	0.000163%	0.0	1	0.000435	9.09e-05	7.13e-05	6.67e-06	0.000214%
**OTU_985**	**True**	**PBS-25**	1.77e-06	0.000162%	0.0	1	0.000518	3.65e-05	7.42e-08	3.26e-06	0.000104%
**L382**	**False**	**OD1**	1.76e-06	0.000161%	0.0	1	0.000897	4.5e-05	5.3e-06	2.86e-06	9.17e-05%
**L1127**	**False**	**koll11**	1.76e-06	0.00016%	0.0	1	0.000769	0.000104	3.61e-05	6.36e-06	0.000204%
**OTU_554**	**True**	**PRR-12**	1.76e-06	0.00016%	0.0	1	0.000444	0.0	0.0	3.49e-06	0.000112%
**OTU_574**	**True**	**koll11**	1.75e-06	0.00016%	0.0	1	0.000253	1.71e-06	3.84e-05	4.82e-06	0.000155%
**OTU_9374**	**True**	**TM7-1**	1.75e-06	0.00016%	0.0	1	0.000291	0.0	0.0	3.35e-06	0.000107%
**L688**	**False**	**koll11**	1.75e-06	0.000159%	0.0	1	0.00486	0.00118	0.00146	3.65e-06	0.000117%
**L1899**	**False**	**OD1**	1.74e-06	0.000159%	0.0	1	0.000877	0.000218	0.000113	6.11e-06	0.000196%
**L1759**	**False**	**OD1**	1.73e-06	0.000158%	0.0	1	0.000317	0.0	0.000272	8.04e-06	0.000258%
**OTU_2194**	**True**	**koll11**	1.73e-06	0.000158%	0.0	1	0.000873	1.32e-05	0.00103	7.38e-06	0.000236%
**OTU_6222**	**True**	**ZB2**	1.73e-06	0.000157%	0.0	1	0.000243	3.29e-05	5.48e-05	6.55e-06	0.00021%
**L1283**	**False**	**TM7-3**	1.71e-06	0.000156%	0.0	1	0.000419	0.0	0.0	3.6e-06	0.000115%
**L1508**	**False**	**OD1**	1.7e-06	0.000155%	0.0	1	0.0136	0.00262	0.00749	2.93e-06	9.4e-05%
**L1203**	**False**	**koll11**	1.69e-06	0.000154%	0.0	1	0.000537	5.12e-05	9.29e-05	4.84e-06	0.000155%
**L615**	**False**	**BD4-9**	1.68e-06	0.000153%	0.0	1	0.000412	1.39e-05	0.0	3.46e-06	0.000111%
**OTU_2009**	**True**	**PRR-12**	1.66e-06	0.000152%	0.0	1	0.00137	0.000146	0.000214	3.34e-06	0.000107%
**OTU_7949**	**True**	**ABY1**	1.66e-06	0.000151%	0.0	1	0.000762	0.000104	6.04e-06	2.78e-06	8.9e-05%
**L225**	**False**	**ABY1**	1.65e-06	0.00015%	0.0	1	0.00122	0.00091	0.000582	4.63e-05	0.00148%
**OTU_5075**	**True**	**GIF10**	1.64e-06	0.00015%	0.0	1	0.000121	0.0	0.0	2.66e-06	8.51e-05%
**OTU_8213**	**True**	**ABY1**	1.64e-06	0.000149%	0.0	1	0.000142	9.42e-07	0.0	2.5e-06	8.01e-05%
**OTU_981**	**True**	**PRR-12**	1.63e-06	0.000149%	0.0	1	0.000759	9.62e-07	0.0	3.39e-06	0.000109%
**L152**	**False**	**ABY1**	1.62e-06	0.000148%	0.0	1	0.000476	0.000296	3.96e-05	9.84e-06	0.000315%
**L997**	**False**	**koll11**	1.62e-06	0.000147%	0.0	1	0.000943	0.000147	0.000369	6.11e-06	0.000196%
**OTU_7211**	**True**	**ABY1**	1.62e-06	0.000147%	0.0	1	8.18e-05	0.0	0.0	3.92e-06	0.000125%
**OTU_6184**	**True**	**SM2F11**	1.61e-06	0.000147%	0.0	1	0.0	0.000213	1.5e-05	3.95e-06	0.000127%
**OTU_363**	**True**	**koll11**	1.61e-06	0.000146%	0.0	1	0.00037	4.42e-07	0.0	2.48e-06	7.94e-05%
**OTU_498**	**True**	**wb1_H11**	1.59e-06	0.000145%	0.0	1	0.000353	0.0	0.0	2.95e-06	9.46e-05%
**OTU_2685**	**True**	**PBS-25**	1.59e-06	0.000145%	0.0	1	0.0	0.0	0.000366	3.76e-06	0.00012%
**OTU_5837**	**True**	**OD1**	1.59e-06	0.000144%	0.0	1	0.000179	7.57e-06	4.63e-05	6.42e-06	0.000206%
**OTU_9815**	**True**	**ABY1**	1.58e-06	0.000144%	0.0	1	0.000118	0.0	0.0	3.18e-06	0.000102%

**OTU_1232**	**True**	**PBS-25**	1.58e-06	0.000144%	0.0	1	0.00215	8.59e-05	9.04e-05	3.67e-06	0.000117%
**OTU_6708**	**True**	**ZB2**	1.57e-06	0.000143%	0.0	1	0.000106	0.0	0.0	2.87e-06	9.18e-05%
**OTU_8252**	**True**	**ABY1**	1.57e-06	0.000143%	0.0	1	0.00127	4.21e-05	5.93e-06	2.17e-06	6.95e-05%
**OTU_454**	**True**	**koll11**	1.57e-06	0.000143%	0.0	1	0.000484	4.35e-05	2.69e-05	2.91e-06	9.31e-05%
**L786**	**False**	**PBS-25**	1.56e-06	0.000142%	0.0	1	0.000831	0.00011	0.000375	7.61e-06	0.000244%
**OTU_295**	**True**	**koll11**	1.56e-06	0.000142%	0.0	1	2.32e-05	0.000219	0.0	5.83e-06	0.000187%
**L1285**	**False**	**TM7-3**	1.55e-06	0.000141%	0.0	1	0.000419	0.0	0.0	3.27e-06	0.000105%
**OTU_440**	**True**	**PBS-25**	1.55e-06	0.000141%	0.0	1	0.000802	0.000101	3.68e-05	2.95e-06	9.44e-05%
**OTU_6995**	**True**	**ABY1**	1.55e-06	0.000141%	0.0	1	0.001	0.000326	9.49e-05	3.69e-06	0.000118%
**L546**	**False**	**SJA-4**	1.54e-06	0.00014%	0.0	1	0.00044	0.00023	0.00101	2.06e-05	0.000661%
**OTU_7958**	**True**	**ABY1**	1.53e-06	0.000139%	0.0	1	0.000116	9.53e-06	0.0	4e-06	0.000128%
**L414**	**False**	**ABY1**	1.52e-06	0.000138%	0.0	1	5.72e-06	0.000569	0.00196	4.66e-06	0.000149%
**OTU_7943**	**True**	**ABY1**	1.52e-06	0.000138%	0.0	1	0.0001	0.0	0.0	3.56e-06	0.000114%
**OTU_501**	**True**	**koll11**	1.52e-06	0.000138%	0.0	1	0.000244	0.0	0.0	2.16e-06	6.92e-05%
**OTU_8865**	**True**	**TM7-1**	1.51e-06	0.000138%	0.0	1	0.0	5.31e-05	0.000297	5.28e-06	0.000169%
**OTU_659**	**True**	**koll11**	1.5e-06	0.000137%	0.0	1	0.000144	0.0	0.0	2.75e-06	8.81e-05%
**OTU_3274**	**True**	**PBS-25**	1.49e-06	0.000136%	0.0	1	0.000322	2.36e-05	0.0	3.17e-06	0.000102%
**L48**	**False**	**OD1**	1.49e-06	0.000136%	0.0	1	0.000493	0.000405	5.86e-06	7.44e-06	0.000238%
**OTU_893**	**True**	**PBS-25**	1.49e-06	0.000136%	0.0	1	0.000582	6.58e-05	7.42e-08	3.47e-06	0.000111%
**OTU_6633**	**True**	**ZB2**	1.47e-06	0.000134%	0.0	1	0.000164	4.94e-06	8.03e-05	5.64e-06	0.000181%
**L1639**	**False**	**ZB2**	1.47e-06	0.000134%	0.0	1	0.00118	0.000307	0.00104	1.07e-05	0.000343%
**L571**	**False**	**SJA-4**	1.46e-06	0.000133%	0.0	1	0.000167	1.47e-05	0.0	3.79e-06	0.000122%
**OTU_6305**	**True**	**OD1**	1.46e-06	0.000133%	0.0	1	6.19e-05	0.0	0.0	3.51e-06	0.000112%
**OTU_6515**	**True**	**SM2F11**	1.45e-06	0.000133%	0.0	1	0.000267	0.0	3.22e-05	3.77e-06	0.000121%
**OTU_7029**	**True**	**OD1**	1.45e-06	0.000132%	0.0	1	0.00022	0.000249	7.35e-05	2.02e-05	0.000648%
**OTU_562**	**True**	**koll11**	1.45e-06	0.000132%	0.0	1	0.000395	2.78e-05	6.04e-05	3.68e-06	0.000118%
**OTU_1042**	**True**	**OP3**	1.44e-06	0.000131%	0.0	1	0.000315	4.48e-05	5.7e-06	2.57e-06	8.24e-05%
**OTU_5491**	**True**	**OD1**	1.43e-06	0.00013%	0.0	1	0.000443	0.0	0.0	2.49e-06	7.99e-05%
**OTU_8762**	**True**	**OD1**	1.42e-06	0.00013%	0.0	1	0.000236	9.94e-06	2.71e-05	3.16e-06	0.000101%
**OTU_8757**	**True**	**OD1**	1.42e-06	0.000129%	0.0	1	0.000127	0.0	8.21e-05	6.2e-06	0.000199%
**OTU_6930**	**True**	**ZB2**	1.42e-06	0.000129%	0.0	1	0.000169	0.0	0.0	3.02e-06	9.66e-05%
**OTU_7980**	**True**	**OP11-4**	1.41e-06	0.000129%	0.0	1	0.000145	2.53e-05	2.3e-05	4.96e-06	0.000159%
**OTU_6005**	**True**	**SM2F11**	1.41e-06	0.000129%	0.0	1	0.00024	2.67e-05	3.03e-05	4.04e-06	0.00013%
**OTU_5377**	**True**	**ZB2**	1.41e-06	0.000128%	0.0	1	0.000218	2.16e-05	5.83e-05	7.24e-06	0.000232%
**L553**	**False**	**SJA-4**	1.39e-06	0.000126%	0.0	1	0.000694	6.99e-05	5.85e-05	4.74e-06	0.000152%
**OTU_10934**	**True**	**GIF10**	1.38e-06	0.000126%	0.0	1	0.000161	0.0	0.0	2.56e-06	8.19e-05%
**OTU_550**	**True**	**koll11**	1.38e-06	0.000126%	0.0	1	0.000231	0.0	5.69e-06	3.57e-06	0.000114%
**OTU_906**	**True**	**koll11**	1.38e-06	0.000125%	0.0	1	0.000847	0.000131	7.56e-05	2.73e-06	8.75e-05%
**L719**	**False**	**PBS-25**	1.37e-06	0.000125%	0.0	1	0.00258	0.000418	0.000726	3.44e-06	0.00011%
**OTU_5392**	**True**	**ZB2**	1.36e-06	0.000124%	0.0	1	6.06e-05	0.0	6.9e-06	4.24e-06	0.000136%
**OTU_6579**	**True**	**ZB2**	1.36e-06	0.000124%	0.0	1	1.85e-06	0.000127	0.000531	5.88e-06	0.000188%
**L1749**	**False**	**OD1**	1.35e-06	0.000123%	0.0	1	0.00114	0.000751	0.00196	2.25e-05	0.000722%
**OTU_4122**	**True**	**koll11**	1.35e-06	0.000123%	0.0	1	0.000522	8.62e-05	0.000163	9.51e-06	0.000305%
**L1359**	**False**	**ZB2**	1.35e-06	0.000123%	0.0	1	0.000292	0.0	0.000365	7.09e-06	0.000227%
**OTU_8373**	**True**	**ABY1**	1.34e-06	0.000122%	0.0	1	0.000151	2.25e-07	1.74e-05	3e-06	9.62e-05%
**OTU_580**	**True**	**PBS-25**	1.33e-06	0.000122%	0.0	1	0.00146	0.00016	0.000161	2.79e-06	8.95e-05%
**OTU_7215**	**True**	**ABY1**	1.33e-06	0.000121%	0.0	1	0.00059	0.000146	2.46e-05	3.21e-06	0.000103%
**OTU_1140**	**True**	**PBS-25**	1.33e-06	0.000121%	0.0	1	0.000447	5.68e-05	0.000298	1.17e-05	0.000376%
**OTU_8143**	**True**	**OP11-4**	1.33e-06	0.000121%	0.0	1	9.94e-06	1.08e-06	0.000109	4.74e-06	0.000152%
**OTU_4802**	**True**	**GIF10**	1.32e-06	0.000121%	0.0	1	0.000227	3.88e-05	0.0	4.25e-06	0.000136%
**OTU_1244**	**True**	**koll11**	1.31e-06	0.000119%	0.0	1	0.000602	0.000216	0.000218	1.34e-05	0.000428%
**OTU_4247**	**True**	**koll11**	1.31e-06	0.000119%	0.0	1	0.000492	1.04e-05	3.08e-05	2.93e-06	9.39e-05%
**OTU_6933**	**True**	**ZB2**	1.31e-06	0.000119%	0.0	1	0.000271	6.07e-05	0.0	4.03e-06	0.000129%
**OTU_7930**	**True**	**ZB2**	1.31e-06	0.000119%	0.0	1	0.000134	0.0	0.0	2.62e-06	8.39e-05%
**OTU_8126**	**True**	**ABY1**	1.3e-06	0.000119%	0.0	1	0.0009	1.07e-06	3e-05	3.49e-06	0.000112%
**L817**	**False**	**PBS-25**	1.29e-06	0.000118%	0.0	1	0.00131	0.00012	7.42e-08	2.95e-06	9.46e-05%
**OTU_7041**	**True**	**ZB2**	1.29e-06	0.000118%	0.0	1	1.5e-06	0.000492	0.000384	4.2e-06	0.000135%
**L693**	**False**	**koll11**	1.28e-06	0.000117%	0.0	1	0.00162	0.000317	0.000582	4.83e-06	0.000155%
**OTU_5887**	**True**	**OD1**	1.28e-06	0.000116%	0.0	1	0.000311	2.51e-05	4e-06	2.31e-06	7.39e-05%
**OTU_966**	**True**	**PRR-12**	1.28e-06	0.000116%	0.0	1	0.0	4.5e-05	0.000126	6.97e-06	0.000223%
**OTU_4833**	**True**	**OD1**	1.27e-06	0.000116%	0.0	1	7.5e-05	0.0	7.42e-08	3e-06	9.59e-05%
**OTU_171**	**True**	**koll11**	1.27e-06	0.000116%	0.0	1	0.00128	0.000373	0.00102	1.1e-05	0.000352%
**L683**	**False**	**koll11**	1.27e-06	0.000116%	0.0	1	0.000979	3.69e-05	3.08e-05	2.9e-06	9.3e-05%
**OTU_429**	**True**	**PBS-25**	1.27e-06	0.000116%	0.0	1	2.91e-05	8.98e-06	0.000338	4.61e-06	0.000148%

**OTU_5265**	**True**	**OD1**	1.26e-06	0.000115%	0.0	1	0.000644	7.57e-05	2.02e-05	2.46e-06	7.87e-05%
**OTU_6542**	**True**	**OD1**	1.26e-06	0.000115%	0.0	1	0.000238	0.000532	4.47e-05	8.2e-06	0.000263%
**OTU_1218**	**True**	**koll11**	1.26e-06	0.000114%	0.0	1	0.000268	0.000111	2.62e-05	5.57e-06	0.000179%
**OTU_3804**	**True**	**PBS-25**	1.25e-06	0.000114%	0.0	1	0.000126	0.0	0.0	2.66e-06	8.51e-05%
**L1104**	**False**	**koll11**	1.25e-06	0.000114%	0.0	1	0.000863	7.33e-05	0.000402	5.65e-06	0.000181%
**OTU_7225**	**True**	**ZB2**	1.24e-06	0.000113%	0.0	1	0.00288	0.000798	0.00135	8.45e-06	0.000271%
**OTU_926**	**True**	**koll11**	1.24e-06	0.000113%	0.0	1	0.00103	0.000212	0.000106	2.64e-06	8.46e-05%
**L1645**	**False**	**ZB2**	1.24e-06	0.000113%	0.0	1	0.000827	0.000304	0.00104	1.32e-05	0.000424%
**OTU_8009**	**True**	**ABY1**	1.24e-06	0.000113%	0.0	1	0.000785	0.0	0.0	2.59e-06	8.31e-05%
**OTU_1133**	**True**	**GIF10**	1.24e-06	0.000113%	0.0	1	0.000562	0.000234	3.34e-05	5.01e-06	0.000161%
**OTU_6758**	**True**	**ZB2**	1.23e-06	0.000112%	0.0	1	0.000213	9.09e-07	0.0	3.3e-06	0.000106%
**L1635**	**False**	**ZB2**	1.23e-06	0.000112%	0.0	1	0.000413	1.14e-05	2.97e-06	2.23e-06	7.13e-05%
**L302**	**False**	**ABY1**	1.22e-06	0.000111%	0.0	1	0.000349	1.37e-05	7e-06	2.28e-06	7.31e-05%
**OTU_5794**	**True**	**OD1**	1.22e-06	0.000111%	0.0	1	0.000305	2.46e-05	3.33e-05	2.1e-06	6.73e-05%
**OTU_6117**	**True**	**OD1**	1.21e-06	0.00011%	0.0	1	0.000358	5.65e-05	0.000388	1.12e-05	0.000359%
**L562**	**False**	**SJA-4**	1.21e-06	0.00011%	0.0	1	0.000289	0.000119	2.99e-07	3.49e-06	0.000112%
**OTU_8903**	**True**	**ABY1**	1.2e-06	0.00011%	0.0	1	0.00048	0.0	0.000103	3.78e-06	0.000121%
**OTU_10776**	**True**	**OP11-4**	1.2e-06	0.000109%	0.0	1	5.9e-05	3.7e-06	0.0	2.06e-06	6.59e-05%
**OTU_4482**	**True**	**koll11**	1.2e-06	0.000109%	0.0	1	0.000176	0.0	0.0	2.87e-06	9.18e-05%
**L1737**	**False**	**Mb-NB09**	1.19e-06	0.000109%	0.0	1	0.000328	1.43e-05	0.0	1.83e-06	5.85e-05%
**L1646**	**False**	**ZB2**	1.19e-06	0.000109%	0.0	1	0.00065	0.000219	0.000219	8.43e-06	0.00027%
**OTU_6070**	**True**	**ZB2**	1.19e-06	0.000109%	0.0	1	1.27e-05	0.000699	0.000636	6.83e-06	0.000219%
**OTU_2885**	**True**	**PBS-25**	1.19e-06	0.000108%	0.0	1	0.000473	5.18e-06	4.45e-06	1.32e-06	4.22e-05%
**L677**	**False**	**koll11**	1.19e-06	0.000108%	0.0	1	0.00156	0.000216	0.000885	6.12e-06	0.000196%
**L882**	**False**	**koll11**	1.18e-06	0.000108%	0.0	1	0.00686	0.00134	0.0029	2.04e-06	6.54e-05%
**L1578**	**False**	**ZB2**	1.18e-06	0.000108%	0.0	1	0.000519	3.12e-05	6.95e-05	3.3e-06	0.000106%
**OTU_6835**	**True**	**OD1**	1.18e-06	0.000107%	0.0	1	0.000154	0.0	1.65e-05	2.88e-06	9.21e-05%
**L1287**	**False**	**EW055**	1.18e-06	0.000107%	0.0	1	0.000402	0.0	0.0	2.52e-06	8.07e-05%
**OTU_5878**	**True**	**OD1**	1.17e-06	0.000107%	0.0	1	0.000176	6.41e-05	1.68e-05	5.48e-06	0.000176%
**OTU_6561**	**True**	**OD1**	1.17e-06	0.000107%	0.0	1	0.000106	2.25e-07	0.0	2.38e-06	7.62e-05%
**OTU_5804**	**True**	**OD1**	1.17e-06	0.000107%	0.0	1	0.00045	0.0	2.87e-06	2.54e-06	8.15e-05%
**L1577**	**False**	**ZB2**	1.17e-06	0.000106%	0.0	1	0.000907	0.000318	9.79e-05	4.07e-06	0.00013%
**OTU_500**	**True**	**PBS-25**	1.17e-06	0.000106%	0.0	1	0.000164	0.0	5.37e-06	2.58e-06	8.25e-05%
**L1033**	**False**	**koll11**	1.16e-06	0.000106%	0.0	1	0.000507	6.34e-06	0.000951	5.81e-06	0.000186%
**L1024**	**False**	**koll11**	1.16e-06	0.000106%	0.0	1	0.0023	0.000112	0.000288	1.43e-06	4.58e-05%
**L1465**	**False**	**ZB2**	1.15e-06	0.000104%	0.0	1	0.000297	0.00267	0.00103	6.24e-06	0.0002%
**L1347**	**False**	**ZB2**	1.15e-06	0.000104%	0.0	1	0.000632	8.69e-05	0.000177	3.21e-06	0.000103%
**OTU_300**	**True**	**koll11**	1.15e-06	0.000104%	0.0	1	0.0	0.000137	0.0	2.93e-06	9.38e-05%
**OTU_1795**	**True**	**koll11**	1.14e-06	0.000104%	0.0	1	0.000295	2.15e-05	1.77e-05	3.04e-06	9.73e-05%
**L1436**	**False**	**ZB2**	1.13e-06	0.000103%	0.0	1	0.00231	0.000376	0.000299	2.4e-06	7.68e-05%
**OTU_10774**	**True**	**OP11-4**	1.12e-06	0.000102%	0.0	1	0.000152	0.0	0.0	2.38e-06	7.63e-05%
**L1814**	**False**	**OD1**	1.12e-06	0.000102%	0.0	1	0.00106	8.7e-05	0.000146	2.51e-06	8.03e-05%
**L797**	**False**	**PBS-25**	1.12e-06	0.000102%	0.0	1	0.000599	5.18e-06	4.45e-06	1.29e-06	4.15e-05%
**L1398**	**False**	**ZB2**	1.12e-06	0.000102%	0.0	1	0.00178	0.00225	0.00106	3.32e-05	0.00106%
**OTU_4019**	**True**	**koll11**	1.12e-06	0.000102%	0.0	1	0.0	0.0	0.000131	2.66e-06	8.51e-05%
**L151**	**False**	**ABY1**	1.12e-06	0.000102%	0.0	1	0.000766	0.00032	4.28e-05	4.33e-06	0.000139%
**OTU_8644**	**True**	**ABY1**	1.11e-06	0.000101%	0.0	1	0.000504	0.000222	2.27e-05	3.74e-06	0.00012%
**OTU_965**	**True**	**PBS-25**	1.1e-06	0.0001%	0.0	1	0.0	4.08e-05	0.000147	4.76e-06	0.000152%
**OTU_1608**	**True**	**koll11**	1.1e-06	0.0001%	0.0	1	0.000251	1.06e-05	0.0	2.33e-06	7.48e-05%
**OTU_8324**	**True**	**ZB2**	1.1e-06	0.0001%	0.0	1	0.0	4.14e-06	8.7e-05	2.73e-06	8.74e-05%
**OTU_8787**	**True**	**ABY1**	1.1e-06	0.0001%	0.0	1	7.23e-05	0.000283	3.96e-05	4.7e-06	0.000151%
**OTU_266**	**True**	**koll11**	1.1e-06	0.0001%	0.0	1	0.000342	6.9e-06	2.25e-05	2.37e-06	7.6e-05%
**OTU_6989**	**True**	**ABY1**	1.1e-06	0.0001%	0.0	1	0.0001	6.2e-05	1.53e-05	8.73e-06	0.00028%
**OTU_2694**	**True**	**SJA-4**	1.09e-06	9.94e-05%	0.0	1	0.0	0.0	0.000153	3.32e-06	0.000106%
**OTU_10757**	**True**	**OP11-4**	1.09e-06	9.93e-05%	0.0	1	0.000342	7.25e-05	7.26e-05	3.92e-06	0.000126%
**OTU_8150**	**True**	**OP11-4**	1.09e-06	9.89e-05%	0.0	1	0.00011	0.0	9.17e-06	3.1e-06	9.93e-05%
**OTU_6312**	**True**	**ZB2**	1.08e-06	9.8e-05%	0.0	1	0.0012	0.000172	9.23e-05	2.65e-06	8.47e-05%
**OTU_384**	**True**	**PBS-25**	1.07e-06	9.75e-05%	0.0	1	0.000175	9.67e-05	7.42e-08	4.56e-06	0.000146%
**L880**	**False**	**koll11**	1.07e-06	9.72e-05%	0.0	1	0.0069	0.00143	0.0029	1.92e-06	6.16e-05%
**OTU_2628**	**True**	**SJA-4**	1.06e-06	9.7e-05%	0.0	1	0.0	0.0	0.000161	3.2e-06	0.000103%
**L691**	**False**	**koll11**	1.06e-06	9.68e-05%	0.0	1	0.00281	0.00056	0.00141	3.54e-06	0.000114%
**OTU_738**	**True**	**koll11**	1.06e-06	9.65e-05%	0.0	1	0.000172	6.02e-05	3.6e-06	5.24e-06	0.000168%
**L471**	**False**	**OP11-4**	1.06e-06	9.63e-05%	0.0	1	0.000133	3.05e-06	6.15e-06	2.26e-06	7.23e-05%
**L897**	**False**	**koll11**	1.05e-06	9.54e-05%	0.0	1	0.00186	0.000361	0.0011	4.48e-06	0.000144%
**L819**	**False**	**PBS-25**	1.05e-06	9.53e-05%	0.0	1	0.000498	0.00012	7.42e-08	2.36e-06	7.56e-05%

**L25**	**False**	**Bacteria**	1.05e-06	9.53e-05%	0.0	1	0.992	0.999	1.0	-5.24329645217e-07	-1.67960038543[illegible]05%
**OTU_388**	**True**	**koll11**	1.04e-06	9.52e-05%	0.0	1	0.000314	5.48e-05	0.0	1.94e-06	6.22e-05%
**L453**	**False**	**OP11-4**	1.04e-06	9.48e-05%	0.0	1	0.00113	4.99e-05	0.000759	5.65e-06	0.000181%
**L1341**	**False**	**ZB2**	1.04e-06	9.47e-05%	0.0	1	0.000409	0.000156	1.9e-05	3.73e-06	0.000119%
**L559**	**False**	**SJA-4**	1.04e-06	9.45e-05%	0.0	1	0.002	0.000119	0.000603	3.87e-06	0.000124%
**L888**	**False**	**koll11**	1.04e-06	9.44e-05%	0.0	1	0.000652	0.000216	0.000218	8.7e-06	0.000279%
**L1674**	**False**	**OD1**	1.03e-06	9.42e-05%	0.0	1	0.000362	8.38e-05	0.00039	1.23e-05	0.000393%
**OTU_5249**	**True**	**OD1**	1.03e-06	9.35e-05%	0.0	1	0.000421	0.000101	1.47e-05	2.94e-06	9.41e-05%
**L22**	**False**	**OD1**	1.02e-06	9.29e-05%	0.0	1	0.000259	7.24e-05	1.53e-05	2.95e-06	9.46e-05%
**OTU_4056**	**True**	**koll11**	1.01e-06	9.18e-05%	0.0	1	0.000487	2.66e-05	0.0	2.22e-06	7.11e-05%
**OTU_354**	**True**	**koll11**	1e-06	9.14e-05%	0.0	1	0.000364	0.0	1.66e-06	1.39e-06	4.46e-05%
**OTU_827**	**True**	**koll11**	9.96e-07	9.07e-05%	0.0	1	0.000203	0.0	0.0	2.65e-06	8.48e-05%
**L543**	**False**	**SJA-4**	9.93e-07	9.05e-05%	0.0	1	0.00178	0.0003	0.00219	8.33e-06	0.000267%
**L1395**	**False**	**ZB2**	9.86e-07	8.98e-05%	0.0	1	0.00275	0.000905	0.00112	8.96e-06	0.000287%
**OTU_433**	**True**	**PBS-25**	9.8e-07	8.93e-05%	0.0	1	0.000414	0.000104	0.000101	3.99e-06	0.000128%
**OTU_4832**	**True**	**ZB2**	9.8e-07	8.92e-05%	0.0	1	0.000167	4.07e-06	0.0	1.35e-06	4.32e-05%
**OTU_1558**	**True**	**PBS-25**	9.76e-07	8.89e-05%	0.0	1	0.000138	2.55e-05	0.0	3.13e-06	0.0001%
**OTU_5840**	**True**	**OD1**	9.74e-07	8.87e-05%	0.0	1	0.000198	0.000148	1.01e-05	5.73e-06	0.000184%
**L1868**	**False**	**OD1**	9.67e-07	8.81e-05%	0.0	1	0.00133	0.000146	0.000181	1.87e-06	6e-05%
**OTU_6217**	**True**	**ZB2**	9.64e-07	8.78e-05%	0.0	1	0.0	2.28e-05	0.000188	2.78e-06	8.9e-05%
**OTU_9001**	**True**	**ABY1**	9.58e-07	8.73e-05%	0.0	1	0.000131	4.8e-05	1.75e-05	5.14e-06	0.000165%
**OTU_3762**	**True**	**SJA-4**	9.58e-07	8.73e-05%	0.0	1	0.000215	0.0	0.0	1.77e-06	5.68e-05%
**L1366**	**False**	**ZB2**	9.53e-07	8.68e-05%	0.0	1	1.63e-06	0.00119	0.000341	1.69e-06	5.41e-05%
**OTU_365**	**True**	**koll11**	9.5e-07	8.66e-05%	0.0	1	0.000498	0.0	0.000122	3.43e-06	0.00011%
**L780**	**False**	**PBS-25**	9.48e-07	8.64e-05%	0.0	1	0.000658	3.65e-05	7.42e-08	1.38e-06	4.41e-05%
**L1881**	**False**	**OD1**	9.45e-07	8.61e-05%	0.0	1	0.000226	2.25e-07	0.0	1.46e-06	4.67e-05%
**OTU_6180**	**True**	**OD1**	9.4e-07	8.57e-05%	0.0	1	0.000366	0.000175	3.57e-06	3.31e-06	0.000106%
**OTU_8125**	**True**	**ABY1**	9.37e-07	8.53e-05%	0.0	1	0.000592	0.000328	7.2e-06	2.77e-06	8.88e-05%
**OTU_510**	**True**	**koll11**	9.36e-07	8.53e-05%	0.0	1	0.000393	2.95e-05	2.49e-05	2.12e-06	6.8e-05%
**OTU_6988**	**True**	**ZB2**	9.33e-07	8.5e-05%	0.0	1	0.00175	0.000562	0.000938	9.48e-06	0.000304%
**OTU_1444**	**True**	**koll11**	9.33e-07	8.49e-05%	0.0	1	0.000332	8.13e-06	0.000329	3.76e-06	0.000121%
**OTU_6349**	**True**	**WCHB1-64**	9.27e-07	8.45e-05%	0.0	1	0.000316	6.01e-05	8.13e-05	4.24e-06	0.000136%
**OTU_8743**	**True**	**OD1**	9.19e-07	8.37e-05%	0.0	1	0.000241	0.0	0.0	1.7e-06	5.46e-05%
**OTU_9710**	**True**	**ABY1**	9.14e-07	8.33e-05%	0.0	1	0.000394	0.000134	6.12e-05	4.37e-06	0.00014%
**OTU_3117**	**True**	**koll11**	9.11e-07	8.29e-05%	0.0	1	0.000305	8.32e-05	0.000176	9.27e-06	0.000297%
**OTU_10854**	**True**	**OP11-4**	9.1e-07	8.29e-05%	0.0	1	0.000179	0.0	0.0	2.2e-06	7.05e-05%
**L1138**	**False**	**koll11**	9.07e-07	8.27e-05%	0.0	1	0.000464	7.12e-06	2.25e-05	1.45e-06	4.64e-05%
**L1494**	**False**	**ZB2**	9.07e-07	8.26e-05%	0.0	1	0.000641	0.000137	2.15e-05	1.7e-06	5.44e-05%
**OTU_7216**	**True**	**ZB2**	9.06e-07	8.25e-05%	0.0	1	0.00024	0.0	0.0	1.69e-06	5.42e-05%
**OTU_8246**	**True**	**OD1**	8.99e-07	8.19e-05%	0.0	1	0.0024	0.000179	9.29e-05	1.18e-06	3.79e-05%
**OTU_353**	**True**	**koll11**	8.96e-07	8.16e-05%	0.0	1	0.000579	0.000144	0.000248	7.33e-06	0.000235%
**OTU_2636**	**True**	**koll11**	8.95e-07	8.16e-05%	0.0	1	0.000105	0.0	4.74e-05	3.44e-06	0.00011%
**OTU_8112**	**True**	**ABY1**	8.87e-07	8.08e-05%	0.0	1	0.00209	0.000889	0.000121	1.88e-06	6.01e-05%
**OTU_152**	**True**	**ZB2**	8.83e-07	8.04e-05%	0.0	1	0.000223	7.22e-06	6.95e-05	3.67e-06	0.000117%
**L1418**	**False**	**ZB2**	8.83e-07	8.04e-05%	0.0	1	0.000539	2.69e-05	0.000877	5.25e-06	0.000168%
**OTU_339**	**True**	**koll11**	8.81e-07	8.02e-05%	0.0	1	0.000692	9.41e-05	3.61e-05	3.68e-06	0.000118%
**L1217**	**False**	**koll11**	8.79e-07	8.01e-05%	0.0	1	0.000181	3.39e-05	0.000469	7.83e-06	0.000251%
**OTU_5241**	**True**	**Mb-NB09**	8.79e-07	8e-05%	0.0	1	0.0	0.000109	0.0	2.25e-06	7.21e-05%
**L446**	**False**	**OP11-4**	8.74e-07	7.96e-05%	0.0	1	0.000217	2.07e-05	0.000134	4.27e-06	0.000137%
**L883**	**False**	**koll11**	8.73e-07	7.95e-05%	0.0	1	0.00385	0.000732	0.000748	1.76e-06	5.65e-05%
**L629**	**False**	**PBS-25**	8.72e-07	7.94e-05%	0.0	1	0.00229	0.000111	9.04e-05	1.91e-06	6.13e-05%
**L870**	**False**	**koll11**	8.6e-07	7.84e-05%	0.0	1	0.000381	4.01e-05	1.57e-05	1.85e-06	5.92e-05%
**L1590**	**False**	**OD1**	8.58e-07	7.81e-05%	0.0	1	0.0117	0.00919	0.00796	1.59e-05	0.00051%
**L903**	**False**	**koll11**	8.51e-07	7.75e-05%	0.0	1	0.000919	0.000293	0.000736	8.74e-06	0.00028%
**L749**	**False**	**PBS-25**	8.5e-07	7.74e-05%	0.0	1	0.000585	7.28e-05	0.0	2.03e-06	6.49e-05%
**L1255**	**False**	**TM7-1**	8.47e-07	7.71e-05%	0.0	1	0.000134	0.0	0.0	2.05e-06	6.57e-05%
**OTU_6575**	**True**	**ZB2**	8.46e-07	7.71e-05%	0.0	1	0.00027	2.61e-05	5.44e-05	1.81e-06	5.79e-05%
**L1475**	**False**	**ZB2**	8.44e-07	7.69e-05%	0.0	1	0.000787	0.000628	0.000412	2.73e-05	0.000876%
**OTU_16**	**True**	**ZB2**	8.44e-07	7.69e-05%	0.0	1	5.97e-05	0.0	0.0	1.43e-06	4.59e-05%
**OTU_6533**	**True**	**OD1**	8.42e-07	7.67e-05%	0.0	1	0.0	0.000495	0.000773	3.56e-06	0.000114%
**L1224**	**False**	**koll11**	8.41e-07	7.66e-05%	0.0	1	0.000461	0.000197	0.000547	1.5e-05	0.000481%
**L401**	**False**	**ABY1**	8.38e-07	7.64e-05%	0.0	1	0.00805	0.00243	0.00106	2e-06	6.42e-05%
**OTU_1456**	**True**	**kpj58rc**	8.37e-07	7.63e-05%	0.0	1	9.58e-05	6.28e-06	0.0	1.85e-06	5.92e-05%
**OTU_8761**	**True**	**OD1**	8.36e-07	7.62e-05%	0.0	1	0.00015	2.93e-05	6.68e-07	2.01e-06	6.43e-05%

**OTU_10753**	**True**	**OP11-3**	8.35e-07	7.61e-05%	0.0	1	0.000188	7.81e-05	7.16e-05	1.14e-05	0.000365%
**OTU_7937**	**True**	**ABY1**	8.33e-07	7.59e-05%	0.0	1	0.000584	0.0	0.0	1.61e-06	5.15e-05%
**L865**	**False**	**koll11**	8.3e-07	7.56e-05%	0.0	1	0.000695	6.24e-05	0.000182	2.03e-06	6.49e-05%
**OTU_10851**	**True**	**OP11-4**	8.3e-07	7.56e-05%	0.0	1	0.000133	2.25e-07	0.0	1.38e-06	4.42e-05%
**OTU_619**	**True**	**koll11**	8.28e-07	7.54e-05%	0.0	1	0.000195	1.71e-05	0.0	1.88e-06	6.01e-05%
**OTU_6585**	**True**	**ZB2**	8.22e-07	7.49e-05%	0.0	1	0.000178	5.77e-05	0.000151	1.29e-05	0.000413%
**L1066**	**False**	**koll11**	8.2e-07	7.47e-05%	0.0	1	0.00153	5.52e-05	0.0002	1.72e-06	5.53e-05%
**L1254**	**False**	**TM7-1**	8.19e-07	7.46e-05%	0.0	1	0.000358	3.22e-06	3.68e-05	1.61e-06	5.17e-05%
**OTU_4507**	**True**	**koll11**	8.19e-07	7.46e-05%	0.0	1	0.00011	2.2e-06	0.0	1.9e-06	6.09e-05%
**OTU_6160**	**True**	**ZB2**	8.1e-07	7.38e-05%	0.0	1	4.05e-05	0.000773	0.000683	2.27e-06	7.26e-05%
**L1907**	**False**	**OD1**	8.06e-07	7.34e-05%	0.0	1	0.000881	5.34e-05	7.6e-06	1.1e-06	3.53e-05%
**OTU_6190**	**True**	**ZB2**	7.98e-07	7.27e-05%	0.0	1	0.000388	0.000287	2.84e-05	4.61e-06	0.000148%
**L316**	**False**	**ABY1**	7.95e-07	7.24e-05%	0.0	1	0.000188	0.000665	0.000271	1.09e-05	0.00035%
**OTU_7173**	**True**	**OD1**	7.91e-07	7.21e-05%	0.0	1	0.000128	3.21e-06	3.67e-06	1.59e-06	5.1e-05%
**OTU_588**	**True**	**koll11**	7.9e-07	7.2e-05%	0.0	1	7.71e-05	1.03e-05	0.0	1.88e-06	6.03e-05%
**L18**	**False**	**OD1**	7.88e-07	7.18e-05%	0.0	1	0.000315	4.16e-05	6.93e-06	1.38e-06	4.42e-05%
**OTU_3345**	**True**	**PBS-25**	7.86e-07	7.16e-05%	0.0	1	0.000204	0.0	2.99e-07	1.45e-06	4.66e-05%
**L560**	**False**	**SJA-4**	7.84e-07	7.15e-05%	0.0	1	0.000462	0.000119	2.18e-05	1.99e-06	6.39e-05%
**L1205**	**False**	**koll11**	7.81e-07	7.12e-05%	0.0	1	0.000168	1.26e-05	8.48e-05	2.33e-06	7.46e-05%
**OTU_421**	**True**	**koll11**	7.78e-07	7.09e-05%	0.0	1	0.00068	2.06e-05	6.05e-06	9.13e-07	2.93e-05%
**OTU_129**	**True**	**OD1**	7.69e-07	7.01e-05%	0.0	1	9.49e-06	8.42e-05	4.39e-05	1.03e-05	0.000331%
**OTU_602**	**True**	**koll11**	7.67e-07	6.99e-05%	0.0	1	0.00023	9.68e-05	2.5e-05	4.33e-06	0.000139%
**OTU_8390**	**True**	**ABY1**	7.67e-07	6.99e-05%	0.0	1	0.000431	2.83e-05	5.3e-06	1.95e-06	6.25e-05%
**OTU_5626**	**True**	**OD1**	7.63e-07	6.95e-05%	0.0	1	0.00012	0.0	0.0	1.4e-06	4.47e-05%
**OTU_1428**	**True**	**GIF10**	7.6e-07	6.93e-05%	0.0	1	0.000104	5.84e-06	1.83e-05	2.71e-06	8.69e-05%
**L1233**	**False**	**TM7**	7.59e-07	6.92e-05%	0.0	1	0.0118	0.0122	0.0104	0.000256	0.00819%
**OTU_2664**	**True**	**koll11**	7.56e-07	6.88e-05%	0.0	1	0.000108	2.32e-05	3.57e-06	2.67e-06	8.56e-05%
**OTU_282**	**True**	**koll11**	7.55e-07	6.88e-05%	0.0	1	0.000108	7.12e-06	3.8e-06	1.61e-06	5.14e-05%
**OTU_1061**	**True**	**PBS-25**	7.55e-07	6.87e-05%	0.0	1	9.77e-05	2.87e-05	0.0	3.62e-06	0.000116%
**OTU_9434**	**True**	**ABY1**	7.54e-07	6.87e-05%	0.0	1	0.00011	0.0	0.0	1.16e-06	3.72e-05%
**L3**	**False**	**Bacteria**	7.53e-07	6.86e-05%	0.0	1	0.997	1.0	1.0	-9.57466410978e-08	-3.06707997076e-06%
**OTU_9703**	**True**	**Mb-NB09**	7.48e-07	6.82e-05%	0.0	1	0.000148	0.0	0.0	1.78e-06	5.72e-05%
**L545**	**False**	**SJA-4**	7.46e-07	6.79e-05%	0.0	1	0.00044	0.00023	0.00198	3.78e-06	0.000121%
**OTU_6574**	**True**	**ZB2**	7.44e-07	6.78e-05%	0.0	1	0.00037	7.58e-05	2.15e-05	1.79e-06	5.72e-05%
**OTU_8350**	**True**	**ABY1**	7.37e-07	6.71e-05%	0.0	1	0.000102	0.0	0.0	1.2e-06	3.85e-05%
**L567**	**False**	**SJA-4**	7.34e-07	6.68e-05%	0.0	1	0.000851	4.42e-07	0.000581	3.7e-06	0.000119%
**L21**	**False**	**Bacteria**	7.32e-07	6.67e-05%	0.0	1	0.993	0.999	1.0	-3.71512318974e-07	-1.1900762046e-05%
**OTU_1550**	**True**	**PRR-12**	7.3e-07	6.65e-05%	0.0	1	0.000213	2.06e-05	0.0	1.71e-06	5.48e-05%
**OTU_10816**	**True**	**OP11-4**	7.3e-07	6.65e-05%	0.0	1	0.00018	0.000103	5.43e-06	3.59e-06	0.000115%
**OTU_211**	**True**	**koll11**	7.23e-07	6.59e-05%	0.0	1	6.15e-05	9.65e-05	0.0	5.08e-06	0.000163%
**OTU_4039**	**True**	**koll11**	7.16e-07	6.52e-05%	0.0	1	0.000127	5.51e-05	0.0	3.2e-06	0.000102%
**OTU_6076**	**True**	**SM2F11**	7.14e-07	6.51e-05%	0.0	1	9.84e-05	1.62e-05	0.0	2.02e-06	6.46e-05%
**L675**	**False**	**koll11**	7.1e-07	6.47e-05%	0.0	1	0.00202	0.000399	0.000909	3.61e-06	0.000116%
**OTU_8156**	**True**	**OP11-4**	7.09e-07	6.46e-05%	0.0	1	3.71e-05	3.85e-06	1.58e-05	3.83e-06	0.000123%
**OTU_5599**	**True**	**OD1**	7.07e-07	6.44e-05%	0.0	1	6.12e-05	0.000119	7.18e-06	5.01e-06	0.00016%
**OTU_4071**	**True**	**koll11**	7.01e-07	6.39e-05%	0.0	1	0.000398	2.4e-05	1.41e-05	1.39e-06	4.47e-05%
**OTU_8732**	**True**	**ABY1**	6.96e-07	6.34e-05%	0.0	1	0.000251	1.61e-05	9.53e-06	1.05e-06	3.35e-05%
**OTU_7016**	**True**	**ZB2**	6.95e-07	6.33e-05%	0.0	1	0.0	0.000136	2.78e-05	2.02e-06	6.46e-05%
**L591**	**False**	**PRR-12**	6.91e-07	6.29e-05%	0.0	1	0.00204	0.000192	0.000216	9.99e-07	3.2e-05%
**OTU_4177**	**True**	**koll11**	6.91e-07	6.29e-05%	0.0	1	0.000197	2.11e-05	2.86e-05	2.62e-06	8.4e-05%
**L1216**	**False**	**koll11**	6.85e-07	6.24e-05%	0.0	1	0.000844	0.000182	0.000539	6.08e-06	0.000195%
**L265**	**False**	**ABY1**	6.83e-07	6.22e-05%	0.0	1	0.00264	0.00191	0.00125	1.36e-05	0.000436%
**L650**	**False**	**koll11**	6.79e-07	6.18e-05%	0.0	1	0.000464	4.99e-05	4.5e-05	2.03e-06	6.5e-05%
**L1625**	**False**	**OD1**	6.76e-07	6.16e-05%	0.0	1	0.000243	3.78e-05	5.48e-05	2.68e-06	8.57e-05%
**OTU_19**	**True**	**ZB2**	6.75e-07	6.14e-05%	0.0	1	0.000149	9.68e-06	2.29e-05	2.12e-06	6.78e-05%
**OTU_1538**	**True**	**PBS-25**	6.7e-07	6.11e-05%	0.0	1	0.000155	7.28e-05	0.0	3.19e-06	0.000102%
**OTU_6199**	**True**	**ZB2**	6.64e-07	6.05e-05%	0.0	1	0.000379	0.0	0.0	1.35e-06	4.33e-05%
**OTU_8181**	**True**	**OP11-4**	6.59e-07	6.01e-05%	0.0	1	0.000123	6.88e-06	4.42e-06	1.01e-06	3.24e-05%
**OTU_8065**	**True**	**ABY1**	6.59e-07	6e-05%	0.0	1	3.68e-06	2.83e-05	0.0	2.64e-06	8.47e-05%
**L44**	**False**	**OD1**	6.56e-07	5.97e-05%	0.0	1	0.113	0.152	0.0937	9.18e-06	0.000294%
**OTU_5311**	**True**	**OD1**	6.52e-07	5.94e-05%	0.0	1	0.000302	5.81e-05	2.57e-05	1.86e-06	5.97e-05%
**OTU_2434**	**True**	**SBRH58**	6.51e-07	5.93e-05%	0.0	1	0.0	2.49e-05	0.0	1.63e-06	5.22e-05%
**OTU_4986**	**True**	**ZB2**	6.49e-07	5.91e-05%	0.0	1	0.000384	2.73e-05	7.3e-05	1.95e-06	6.24e-05%

**L179**	**False**	**SM2F11**	6.48e-07	5.9e-05%	0.0	1	0.000339	4.29e-05	3.03e-05	1.37e-06	4.4e-05%
**OTU_8811**	**True**	**TM7-1**	6.43e-07	5.86e-05%	0.0	1	0.000145	5.69e-05	0.000141	1.05e-05	0.000335%
**OTU_8045**	**True**	**OD1**	6.36e-07	5.79e-05%	0.0	1	0.000762	0.000285	0.000189	3.35e-06	0.000107%
**OTU_2442**	**True**	**koll11**	6.35e-07	5.79e-05%	0.0	1	0.000123	1.91e-05	0.0	2.34e-06	7.5e-05%
**OTU_1067**	**True**	**koll11**	6.3e-07	5.74e-05%	0.0	1	0.000292	6.24e-05	0.000168	5.13e-06	0.000164%
**OTU_10815**	**True**	**OP11-4**	6.25e-07	5.7e-05%	0.0	1	0.0	0.0	4.93e-05	1.91e-06	6.11e-05%
**OTU_912**	**True**	**BD4-9**	6.18e-07	5.63e-05%	0.0	1	0.000339	0.0	0.0	1.16e-06	3.7e-05%
**L1226**	**False**	**koll11**	6.17e-07	5.62e-05%	0.0	1	0.000234	0.000158	0.000547	8.84e-06	0.000283%
**OTU_7089**	**True**	**ABY1**	6.09e-07	5.55e-05%	0.0	1	0.000106	1.48e-05	8.98e-07	1.64e-06	5.27e-05%
**OTU_8638**	**True**	**ABY1**	6.07e-07	5.53e-05%	0.0	1	0.000174	0.0	0.0	1.13e-06	3.63e-05%
**OTU_402**	**True**	**PBS-25**	6.03e-07	5.49e-05%	0.0	1	0.0	0.0	0.000222	1.42e-06	4.54e-05%
**L671**	**False**	**koll11**	5.94e-07	5.41e-05%	0.0	1	0.000362	3.53e-05	2.86e-05	1.47e-06	4.71e-05%
**OTU_7976**	**True**	**ABY1**	5.91e-07	5.38e-05%	0.0	1	2.65e-05	0.000178	3.12e-05	4.09e-06	0.000131%
**OTU_5076**	**True**	**ZB2**	5.83e-07	5.31e-05%	0.0	1	0.000249	5.96e-05	0.000104	3.34e-06	0.000107%
**L885**	**False**	**koll11**	5.83e-07	5.31e-05%	0.0	1	0.00211	0.000556	0.000324	1.41e-06	4.53e-05%
**OTU_6316**	**True**	**ZB2**	5.8e-07	5.28e-05%	0.0	1	0.000539	2.69e-05	3.65e-05	1e-06	3.21e-05%
**OTU_4334**	**True**	**koll11**	5.78e-07	5.27e-05%	0.0	1	0.00123	0.000293	0.000568	3.18e-06	0.000102%
**L1053**	**False**	**koll11**	5.75e-07	5.24e-05%	0.0	1	0.00514	0.000513	0.000812	7.21e-07	2.31e-05%
**OTU_456**	**True**	**koll11**	5.72e-07	5.21e-05%	0.0	1	0.000273	4.33e-05	3.98e-05	1.75e-06	5.61e-05%
**OTU_4770**	**True**	**GIF10**	5.71e-07	5.2e-05%	0.0	1	0.000317	8.83e-05	0.000209	7.04e-06	0.000225%
**L1274**	**False**	**TM7**	5.71e-07	5.2e-05%	0.0	1	0.000101	6.43e-07	2.07e-05	1.52e-06	4.86e-05%
**L1081**	**False**	**koll11**	5.61e-07	5.11e-05%	0.0	1	0.000424	6.19e-05	4.2e-05	1.78e-06	5.71e-05%
**L541**	**False**	**SJA-4**	5.61e-07	5.11e-05%	0.0	1	0.00182	0.000324	0.0022	4.73e-06	0.000152%
**OTU_10860**	**True**	**OP11**	5.6e-07	5.1e-05%	0.0	1	0.000317	4.05e-05	1.05e-05	1e-06	3.21e-05%
**L290**	**False**	**ABY1**	5.58e-07	5.08e-05%	0.0	1	8.23e-05	0.0	1.7e-05	1.65e-06	5.27e-05%
**OTU_1982**	**True**	**koll11**	5.53e-07	5.04e-05%	0.0	1	0.000152	5.4e-05	1.8e-05	3.09e-06	9.9e-05%
**OTU_5818**	**True**	**OD1**	5.53e-07	5.03e-05%	0.0	1	0.00169	0.000109	0.00049	1.11e-06	3.54e-05%
**L901**	**False**	**koll11**	5.51e-07	5.02e-05%	0.0	1	0.00125	0.000301	0.00107	3.25e-06	0.000104%
**OTU_6059**	**True**	**ABY1**	5.48e-07	4.99e-05%	0.0	1	0.0	4.27e-05	7.9e-06	3.03e-06	9.71e-05%
**OTU_305**	**True**	**PBS-25**	5.42e-07	4.94e-05%	0.0	1	0.000194	1.04e-05	0.000347	2.75e-06	8.82e-05%
**OTU_4272**	**True**	**koll11**	5.38e-07	4.9e-05%	0.0	1	0.00046	0.000183	2.4e-05	2.17e-06	6.95e-05%
**OTU_9183**	**True**	**ABY1**	5.34e-07	4.87e-05%	0.0	1	0.000391	8.44e-05	3.12e-05	1.94e-06	6.21e-05%
**OTU_1725**	**True**	**koll11**	5.24e-07	4.77e-05%	0.0	1	4.99e-05	0.0	0.0	1.15e-06	3.67e-05%
**OTU_6645**	**True**	**ZB2**	5.2e-07	4.73e-05%	0.0	1	0.000183	2.92e-05	4.64e-05	2.1e-06	6.73e-05%
**OTU_880**	**True**	**koll11**	5.05e-07	4.6e-05%	0.0	1	8.47e-05	0.0	0.0	1.3e-06	4.16e-05%
**OTU_7954**	**True**	**OD1**	4.84e-07	4.41e-05%	0.0	1	0.000733	0.000116	5.95e-05	1.53e-06	4.89e-05%
**L1252**	**False**	**TM7-1**	4.82e-07	4.39e-05%	0.0	1	0.000702	9.18e-05	5.98e-05	1.08e-06	3.45e-05%
**OTU_603**	**True**	**koll11**	4.78e-07	4.36e-05%	0.0	1	0.00011	5.17e-06	0.0	1.13e-06	3.62e-05%
**OTU_5955**	**True**	**OD1**	4.78e-07	4.35e-05%	0.0	1	0.000141	4.78e-05	1.31e-05	2.22e-06	7.1e-05%
**L1154**	**False**	**koll11**	4.74e-07	4.32e-05%	0.0	1	0.00288	0.000648	0.00211	3.53e-06	0.000113%
**OTU_3798**	**True**	**PBS-25**	4.72e-07	4.3e-05%	0.0	1	0.000126	0.0	0.0	1.02e-06	3.27e-05%
**OTU_718**	**True**	**PBS-25**	4.7e-07	4.28e-05%	0.0	1	0.000578	0.000137	3.47e-05	1.25e-06	4.01e-05%
**L517**	**False**	**OP11-3**	4.66e-07	4.25e-05%	0.0	1	0.000188	7.81e-05	7.16e-05	6.36e-06	0.000204%
**OTU_5444**	**True**	**OD1**	4.64e-07	4.23e-05%	0.0	1	7.19e-05	2.42e-05	2.9e-06	2.02e-06	6.46e-05%
**L637**	**False**	**PBS-25**	4.58e-07	4.17e-05%	0.0	1	0.000254	1.53e-05	5.22e-06	1.16e-06	3.71e-05%
**L1755**	**False**	**OD1**	4.55e-07	4.15e-05%	0.0	1	0.000542	0.000495	0.00104	1.33e-05	0.000425%
**L755**	**False**	**PBS-25**	4.51e-07	4.11e-05%	0.0	1	0.00139	0.000149	9.12e-05	9.57e-07	3.07e-05%
**OTU_9132**	**True**	**TM7**	4.46e-07	4.06e-05%	0.0	1	3.1e-05	0.0	0.0	8.95e-07	2.87e-05%
**L891**	**False**	**koll11**	4.44e-07	4.04e-05%	0.0	1	0.000433	0.000127	0.0	1.41e-06	4.51e-05%
**OTU_7964**	**True**	**ABY1**	4.43e-07	4.03e-05%	0.0	1	8.82e-05	0.0	0.0	1.03e-06	3.31e-05%
**L1477**	**False**	**ZB2**	4.41e-07	4.01e-05%	0.0	1	0.000787	0.000492	0.000384	1.06e-05	0.000341%
**OTU_875**	**True**	**koll11**	4.37e-07	3.98e-05%	0.0	1	0.000122	2.25e-07	0.0	8.82e-07	2.82e-05%
**OTU_4170**	**True**	**koll11**	4.35e-07	3.96e-05%	0.0	1	3.05e-05	0.0	0.0	8.71e-07	2.79e-05%
**OTU_1058**	**True**	**koll11**	4.35e-07	3.96e-05%	0.0	1	0.000624	0.000271	0.000719	7.26e-06	0.000233%
**OTU_8179**	**True**	**EW055**	4.34e-07	3.95e-05%	0.0	1	0.000398	0.0	0.0	9.29e-07	2.98e-05%
**L36**	**False**	**ABY1**	4.32e-07	3.94e-05%	0.0	1	0.000351	1.93e-05	7.42e-08	6.2e-07	1.99e-05%
**OTU_6006**	**True**	**OD1**	4.24e-07	3.86e-05%	0.0	1	2.14e-05	0.0	0.0	8.45e-07	2.71e-05%
**OTU_10759**	**True**	**OP11-4**	4.23e-07	3.86e-05%	0.0	1	0.000492	0.000118	1.94e-05	7.57e-07	2.42e-05%
**L288**	**False**	**ABY1**	4.2e-07	3.82e-05%	0.0	1	0.000334	1.57e-05	1.7e-05	8.15e-07	2.61e-05%
**L221**	**False**	**ABY1**	4.17e-07	3.8e-05%	0.0	1	0.00124	0.0022	0.00146	4.66e-05	0.00149%
**OTU_9588**	**True**	**ABY1**	4.16e-07	3.79e-05%	0.0	1	7.25e-05	0.0	1.7e-05	1.45e-06	4.64e-05%
**OTU_6019**	**True**	**ABY1**	4.15e-07	3.78e-05%	0.0	1	0.000117	3.97e-05	4.61e-05	4.33e-06	0.000139%
**OTU_675**	**True**	**PBS-25**	4.15e-07	3.78e-05%	0.0	1	0.000621	5.04e-05	7.59e-05	8.96e-07	2.87e-05%
**OTU_287**	**True**	**koll11**	4.12e-07	3.76e-05%	0.0	1	0.000272	5.18e-05	1.6e-05	1.15e-06	3.69e-05%

**OTU_1319**	**True**	**koll11**	4.04e-07	3.68e-05%	0.0	1	0.000217	3.28e-05	3.43e-05	1.68e-06	5.39e-05%
**OTU_9084**	**True**	**TM7-1**	4.04e-07	3.68e-05%	0.0	1	7.01e-05	6.43e-07	2.07e-05	1.54e-06	4.95e-05%
**OTU_5953**	**True**	**OD1**	4.02e-07	3.66e-05%	0.0	1	0.000418	0.0	9.24e-05	9.66e-07	3.09e-05%
**OTU_277**	**True**	**koll11**	3.95e-07	3.6e-05%	0.0	1	0.000364	3.25e-06	0.000121	9.25e-07	2.96e-05%
**OTU_4088**	**True**	**koll11**	3.95e-07	3.59e-05%	0.0	1	0.000201	4.99e-05	4.5e-05	2.74e-06	8.79e-05%
**L1489**	**False**	**ZB2**	3.91e-07	3.56e-05%	0.0	1	0.00044	6.5e-05	0.000116	1.19e-06	3.8e-05%
**OTU_424**	**True**	**PBS-25**	3.9e-07	3.56e-05%	0.0	1	0.000136	0.000204	1.48e-07	2.06e-06	6.58e-05%
**L27**	**False**	**Bacteria**	3.85e-07	3.51e-05%	0.0	1	0.991	0.999	1.0	-2.8633961136e-07	-9.17239995855[illegible] 06%
**OTU_6157**	**True**	**ZB2**	3.83e-07	3.49e-05%	0.0	1	0.000369	9.9e-05	0.000107	1.88e-06	6.03e-05%
**OTU_6099**	**True**	**OD1**	3.81e-07	3.47e-05%	0.0	1	0.000265	3.34e-05	5.7e-06	1.37e-06	4.39e-05%
**OTU_397**	**True**	**koll11**	3.79e-07	3.46e-05%	0.0	1	0.000227	0.000163	3.43e-05	3.07e-06	9.83e-05%
**L617**	**False**	**BD4-9**	3.67e-07	3.34e-05%	0.0	1	0.000119	1.39e-05	0.0	9.42e-07	3.02e-05%
**OTU_3150**	**True**	**TM6**	3.65e-07	3.32e-05%	0.0	1	0.000189	3.94e-05	3.98e-06	1.05e-06	3.37e-05%
**OTU_6251**	**True**	**ZB2**	3.57e-07	3.25e-05%	0.0	1	0.000201	4.55e-05	3.16e-05	1.74e-06	5.56e-05%
**OTU_8669**	**True**	**EW055**	3.54e-07	3.23e-05%	0.0	1	0.000152	0.0	6.28e-05	1.57e-06	5.03e-05%
**OTU_7113**	**True**	**ABY1**	3.47e-07	3.16e-05%	0.0	1	3.77e-05	3.46e-05	0.0	1.06e-06	3.4e-05%
**OTU_7939**	**True**	**OD1**	3.47e-07	3.16e-05%	0.0	1	8.07e-05	2.74e-05	6.75e-06	1.49e-06	4.77e-05%
**OTU_5435**	**True**	**OD1**	3.47e-07	3.16e-05%	0.0	1	0.000205	0.000154	5.12e-05	4.36e-06	0.00014%
**L11**	**False**	**Bacteria**	3.46e-07	3.15e-05%	0.0	1	0.994	0.999	1.0	-7.75927753182e-08	-2.48555191415[illegible] 06%
**OTU_5883**	**True**	**OD1**	3.4e-07	3.1e-05%	0.0	1	0.000135	1.04e-06	1.96e-05	8.76e-07	2.81e-05%
**L804**	**False**	**PBS-25**	3.38e-07	3.08e-05%	0.0	1	1.66e-05	2.25e-07	0.000366	9.72e-07	3.11e-05%
**OTU_2708**	**True**	**koll11**	3.34e-07	3.04e-05%	0.0	1	0.000318	8.57e-05	9.3e-05	2.11e-06	6.77e-05%
**L570**	**False**	**SJA-4**	3.34e-07	3.04e-05%	0.0	1	0.000167	1.47e-05	0.000161	3.06e-06	9.81e-05%
**OTU_528**	**True**	**koll11**	3.33e-07	3.03e-05%	0.0	1	0.000113	1.59e-05	2.36e-05	1.6e-06	5.13e-05%
**OTU_6066**	**True**	**OD1**	3.3e-07	3.01e-05%	0.0	1	2.2e-05	0.000136	2.01e-05	2.76e-06	8.85e-05%
**OTU_4179**	**True**	**koll11**	3.3e-07	3.01e-05%	0.0	1	0.00087	5.93e-05	0.000374	1.36e-06	4.35e-05%
**OTU_9719**	**True**	**Mb-NB09**	3.29e-07	3e-05%	0.0	1	0.000102	0.0	7.5e-06	1.08e-06	3.46e-05%
**OTU_6920**	**True**	**OD1**	3.29e-07	2.99e-05%	0.0	1	0.000128	0.0	0.0	5.99e-07	1.92e-05%
**OTU_1096**	**True**	**koll11**	3.28e-07	2.99e-05%	0.0	1	0.000126	1.16e-05	2.58e-05	7.54e-07	2.41e-05%
**OTU_928**	**True**	**koll11**	3.25e-07	2.96e-05%	0.0	1	0.0	0.000117	0.0	1.05e-06	3.36e-05%
**OTU_8795**	**True**	**Mb-NB09**	3.21e-07	2.92e-05%	0.0	1	0.000184	5.77e-05	0.000165	3.26e-06	0.000104%
**OTU_8641**	**True**	**ABY1**	3.12e-07	2.84e-05%	0.0	1	0.000105	0.0	0.0	6.39e-07	2.05e-05%
**OTU_8162**	**True**	**ABY1**	3.04e-07	2.77e-05%	0.0	1	0.0	2.25e-07	2.47e-05	7.3e-07	2.34e-05%
**OTU_468**	**True**	**koll11**	3.01e-07	2.74e-05%	0.0	1	5.52e-05	3.5e-05	1.18e-05	3.39e-06	0.000109%
**OTU_7852**	**True**	**ZB2**	3.01e-07	2.74e-05%	0.0	1	5.52e-05	0.0	0.0	8.08e-07	2.59e-05%
**L219**	**False**	**ABY1**	3.01e-07	2.74e-05%	0.0	1	0.00167	0.00223	0.00149	5.92e-05	0.0019%
**OTU_485**	**True**	**koll11**	2.96e-07	2.7e-05%	0.0	1	0.000141	0.0	9.92e-07	5.6e-07	1.79e-05%
**OTU_9063**	**True**	**Mb-NB09**	2.93e-07	2.67e-05%	0.0	1	0.000269	7.65e-06	0.0	4.79e-07	1.54e-05%
**OTU_2288**	**True**	**PBS-25**	2.9e-07	2.64e-05%	0.0	1	5.75e-05	1.3e-05	0.0	8.56e-07	2.74e-05%
**L1156**	**False**	**koll11**	2.87e-07	2.61e-05%	0.0	1	0.00278	0.000611	0.00211	2.2e-06	7.05e-05%
**OTU_8023**	**True**	**ABY1**	2.83e-07	2.58e-05%	0.0	1	1.31e-05	3.17e-05	0.0	1.78e-06	5.71e-05%
**L869**	**False**	**koll11**	2.81e-07	2.56e-05%	0.0	1	0.000863	4.52e-05	1.57e-05	4.15e-07	1.33e-05%
**L1211**	**False**	**GIF10**	2.78e-07	2.54e-05%	0.0	1	0.0002	1.21e-05	1.83e-05	6.37e-07	2.04e-05%
**OTU_5486**	**True**	**OD1**	2.76e-07	2.52e-05%	0.0	1	0.000165	0.0	0.0	5.63e-07	1.8e-05%
**L515**	**False**	**OP11-3**	2.65e-07	2.41e-05%	0.0	1	0.000195	8.71e-05	7.23e-05	3.55e-06	0.000114%
**OTU_6642**	**True**	**ZB2**	2.64e-07	2.41e-05%	0.0	1	0.000197	1.71e-05	0.0	5.56e-07	1.78e-05%
**OTU_427**	**True**	**koll11**	2.6e-07	2.37e-05%	0.0	1	9.66e-05	3.7e-05	0.0	1.05e-06	3.35e-05%
**L679**	**False**	**koll11**	2.6e-07	2.37e-05%	0.0	1	0.000824	0.000146	0.000842	2.23e-06	7.15e-05%
**OTU_1496**	**True**	**koll11**	2.58e-07	2.35e-05%	0.0	1	3.65e-05	8.83e-05	0.0	1.24e-06	3.97e-05%
**L1030**	**False**	**koll11**	2.48e-07	2.26e-05%	0.0	1	0.00105	0.000158	0.000976	1.75e-06	5.6e-05%
**OTU_8328**	**True**	**OD1**	2.48e-07	2.26e-05%	0.0	1	0.000134	3.11e-05	6.86e-05	2.05e-06	6.55e-05%
**OTU_5270**	**True**	**OD1**	2.44e-07	2.22e-05%	0.0	1	0.000142	2.63e-05	1.72e-05	1.31e-06	4.19e-05%
**OTU_6085**	**True**	**ZB2**	2.4e-07	2.18e-05%	0.0	1	0.000188	0.0	4e-06	5.16e-07	1.65e-05%
**OTU_3602**	**True**	**PBS-25**	2.33e-07	2.12e-05%	0.0	1	0.000111	1.31e-05	3.53e-05	7.32e-07	2.35e-05%
**OTU_1514**	**True**	**koll11**	2.3e-07	2.1e-05%	0.0	1	9.16e-05	2.49e-05	0.0	8.15e-07	2.61e-05%
**OTU_6516**	**True**	**OD1**	2.26e-07	2.06e-05%	0.0	1	8.44e-05	4.78e-05	1.68e-05	2.46e-06	7.87e-05%
**L448**	**False**	**OP11-4**	2.24e-07	2.04e-05%	0.0	1	0.000133	7.96e-06	0.000113	1.14e-06	3.65e-05%
**OTU_1439**	**True**	**koll11**	2.19e-07	1.99e-05%	0.0	1	0.000121	3.6e-05	0.0	7.45e-07	2.39e-05%
**OTU_9194**	**True**	**ABY1**	2.17e-07	1.98e-05%	0.0	1	6.26e-05	0.0	1.9e-05	8.12e-07	2.6e-05%
**OTU_2845**	**True**	**PBS-25**	2.14e-07	1.95e-05%	0.0	1	0.000426	0.000232	0.000135	1.99e-06	6.38e-05%
**OTU_3297**	**True**	**PBS-25**	2.13e-07	1.94e-05%	0.0	1	0.000388	0.000113	0.00028	1.54e-06	4.94e-05%
**OTU_8041**	**True**	**TM7-3**	2.09e-07	1.9e-05%	0.0	1	6.68e-05	0.0	0.0	4.77e-07	1.53e-05%
**OTU_1626**	**True**	**PBS-25**	2.06e-07	1.87e-05%	0.0	1	0.000229	0.0	0.0	3.45e-07	1.1e-05%

**L668**	**False**	**koll11**	2.04e-07	1.86e-05%	0.0	1	0.000243	5.27e-05	0.000162	1.65e-06	5.28e-05%
**OTU_7059**	**True**	**ZB2**	2.02e-07	1.84e-05%	0.0	1	7.22e-05	0.000151	6.94e-06	1.05e-06	3.36e-05%
**OTU_2801**	**True**	**PBS-25**	2e-07	1.82e-05%	0.0	1	1.6e-05	3.87e-05	7.42e-08	1.17e-06	3.75e-05%
**OTU_1305**	**True**	**TM6**	1.97e-07	1.79e-05%	0.0	1	2.12e-06	8.05e-05	0.000167	8.46e-07	2.71e-05%
**L1439**	**False**	**ZB2**	1.94e-07	1.76e-05%	0.0	1	0.000353	0.000102	0.000118	1.37e-06	4.37e-05%
**OTU_2611**	**True**	**SJA-4**	1.9e-07	1.73e-05%	0.0	1	7.43e-05	0.000119	2.99e-07	8.1e-07	2.6e-05%
**L217**	**False**	**ABY1**	1.89e-07	1.73e-05%	0.0	1	0.00183	0.00227	0.00149	2.52e-05	0.000806%
**L1640**	**False**	**ZB2**	1.87e-07	1.71e-05%	0.0	1	7.3e-06	0.0	1.48e-07	5.06e-07	1.62e-05%
**OTU_8089**	**True**	**ABY1**	1.86e-07	1.7e-05%	0.0	1	0.0	5.01e-05	6.65e-05	1.17e-06	3.76e-05%
**OTU_136**	**True**	**OD1**	1.84e-07	1.67e-05%	0.0	1	4.41e-05	3.67e-05	0.0	9.17e-07	2.94e-05%
**OTU_643**	**True**	**koll11**	1.81e-07	1.65e-05%	0.0	1	0.00028	0.000237	2.07e-05	1.1e-06	3.52e-05%
**OTU_10804**	**True**	**OP11-4**	1.81e-07	1.65e-05%	0.0	1	0.000387	1.28e-05	0.000102	5.04e-07	1.61e-05%
**L531**	**False**	**TM6**	1.72e-07	1.57e-05%	0.0	1	0.000191	0.00012	0.000171	7.72e-06	0.000247%
**OTU_8167**	**True**	**ZB2**	1.72e-07	1.57e-05%	0.0	1	0.000164	3.65e-05	0.000225	1.38e-06	4.41e-05%
**OTU_6580**	**True**	**ZB2**	1.7e-07	1.55e-05%	0.0	1	2.1e-05	7.22e-05	0.000102	3.34e-06	0.000107%
**OTU_878**	**True**	**koll11**	1.65e-07	1.51e-05%	0.0	1	0.000104	8e-06	1.64e-05	6.01e-07	1.92e-05%
**L965**	**False**	**koll11**	1.58e-07	1.44e-05%	0.0	1	0.000607	0.000328	9.89e-05	9.81e-07	3.14e-05%
**OTU_10738**	**True**	**OP11-3**	1.56e-07	1.42e-05%	0.003	0	7.2e-06	8.98e-06	6.73e-07	1.42e-06	4.54e-05%
**OTU_7092**	**True**	**OD1**	1.53e-07	1.39e-05%	0.0	1	3.94e-05	4.44e-05	1.05e-05	2.28e-06	7.29e-05%
**OTU_1331**	**True**	**koll11**	1.43e-07	1.3e-05%	0.0	1	4.22e-05	1.02e-06	5.9e-05	6.23e-07	2e-05%
**OTU_5359**	**True**	**Mb-NB09**	1.42e-07	1.29e-05%	0.0	1	8.11e-05	9.71e-07	3.6e-06	3.83e-07	1.23e-05%
**OTU_6684**	**True**	**ZB2**	1.41e-07	1.28e-05%	0.0	1	0.00017	3.89e-05	6.14e-05	9.64e-07	3.09e-05%
**OTU_4389**	**True**	**koll11**	1.37e-07	1.25e-05%	0.0	1	0.000714	0.000144	0.000842	1.33e-06	4.25e-05%
**OTU_459**	**True**	**koll11**	1.37e-07	1.25e-05%	0.0	1	0.000545	0.000232	9.89e-05	8.08e-07	2.59e-05%
**OTU_1383**	**True**	**koll11**	1.25e-07	1.14e-05%	0.0	1	0.0	2.06e-05	0.0	3.19e-07	1.02e-05%
**OTU_6231**	**True**	**ZB2**	1.22e-07	1.12e-05%	0.0	1	3.9e-06	2.73e-05	1.3e-06	5.95e-07	1.91e-05%
**OTU_8017**	**True**	**ABY1**	1.22e-07	1.11e-05%	0.0	1	0.0	5.88e-05	2.7e-05	2.27e-07	7.28e-06%
**OTU_4502**	**True**	**koll11**	1.21e-07	1.11e-05%	0.0	1	0.000198	2.25e-07	0.0	2.6e-07	8.34e-06%
**L1032**	**False**	**koll11**	1.21e-07	1.1e-05%	0.0	1	0.000737	0.000103	0.000976	8.79e-07	2.82e-05%
**OTU_1646**	**True**	**PBS-25**	1.19e-07	1.08e-05%	0.0	1	0.000158	2.26e-05	0.000107	1.1e-06	3.52e-05%
**OTU_9018**	**True**	**Mb-NB09**	1.17e-07	1.07e-05%	0.0	1	7.7e-05	2.11e-05	4.57e-05	1.5e-06	4.8e-05%
**OTU_2558**	**True**	**SJA-4**	1.15e-07	1.05e-05%	0.0	1	4.21e-05	2.43e-05	1.53e-05	2.4e-06	7.68e-05%
**OTU_8234**	**True**	**ABY1**	1.11e-07	1.01e-05%	0.0	1	7.93e-05	7.62e-05	2.31e-05	1.92e-06	6.15e-05%
**OTU_5924**	**True**	**OD1**	1.08e-07	9.87e-06%	0.0	1	0.000122	5.02e-05	3.71e-07	3.84e-07	1.23e-05%
**L898**	**False**	**koll11**	1.03e-07	9.38e-06%	0.0	1	0.000611	5.95e-05	3.43e-05	3.22e-07	1.03e-05%
**OTU_1374**	**True**	**PBS-25**	9.97e-08	9.08e-06%	0.0	1	5.49e-05	9.98e-06	0.0	3.18e-07	1.02e-05%
**OTU_4774**	**True**	**koll11**	9.89e-08	9.01e-06%	0.0	1	0.000232	0.000146	0.000153	4.44e-06	0.000142%
**OTU_1117**	**True**	**BD4-9**	9.88e-08	9e-06%	0.0	1	0.0	1.39e-05	0.0	2.54e-07	8.13e-06%
**L1619**	**False**	**OD1**	9.73e-08	8.87e-06%	0.0	1	0.00229	0.00153	0.000928	1.1e-06	3.52e-05%
**OTU_3007**	**True**	**koll11**	9.71e-08	8.84e-06%	0.0	1	0.000138	5.27e-05	0.000114	1.78e-06	5.7e-05%
**OTU_9148**	**True**	**ABY1**	9.64e-08	8.78e-06%	0.0	1	1.07e-05	0.0	0.0	2.46e-07	7.89e-06%
**OTU_8738**	**True**	**OD1**	9.43e-08	8.59e-06%	0.0	1	0.0	1.67e-05	0.0	3.17e-07	1.02e-05%
**OTU_9129**	**True**	**Mb-NB09**	9.32e-08	8.49e-06%	0.0	1	4.43e-05	2.19e-05	7.01e-07	4.62e-07	1.48e-05%
**OTU_460**	**True**	**koll11**	8.87e-08	8.08e-06%	0.0	1	2.23e-05	0.0	0.0	2.22e-07	7.11e-06%
**OTU_1344**	**True**	**PBS-25**	8.7e-08	7.92e-06%	0.0	1	4.08e-05	0.0	2.07e-05	3.73e-07	1.19e-05%
**OTU_8890**	**True**	**ABY1**	8.48e-08	7.73e-06%	0.0	1	0.0	6.97e-06	0.0	2.18e-07	7e-06%
**OTU_6282**	**True**	**OD1**	8.47e-08	7.72e-06%	0.004	0	0.0	4.89e-06	0.0	1.94e-07	6.2e-06%
**OTU_9088**	**True**	**ABY1**	8.32e-08	7.58e-06%	0.0	1	9.8e-06	0.0	0.0	1.67e-07	5.34e-06%
**OTU_6617**	**True**	**ZB2**	8.21e-08	7.48e-06%	0.0	1	0.0	3.73e-05	4.16e-05	4.47e-07	1.43e-05%
**OTU_8154**	**True**	**OP11-4**	8.07e-08	7.35e-06%	0.0	1	8.47e-05	1.28e-05	2.06e-05	4.4e-07	1.41e-05%
**OTU_9100**	**True**	**TM7**	8.05e-08	7.33e-06%	0.0	1	5.47e-06	0.0	0.0	2.01e-07	6.43e-06%
**OTU_386**	**True**	**PBS-25**	7.66e-08	6.97e-06%	0.0	1	4.21e-05	0.0	4.24e-06	2.63e-07	8.41e-06%
**L223**	**False**	**ABY1**	7.16e-08	6.52e-06%	0.0	1	0.00122	0.00136	0.00103	3.08e-05	0.000986%
**L1159**	**False**	**koll11**	6.75e-08	6.15e-06%	0.0	1	0.000254	1.59e-05	2.46e-05	1.58e-07	5.05e-06%
**OTU_2809**	**True**	**SJA-4**	6.7e-08	6.1e-06%	0.002	0	0.0	5.88e-06	0.0	1.94e-07	6.21e-06%
**L1235**	**False**	**TM7**	6.69e-08	6.1e-06%	0.0	1	0.0113	0.0122	0.0104	0.000146	0.00466%
**OTU_3013**	**True**	**PBS-25**	6.49e-08	5.92e-06%	0.0	1	1.66e-05	2.25e-07	0.0	1.48e-07	4.76e-06%
**OTU_8165**	**True**	**ABY1**	6.47e-08	5.9e-06%	0.0	1	3.75e-05	1.34e-05	3.14e-05	1.46e-06	4.68e-05%
**OTU_4497**	**True**	**koll11**	6.13e-08	5.58e-06%	0.0	1	0.000165	1.42e-05	0.0	1.67e-07	5.34e-06%
**OTU_1883**	**True**	**koll11**	6e-08	5.47e-06%	0.0	1	0.000162	1.88e-05	1.07e-05	1.87e-07	5.98e-06%
**OTU_4959**	**True**	**ZB2**	5.93e-08	5.4e-06%	0.0	1	0.0	1.41e-05	9.8e-06	5.22e-07	1.67e-05%
**OTU_8032**	**True**	**ABY1**	5.68e-08	5.18e-06%	0.0	1	0.0	1.45e-05	0.0	1.44e-07	4.61e-06%
**OTU_4864**	**True**	**GIF10**	5.26e-08	4.8e-06%	0.0	1	0.000107	9.66e-06	0.0	1.24e-07	3.99e-06%
**OTU_831**	**True**	**koll11**	5.09e-08	4.63e-06%	0.0	1	0.00031	3.66e-05	0.0	1.2e-07	3.84e-06%
**L1362**	**False**	**OD1**	4.97e-08	4.53e-06%	0.0	1	0.0826	0.739	0.774	5.56e-09	1.78e-07%

**L1377**	**False**	**OD1**	4.85e-08	4.42e-06%	0.0	1	0.015	0.599	0.456	1.62e-08	5.18e-07%
**OTU_8159**	**True**	**ABY1**	4.66e-08	4.24e-06%	0.00701	0	0.0	4.07e-06	6.93e-06	3.18e-07	1.02e-05%
**OTU_464**	**True**	**koll11**	4.49e-08	4.09e-06%	0.002	0	5.34e-06	7.1e-06	0.0	3.23e-07	1.03e-05%
**OTU_1326**	**True**	**PBS-25**	4.48e-08	4.08e-06%	0.0	1	6.18e-06	0.0	0.0	8.81e-08	2.82e-06%
**OTU_6696**	**True**	**ZB2**	4.28e-08	3.9e-06%	0.0	1	7.3e-06	0.0	0.0	1.07e-07	3.43e-06%
**OTU_8175**	**True**	**TM7-3**	4.22e-08	3.85e-06%	0.0	1	1.75e-05	0.0	0.0	7.35e-08	2.35e-06%
**L1364**	**False**	**OD1**	4.18e-08	3.81e-06%	0.0	1	0.0701	0.688	0.58	9.96e-09	3.19e-07%
**OTU_10899**	**True**	**OP11-4**	4.07e-08	3.7e-06%	0.0	1	6.01e-06	2.05e-06	0.0	2.05e-07	6.57e-06%
**OTU_8084**	**True**	**ABY1**	3.89e-08	3.54e-06%	0.0	1	0.000142	0.00011	0.000211	1.83e-06	5.85e-05%
**OTU_10835**	**True**	**OP11-4**	3.76e-08	3.43e-06%	0.0541	0	4.73e-07	2.82e-06	6.15e-06	3.94e-07	1.26e-05%
**OTU_9123**	**True**	**ABY1**	3.6e-08	3.28e-06%	0.001	0	0.0	1.02e-06	7.13e-06	1.78e-07	5.69e-06%
**OTU_8186**	**True**	**OD1**	3.06e-08	2.78e-06%	0.0	1	0.0	0.0	3.57e-06	9.8e-08	3.14e-06%
**OTU_8172**	**True**	**EW055**	2.76e-08	2.51e-06%	0.0	1	4.25e-06	0.0	0.0	7.26e-08	2.33e-06%
**L529**	**False**	**TM6**	2.72e-08	2.48e-06%	0.0	1	0.000191	0.000145	0.000171	2.49e-06	7.98e-05%
**OTU_8169**	**True**	**TM7-3**	2.46e-08	2.24e-06%	0.0	1	4.01e-06	0.0	0.0	5.23e-08	1.67e-06%
**OTU_4166**	**True**	**koll11**	2.4e-08	2.19e-06%	0.0	1	5.44e-06	0.0	0.0	6.32e-08	2.03e-06%
**OTU_4996**	**True**	**GIF10**	1.97e-08	1.8e-06%	0.0	1	1.51e-06	0.0	0.0	3.99e-08	1.28e-06%
**OTU_5992**	**True**	**ZB2**	1.82e-08	1.66e-06%	0.0	1	0.00164	0.22	0.127	2.55e-08	8.15e-07%
**OTU_1177**	**True**	**OP3**	1.79e-08	1.63e-06%	0.001	0	0.000137	0.000112	0.000113	2.52e-06	8.08e-05%
**L970**	**False**	**koll11**	1.28e-08	1.16e-06%	0.0	1	0.175	0.0204	0.0281	2.68e-09	8.57e-08%
**OTU_6682**	**True**	**ZB2**	1.1e-08	9.99e-07%	0.0	1	0.0	0.0	6.86e-06	2.13e-08	6.81e-07%
**OTU_188**	**True**	**koll11**	1.02e-08	9.27e-07%	0.101	0	1.81e-06	1.6e-07	0.0	2.75e-08	8.81e-07%
**OTU_2868**	**True**	**SJA-4**	7.02e-09	6.39e-07%	0.148	0	0.0	0.0	1.33e-06	2.24e-08	7.18e-07%
**L1007**	**False**	**koll11**	6.27e-09	5.71e-07%	0.0	1	0.0862	0.00956	0.016	1.2e-09	3.84e-08%
**L1433**	**False**	**ZB2**	4.68e-09	4.26e-07%	0.0	1	0.00559	0.0509	0.079	4e-09	1.28e-07%
**L808**	**False**	**OP3**	3.19e-09	2.91e-07%	0.0	1	0.0421	0.00375	0.00886	2.55e-09	8.18e-08%
**L1498**	**False**	**ZB2**	2.72e-09	2.48e-07%	0.0	1	0.000476	0.0291	0.0322	6.81e-09	2.18e-07%
**L170**	**False**	**OD1**	2.36e-09	2.15e-07%	0.0	1	0.0486	0.017	0.00919	2.13e-09	6.83e-08%
**L468**	**False**	**OP11-4**	2.32e-09	2.11e-07%	0.0	1	0.0293	0.00439	0.00327	1.6e-09	5.14e-08%
**L953**	**False**	**koll11**	2.23e-09	2.03e-07%	0.0	1	0.0216	0.0017	0.00119	1.39e-09	4.45e-08%
**L319**	**False**	**OD1**	1.92e-09	1.75e-07%	0.0	1	0.0199	0.00202	0.00134	9.7e-10	3.11e-08%
**L1042**	**False**	**koll11**	1.62e-09	1.47e-07%	0.0	1	0.0194	0.00252	0.00213	5.65e-10	1.81e-08%
**L1682**	**False**	**OD1**	1.45e-09	1.32e-07%	0.0	1	0.0171	0.00273	0.00139	6.39e-10	2.05e-08%
**L1043**	**False**	**koll11**	1.36e-09	1.24e-07%	0.0	1	0.0158	0.00182	0.00173	6.31e-10	2.02e-08%
**L230**	**False**	**ABY1**	1.12e-09	1.02e-07%	0.0	1	0.0134	0.00172	0.00155	9.75e-10	3.12e-08%
**OTU_7230**	**True**	**ABY1**	9.49e-10	8.64e-08%	0.0	1	0.00914	0.00113	0.000286	8.79e-10	2.82e-08%
**OTU_6022**	**True**	**OD1**	7.61e-10	6.93e-08%	0.0	1	0.00717	0.00092	0.000174	7.44e-10	2.38e-08%
**L1421**	**False**	**ZB2**	5.92e-10	5.39e-08%	0.0	1	0.000111	0.00392	0.00803	1.05e-09	3.38e-08%
**L169**	**False**	**OD1**	5.77e-10	5.26e-08%	0.0	1	0.0603	0.0406	0.0352	4.09e-09	1.31e-07%
**L1280**	**False**	**TM7-3**	5.7e-10	5.19e-08%	0.0	1	0.00461	0.000237	9.33e-05	1.03e-09	3.29e-08%
**L249**	**False**	**ABY1**	5.3e-10	4.83e-08%	0.0	1	0.00579	0.00139	0.000179	7.43e-10	2.38e-08%
**L1124**	**False**	**koll11**	5.06e-10	4.61e-08%	0.0	1	0.00504	0.000381	0.000441	7.17e-10	2.3e-08%
**L324**	**False**	**ABY1**	4.63e-10	4.22e-08%	0.0	1	0.00387	0.000221	0.000112	4.55e-10	1.46e-08%
**L1455**	**False**	**ZB2**	4.63e-10	4.21e-08%	0.0	1	0.000856	0.00492	0.00924	8.72e-10	2.79e-08%
**OTU_6693**	**True**	**ZB2**	4.25e-10	3.87e-08%	1.0	0	0.0	0.0	1.48e-07	1.14e-09	3.65e-08%
**L1788**	**False**	**OD1**	3.62e-10	3.3e-08%	0.0	1	0.00405	0.000564	0.000357	2.97e-10	9.53e-09%
**L763**	**False**	**PBS-25**	3.53e-10	3.21e-08%	0.0	1	0.00248	6.58e-05	7.42e-08	8.3e-10	2.66e-08%
**L682**	**False**	**koll11**	3.35e-10	3.05e-08%	0.0	1	0.00584	0.00122	0.00149	5.2e-10	1.67e-08%
**L1566**	**False**	**ZB2**	3.16e-10	2.87e-08%	0.0	1	0.00432	0.000611	0.000841	4.16e-10	1.33e-08%
**OTU_5548**	**True**	**OD1**	3.13e-10	2.85e-08%	0.0	1	0.0031	7.56e-05	0.000601	2.85e-10	9.12e-09%
**L1300**	**False**	**EW055**	2.86e-10	2.6e-08%	0.0	1	0.00251	0.000236	6.28e-05	9.15e-10	2.93e-08%
**L164**	**False**	**ABY1**	2.43e-10	2.21e-08%	0.0	1	0.00208	0.000161	5.72e-05	2.77e-10	8.87e-09%
**L884**	**False**	**koll11**	2.41e-10	2.2e-08%	0.0	1	0.00368	0.000713	0.000737	5.31e-10	1.7e-08%
**L1103**	**False**	**koll11**	2.32e-10	2.11e-08%	0.0	1	0.00243	0.000114	0.000446	3.08e-10	9.86e-09%
**OTU_7396**	**True**	**ABY1**	2.29e-10	2.09e-08%	0.0	1	0.00286	0.000753	0.000204	8.5e-10	2.72e-08%
**L369**	**False**	**Bacteria**	2.25e-10	2.05e-08%	0.0	1	0.837	0.844	0.902	2.31e-09	7.39e-08%
**L774**	**False**	**PBS-25**	2.14e-10	1.95e-08%	0.0	1	0.00254	0.000273	0.000408	7.04e-10	2.25e-08%
**L1729**	**False**	**Mb-NB09**	2.01e-10	1.83e-08%	0.0	1	0.00307	0.000492	0.000769	5.05e-10	1.62e-08%
**L922**	**False**	**koll11**	2e-10	1.82e-08%	0.0	1	0.00132	4.42e-06	4.19e-06	3.23e-10	1.03e-08%
**L1732**	**False**	**Mb-NB09**	1.72e-10	1.57e-08%	0.0	1	0.0019	0.000421	8.9e-05	4.78e-10	1.53e-08%
**L1633**	**False**	**ZB2**	1.45e-10	1.32e-08%	0.0	1	0.00329	0.000809	0.00135	7.82e-10	2.51e-08%
**L896**	**False**	**koll11**	1.38e-10	1.25e-08%	0.0	1	0.00302	0.000611	0.00215	6.01e-10	1.92e-08%
**L689**	**False**	**koll11**	1.37e-10	1.24e-08%	0.0	1	0.00294	0.000616	0.00141	4.32e-10	1.38e-08%
**OTU_4847**	**True**	**ZB2**	1.32e-10	1.2e-08%	0.0	1	0.00101	6.21e-05	6.4e-06	1.77e-10	5.67e-09%
**L384**	**False**	**ABY1**	1.25e-10	1.14e-08%	0.0	1	0.000897	2.83e-05	5.3e-06	1.89e-10	6.06e-09%

**L640**	**False**	**PBS-25**	1.14e-10	1.04e-08%	0.0	1	0.000807	2.03e-05	5.37e-06	1.9e-10	6.08e-09%
**L388**	**False**	**ABY1**	1.13e-10	1.03e-08%	0.0	1	0.0134	0.00855	0.00966	1.49e-09	4.77e-08%
**OTU_8209**	**True**	**ABY1**	1.08e-10	9.87e-09%	0.0	1	6.66e-05	0.00176	0.000615	3.52e-10	1.13e-08%
**OTU_6407**	**True**	**ZB2**	8.95e-11	8.16e-09%	0.0	1	1.24e-05	0.00129	0.000642	1.29e-10	4.12e-09%
**OTU_851**	**True**	**koll11**	8.07e-11	7.35e-09%	0.0	1	0.000899	0.000101	0.000116	2.08e-10	6.67e-09%
**L795**	**False**	**PBS-25**	7.94e-11	7.23e-09%	0.0	1	0.000615	4.38e-05	4.53e-06	1.08e-10	3.46e-09%
**OTU_7956**	**True**	**OD1**	7.71e-11	7.03e-09%	0.0	1	0.000608	8.93e-05	0.00125	3.67e-10	1.18e-08%
**OTU_876**	**True**	**koll11**	7.07e-11	6.44e-09%	0.0	1	0.000445	0.0	0.0	1.44e-10	4.62e-09%
**L371**	**False**	**ABY1**	7.04e-11	6.41e-09%	0.0	1	0.000793	0.000287	2.15e-05	2.15e-10	6.89e-09%
**OTU_5946**	**True**	**OD1**	6.41e-11	5.84e-09%	0.0	1	0.000615	5.2e-05	5.06e-05	1.58e-10	5.05e-09%
**L1751**	**False**	**OD1**	4.32e-11	3.94e-09%	0.0	1	0.000842	0.000713	0.00196	5.98e-10	1.92e-08%
**OTU_309**	**True**	**PBS-25**	3.97e-11	3.62e-09%	0.0	1	0.000558	7.49e-05	0.000152	1.5e-10	4.8e-09%
**OTU_6109**	**True**	**ZB2**	3.7e-11	3.37e-09%	0.0	1	4.83e-06	0.000515	0.000399	7.65e-11	2.45e-09%
**OTU_5803**	**True**	**OD1**	3.67e-11	3.34e-09%	0.0	1	0.000319	1.78e-05	2.26e-05	1.21e-10	3.88e-09%
**OTU_6715**	**True**	**ZB2**	3.63e-11	3.3e-09%	0.0	1	0.000325	5.57e-05	4.2e-06	1.01e-10	3.22e-09%
**OTU_6578**	**True**	**WCHB1-64**	3.28e-11	2.99e-09%	0.0	1	0.000131	8.03e-05	0.00059	1.77e-10	5.67e-09%
**OTU_5805**	**True**	**OD1**	3.22e-11	2.94e-09%	0.0	1	0.000352	4.62e-05	3.93e-05	8.02e-11	2.57e-09%
**OTU_7190**	**True**	**ABY1**	3.18e-11	2.9e-09%	0.0	1	0.0	0.000415	0.000312	9.9e-11	3.17e-09%
**OTU_411**	**True**	**koll11**	3.12e-11	2.84e-09%	0.0	1	0.000399	7.08e-05	6.07e-05	1.41e-10	4.5e-09%
**OTU_1017**	**True**	**PRR-12**	2.39e-11	2.17e-09%	0.0	1	0.000194	0.0	3.09e-05	9.02e-11	2.89e-09%
**OTU_5244**	**True**	**OD1**	2.36e-11	2.15e-09%	0.0	1	0.000259	7.48e-05	1.05e-05	1.05e-10	3.38e-09%
**OTU_6594**	**True**	**ZB2**	2e-11	1.82e-09%	0.0	1	0.000169	3.11e-05	0.0	7.03e-11	2.25e-09%
**OTU_9186**	**True**	**Mb-NB09**	6.91e-12	6.29e-10%	0.0	1	0.000152	0.000127	3.12e-05	6.99e-11	2.24e-09%
**L1295**	**False**	**TM7-3**	6.84e-13	6.23e-11%	0.0	1	4.01e-06	0.0	0.0	1.45e-12	4.65e-11%
**OTU_144**	**True**	**Archaea**	0.0	0.0%	1.0	0	0.0	0.0	0.0	0.0	0.0%
**OTU_11097**	**True**	**Archaea**	0.0	0.0%	1.0	0	0.0	0.0	0.0	0.0	0.0%
**OTU_10991**	**True**	**Archaea**	0.0	0.0%	1.0	0	0.0	0.0	0.0	0.0	0.0%
**OTU_13**	**True**	**Archaea**	0.0	0.0%	1.0	0	0.0	0.0	0.0	0.0	0.0%
**L1977**	**False**	**Archaea**	0.0	0.0%	1.0	0	0.0	0.0	0.0	0.0	0.0%
**OTU_11044**	**True**	**Archaea**	0.0	0.0%	1.0	0	0.0	0.0	0.0	0.0	0.0%
**OTU_11020**	**True**	**Archaea**	0.0	0.0%	1.0	0	0.0	0.0	0.0	0.0	0.0%
**L1975**	**False**	**Archaea**	0.0	0.0%	1.0	0	0.0	0.0	0.0	0.0	0.0%
**L1953**	**False**	**Archaea**	0.0	0.0%	1.0	0	0.0	0.0	0.0	0.0	0.0%
**OTU_3999**	**True**	**Archaea**	0.0	0.0%	1.0	0	0.0	0.0	0.0	0.0	0.0%
**L1955**	**False**	**Archaea**	0.0	0.0%	1.0	0	0.0	0.0	0.0	0.0	0.0%
**OTU_102**	**True**	**Archaea**	0.0	0.0%	1.0	0	0.0	0.0	0.0	0.0	0.0%
**OTU_112**	**True**	**Archaea**	0.0	0.0%	1.0	0	0.0	0.0	0.0	0.0	0.0%
**OTU_11341**	**True**	**Archaea**	0.0	0.0%	1.0	0	0.0	0.0	0.0	0.0	0.0%
**L1959**	**False**	**Archaea**	0.0	0.0%	1.0	0	0.0	0.0	0.0	0.0	0.0%
**L1960**	**False**	**Archaea**	0.0	0.0%	1.0	0	0.0	0.0	0.0	0.0	0.0%
**OTU_11266**	**True**	**Archaea**	0.0	0.0%	1.0	0	0.0	0.0	0.0	0.0	0.0%
**L1962**	**False**	**Archaea**	0.0	0.0%	1.0	0	0.0	0.0	0.0	0.0	0.0%
**OTU_10884**	**True**	**Archaea**	0.0	0.0%	1.0	0	0.0	0.0	0.0	0.0	0.0%
**OTU_11207**	**True**	**Archaea**	0.0	0.0%	1.0	0	0.0	0.0	0.0	0.0	0.0%
**L1965**	**False**	**Archaea**	0.0	0.0%	1.0	0	0.0	0.0	0.0	0.0	0.0%
**OTU_11297**	**True**	**Archaea**	0.0	0.0%	1.0	0	0.0	0.0	0.0	0.0	0.0%
**OTU_11306**	**True**	**Archaea**	0.0	0.0%	1.0	0	0.0	0.0	0.0	0.0	0.0%
**L1968**	**False**	**Archaea**	0.0	0.0%	1.0	0	0.0	0.0	0.0	0.0	0.0%
**OTU_10977**	**True**	**Archaea**	0.0	0.0%	1.0	0	0.0	0.0	0.0	0.0	0.0%
**L1969**	**False**	**Archaea**	0.0	0.0%	1.0	0	0.0	0.0	0.0	0.0	0.0%
**OTU_11073**	**True**	**Archaea**	0.0	0.0%	1.0	0	0.0	0.0	0.0	0.0	0.0%
**L1951**	**False**	**Archaea**	0.0	0.0%	1.0	0	0.0	0.0	0.0	0.0	0.0%
**L1972**	**False**	**Archaea**	0.0	0.0%	1.0	0	0.0	0.0	0.0	0.0	0.0%
**L1973**	**False**	**Archaea**	0.0	0.0%	1.0	0	0.0	0.0	0.0	0.0	0.0%
**L1974**	**False**	**Archaea**	0.0	0.0%	1.0	0	0.0	0.0	0.0	0.0	0.0%
**L1981**	**False**	**Archaea**	0.0	0.0%	1.0	0	0.0	0.0	0.0	0.0	0.0%
**L2008**	**False**	**Archaea**	0.0	0.0%	1.0	0	0.0	0.0	0.0	0.0	0.0%
**L1983**	**False**	**Archaea**	0.0	0.0%	1.0	0	0.0	0.0	0.0	0.0	0.0%
**L1984**	**False**	**Archaea**	0.0	0.0%	1.0	0	0.0	0.0	0.0	0.0	0.0%
**L1987**	**False**	**Archaea**	0.0	0.0%	1.0	0	0.0	0.0	0.0	0.0	0.0%
**L1988**	**False**	**Archaea**	0.0	0.0%	1.0	0	0.0	0.0	0.0	0.0	0.0%
**OTU_10976**	**True**	**Archaea**	0.0	0.0%	1.0	0	0.0	0.0	0.0	0.0	0.0%
**OTU_10957**	**True**	**Archaea**	0.0	0.0%	1.0	0	0.0	0.0	0.0	0.0	0.0%
**OTU_11000**	**True**	**Archaea**	0.0	0.0%	1.0	0	0.0	0.0	0.0	0.0	0.0%
**L1992**	**False**	**Archaea**	0.0	0.0%	1.0	0	0.0	0.0	0.0	0.0	0.0%

**L1993**	**False**	**Archaea**	0.0	0.0%	1.0	0	0.0	0.0	0.0	0.0	0.0%
**OTU_11180**	**True**	**Archaea**	0.0	0.0%	1.0	0	0.0	0.0	0.0	0.0	0.0%
**L1995**	**False**	**Archaea**	0.0	0.0%	1.0	0	0.0	0.0	0.0	0.0	0.0%
**L1996**	**False**	**Archaea**	0.0	0.0%	1.0	0	0.0	0.0	0.0	0.0	0.0%
**OTU_17**	**True**	**Archaea**	0.0	0.0%	1.0	0	0.0	0.0	0.0	0.0	0.0%
**L1998**	**False**	**Archaea**	0.0	0.0%	1.0	0	0.0	0.0	0.0	0.0	0.0%
**OTU_11336**	**True**	**Archaea**	0.0	0.0%	1.0	0	0.0	0.0	0.0	0.0	0.0%
**L2000**	**False**	**Archaea**	0.0	0.0%	1.0	0	0.0	0.0	0.0	0.0	0.0%
**OTU_22**	**True**	**Archaea**	0.0	0.0%	1.0	0	0.0	0.0	0.0	0.0	0.0%
**L2002**	**False**	**Archaea**	0.0	0.0%	1.0	0	0.0	0.0	0.0	0.0	0.0%
**OTU_11284**	**True**	**Archaea**	0.0	0.0%	1.0	0	0.0	0.0	0.0	0.0	0.0%
**L1949**	**False**	**Archaea**	0.0	0.0%	1.0	0	0.0	0.0	0.0	0.0	0.0%
**L2004**	**False**	**Archaea**	0.0	0.0%	1.0	0	0.0	0.0	0.0	0.0	0.0%
**OTU_11308**	**True**	**Archaea**	0.0	0.0%	1.0	0	0.0	0.0	0.0	0.0	0.0%
**OTU_10936**	**True**	**Archaea**	0.0	0.0%	1.0	0	0.0	0.0	0.0	0.0	0.0%
**OTU_11271**	**True**	**Archaea**	0.0	0.0%	1.0	0	0.0	0.0	0.0	0.0	0.0%
**OTU_11285**	**True**	**Archaea**	0.0	0.0%	1.0	0	0.0	0.0	0.0	0.0	0.0%
**OTU_10959**	**True**	**Archaea**	0.0	0.0%	1.0	0	0.0	0.0	0.0	0.0	0.0%
**L2010**	**False**	**Archaea**	0.0	0.0%	1.0	0	0.0	0.0	0.0	0.0	0.0%
**OTU_20**	**True**	**Archaea**	0.0	0.0%	1.0	0	0.0	0.0	0.0	0.0	0.0%
**L2012**	**False**	**Archaea**	0.0	0.0%	1.0	0	0.0	0.0	0.0	0.0	0.0%
**OTU_21**	**True**	**Archaea**	0.0	0.0%	1.0	0	0.0	0.0	0.0	0.0	0.0%
**OTU_10954**	**True**	**Archaea**	0.0	0.0%	1.0	0	0.0	0.0	0.0	0.0	0.0%
**L1950**	**False**	**Archaea**	0.0	0.0%	1.0	0	0.0	0.0	0.0	0.0	0.0%
**OTU_11312**	**True**	**Archaea**	0.0	0.0%	1.0	0	0.0	0.0	0.0	0.0	0.0%
**OTU_11287**	**True**	**Archaea**	0.0	0.0%	1.0	0	0.0	0.0	0.0	0.0	0.0%
**OTU_9114**	**True**	**TM7-1**	0.0	0.0%	1.0	0	0.0	0.0	0.0	0.0	0.0%
**OTU_11281**	**True**	**Archaea**	0.0	0.0%	1.0	0	0.0	0.0	0.0	0.0	0.0%
**L1914**	**False**	**Archaea**	0.0	0.0%	1.0	0	0.0	0.0	0.0	0.0	0.0%
**L1913**	**False**	**Archaea**	0.0	0.0%	1.0	0	0.0	0.0	0.0	0.0	0.0%
**L1912**	**False**	**Archaea**	0.0	0.0%	1.0	0	0.0	0.0	0.0	0.0	0.0%
**OTU_5846**	**True**	**OD1**	0.0	0.0%	1.0	0	0.0	0.0	0.0	0.0	0.0%
**OTU_8168**	**True**	**ZB2**	0.0	0.0%	1.0	0	0.0	0.0	0.0	0.0	0.0%
**OTU_6694**	**True**	**ZB2**	0.0	0.0%	1.0	0	0.0	0.0	0.0	0.0	0.0%
**OTU_7192**	**True**	**ZB2**	0.0	0.0%	1.0	0	0.0	0.0	0.0	0.0	0.0%
**OTU_5341**	**True**	**OD1**	0.0	0.0%	1.0	0	0.0	0.0	0.0	0.0	0.0%
**OTU_8196**	**True**	**TM7-3**	0.0	0.0%	1.0	0	0.0	0.0	0.0	0.0	0.0%
**OTU_8171**	**True**	**TM7-3**	0.0	0.0%	1.0	0	0.0	0.0	0.0	0.0	0.0%
**OTU_8182**	**True**	**EW055**	0.0	0.0%	1.0	0	0.0	0.0	0.0	0.0	0.0%
**OTU_8180**	**True**	**EW055**	0.0	0.0%	1.0	0	0.0	0.0	0.0	0.0	0.0%
**OTU_9113**	**True**	**TM7-1**	0.0	0.0%	1.0	0	0.0	0.0	0.0	0.0	0.0%
**OTU_11012**	**True**	**Archaea**	0.0	0.0%	1.0	0	0.0	0.0	0.0	0.0	0.0%
**L1269**	**False**	**TM7-1**	0.0	0.0%	1.0	0	0.0	0.0	0.0	0.0	0.0%
**OTU_8178**	**True**	**TM7-1**	0.0	0.0%	1.0	0	0.0	0.0	0.0	0.0	0.0%
**OTU_9149**	**True**	**TM7-1**	0.0	0.0%	1.0	0	0.0	0.0	0.0	0.0	0.0%
**OTU_470**	**True**	**koll11**	0.0	0.0%	1.0	0	0.0	0.0	0.0	0.0	0.0%
**OTU_472**	**True**	**koll11**	0.0	0.0%	1.0	0	0.0	0.0	0.0	0.0	0.0%
**OTU_4223**	**True**	**koll11**	0.0	0.0%	1.0	0	0.0	0.0	0.0	0.0	0.0%
**OTU_1354**	**True**	**PBS-25**	0.0	0.0%	1.0	0	0.0	0.0	0.0	0.0	0.0%
**OTU_477**	**True**	**PRR-12**	0.0	0.0%	1.0	0	0.0	0.0	0.0	0.0	0.0%
**OTU_3027**	**True**	**SJA-4**	0.0	0.0%	1.0	0	0.0	0.0	0.0	0.0	0.0%
**OTU_10777**	**True**	**OP11-3**	0.0	0.0%	1.0	0	0.0	0.0	0.0	0.0	0.0%
**OTU_10819**	**True**	**OP11-4**	0.0	0.0%	1.0	0	0.0	0.0	0.0	0.0	0.0%
**OTU_5181**	**True**	**BB36**	0.0	0.0%	1.0	0	0.0	0.0	0.0	0.0	0.0%
**OTU_9137**	**True**	**ABY1**	0.0	0.0%	1.0	0	0.0	0.0	0.0	0.0	0.0%
**OTU_11188**	**True**	**Archaea**	0.0	0.0%	1.0	0	0.0	0.0	0.0	0.0	0.0%
**L1915**	**False**	**Archaea**	0.0	0.0%	1.0	0	0.0	0.0	0.0	0.0	0.0%
**L1918**	**False**	**Archaea**	0.0	0.0%	1.0	0	0.0	0.0	0.0	0.0	0.0%
**OTU_11167**	**True**	**Archaea**	0.0	0.0%	1.0	0	0.0	0.0	0.0	0.0	0.0%
**L1946**	**False**	**Archaea**	0.0	0.0%	1.0	0	0.0	0.0	0.0	0.0	0.0%
**L1945**	**False**	**Archaea**	0.0	0.0%	1.0	0	0.0	0.0	0.0	0.0	0.0%
**OTU_10979**	**True**	**Archaea**	0.0	0.0%	1.0	0	0.0	0.0	0.0	0.0	0.0%
**OTU_11015**	**True**	**Archaea**	0.0	0.0%	1.0	0	0.0	0.0	0.0	0.0	0.0%
**OTU_11153**	**True**	**Archaea**	0.0	0.0%	1.0	0	0.0	0.0	0.0	0.0	0.0%
**L1941**	**False**	**Archaea**	0.0	0.0%	1.0	0	0.0	0.0	0.0	0.0	0.0%

**L1940**	**False**	**Archaea**	0.0	0.0%	1.0	0	0.0	0.0	0.0	0.0	0.0%
**OTU_10982**	**True**	**Archaea**	0.0	0.0%	1.0	0	0.0	0.0	0.0	0.0	0.0%
**L1938**	**False**	**Archaea**	0.0	0.0%	1.0	0	0.0	0.0	0.0	0.0	0.0%
**OTU_10944**	**True**	**Archaea**	0.0	0.0%	1.0	0	0.0	0.0	0.0	0.0	0.0%
**OTU_10946**	**True**	**Archaea**	0.0	0.0%	1.0	0	0.0	0.0	0.0	0.0	0.0%
**L1935**	**False**	**Archaea**	0.0	0.0%	1.0	0	0.0	0.0	0.0	0.0	0.0%
**L1919**	**False**	**Archaea**	0.0	0.0%	1.0	0	0.0	0.0	0.0	0.0	0.0%
**OTU_11121**	**True**	**Archaea**	0.0	0.0%	1.0	0	0.0	0.0	0.0	0.0	0.0%
**L1932**	**False**	**Archaea**	0.0	0.0%	1.0	0	0.0	0.0	0.0	0.0	0.0%
**OTU_11194**	**True**	**Archaea**	0.0	0.0%	1.0	0	0.0	0.0	0.0	0.0	0.0%
**OTU_10967**	**True**	**Archaea**	0.0	0.0%	1.0	0	0.0	0.0	0.0	0.0	0.0%
**L1921**	**False**	**Archaea**	0.0	0.0%	1.0	0	0.0	0.0	0.0	0.0	0.0%
**L1922**	**False**	**Archaea**	0.0	0.0%	1.0	0	0.0	0.0	0.0	0.0	0.0%
**L1923**	**False**	**Archaea**	0.0	0.0%	1.0	0	0.0	0.0	0.0	0.0	0.0%
**OTU_10973**	**True**	**Archaea**	0.0	0.0%	1.0	0	0.0	0.0	0.0	0.0	0.0%
**L1925**	**False**	**Archaea**	0.0	0.0%	1.0	0	0.0	0.0	0.0	0.0	0.0%
**L1926**	**False**	**Archaea**	0.0	0.0%	1.0	0	0.0	0.0	0.0	0.0	0.0%
**OTU_11120**	**True**	**Archaea**	0.0	0.0%	1.0	0	0.0	0.0	0.0	0.0	0.0%
**OTU_10942**	**True**	**Archaea**	0.0	0.0%	1.0	0	0.0	0.0	0.0	0.0	0.0%
**OTU_11276**	**True**	**Archaea**	0.0	0.0%	1.0	0	0.0	0.0	0.0	0.0	0.0%
**L1930**	**False**	**Archaea**	0.0	0.0%	1.0	0	0.0	0.0	0.0	0.0	0.0%
**L1**	**False**	**Bacteria**	-2.60311691088e-17	-2.37130878137e-15%	0.993	0	1.0	1.0	1.0	6.47e-17	2.07e-15%
**L0**	**False**	**Bacteria**	-5.66747234479e-17	-5.16278269457e-15%	0.993	0	1.0	1.0	1.0	1.41e-16	4.51e-15%
**OTU_458**	**True**	**koll11**	-5.6197248056e-10	-5.11928708417e-08%	1.0	0	3.22e-05	3.43e-05	4.33e-05	8.18e-07	2.62e-05%
**L1478**	**False**	**ZB2**	-1.15250869287e-08	-1.04987754204e-06%	1.0	0	0.000313	0.000492	0.000384	1.11e-05	0.000357%
**L1237**	**False**	**TM7**	-2.28331450697e-07	-2.07998485139e-05%	1.0	0	0.0105	0.0121	0.0102	0.000256	0.00821%

phyloh_output_all_sample

Partitioning information in /home/anna/Scrivania/openw/progetto_acqua/16S_merge_work/phyloH.raxml/

Table of Contents

The run call was:

esecutorePhyloHPandas.py -s /home/anna/Scrivania/openw/progetto_acqua/16S_merge_work/phyloH.raxml/raxml.nano01.01.sample.txt -t /home/anna/Scrivania/openw/progetto_acqua/16S_merge_work/phyloH.raxml/nanoarchea_taxa_mod.txt -f /home/anna/Scrivania/openw/progetto_acqua/16S_merge_work/phyloH.raxml/nanoarchea.lb.rm.reroot_newick.txt -g /home/anna/Scrivania/openw/progetto_acqua/16S_merge_work/phyloH.raxml/map_h2o_M.txt -r 999 -h 1 -q 1 -e 1 -k 1 -o /home/anna/Scrivania/openw/progetto_acqua/16S_merge_work/phyloH.raxml/nano_arche.lb.rm.all.paper999taxamode1k1

Experimental Design:

Counts of observations across groups and samples within groups

Total Counts	Group Name	Group Counts	Sample Name	Sample Counts
4267536	Aquifer	542957	10A.r.s	2381
			10A.s	62411
			11A.r.s	151853
			11A.s	101531
			12A.r.s	20449
			12A.s	56553
			13A.r.s	14488
			13A.s	20192
			14A.r.s	43550
			14A.s	39416
			1A.cr.r.s	33918
			1A.cr.s	88003
			2A.cr.r.s	14254
			2A.cr.s	11758
			3A.r.s	8572
			3A.s	14119
			4A.r.s	4789
			4A.s	14021
			5A.r.s	13944
			5A.s	228900
			6A.r.s	46101
			6A.s	9801
			7A.r.s	85755
			7A.s	8683
			8A.r.s	12083
			8A.s	2412
			9A.r.s	10728
			9A.s	17584
	Cfilters	2032592	10B.r.s	102777
			10B.s	2654
			11B.r.s	2580
			11B.s	1795
			12B.r.s	87148
			12B.s	8342
			13B.r.s	9825
			13B.s	5704
			14B.r.s	2552
			14B.s	18927
			1B.cr.r.s	612596
			1B.cr.s	212195
			2B.cr.r.s	1207
			2B.cr.s	5133
			3B.r.s	12796
			3B.s	196761
			4B.r.s	58469
			4B.s	63137
			5B.r.s	14924

			5B.s	7225	
			6B.r.s	12747	
			6B.s	1106	
			7B.r.s	50607	
			7B.s	34101	
			8B.r.s	22187	
			8B.s	283518	
			9B.r.s	22565	
			9B.s	3972	
	Chlor	1691987	10C.r.s	27577	
			10C.s	7622	
			11C.r.s	4444	
			11C.s	19608	
			12C.r.s	202073	
			12C.s	7657	
			13C.r.s	64882	
			13C.s	80259	
			14C.r.s	18105	
			14C.s	10395	
			1C.cr.r.s	1790	
			1C.cr.s	27784	
			2C.cr.r.s	21877	
			2C.cr.s	84514	
			3C.r.s	48275	
			3C.s	6724	
			4C.r.s	53103	
			4C.s	5600	
			5C.r.s	3430	
			5C.s	251395	
			6C.r.s	100190	
			6C.s	21846	
			7C.r.s	25605	
			7C.s	8852	
			8C.r.s	51516	
			8C.s	20891	
			9C.r.s	73243	
			9C.s	22480	

Entropy across samples, groups and samples within groups

MaxDiversity gives the values for a maximally balanced experimental design

	nats	Diversity	MaxDiversity
H(G)	1.1	3.0	3.0
H(S)	4.43	84.0	84.0
H(S|G)	3.33	28.0	28.0

Gamma diversities:

Total entropy and diversity within each group and overall data

Unit measure for Diversity is equivalent number of independent equi-abundant linneages

	nats	Diversity
Group		
Aquifer	0.783	2.19
Cfilters	0.501	1.65
Chlor	0.472	1.6
Overall	0.682	1.98

Alpha diversities:

Mean entropy and diversity within sample or group

Unit measure for Diversity is equivalent number of independent equi-abundant linneages

	nats	Diversity
H(T|S)	0.482	1.62

**H(T|G)**	0.585	1.8

Beta diversity:

Information shared across Tree and Sample or Group vector expressed as nats and turnover of linneage.

Turnover is the percentage of observations not shared across groups

Pvalue is computed with Permutation procedure

	nats	TurnOver	pvalue
**I(T,G)**	0.0971	8.84%	0.0
**I(T,S|G)**	0.104	3.11%	0.0

Difference of each group from total:

phylogenetic Kullback-Leiber distance between each group and the overall data

	KullBack-Lieber(PG(i)||Ptot(i)
**Group**	
**Aquifer**	0.19
**Cfilters**	0.0397
**Chlor**	0.0526

Pairwise TurnOver between groups

	Aquifer	Cfilters	Chlor
**Aquifer**			
**Cfilters**	13.9%		
**Chlor**	13.6%	0.804%	

Per Node Statistics mapped on the phylogeny

Three types of data are shown on the tree:

1. The color of the branches cyan indicates a contribution to I(T,E) higher than the null distribution, while branches are black otherwise.
2. The background of each branch is a gradient from yellow to red for increased contribution to I(T,E). For details look at the legend on the side
3. Bar plot on each tips indicates the relative frequencies in each group

Look at the tree find an relevant branches and text search the label of the branch to access the correct row on the by node statistics table.

Go itol using the link to modify the tree, or use the itol table and the labelled tree to add further data set (i.e. taxonomic name)

Click here to modify image

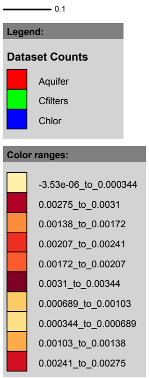

Per Node Statistics in tabular format:

		Metric	I(Ti,G)				By Group Relative Frequency			I(Ti,S|G)	
		Stat	nats	TurnOver	pvalue	MultTest	Aquifer	Cfilters	Chlor	nats	TurnOver
Name	Is_Leaf	Taxonomy									
L575	False	Bacteria	0.00378	0.344%	0.0	1	0.536	0.0814	0.0991	0.00129	0.0386%
L526	False	Bacteria	0.0032	0.291%	0.0	1	0.543	0.085	0.102	0.00115	0.0346%
L420	False	Bacteria	0.00309	0.282%	0.0	1	0.613	0.109	0.16	0.00202	0.0607%
OTU_6998	True	ZB2	0.00291	0.265%	0.0	1	0.000611	0.0393	0.152	0.00323	0.0968%
L1372	False	OD1	0.00279	0.254%	0.0	1	0.0413	0.637	0.576	0.00127	0.0381%
L1326	False	OD1	0.00269	0.245%	0.0	1	0.0861	0.701	0.742	0.000939	0.0282%
L422	False	Bacteria	0.00253	0.23%	0.0	1	0.582	0.0957	0.11	0.000919	0.0276%
L1379	False	ZB2	0.0024	0.219%	0.0	1	0.0156	0.564	0.479	0.00144	0.0432%
L1375	False	OD1	0.00237	0.216%	0.0	1	0.0166	0.564	0.479	0.00144	0.0432%
L1324	False	OD1	0.00236	0.214%	0.0	1	0.126	0.711	0.746	0.00091	0.0273%
L1331	False	OD1	0.00232	0.212%	0.0	1	0.085	0.701	0.742	0.000804	0.0241%
L527	False	Bacteria	0.00226	0.206%	0.0	1	0.542	0.085	0.102	0.000817	0.0245%
L1363	False	OD1	0.00215	0.196%	0.0	1	0.0661	0.682	0.732	0.000759	0.0228%
L1325	False	OD1	0.00189	0.172%	0.0	1	0.108	0.707	0.745	0.000689	0.0207%
L1328	False	OD1	0.00162	0.147%	0.0	1	0.0853	0.701	0.742	0.000561	0.0168%
L921	False	koll11	0.00162	0.147%	0.0	1	0.244	0.038	0.0476	0.0006	0.018%
L421	False	Bacteria	0.00154	0.14%	0.0	1	0.607	0.109	0.159	0.00103	0.031%
L1391	False	ZB2	0.00133	0.121%	0.0	1	0.013	0.349	0.346	0.00148	0.0445%
L1315	False	OD1	0.00128	0.117%	0.0	1	0.223	0.736	0.753	0.000726	0.0218%
L1350	False	OD1	0.00111	0.101%	0.0	1	0.08	0.697	0.739	0.000383	0.0115%
L612	False	OP3	0.00109	0.0991%	0.0	1	0.5	0.0772	0.0969	0.000421	0.0126%
L1323	False	OD1	0.00107	0.097%	0.0	1	0.211	0.735	0.752	0.000569	0.0171%
L1330	False	OD1	0.00105	0.0958%	0.0	1	0.0852	0.701	0.742	0.000364	0.0109%
L1373	False	OD1	0.000981	0.0893%	0.0	1	0.0169	0.564	0.479	0.000596	0.0179%
L1381	False	ZB2	0.000962	0.0876%	0.0	1	0.0156	0.564	0.479	0.000576	0.0173%
L920	False	koll11	0.000958	0.0872%	0.0	1	0.255	0.0424	0.0511	0.000369	0.0111%
L576	False	Bacteria	0.000955	0.0869%	0.0	1	0.532	0.0813	0.0989	0.000326	0.00978%
L1332	False	OD1	0.00094	0.0855%	0.0	1	0.084	0.699	0.74	0.000327	0.00981%
L914	False	OP3	0.000912	0.083%	0.0	1	0.256	0.0424	0.0511	0.000346	0.0104%
L973	False	koll11	0.00086	0.0782%	0.0	1	0.177	0.0267	0.0352	0.000313	0.0094%
L983	False	koll11	0.000811	0.0738%	0.0	1	0.168	0.025	0.0344	0.00031	0.00931%
L1386	False	ZB2	0.000789	0.0718%	0.0	1	0.0025	0.21	0.131	0.0012	0.0359%
L1407	False	ZB2	0.000689	0.0628%	0.0	1	0.00598	0.296	0.32	0.000912	0.0274%
L1383	False	ZB2	0.000579	0.0527%	0.0	1	0.0156	0.56	0.478	0.000355	0.0106%
L1587	False	ZB2	0.000568	0.0517%	0.0	1	0.000753	0.0393	0.152	0.000633	0.019%
L840	False	OP3	0.000566	0.0515%	0.0	1	0.0647	0.00704	0.00868	0.000272	0.00817%
L1401	False	ZB2	0.000519	0.0472%	0.0	1	0.00856	0.346	0.344	0.000552	0.0166%

**L1768**	**False**	**OD1**	0.000511	0.0465%	0.0	1	0.0277	0.00537	0.00169	0.000855	0.0257%
**L1393**	**False**	**ZB2**	0.000509	0.0464%	0.0	1	0.0124	0.349	0.346	0.000563	0.0169%
**L620**	**False**	**OP3**	0.0005	0.0455%	0.0	1	0.499	0.0771	0.0969	0.000194	0.00581%
**L1370**	**False**	**OD1**	0.00048	0.0437%	0.0	1	0.0654	0.641	0.578	0.00025	0.00751%
**L706**	**False**	**OP3**	0.000455	0.0414%	0.0	1	0.116	0.0148	0.0253	0.000327	0.0098%
**L622**	**False**	**OP3**	0.000422	0.0384%	0.0	1	0.152	0.0209	0.0309	0.000233	0.007%
**L990**	**False**	**koll11**	0.00041	0.0373%	0.0	1	0.153	0.0223	0.0335	0.000162	0.00485%
**OTU_7148**	**True**	**OD1**	0.000407	0.037%	0.0	1	0.0257	0.00496	0.00159	0.000767	0.023%
**L1371**	**False**	**OD1**	0.000407	0.037%	0.0	1	0.0648	0.641	0.578	0.000212	0.00636%
**L938**	**False**	**koll11**	0.000403	0.0367%	0.0	1	0.238	0.0377	0.0472	0.000153	0.0046%
**L925**	**False**	**koll11**	0.000391	0.0356%	0.0	1	0.243	0.038	0.0476	0.000147	0.00441%
**L621**	**False**	**OP3**	0.000374	0.0341%	0.0	1	0.244	0.0347	0.0458	0.000181	0.00543%
**L458**	**False**	**OP11-4**	0.000364	0.0331%	0.0	1	0.0321	0.00626	0.00366	0.000265	0.00796%
**L783**	**False**	**OP3**	0.000363	0.0331%	0.0	1	0.056	0.00728	0.00883	0.000272	0.00816%
**L443**	**False**	**OP11**	0.000355	0.0323%	0.0	1	0.0365	0.00725	0.00475	0.000249	0.00746%
**L1388**	**False**	**ZB2**	0.000353	0.0322%	0.0	1	0.00249	0.209	0.13	0.000546	0.0164%
**L861**	**False**	**koll11**	0.000332	0.0302%	0.0	1	0.0268	0.00674	0.00625	0.000418	0.0125%
**L838**	**False**	**OP3**	0.00032	0.0291%	0.0	1	0.065	0.00704	0.00868	0.000154	0.00461%
**L644**	**False**	**OP3**	0.000306	0.0278%	0.0	1	0.14	0.0188	0.0305	0.000189	0.00568%
**OTU_5414**	**True**	**ZB2**	0.000297	0.0271%	0.0	1	0.0048	0.268	0.248	0.000569	0.0171%
**L927**	**False**	**koll11**	0.000297	0.027%	0.0	1	0.241	0.038	0.0476	0.000113	0.00338%
**L1317**	**False**	**OD1**	0.000291	0.0265%	0.0	1	0.00977	0.000483	0.000393	0.000195	0.00584%
**L1680**	**False**	**OD1**	0.000288	0.0262%	0.0	1	0.0178	0.0039	0.00109	0.000209	0.00626%
**L929**	**False**	**koll11**	0.000282	0.0257%	0.0	1	0.241	0.0379	0.0473	0.000106	0.00318%
**OTU_6187**	**True**	**ZB2**	0.000276	0.0251%	0.0	1	0.000135	0.0119	0.0193	0.000752	0.0226%
**L1384**	**False**	**ZB2**	0.000273	0.0248%	0.0	1	0.00255	0.212	0.132	0.000399	0.012%
**OTU_65**	**True**	**OD1**	0.000247	0.0225%	0.0	1	0.00521	0.00203	0.000158	0.000604	0.0181%
**L577**	**False**	**PRR-12**	0.000244	0.0223%	0.0	1	0.0324	0.00409	0.00199	0.000202	0.00606%
**L173**	**False**	**SM2F11**	0.00024	0.0218%	0.0	1	0.01	0.00209	0.000433	0.000202	0.00608%
**L940**	**False**	**koll11**	0.000239	0.0218%	0.0	1	0.238	0.0376	0.0472	9.05e-05	0.00272%
**L1403**	**False**	**ZB2**	0.000238	0.0217%	0.0	1	0.000542	0.05	0.0241	0.000582	0.0175%
**L968**	**False**	**koll11**	0.000236	0.0215%	0.0	1	0.195	0.0296	0.041	8.94e-05	0.00268%
**L1410**	**False**	**ZB2**	0.000236	0.0215%	0.0	1	0.0058	0.292	0.313	0.000331	0.00993%
**L950**	**False**	**koll11**	0.000233	0.0212%	0.0	1	0.228	0.0353	0.046	9.2e-05	0.00276%
**L1431**	**False**	**ZB2**	0.00023	0.021%	0.0	1	0.00512	0.0436	0.064	0.000332	0.00996%
**L931**	**False**	**koll11**	0.000221	0.0201%	0.0	1	0.241	0.0379	0.0473	8.32e-05	0.0025%
**L39**	**False**	**OD1**	0.000219	0.0199%	0.0	1	0.0146	0.00251	0.00184	0.000285	0.00857%
**L1767**	**False**	**OD1**	0.000217	0.0198%	0.0	1	0.0668	0.0182	0.00455	0.000193	0.0058%
**L707**	**False**	**PBS-25**	0.000215	0.0196%	0.0	1	0.0458	0.00624	0.0135	0.000337	0.0101%
**L645**	**False**	**koll11**	0.000214	0.0195%	0.0	1	0.0235	0.00407	0.00514	0.000218	0.00655%
**OTU_81**	**True**	**OD1**	0.000203	0.0185%	0.0	1	0.00796	0.00299	0.00102	0.000617	0.0185%
**L1427**	**False**	**ZB2**	0.000198	0.0181%	0.0	1	0.0111	0.0695	0.0903	0.000381	0.0114%
**L1408**	**False**	**ZB2**	0.00019	0.0173%	0.0	1	0.00581	0.293	0.313	0.000266	0.00798%
**OTU_4624**	**True**	**PRR-11**	0.000186	0.017%	0.0	1	0.00323	1.32e-05	0.000217	0.000452	0.0136%
**L744**	**False**	**OP3**	0.000185	0.0168%	0.0	1	0.0704	0.00852	0.0118	0.000105	0.00314%
**L987**	**False**	**koll11**	0.000179	0.0163%	0.0	1	0.163	0.0243	0.0342	6.93e-05	0.00208%
**L1233**	**False**	**TM7**	0.000179	0.0163%	0.0	1	0.0252	0.013	0.0488	0.00176	0.0528%
**L1741**	**False**	**OD1**	0.000171	0.0155%	0.0	1	0.0853	0.0239	0.00655	0.000135	0.00405%
**OTU_185**	**True**	**PRR-12**	0.000169	0.0154%	0.0	1	0.0121	0.00205	0.00112	0.000508	0.0152%
**L1237**	**False**	**TM7**	0.000169	0.0154%	0.0	1	0.0241	0.0127	0.0482	0.00166	0.0499%
**L1782**	**False**	**OD1**	0.000168	0.0153%	0.0	1	0.0308	0.0103	0.00244	0.000237	0.00712%
**L457**	**False**	**OP11**	0.000164	0.0149%	0.0	1	0.0352	0.00708	0.00373	0.000104	0.00312%
**OTU_8000**	**True**	**OD1**	0.000154	0.014%	0.0	1	0.00944	0.000483	0.00038	0.000106	0.00318%
**L969**	**False**	**koll11**	0.000141	0.0128%	0.0	1	0.188	0.0283	0.0364	4.91e-05	0.00147%
**L1428**	**False**	**ZB2**	0.000139	0.0127%	0.0	1	0.00961	0.0453	0.0649	0.000266	0.00797%
**L1809**	**False**	**OD1**	0.000138	0.0126%	0.0	1	0.00834	0.00213	0.000169	0.000197	0.00592%
**L1429**	**False**	**ZB2**	0.000138	0.0126%	0.0	1	0.00539	0.0436	0.064	0.000201	0.00604%
**L90**	**False**	**ABY1**	0.000135	0.0123%	0.0	1	0.0206	0.00608	0.000982	0.000124	0.00372%
**L1278**	**False**	**TM7-3**	0.000134	0.0122%	0.0	1	0.0103	0.000317	0.0271	0.000642	0.0193%
**L583**	**False**	**PRR-12**	0.000133	0.0121%	0.0	1	0.012	0.00143	0.000674	0.000143	0.0043%
**L977**	**False**	**koll11**	0.00013	0.0118%	0.0	1	0.172	0.0259	0.0348	4.81e-05	0.00144%
**L785**	**False**	**OP3**	0.000129	0.0118%	0.0	1	0.0559	0.00728	0.00883	9.67e-05	0.0029%
**L423**	**False**	**Bacteria**	0.000128	0.0117%	0.0	1	0.0388	0.0107	0.00834	0.000181	0.00544%
**L1742**	**False**	**OD1**	0.000126	0.0115%	0.0	1	0.0185	0.0057	0.002	0.000159	0.00479%

**L934**	**False**	**koll11**	0.000123	0.0112%	0.0	1	0.239	0.0379	0.0472	4.66e-05	0.0014%
**L1415**	**False**	**ZB2**	0.000123	0.0112%	0.0	1	0.000533	0.0245	0.0641	0.000251	0.00752%
**L52**	**False**	**ABY1**	0.000119	0.0108%	0.0	1	0.00957	0.0412	0.0175	0.000584	0.0175%
**L1539**	**False**	**ZB2**	0.000115	0.0105%	0.0	1	0.0234	0.00394	0.00218	0.000114	0.00342%
**OTU_8178**	**True**	**TM7-1**	0.000115	0.0105%	0.0	1	0.0	0.0	0.00724	0.000289	0.00866%
**L1316**	**False**	**OD1**	0.000115	0.0104%	0.0	1	0.0121	0.00064	0.000398	6.51e-05	0.00195%
**L837**	**False**	**OP3**	0.000114	0.0104%	0.0	1	0.0918	0.0138	0.0149	5.67e-05	0.0017%
**L1797**	**False**	**OD1**	0.000114	0.0104%	0.0	1	0.0268	0.00967	0.00203	0.000184	0.00553%
**L467**	**False**	**OP11-4**	0.000113	0.0103%	0.0	1	0.0257	0.00548	0.00257	0.000123	0.0037%
**L1829**	**False**	**OD1**	0.000112	0.0102%	0.0	1	0.0133	0.00636	0.00152	0.000287	0.00861%
**L1013**	**False**	**koll11**	0.000109	0.00995%	0.0	1	0.0221	0.00426	0.00246	9.49e-05	0.00285%
**L790**	**False**	**OP3**	0.000108	0.00985%	0.0	1	0.0545	0.00719	0.00853	8.23e-05	0.00247%
**L1841**	**False**	**OD1**	0.000107	0.00976%	0.0	1	0.0101	0.00336	0.000654	0.000187	0.00562%
**L441**	**False**	**Bacteria**	0.000104	0.0095%	0.0	1	0.0365	0.0099	0.00497	0.000105	0.00315%
**L1452**	**False**	**ZB2**	0.000104	0.00948%	0.0	1	0.00049	0.0205	0.032	0.000158	0.00473%
**OTU_9143**	**True**	**OD1**	0.000104	0.00946%	0.0	1	0.0	0.00264	0.000223	0.000441	0.0132%
**L1446**	**False**	**ZB2**	0.000102	0.0093%	0.0	1	0.000116	0.0167	0.0229	0.000133	0.004%
**L847**	**False**	**koll11**	0.000102	0.00928%	0.0	1	0.0276	0.00358	0.00463	0.000107	0.0032%
**L581**	**False**	**PRR-12**	0.000101	0.00917%	0.0	1	0.0127	0.00143	0.000674	0.000101	0.00302%
**L171**	**False**	**OD1**	0.0001	0.00911%	0.0	1	0.029	0.00765	0.00349	0.000108	0.00323%
**OTU_6344**	**True**	**OD1**	9.96e-05	0.00907%	0.0	1	0.00623	0.000622	5.18e-05	0.000158	0.00473%
**L1235**	**False**	**TM7**	9.91e-05	0.00902%	0.0	1	0.0248	0.013	0.0488	0.000977	0.0293%
**L1307**	**False**	**OD1**	9.76e-05	0.00888%	0.0	1	0.00548	0.000419	0.000664	0.000142	0.00427%
**L711**	**False**	**PBS-25**	9.75e-05	0.00887%	0.0	1	0.0455	0.00624	0.0133	0.000155	0.00464%
**L578**	**False**	**PRR-12**	9.56e-05	0.0087%	0.0	1	0.0287	0.00375	0.00198	0.000102	0.00306%
**L1743**	**False**	**OD1**	9.42e-05	0.00858%	0.0	1	0.0171	0.00398	0.000155	5.93e-05	0.00178%
**L1497**	**False**	**ZB2**	9.23e-05	0.0084%	0.0	1	0.00146	0.0242	0.0254	0.000367	0.011%
**L51**	**False**	**OD1**	9.15e-05	0.00833%	0.0	1	0.0454	0.0933	0.0459	0.000616	0.0185%
**L1783**	**False**	**OD1**	9.15e-05	0.00833%	0.0	1	0.0306	0.0103	0.00238	0.000129	0.00386%
**L495**	**False**	**OP11-4**	9.09e-05	0.00827%	0.0	1	0.0133	0.00323	0.0017	0.000235	0.00706%
**L31**	**False**	**OD1**	9.02e-05	0.00821%	0.0	1	0.0159	0.00253	0.00186	0.000111	0.00332%
**L709**	**False**	**PBS-25**	8.97e-05	0.00816%	0.0	1	0.0455	0.00624	0.0135	0.000143	0.0043%
**L1010**	**False**	**koll11**	8.88e-05	0.00808%	0.0	1	0.0899	0.0148	0.0238	5.64e-05	0.00169%
**L988**	**False**	**koll11**	8.82e-05	0.00802%	0.0	1	0.155	0.0226	0.0336	3.4e-05	0.00102%
**L1269**	**False**	**TM7-1**	8.77e-05	0.00798%	0.0	1	0.00813	0.0	0.0	0.000207	0.0062%
**L1656**	**False**	**OD1**	8.74e-05	0.00796%	0.0	1	0.00869	0.00319	0.00123	0.000248	0.00744%
**L932**	**False**	**koll11**	8.71e-05	0.00793%	0.0	1	0.239	0.0379	0.0472	3.3e-05	0.000989%
**OTU_9113**	**True**	**TM7-1**	8.66e-05	0.00788%	0.0	1	0.00482	0.0	0.0	0.000239	0.00716%
**OTU_7453**	**True**	**ABY1**	8.63e-05	0.00785%	0.0	1	0.00349	0.000655	0.000208	0.000158	0.00474%
**OTU_5981**	**True**	**ZB2**	8.57e-05	0.0078%	0.0	1	0.000474	0.0196	0.0601	0.000178	0.00533%
**L28**	**False**	**OD1**	8.57e-05	0.0078%	0.0	1	0.142	0.143	0.0792	0.000722	0.0217%
**OTU_6636**	**True**	**ABY1**	8.56e-05	0.00779%	0.0	1	0.00596	0.00229	0.000736	0.000294	0.00882%
**L1009**	**False**	**koll11**	8.55e-05	0.00778%	0.0	1	0.0985	0.0149	0.0241	5.1e-05	0.00153%
**L459**	**False**	**OP11-4**	8.35e-05	0.0076%	0.0	1	0.0264	0.00558	0.0031	8.82e-05	0.00265%
**L1842**	**False**	**OD1**	8.28e-05	0.00754%	0.0	1	0.00492	0.00132	0.000497	0.000189	0.00566%
**L1028**	**False**	**koll11**	7.94e-05	0.00723%	0.0	1	0.033	0.00454	0.0123	0.000106	0.00317%
**L1283**	**False**	**TM7-3**	7.83e-05	0.00713%	0.0	1	0.00036	9.06e-07	0.0238	0.000209	0.00626%
**L1594**	**False**	**OD1**	7.76e-05	0.00707%	0.0	1	0.000224	0.00884	0.00327	0.000164	0.00493%
**L1246**	**False**	**TM7-1**	7.7e-05	0.00701%	0.0	1	2.45e-05	0.0105	0.00754	0.000162	0.00485%
**L1449**	**False**	**ZB2**	7.63e-05	0.00694%	0.0	1	0.00127	0.0256	0.0394	9.9e-05	0.00297%
**L1277**	**False**	**TM7**	7.58e-05	0.0069%	0.0	1	0.011	0.000535	0.0273	0.000372	0.0112%
**L390**	**False**	**ABY1**	7.54e-05	0.00686%	0.0	1	0.00446	0.000429	0.000268	6.48e-05	0.00195%
**L1450**	**False**	**ZB2**	7.44e-05	0.00677%	0.0	1	0.000523	0.0208	0.032	0.000113	0.0034%
**L792**	**False**	**OP3**	7.38e-05	0.00672%	0.0	1	0.0533	0.00697	0.0084	5.66e-05	0.0017%
**L1807**	**False**	**OD1**	7.35e-05	0.00669%	0.0	1	0.011	0.00309	0.000321	0.000108	0.00325%
**L1655**	**False**	**OD1**	7.28e-05	0.00663%	0.0	1	0.0221	0.00657	0.00243	9.31e-05	0.00279%
**L1443**	**False**	**ZB2**	7.2e-05	0.00655%	0.0	1	0.00288	0.0428	0.063	8.95e-05	0.00269%
**L766**	**False**	**OP3**	7.17e-05	0.00653%	0.0	1	0.0598	0.00764	0.00917	4.76e-05	0.00143%
**OTU_7115**	**True**	**ABY1**	7.12e-05	0.00648%	0.0	1	9.62e-05	0.0125	0.0104	0.00017	0.0051%
**OTU_6068**	**True**	**OD1**	7.11e-05	0.00647%	0.0	1	0.00236	0.000157	5.86e-06	0.000104	0.00312%
**L232**	**False**	**ABY1**	7.01e-05	0.00638%	0.0	1	0.0114	0.00276	0.00123	0.000114	0.00341%
**L1777**	**False**	**OD1**	7.01e-05	0.00638%	0.0	1	0.0391	0.0128	0.00285	7.67e-05	0.0023%
**L713**	**False**	**PBS-25**	6.97e-05	0.00635%	0.0	1	0.0451	0.00623	0.0133	0.000112	0.00338%
**L1781**	**False**	**OD1**	6.95e-05	0.00633%	0.0	1	0.0382	0.0128	0.00285	7.95e-05	0.00239%
**L1285**	**False**	**TM7-3**	6.86e-05	0.00624%	0.0	1	0.00036	9.06e-07	0.023	0.000183	0.0055%

**L605**	**False**	**PRR-12**	6.83e-05	0.00622%	0.0	1	0.00374	0.000341	2.96e-06	0.000116	0.00348%
**OTU_6581**	**True**	**ZB2**	6.79e-05	0.00618%	0.0	1	0.000284	0.0205	0.032	9.81e-05	0.00294%
**OTU_9100**	**True**	**TM7**	6.72e-05	0.00612%	0.0	1	1.51e-05	1.27e-06	0.00583	0.000215	0.00646%
**L843**	**False**	**koll11**	6.7e-05	0.0061%	0.0	1	0.0393	0.00429	0.00603	4.93e-05	0.00148%
**L594**	**False**	**PRR-12**	6.62e-05	0.00602%	0.0	1	0.016	0.00229	0.00121	0.000122	0.00365%
**L989**	**False**	**koll11**	6.52e-05	0.00594%	0.0	1	0.155	0.0225	0.0336	2.55e-05	0.000765%
**L1596**	**False**	**OD1**	6.39e-05	0.00582%	0.0	1	0.000224	0.00883	0.00275	0.000119	0.00358%
**L628**	**False**	**PBS-25**	6.27e-05	0.0057%	0.0	1	0.00536	0.000215	7.94e-05	8.42e-05	0.00253%
**OTU_7957**	**True**	**OD1**	6.2e-05	0.00564%	0.0	1	0.00218	0.000711	1.73e-05	0.000141	0.00423%
**L789**	**False**	**OP3**	6.15e-05	0.0056%	0.0	1	0.0547	0.00719	0.00853	4.64e-05	0.00139%
**L360**	**False**	**ABY1**	6.13e-05	0.00558%	0.0	1	0.00528	0.000904	0.000212	0.000101	0.00305%
**OTU_2703**	**True**	**koll11**	6.09e-05	0.00554%	0.0	1	0.0128	0.00178	0.000761	6.52e-05	0.00196%
**L942**	**False**	**koll11**	6.07e-05	0.00552%	0.0	1	0.238	0.0373	0.0471	2.27e-05	0.000683%
**L123**	**False**	**ABY1**	6.01e-05	0.00547%	0.0	1	6.57e-05	0.00946	0.00624	0.000124	0.00372%
**L1843**	**False**	**OD1**	5.98e-05	0.00544%	0.0	1	0.00481	0.00117	0.000494	0.00013	0.00389%
**L767**	**False**	**OP3**	5.95e-05	0.00542%	0.0	1	0.0596	0.00764	0.00917	3.97e-05	0.00119%
**L72**	**False**	**ABY1**	5.93e-05	0.0054%	0.0	1	0.000583	0.0313	0.00889	0.00013	0.00389%
**OTU_260**	**True**	**koll11**	5.93e-05	0.0054%	0.0	1	0.0244	0.00264	0.00183	5.78e-05	0.00173%
**L148**	**False**	**OD1**	5.93e-05	0.0054%	0.0	1	0.00304	0.000574	0.000137	9.28e-05	0.00278%
**L61**	**False**	**ABY1**	5.92e-05	0.00539%	0.0	1	0.00431	0.04	0.0174	0.000185	0.00555%
**L623**	**False**	**PBS-25**	5.81e-05	0.00529%	0.0	1	0.0123	0.00208	0.000434	3.75e-05	0.00113%
**OTU_6197**	**True**	**OD1**	5.72e-05	0.0052%	0.0	1	0.00341	0.000185	0.000455	0.000103	0.0031%
**OTU_347**	**True**	**koll11**	5.68e-05	0.00517%	0.0	1	0.00682	0.000429	0.000813	0.000143	0.00429%
**OTU_8254**	**True**	**OD1**	5.62e-05	0.00512%	0.0	1	0.000777	3.89e-05	1.52e-05	8.43e-05	0.00253%
**L512**	**False**	**OP11-3**	5.51e-05	0.00502%	0.0	1	0.00226	0.000407	5.68e-05	0.000147	0.0044%
**L1011**	**False**	**koll11**	5.51e-05	0.00501%	0.0	1	0.0788	0.0128	0.0207	4.14e-05	0.00124%
**L410**	**False**	**ABY1**	5.51e-05	0.00501%	0.0	1	0.000306	0.00469	0.0065	0.000154	0.00463%
**L1808**	**False**	**OD1**	5.44e-05	0.00496%	0.0	1	0.00896	0.00215	0.000177	7.05e-05	0.00212%
**OTU_10802**	**True**	**OP11-4**	5.41e-05	0.00493%	0.0	1	0.00169	9.87e-05	1.33e-05	6.35e-05	0.00191%
**L1661**	**False**	**OD1**	5.35e-05	0.00487%	0.0	1	0.0134	0.00339	0.00121	7.53e-05	0.00226%
**L733**	**False**	**PBS-25**	5.34e-05	0.00486%	0.0	1	0.02	0.0024	0.00211	6.83e-05	0.00205%
**L1663**	**False**	**OD1**	5.33e-05	0.00485%	0.0	1	0.00672	0.000633	5.18e-05	7.12e-05	0.00214%
**OTU_7960**	**True**	**OD1**	5.31e-05	0.00484%	0.0	1	0.000864	1.04e-05	1.61e-05	8.36e-05	0.00251%
**L648**	**False**	**koll11**	5.27e-05	0.0048%	0.0	1	0.0221	0.00402	0.00503	6.05e-05	0.00181%
**L951**	**False**	**koll11**	5.27e-05	0.0048%	0.0	1	0.0333	0.00569	0.00493	5.88e-05	0.00176%
**OTU_281**	**True**	**koll11**	5.26e-05	0.00479%	0.0	1	0.00597	0.000432	0.00126	0.000117	0.00353%
**L809**	**False**	**OP3**	5.26e-05	0.00478%	0.0	1	0.0393	0.00465	0.00556	4.04e-05	0.00121%
**OTU_8880**	**True**	**ABY1**	5.26e-05	0.00478%	0.0	1	0.00237	0.000128	9.76e-07	7.26e-05	0.00218%
**OTU_6128**	**True**	**ZB2**	5.21e-05	0.00474%	0.0	1	0.00425	0.000472	0.000233	8.41e-05	0.00252%
**L1282**	**False**	**TM7-3**	5.19e-05	0.00473%	0.0	1	0.0103	0.000291	0.0265	0.000259	0.00776%
**OTU_5941**	**True**	**OD1**	5.17e-05	0.00471%	0.0	1	0.00115	0.000783	6.32e-05	0.000255	0.00764%
**L1309**	**False**	**OD1**	5.12e-05	0.00466%	0.0	1	0.00514	0.000419	0.000661	8.03e-05	0.00241%
**L162**	**False**	**ABY1**	5.08e-05	0.00463%	0.0	1	0.00524	0.000518	5.12e-05	9.2e-05	0.00276%
**L760**	**False**	**OP3**	5.08e-05	0.00463%	0.0	1	0.0619	0.00789	0.00929	3.43e-05	0.00103%
**OTU_8200**	**True**	**ABY1**	5.03e-05	0.00457%	0.0	1	0.00164	7.29e-05	8.4e-06	6.28e-05	0.00188%
**L800**	**False**	**OP3**	5.02e-05	0.00457%	0.0	1	0.0521	0.00649	0.00837	3.81e-05	0.00114%
**L81**	**False**	**ABY1**	4.96e-05	0.00451%	0.0	1	0.0224	0.00919	0.00225	6.09e-05	0.00183%
**OTU_8447**	**True**	**ABY1**	4.95e-05	0.0045%	0.0	1	0.000623	0.00601	0.0108	0.000167	0.00501%
**OTU_9114**	**True**	**TM7-1**	4.92e-05	0.00448%	0.0	1	0.0033	0.0	0.0	0.000108	0.00324%
**L1853**	**False**	**OD1**	4.92e-05	0.00448%	0.0	1	0.00738	0.00247	0.000408	5.08e-05	0.00152%
**L184**	**False**	**SM2F11**	4.78e-05	0.00435%	0.0	1	0.0077	0.00167	0.00021	4.12e-05	0.00124%
**L389**	**False**	**ABY1**	4.78e-05	0.00435%	0.0	1	0.0114	0.0038	0.00112	8.3e-05	0.00249%
**L1005**	**False**	**koll11**	4.76e-05	0.00433%	0.0	1	0.106	0.0154	0.0254	2.69e-05	0.000806%
**L1833**	**False**	**OD1**	4.73e-05	0.0043%	0.0	1	7.22e-05	0.00166	0.000125	0.000233	0.00699%
**L1412**	**False**	**ZB2**	4.7e-05	0.00428%	0.0	1	0.000995	0.0246	0.0649	9.63e-05	0.00289%
**OTU_10861**	**True**	**OP11-4**	4.63e-05	0.00422%	0.0	1	0.00522	0.000679	0.000561	6.88e-05	0.00206%
**L47**	**False**	**OD1**	4.6e-05	0.00419%	0.0	1	0.0458	0.0936	0.0459	0.000309	0.00929%
**L481**	**False**	**OP11-4**	4.6e-05	0.00418%	0.0	1	0.0225	0.00498	0.00244	6.38e-05	0.00191%
**L68**	**False**	**ABY1**	4.6e-05	0.00418%	0.0	1	0.00215	0.0379	0.0143	0.000113	0.0034%
**L1166**	**False**	**koll11**	4.53e-05	0.00412%	0.0	1	0.011	0.00437	0.00343	0.000205	0.00616%
**L1567**	**False**	**ZB2**	4.53e-05	0.00412%	0.0	1	0.00314	0.000473	0.000589	6.4e-05	0.00192%
**OTU_7795**	**True**	**ABY1**	4.5e-05	0.00409%	0.0	1	1.14e-05	0.00417	0.00496	0.000109	0.00328%
**L158**	**False**	**ABY1**	4.49e-05	0.00409%	0.0	1	0.00584	0.00062	7.92e-05	7.15e-05	0.00215%
**OTU_7221**	**True**	**ABY1**	4.46e-05	0.00406%	0.0	1	0.00512	0.000186	7.78e-05	3.57e-05	0.00107%
**L320**	**False**	**OD1**	4.43e-05	0.00403%	0.0	1	0.0118	0.00195	0.000849	4.1e-05	0.00123%

**L1543**	**False**	**ZB2**	4.38e-05	0.00399%	0.0	1	0.0231	0.00394	0.00218	4.46e-05	0.00134%
**L366**	**False**	**ABY1**	4.34e-05	0.00395%	0.0	1	0.0049	0.000528	8.29e-05	3.75e-05	0.00112%
**L794**	**False**	**OP3**	4.34e-05	0.00395%	0.0	1	0.0526	0.00689	0.00838	3.36e-05	0.00101%
**L480**	**False**	**OP11-4**	4.31e-05	0.00393%	0.0	1	0.023	0.00502	0.00247	5.69e-05	0.00171%
**L952**	**False**	**koll11**	4.27e-05	0.00389%	0.0	1	0.0328	0.00512	0.00485	4.82e-05	0.00145%
**L579**	**False**	**PRR-12**	4.21e-05	0.00383%	0.0	1	0.0127	0.00146	0.000773	4.31e-05	0.00129%
**L54**	**False**	**ABY1**	4.19e-05	0.00382%	0.0	1	0.00957	0.0408	0.0175	0.000212	0.00636%
**L89**	**False**	**ABY1**	4.09e-05	0.00372%	0.0	1	0.0206	0.00841	0.00224	5.01e-05	0.0015%
**L45**	**False**	**OD1**	4e-05	0.00364%	0.0	1	0.103	0.136	0.076	0.000461	0.0138%
**L807**	**False**	**OP3**	4e-05	0.00364%	0.0	1	0.0463	0.00556	0.00712	3.16e-05	0.000948%
**L1593**	**False**	**OD1**	3.99e-05	0.00363%	0.0	1	0.000224	0.0091	0.00328	7.89e-05	0.00237%
**L1791**	**False**	**OD1**	3.98e-05	0.00363%	0.0	1	0.00253	0.000443	0.000275	5.03e-05	0.00151%
**OTU_7384**	**True**	**ABY1**	3.98e-05	0.00362%	0.0	1	6.57e-05	0.00816	0.006	0.0001	0.00302%
**OTU_6646**	**True**	**ZB2**	3.92e-05	0.00357%	0.0	1	2.15e-05	0.0042	0.00473	9.95e-05	0.00299%
**L511**	**False**	**OP11**	3.92e-05	0.00357%	0.0	1	0.00308	0.000822	6.54e-05	8.8e-05	0.00264%
**L507**	**False**	**OP11-4**	3.9e-05	0.00355%	0.0	1	0.00555	0.000681	0.000561	5.09e-05	0.00153%
**OTU_6705**	**True**	**ZB2**	3.88e-05	0.00353%	0.0	1	2.2e-05	0.00175	0.0047	4.95e-05	0.00149%
**L1500**	**False**	**ZB2**	3.88e-05	0.00353%	0.0	1	0.000362	0.0121	0.00608	0.000218	0.00656%
**OTU_188**	**True**	**koll11**	3.84e-05	0.00349%	0.0	1	0.00649	1.26e-07	0.0	8.53e-05	0.00256%
**L1402**	**False**	**ZB2**	3.84e-05	0.00349%	0.0	1	0.00258	0.05	0.0241	0.000119	0.00356%
**L46**	**False**	**OD1**	3.8e-05	0.00346%	0.0	1	0.0516	0.0943	0.046	0.000271	0.00812%
**OTU_5604**	**True**	**OD1**	3.8e-05	0.00346%	0.0	1	0.0152	0.00372	0.00013	2.7e-05	0.000811%
**L93**	**False**	**ABY1**	3.73e-05	0.00339%	0.0	1	0.019	0.00498	0.000915	3e-05	0.0009%
**L646**	**False**	**koll11**	3.72e-05	0.00339%	0.0	1	0.0221	0.00402	0.00514	4.29e-05	0.00129%
**OTU_314**	**True**	**koll11**	3.72e-05	0.00338%	0.0	1	0.0105	0.00165	0.00331	0.000109	0.00326%
**OTU_6570**	**True**	**SM2F11**	3.71e-05	0.00338%	0.0	1	0.00161	2.07e-05	1.05e-06	3.78e-05	0.00114%
**L597**	**False**	**PRR-12**	3.69e-05	0.00336%	0.0	1	0.00366	0.000242	9.13e-05	3.66e-05	0.0011%
**L1855**	**False**	**OD1**	3.64e-05	0.00331%	0.0	1	0.00683	0.00245	0.000404	4.29e-05	0.00129%
**OTU_10771**	**True**	**OP11-3**	3.62e-05	0.00329%	0.0	1	0.00135	0.0	0.0	7.88e-05	0.00236%
**L1092**	**False**	**koll11**	3.6e-05	0.00327%	0.0	1	0.00992	0.00186	0.00186	6.11e-05	0.00184%
**L1444**	**False**	**ZB2**	3.6e-05	0.00327%	0.0	1	0.00161	0.0172	0.0237	7.03e-05	0.00211%
**L1255**	**False**	**TM7-1**	3.59e-05	0.00327%	0.0	1	0.000115	0.0	0.00724	9.75e-05	0.00293%
**L1459**	**False**	**ZB2**	3.57e-05	0.00325%	0.0	1	0.0	0.000231	0.00178	9.48e-05	0.00285%
**L300**	**False**	**ABY1**	3.55e-05	0.00323%	0.0	1	0.000693	0.00986	0.00586	9.84e-05	0.00295%
**L488**	**False**	**OP11-4**	3.54e-05	0.00322%	0.0	1	0.00732	0.00153	0.000723	4.2e-05	0.00126%
**L260**	**False**	**OD1**	3.52e-05	0.0032%	0.0	1	0.0126	0.0134	0.00599	0.000417	0.0125%
**L984**	**False**	**koll11**	3.48e-05	0.00317%	0.0	1	0.00545	0.000735	0.000162	5.79e-05	0.00174%
**L1426**	**False**	**OD1**	3.48e-05	0.00316%	0.0	1	0.0244	0.0729	0.0965	0.000112	0.00337%
**L359**	**False**	**OD1**	3.46e-05	0.00315%	0.0	1	0.00615	0.00092	0.000213	4.8e-05	0.00144%
**L811**	**False**	**OP3**	3.44e-05	0.00313%	0.0	1	0.0392	0.00462	0.00556	2.65e-05	0.000795%
**L140**	**False**	**ABY1**	3.39e-05	0.00309%	0.0	1	0.0042	0.0169	0.0056	0.000236	0.00708%
**OTU_7302**	**True**	**ABY1**	3.39e-05	0.00309%	0.0	1	0.00417	0.000979	0.000116	6.8e-05	0.00204%
**L803**	**False**	**OP3**	3.39e-05	0.00308%	0.0	1	0.0463	0.00581	0.00741	2.77e-05	0.000831%
**L585**	**False**	**PRR-12**	3.37e-05	0.00307%	0.0	1	0.0119	0.00143	0.00065	3.73e-05	0.00112%
**OTU_7971**	**True**	**OD1**	3.34e-05	0.00304%	0.0	1	0.000898	0.000107	3.21e-05	6.7e-05	0.00201%
**L1546**	**False**	**ZB2**	3.31e-05	0.00301%	0.0	1	0.0212	0.00376	0.00178	3.44e-05	0.00103%
**L1885**	**False**	**OD1**	3.29e-05	0.003%	0.0	1	0.00372	0.000925	0.00018	3.82e-05	0.00115%
**OTU_380**	**True**	**koll11**	3.27e-05	0.00297%	0.0	1	0.00362	0.000575	3.23e-05	5.82e-05	0.00175%
**L1230**	**False**	**[Acetothermia]**	3.27e-05	0.00297%	0.0	1	0.000697	2.53e-05	0.0	7.07e-05	0.00212%
**L1831**	**False**	**OD1**	3.26e-05	0.00297%	0.0	1	0.0121	0.00558	0.00145	8.34e-05	0.0025%
**L434**	**False**	**WCHB1-64**	3.26e-05	0.00296%	0.0	1	0.000383	0.000126	0.00163	0.000134	0.00401%
**OTU_7053**	**True**	**ABY1**	3.16e-05	0.00288%	0.0	1	0.00485	0.000904	0.000188	6.15e-05	0.00184%
**L624**	**False**	**PBS-25**	3.12e-05	0.00284%	0.0	1	0.00612	0.00186	0.000355	3.88e-05	0.00117%
**L1112**	**False**	**koll11**	3.12e-05	0.00284%	0.0	1	0.0253	0.0041	0.00398	2.48e-05	0.000744%
**OTU_186**	**True**	**koll11**	3.12e-05	0.00284%	0.0	1	0.0219	0.00374	0.00248	2.45e-05	0.000735%
**L1689**	**False**	**OD1**	3.07e-05	0.00279%	0.0	1	0.00812	0.00137	0.000277	2.24e-05	0.000672%
**L1677**	**False**	**OD1**	3.06e-05	0.00279%	0.0	1	0.00342	0.000578	0.000289	6.28e-05	0.00188%
**L1827**	**False**	**OD1**	3.05e-05	0.00278%	0.0	1	0.0134	0.00636	0.00152	7.77e-05	0.00233%
**L321**	**False**	**OD1**	3.04e-05	0.00277%	0.0	1	0.0113	0.00192	0.000846	2.91e-05	0.000874%
**L1027**	**False**	**koll11**	3.04e-05	0.00277%	0.0	1	0.0441	0.00641	0.0141	2.8e-05	0.00084%
**L1259**	**False**	**TM7-1**	3.04e-05	0.00277%	0.0	1	0.0102	0.00135	0.000289	8.52e-05	0.00256%
**L1720**	**False**	**Mb-NB09**	3.03e-05	0.00276%	0.0	1	0.0065	0.00171	0.00076	7.03e-05	0.00211%
**OTU_242**	**True**	**PBS-25**	3.02e-05	0.00275%	0.0	1	0.0126	0.00123	0.000834	4.86e-05	0.00146%
**L1335**	**False**	**ZB2**	3.02e-05	0.00275%	0.0	1	0.00344	0.000828	0.000267	4.14e-05	0.00124%
**L63**	**False**	**ABY1**	3.02e-05	0.00274%	0.0	1	0.00431	0.0398	0.0173	9.43e-05	0.00283%

**L192**	**False**	**ABY1**	3.01e-05	0.00274%	0.0	1	0.0178	0.00554	0.00304	5.5e-05	0.00165%
**L97**	**False**	**ABY1**	3e-05	0.00273%	0.0	1	0.0114	0.00236	0.000344	2.72e-05	0.000816%
**OTU_229**	**True**	**koll11**	2.97e-05	0.0027%	0.0	1	0.00773	0.00108	0.000381	5.76e-05	0.00173%
**OTU_7992**	**True**	**TM7-1**	2.96e-05	0.0027%	0.0	1	2.45e-05	0.0103	0.00754	6.32e-05	0.0019%
**OTU_83**	**True**	**Mb-NB09**	2.95e-05	0.00269%	0.0	1	0.000562	0.000111	3.67e-05	7.12e-05	0.00214%
**OTU_6978**	**True**	**ZB2**	2.94e-05	0.00268%	0.0	1	0.00217	0.000205	2.37e-05	3.77e-05	0.00113%
**L816**	**False**	**PBS-25**	2.92e-05	0.00266%	0.0	1	0.0227	0.00265	0.00186	2.41e-05	0.000724%
**OTU_183**	**True**	**koll11**	2.92e-05	0.00265%	0.0	1	0.00758	0.00152	0.000571	6.17e-05	0.00185%
**L1287**	**False**	**EW055**	2.9e-05	0.00264%	0.0	1	0.000344	9.06e-07	0.0127	8.15e-05	0.00244%
**L842**	**False**	**OP3**	2.87e-05	0.00261%	0.0	1	0.0616	0.00686	0.00793	1.52e-05	0.000455%
**L12**	**False**	**OD1**	2.86e-05	0.0026%	0.0	1	0.00155	0.000939	5.45e-06	0.000101	0.00304%
**L278**	**False**	**ABY1**	2.84e-05	0.00258%	0.0	1	0.00308	0.00149	7.84e-05	7.49e-05	0.00225%
**L649**	**False**	**koll11**	2.83e-05	0.00258%	0.0	1	0.0218	0.00395	0.0049	3.28e-05	0.000986%
**OTU_7002**	**True**	**ZB2**	2.82e-05	0.00256%	0.0	1	0.00077	0.0	0.0	5.8e-05	0.00174%
**OTU_172**	**True**	**PBS-25**	2.82e-05	0.00256%	0.0	1	0.0125	0.00187	0.00787	0.000117	0.00351%
**L1561**	**False**	**ZB2**	2.81e-05	0.00256%	0.0	1	0.00489	0.000523	0.000233	4.48e-05	0.00134%
**L822**	**False**	**PBS-25**	2.81e-05	0.00256%	0.0	1	0.0216	0.00237	0.00186	2.32e-05	0.000696%
**L1697**	**False**	**Mb-NB09**	2.81e-05	0.00256%	0.0	1	0.000885	0.00015	4.37e-05	4.68e-05	0.00141%
**L347**	**False**	**ABY1**	2.79e-05	0.00254%	0.0	1	0.00447	0.000692	0.000222	3.82e-05	0.00115%
**OTU_1080**	**True**	**koll11**	2.79e-05	0.00254%	0.0	1	0.00842	0.000176	0.000609	3.99e-05	0.0012%
**L30**	**False**	**OD1**	2.79e-05	0.00254%	0.0	1	0.119	0.14	0.0781	0.000295	0.00886%
**L1536**	**False**	**OD1**	2.73e-05	0.00248%	0.0	1	0.00393	0.000265	0.000547	4.27e-05	0.00128%
**OTU_1400**	**True**	**PBS-25**	2.72e-05	0.00248%	0.0	1	0.014	0.00127	0.00101	2.99e-05	0.000897%
**L233**	**False**	**ABY1**	2.72e-05	0.00248%	0.0	1	0.00487	0.00129	0.000246	6.75e-05	0.00202%
**L1413**	**False**	**ZB2**	2.69e-05	0.00245%	0.0	1	0.000533	0.0245	0.0642	5.48e-05	0.00164%
**OTU_303**	**True**	**koll11**	2.68e-05	0.00244%	0.0	1	0.00936	0.00173	0.00159	4.8e-05	0.00144%
**L1110**	**False**	**koll11**	2.67e-05	0.00243%	0.0	1	0.0352	0.00481	0.00527	1.3e-05	0.00039%
**L972**	**False**	**koll11**	2.66e-05	0.00242%	0.0	1	0.186	0.0277	0.0362	9.58e-06	0.000288%
**L1004**	**False**	**koll11**	2.64e-05	0.0024%	0.0	1	0.141	0.0202	0.0307	1.08e-05	0.000323%
**L391**	**False**	**ABY1**	2.63e-05	0.0024%	0.0	1	0.00346	0.000153	0.000136	2.53e-05	0.000759%
**L186**	**False**	**SM2F11**	2.63e-05	0.00239%	0.0	1	0.00517	0.0011	0.000185	3.73e-05	0.00112%
**OTU_8054**	**True**	**ABY1**	2.62e-05	0.00239%	0.0	1	0.00128	0.000162	1.89e-05	6.07e-05	0.00182%
**OTU_633**	**True**	**koll11**	2.6e-05	0.00237%	0.0	1	0.0201	0.00197	0.00172	1.43e-05	0.00043%
**L32**	**False**	**ABY1**	2.58e-05	0.00235%	0.0	1	0.00131	2.41e-05	1.78e-05	3.43e-05	0.00103%
**L1544**	**False**	**ZB2**	2.58e-05	0.00234%	0.0	1	0.0217	0.00377	0.00179	2.59e-05	0.000779%
**L1872**	**False**	**OD1**	2.56e-05	0.00233%	0.0	1	0.00108	0.00135	5.3e-05	0.000108	0.00324%
**L1640**	**False**	**ZB2**	2.56e-05	0.00233%	0.0	1	6.26e-06	0.0018	0.00199	0.000183	0.00549%
**L1806**	**False**	**OD1**	2.55e-05	0.00232%	0.0	1	0.0122	0.00329	0.00048	3.83e-05	0.00115%
**OTU_5705**	**True**	**OD1**	2.55e-05	0.00232%	0.0	1	0.0105	0.0021	0.000981	4.69e-05	0.00141%
**OTU_180**	**True**	**koll11**	2.54e-05	0.00232%	0.0	1	0.00541	0.000699	0.000339	4.07e-05	0.00122%
**L815**	**False**	**OP3**	2.51e-05	0.00229%	0.0	1	0.0238	0.00268	0.00186	1.87e-05	0.000561%
**L424**	**False**	**OP11**	2.51e-05	0.00228%	0.0	1	0.00231	0.000816	0.00336	0.000182	0.00547%
**L812**	**False**	**PBS-25**	2.49e-05	0.00227%	0.0	1	0.0154	0.00195	0.0037	4.3e-05	0.00129%
**L333**	**False**	**OD1**	2.44e-05	0.00222%	0.0	1	0.00147	0.000345	5.45e-05	4.96e-05	0.00149%
**L660**	**False**	**koll11**	2.42e-05	0.0022%	0.0	1	0.00367	0.000779	0.000478	5.71e-05	0.00171%
**OTU_6203**	**True**	**OD1**	2.41e-05	0.0022%	0.0	1	5.84e-05	0.00862	0.00232	3.97e-05	0.00119%
**L845**	**False**	**koll11**	2.41e-05	0.00219%	0.0	1	0.0309	0.00412	0.00542	2.5e-05	0.00075%
**L1167**	**False**	**koll11**	2.41e-05	0.00219%	0.0	1	0.0106	0.00409	0.003	0.000103	0.00309%
**L1716**	**False**	**Mb-NB09**	2.39e-05	0.00218%	0.0	1	0.00232	0.000602	8.96e-05	6.33e-05	0.0019%
**L1049**	**False**	**koll11**	2.36e-05	0.00215%	0.0	1	0.00374	0.000575	3.23e-05	4e-05	0.0012%
**OTU_5994**	**True**	**ZB2**	2.36e-05	0.00214%	0.0	1	0.000403	0.0369	0.0185	6.8e-05	0.00204%
**OTU_85**	**True**	**OD1**	2.33e-05	0.00212%	0.0	1	0.000258	2.98e-05	0.0	6.78e-05	0.00204%
**L1097**	**False**	**koll11**	2.31e-05	0.0021%	0.0	1	0.00966	0.00172	0.00255	4.12e-05	0.00124%
**L1177**	**False**	**koll11**	2.31e-05	0.0021%	0.0	1	0.00868	0.00219	0.00101	3.89e-05	0.00117%
**OTU_9009**	**True**	**Mb-NB09**	2.31e-05	0.0021%	0.0	1	0.000847	5.62e-06	2.1e-05	5.81e-05	0.00174%
**OTU_3932**	**True**	**SJA-4**	2.29e-05	0.00209%	0.0	1	0.00144	0.0	0.0	5.33e-05	0.0016%
**L1550**	**False**	**ZB2**	2.29e-05	0.00209%	0.0	1	0.0187	0.00347	0.00167	2.67e-05	0.000802%
**L1658**	**False**	**OD1**	2.29e-05	0.00209%	0.0	1	0.00856	0.00316	0.00122	6.69e-05	0.00201%
**OTU_297**	**True**	**koll11**	2.29e-05	0.00208%	0.0	1	0.00756	0.000441	0.000215	4.18e-05	0.00125%
**OTU_8240**	**True**	**ABY1**	2.28e-05	0.00208%	0.0	1	0.00151	0.000241	0.000462	8.99e-05	0.0027%
**OTU_2037**	**True**	**koll11**	2.28e-05	0.00207%	0.0	1	0.00887	0.0012	0.000932	3.3e-05	0.00099%
**OTU_10762**	**True**	**OP11-4**	2.27e-05	0.00207%	0.0	1	0.00112	0.000204	1.44e-05	2.58e-05	0.000774%
**L1712**	**False**	**Mb-NB09**	2.26e-05	0.00206%	0.0	1	0.00333	0.000792	0.00014	4.48e-05	0.00134%
**L165**	**False**	**ABY1**	2.24e-05	0.00204%	0.0	1	0.00511	0.000484	4.5e-05	4.13e-05	0.00124%
**L193**	**False**	**ABY1**	2.22e-05	0.00202%	0.0	1	0.00628	0.00279	0.00181	9.6e-05	0.00288%

**L92**	**False**	**ABY1**	2.21e-05	0.00202%	0.0	1	0.0197	0.0055	0.000976	1.92e-05	0.000578%
**OTU_9036**	**True**	**Mb-NB09**	2.18e-05	0.00198%	0.0	1	0.000593	0.0	0.0	4.73e-05	0.00142%
**OTU_6056**	**True**	**ABY1**	2.17e-05	0.00197%	0.0	1	0.00458	0.000412	1.46e-05	4.38e-05	0.00131%
**OTU_8751**	**True**	**OD1**	2.16e-05	0.00197%	0.0	1	0.00308	0.000199	0.000588	4.04e-05	0.00121%
**L419**	**False**	**Bacteria**	2.16e-05	0.00196%	0.0	1	0.836	0.845	0.913	0.000164	0.00493%
**L64**	**False**	**ABY1**	2.15e-05	0.00196%	0.0	1	0.0043	0.0381	0.0144	7.14e-05	0.00214%
**L1312**	**False**	**OD1**	2.14e-05	0.00195%	0.0	1	0.000552	0.000107	0.0	5.26e-05	0.00158%
**OTU_6006**	**True**	**OD1**	2.14e-05	0.00194%	0.0	1	1.83e-05	0.0015	0.0	7.65e-05	0.0023%
**L29**	**False**	**OD1**	2.13e-05	0.00194%	0.0	1	0.137	0.142	0.0791	0.00019	0.00571%
**L271**	**False**	**OD1**	2.13e-05	0.00193%	0.0	1	0.00471	0.007	0.00301	0.000531	0.0159%
**OTU_258**	**True**	**koll11**	2.12e-05	0.00193%	0.0	1	0.00778	0.00194	0.000805	3.59e-05	0.00108%
**OTU_8031**	**True**	**ABY1**	2.11e-05	0.00192%	0.0	1	0.00406	0.00203	0.000331	5.32e-05	0.0016%
**OTU_62**	**True**	**ZB2**	2.1e-05	0.00191%	0.0	1	0.000179	0.0111	0.00605	0.000124	0.00371%
**L1514**	**False**	**OD1**	2.09e-05	0.00191%	0.0	1	0.00236	0.000168	0.00102	9.07e-05	0.00272%
**OTU_8002**	**True**	**ABY1**	2.07e-05	0.00189%	0.0	1	0.00119	1.69e-05	1.49e-05	4.51e-05	0.00135%
**OTU_2281**	**True**	**koll11**	2.06e-05	0.00188%	0.0	1	0.00253	8.61e-05	0.000278	3.56e-05	0.00107%
**L1784**	**False**	**OD1**	2.06e-05	0.00188%	0.0	1	0.00379	0.000639	0.000353	2.36e-05	0.000709%
**OTU_689**	**True**	**koll11**	2.05e-05	0.00187%	0.0	1	0.0038	0.00104	0.000831	4.19e-05	0.00126%
**L528**	**False**	**TM6**	2.04e-05	0.00186%	0.0	1	0.00643	0.00365	0.00278	0.000365	0.011%
**OTU_4160**	**True**	**GIF10**	2.04e-05	0.00185%	0.0	1	0.000693	3.56e-07	0.0	4.25e-05	0.00128%
**L248**	**False**	**ABY1**	2.03e-05	0.00185%	0.0	1	0.00564	0.00137	0.000155	2.73e-05	0.000821%
**L1690**	**False**	**OD1**	2.01e-05	0.00183%	0.0	1	0.00467	0.00056	0.000137	1.19e-05	0.000357%
**L856**	**False**	**OP3**	2.01e-05	0.00183%	0.0	1	0.0205	0.00214	0.00181	1.12e-05	0.000335%
**OTU_6723**	**True**	**OD1**	2e-05	0.00182%	0.0	1	6.32e-07	0.00104	0.00025	7.44e-05	0.00223%
**L1512**	**False**	**OD1**	1.99e-05	0.00182%	0.0	1	0.00656	0.00145	0.00446	9.97e-05	0.00299%
**L1683**	**False**	**Mb-NB09**	1.99e-05	0.00181%	0.0	1	0.00263	0.00082	5.39e-05	4.26e-05	0.00128%
**OTU_6014**	**True**	**SM2F11**	1.99e-05	0.00181%	0.0	1	0.00469	0.00101	0.000185	2.93e-05	0.00088%
**OTU_8175**	**True**	**TM7-3**	1.99e-05	0.00181%	0.0	1	1.5e-05	0.0	0.0103	5.02e-05	0.00151%
**L4**	**False**	**ABY1**	1.99e-05	0.00181%	0.0	1	0.00224	0.000634	0.000127	3.36e-05	0.00101%
**OTU_49**	**True**	**OD1**	1.98e-05	0.0018%	0.0	1	0.000262	1.92e-05	0.0	4.27e-05	0.00128%
**L141**	**False**	**ABY1**	1.97e-05	0.00179%	0.0	1	0.00175	0.0154	0.00536	9e-05	0.0027%
**L1244**	**False**	**TM7-1**	1.96e-05	0.00179%	0.0	1	0.0015	0.0106	0.0148	8.02e-05	0.00241%
**OTU_7054**	**True**	**ABY1**	1.96e-05	0.00178%	0.0	1	0.00326	0.000456	7.46e-05	2.59e-05	0.000776%
**L175**	**False**	**SM2F11**	1.93e-05	0.00176%	0.0	1	0.00161	0.000188	1.28e-05	2.89e-05	0.000867%
**L1558**	**False**	**ZB2**	1.93e-05	0.00176%	0.0	1	0.0133	0.00213	0.00112	2.08e-05	0.000624%
**L403**	**False**	**ABY1**	1.93e-05	0.00175%	0.0	1	0.0069	0.00283	0.000848	6.05e-05	0.00182%
**OTU_4910**	**True**	**OD1**	1.92e-05	0.00175%	0.0	1	0.000498	0.000218	0.000106	0.000167	0.00501%
**L1456**	**False**	**ZB2**	1.91e-05	0.00174%	0.0	1	0.000338	0.00463	0.00735	3.88e-05	0.00116%
**OTU_6129**	**True**	**SM2F11**	1.91e-05	0.00174%	0.0	1	0.00253	0.000568	2.44e-05	3.79e-05	0.00114%
**L1261**	**False**	**TM7-1**	1.89e-05	0.00172%	0.0	1	0.0102	0.000594	5.57e-05	3.93e-05	0.00118%
**L297**	**False**	**ABY1**	1.89e-05	0.00172%	0.0	1	0.000892	0.00999	0.00688	5.51e-05	0.00165%
**L1336**	**False**	**ZB2**	1.89e-05	0.00172%	0.0	1	0.00181	0.000132	9.32e-05	2.1e-05	0.000629%
**L137**	**False**	**OD1**	1.89e-05	0.00172%	0.0	1	0.008	0.0303	0.0164	0.00011	0.00329%
**L323**	**False**	**OD1**	1.87e-05	0.0017%	0.0	1	0.00479	0.000834	0.000142	2.11e-05	0.000633%
**L94**	**False**	**ABY1**	1.87e-05	0.0017%	0.0	1	0.0151	0.0043	0.000578	1.88e-05	0.000563%
**L1104**	**False**	**koll11**	1.86e-05	0.00169%	0.0	1	0.00723	7.61e-05	0.000316	4.31e-05	0.00129%
**L725**	**False**	**PBS-25**	1.86e-05	0.00169%	0.0	1	0.0277	0.00349	0.00486	2.83e-05	0.00085%
**OTU_3387**	**True**	**PBS-25**	1.85e-05	0.00169%	0.0	1	0.00163	0.0	0.0	5.16e-05	0.00155%
**L1790**	**False**	**OD1**	1.85e-05	0.00169%	0.0	1	0.00291	0.000443	0.00028	1.93e-05	0.000579%
**L58**	**False**	**ABY1**	1.85e-05	0.00168%	0.0	1	0.00665	0.0407	0.0175	6.98e-05	0.0021%
**L322**	**False**	**OD1**	1.84e-05	0.00168%	0.0	1	0.00485	0.000892	0.000149	2.13e-05	0.00064%
**OTU_8035**	**True**	**OD1**	1.83e-05	0.00167%	0.0	1	0.00144	0.000165	0.00038	8.32e-05	0.0025%
**OTU_6576**	**True**	**ZB2**	1.83e-05	0.00166%	0.0	1	0.000496	1.15e-05	0.0	2.66e-05	0.000799%
**OTU_2093**	**True**	**GIF10**	1.83e-05	0.00166%	0.0	1	0.00261	0.000361	0.00011	3.85e-05	0.00116%
**L613**	**False**	**BD4-9**	1.82e-05	0.00166%	0.0	1	0.000643	0.000132	0.0	5.5e-05	0.00165%
**L485**	**False**	**OP11-4**	1.82e-05	0.00166%	0.0	1	0.00169	0.000156	1.33e-05	2.37e-05	0.000712%
**OTU_5431**	**True**	**OD1**	1.82e-05	0.00166%	0.0	1	0.00055	2.4e-05	3.64e-06	3.57e-05	0.00107%
**OTU_8044**	**True**	**WCHB1**	1.82e-05	0.00165%	0.0	1	0.000531	1.53e-05	0.0	2.54e-05	0.000763%
**L974**	**False**	**koll11**	1.81e-05	0.00165%	0.0	1	0.00543	0.000817	0.000339	2.98e-05	0.000894%
**L520**	**False**	**OP11-3**	1.8e-05	0.00164%	0.0	1	0.00141	0.0	0.0	3.7e-05	0.00111%
**OTU_270**	**True**	**koll11**	1.8e-05	0.00164%	0.0	1	0.00125	1.74e-05	7.47e-06	2.9e-05	0.000871%
**OTU_7950**	**True**	**ABY1**	1.8e-05	0.00164%	0.0	1	0.00112	0.000191	0.000133	5.48e-05	0.00164%
**L874**	**False**	**koll11**	1.8e-05	0.00163%	0.0	1	0.0199	0.00567	0.00568	3.85e-05	0.00115%
**L1793**	**False**	**OD1**	1.8e-05	0.00163%	0.0	1	0.00176	0.000404	0.00026	3.8e-05	0.00114%

**OTU_423**	**True**	**PBS-25**	1.79e-05	0.00163%	0.0	1	0.0058	0.00185	0.000355	2.43e-05	0.000731%
**OTU_9039**	**True**	**ABY1**	1.79e-05	0.00163%	0.0	1	0.00096	8.9e-06	2.8e-06	3.07e-05	0.000921%
**L1365**	**False**	**ZB2**	1.78e-05	0.00162%	0.0	1	2.03e-06	0.00133	0.0013	6.54e-05	0.00196%
**L1290**	**False**	**TM7-3**	1.76e-05	0.0016%	0.0	1	0.0099	0.000291	0.00271	6.16e-05	0.00185%
**OTU_949**	**True**	**koll11**	1.76e-05	0.0016%	0.0	1	0.00466	0.000702	0.00014	2.72e-05	0.000816%
**OTU_6683**	**True**	**ZB2**	1.75e-05	0.00159%	0.0	1	0.000959	0.000139	6.15e-05	4.57e-05	0.00137%
**OTU_36**	**True**	**PBS-25**	1.75e-05	0.00159%	0.0	1	0.00285	0.000163	0.000189	4.25e-05	0.00127%
**OTU_927**	**True**	**PRR-12**	1.75e-05	0.00159%	0.0	1	0.00168	3.93e-05	1.25e-05	2.24e-05	0.000672%
**L1533**	**False**	**OD1**	1.74e-05	0.00159%	0.0	1	3.42e-06	0.000367	0.000957	4.93e-05	0.00148%
**L992**	**False**	**koll11**	1.74e-05	0.00158%	0.0	1	0.0103	0.00162	0.002	3.41e-05	0.00102%
**OTU_7898**	**True**	**ABY1**	1.73e-05	0.00158%	0.0	1	1.39e-05	0.00206	0.00203	6.61e-05	0.00198%
**L393**	**False**	**ABY1**	1.73e-05	0.00158%	0.0	1	0.00322	0.000134	0.000136	1.66e-05	0.000497%
**L1509**	**False**	**OD1**	1.72e-05	0.00157%	0.0	1	0.00859	0.00246	0.00461	7.8e-05	0.00234%
**OTU_216**	**True**	**koll11**	1.72e-05	0.00157%	0.0	1	0.00864	0.00155	0.00232	3.67e-05	0.0011%
**OTU_8186**	**True**	**OD1**	1.72e-05	0.00156%	0.0	1	0.00169	7.06e-07	2.8e-06	3.82e-05	0.00115%
**L595**	**False**	**PRR-12**	1.71e-05	0.00156%	0.0	1	0.00387	0.000242	9.13e-05	1.6e-05	0.00048%
**L5**	**False**	**ABY1**	1.69e-05	0.00154%	0.0	1	0.000863	4.87e-05	0.000119	2.52e-05	0.000755%
**OTU_7450**	**True**	**ABY1**	1.69e-05	0.00154%	0.0	1	0.00294	0.000573	0.000137	2.85e-05	0.000856%
**L1146**	**False**	**koll11**	1.69e-05	0.00154%	0.0	1	0.00825	0.000955	0.00105	4e-05	0.0012%
**OTU_8065**	**True**	**ABY1**	1.68e-05	0.00153%	0.0	1	3.16e-06	0.000535	0.0	4.38e-05	0.00132%
**OTU_214**	**True**	**PBS-25**	1.68e-05	0.00153%	0.0	1	0.00584	0.00088	0.00142	4.29e-05	0.00129%
**L1592**	**False**	**OD1**	1.68e-05	0.00153%	0.0	1	0.00167	0.00918	0.00373	7.81e-05	0.00234%
**OTU_8251**	**True**	**Mb-NB09**	1.67e-05	0.00152%	0.0	1	0.00232	0.000444	7.99e-05	4.03e-05	0.00121%
**OTU_2374**	**True**	**koll11**	1.67e-05	0.00152%	0.0	1	0.000891	2.06e-06	4.88e-07	3.2e-05	0.000961%
**L1511**	**False**	**OD1**	1.66e-05	0.00152%	0.0	1	0.00859	0.00211	0.00454	6.66e-05	0.002%
**OTU_8134**	**True**	**ZB2**	1.66e-05	0.00151%	0.0	1	0.000484	0.0	0.0	3.48e-05	0.00104%
**L261**	**False**	**OD1**	1.66e-05	0.00151%	0.0	1	0.0125	0.0117	0.00496	0.000164	0.00493%
**L1597**	**False**	**OD1**	1.66e-05	0.00151%	0.0	1	0.000205	0.00872	0.00274	3.12e-05	0.000936%
**L1771**	**False**	**OD1**	1.66e-05	0.00151%	0.0	1	0.00133	0.000285	5.7e-05	4.5e-05	0.00135%
**L1798**	**False**	**OD1**	1.65e-05	0.0015%	0.0	1	0.0134	0.00331	0.00051	2.24e-05	0.000674%
**L523**	**False**	**OP11**	1.65e-05	0.0015%	0.0	1	0.000827	0.000415	8.63e-06	6.67e-05	0.002%
**L1157**	**False**	**koll11**	1.64e-05	0.0015%	0.0	1	0.00606	0.000872	0.00466	9.22e-05	0.00277%
**OTU_10738**	**True**	**OP11-3**	1.64e-05	0.00149%	0.0	1	0.000411	7.06e-06	5.29e-07	4.02e-05	0.00121%
**OTU_6571**	**True**	**ZB2**	1.64e-05	0.00149%	0.0	1	0.00203	0.0	5.92e-05	2.31e-05	0.000693%
**L76**	**False**	**ABY1**	1.64e-05	0.00149%	0.0	1	0.00195	0.000689	6.24e-05	5.12e-05	0.00154%
**L405**	**False**	**ABY1**	1.64e-05	0.00149%	0.0	1	0.00624	0.0023	0.000746	5.14e-05	0.00154%
**L482**	**False**	**OP11-4**	1.63e-05	0.00149%	0.0	1	0.00921	0.00175	0.000739	1.41e-05	0.000423%
**L1080**	**False**	**koll11**	1.63e-05	0.00149%	0.0	1	0.0085	0.000739	0.000316	2.87e-05	0.00086%
**L1523**	**False**	**ZB2**	1.63e-05	0.00148%	0.0	1	0.00191	0.000663	7.98e-05	3.68e-05	0.0011%
**OTU_7120**	**True**	**ABY1**	1.62e-05	0.00147%	0.0	1	0.00226	0.000889	9.59e-05	4.7e-05	0.00141%
**L99**	**False**	**ABY1**	1.61e-05	0.00147%	0.0	1	0.00965	0.00166	0.000249	1.76e-05	0.000528%
**OTU_6061**	**True**	**ZB2**	1.61e-05	0.00146%	0.0	1	2.54e-05	0.00154	9.63e-05	3.82e-05	0.00115%
**L1786**	**False**	**OD1**	1.6e-05	0.00146%	0.0	1	0.0036	0.000443	0.000295	1.58e-05	0.000475%
**L1018**	**False**	**koll11**	1.59e-05	0.00145%	0.0	1	0.0119	0.00196	0.00183	3.1e-05	0.000931%
**L1254**	**False**	**TM7-1**	1.59e-05	0.00145%	0.0	1	0.000307	3.44e-06	0.00727	4.62e-05	0.00139%
**L1178**	**False**	**koll11**	1.59e-05	0.00145%	0.0	1	0.00578	0.000586	0.000364	1.51e-05	0.000452%
**L868**	**False**	**koll11**	1.58e-05	0.00144%	0.0	1	0.0207	0.0057	0.00569	3.11e-05	0.000932%
**L1662**	**False**	**OD1**	1.58e-05	0.00144%	0.0	1	0.00967	0.00245	0.000611	2.45e-05	0.000737%
**L864**	**False**	**koll11**	1.57e-05	0.00143%	0.0	1	0.0212	0.00575	0.00584	2.92e-05	0.000876%
**OTU_7263**	**True**	**ABY1**	1.57e-05	0.00143%	0.0	1	0.0035	0.000588	0.000155	1.39e-05	0.000416%
**OTU_400**	**True**	**koll11**	1.57e-05	0.00143%	0.0	1	0.00145	0.000117	0.000248	4.77e-05	0.00143%
**L309**	**False**	**ABY1**	1.56e-05	0.00142%	0.0	1	3.1e-05	0.0037	0.00312	2.78e-05	0.000835%
**L59**	**False**	**ABY1**	1.56e-05	0.00142%	0.0	1	0.00469	0.04	0.0174	5.02e-05	0.00151%
**L994**	**False**	**koll11**	1.56e-05	0.00142%	0.0	1	0.0102	0.00161	0.00199	3.09e-05	0.000927%
**OTU_261**	**True**	**koll11**	1.54e-05	0.0014%	0.0	1	0.00135	0.000108	0.0	3.29e-05	0.000989%
**L1865**	**False**	**OD1**	1.53e-05	0.00139%	0.0	1	0.00142	0.000153	0.000152	2.09e-05	0.000628%
**OTU_8038**	**True**	**ABY1**	1.53e-05	0.00139%	0.0	1	0.000825	6.38e-06	1.05e-06	3.04e-05	0.000912%
**L1117**	**False**	**koll11**	1.52e-05	0.00139%	0.0	1	0.00545	0.00041	0.00094	1.68e-05	0.000506%
**L1528**	**False**	**OD1**	1.52e-05	0.00138%	0.0	1	0.00477	0.000976	0.00166	4.07e-05	0.00122%
**L1111**	**False**	**koll11**	1.52e-05	0.00138%	0.0	1	0.0308	0.00451	0.00492	8.13e-06	0.000244%
**L174**	**False**	**SM2F11**	1.51e-05	0.00137%	0.0	1	0.00211	0.000415	0.000198	2.5e-05	0.00075%
**L730**	**False**	**PBS-25**	1.5e-05	0.00137%	0.0	1	0.0071	0.000872	0.00263	5.43e-05	0.00163%
**L1714**	**False**	**Mb-NB09**	1.5e-05	0.00137%	0.0	1	0.0028	0.000608	0.000108	3.17e-05	0.000951%
**L211**	**False**	**ABY1**	1.5e-05	0.00136%	0.0	1	0.00104	8.14e-06	1.77e-05	2.41e-05	0.000725%
**L851**	**False**	**koll11**	1.49e-05	0.00136%	0.0	1	0.0216	0.00298	0.00169	1.02e-05	0.000306%

**L1572**	**False**	**ZB2**	1.49e-05	0.00136%	0.0	1	0.00163	0.00033	0.00048	4.87e-05	0.00146%
**L1056**	**False**	**koll11**	1.49e-05	0.00136%	0.0	1	0.00362	0.000407	0.00059	2.65e-05	0.000796%
**L96**	**False**	**ABY1**	1.48e-05	0.00135%	0.0	1	0.0123	0.00262	0.000419	1.27e-05	0.000382%
**OTU_358**	**True**	**koll11**	1.48e-05	0.00135%	0.0	1	0.00123	6.38e-05	0.000534	5.81e-05	0.00174%
**L1836**	**False**	**OD1**	1.48e-05	0.00134%	0.0	1	0.00193	0.000563	0.000673	0.000123	0.00369%
**L308**	**False**	**ABY1**	1.48e-05	0.00134%	0.0	1	3.54e-05	0.00392	0.00334	2.7e-05	0.00081%
**L262**	**False**	**OD1**	1.47e-05	0.00134%	0.0	1	0.00776	0.00371	0.0017	5.54e-05	0.00166%
**OTU_8776**	**True**	**Mb-NB09**	1.47e-05	0.00134%	0.0	1	0.00242	0.00072	2.35e-05	3.28e-05	0.000986%
**L535**	**False**	**SBRH58**	1.47e-05	0.00134%	0.0	1	0.00138	0.000314	9.14e-05	5.82e-05	0.00175%
**OTU_7995**	**True**	**ABY1**	1.47e-05	0.00133%	0.0	1	6.32e-07	0.000326	0.00117	5.5e-05	0.00165%
**L183**	**False**	**SM2F11**	1.46e-05	0.00133%	0.0	1	0.00793	0.00167	0.000235	1.29e-05	0.000386%
**OTU_32**	**True**	**Mb-NB09**	1.46e-05	0.00133%	0.0	1	0.000323	3.89e-05	7e-06	3.17e-05	0.000951%
**L1692**	**False**	**OD1**	1.44e-05	0.00131%	0.0	1	0.00321	0.000401	8.26e-05	1.14e-05	0.000342%
**OTU_6635**	**True**	**OD1**	1.44e-05	0.00131%	0.0	1	0.00075	4.43e-06	2.97e-06	1.66e-05	0.000497%
**L116**	**False**	**OD1**	1.44e-05	0.00131%	0.0	1	0.0134	0.0409	0.0242	7.74e-05	0.00232%
**L483**	**False**	**OP11-4**	1.44e-05	0.00131%	0.0	1	0.0019	0.000225	1.57e-05	1.9e-05	0.000569%
**OTU_7179**	**True**	**OD1**	1.43e-05	0.0013%	0.0	1	0.000374	0.000216	0.0	7.3e-05	0.00219%
**L1778**	**False**	**OD1**	1.43e-05	0.0013%	0.0	1	0.000869	4.43e-06	2.97e-06	1.33e-05	0.000399%
**OTU_9001**	**True**	**ABY1**	1.43e-05	0.0013%	0.0	1	0.000974	3.77e-05	1.38e-05	3.24e-05	0.000972%
**OTU_6780**	**True**	**ZB2**	1.42e-05	0.0013%	0.0	1	0.0042	0.00128	0.00345	0.00012	0.00361%
**L1306**	**False**	**OD1**	1.42e-05	0.00129%	0.0	1	0.00603	0.000526	0.000664	2.05e-05	0.000615%
**OTU_10749**	**True**	**OP11-4**	1.41e-05	0.00129%	0.0	1	0.0127	0.00319	0.00164	3.98e-05	0.00119%
**L1356**	**False**	**ZB2**	1.4e-05	0.00128%	0.0	1	0.00115	0.000128	7.77e-06	2.51e-05	0.000753%
**L1463**	**False**	**ZB2**	1.39e-05	0.00126%	0.0	1	0.000276	0.00386	0.0055	3.08e-05	0.000923%
**L80**	**False**	**OD1**	1.38e-05	0.00126%	0.0	1	0.0358	0.0501	0.0264	0.000159	0.00477%
**L1457**	**False**	**ZB2**	1.38e-05	0.00125%	0.0	1	0.0	0.000234	0.00185	3.44e-05	0.00103%
**L1125**	**False**	**koll11**	1.38e-05	0.00125%	0.0	1	0.00299	0.000183	9.89e-05	2.4e-05	0.000721%
**L514**	**False**	**OP11-3**	1.37e-05	0.00125%	0.0	1	0.00211	0.000407	5.68e-05	4.03e-05	0.00121%
**L346**	**False**	**ABY1**	1.37e-05	0.00124%	0.0	1	0.00598	0.000934	0.000684	2.25e-05	0.000675%
**L489**	**False**	**OP11-4**	1.36e-05	0.00124%	0.0	1	0.0059	0.00116	0.000652	2.22e-05	0.000667%
**L226**	**False**	**ABY1**	1.36e-05	0.00124%	0.0	1	0.00105	7.76e-05	0.0	2.82e-05	0.000846%
**L251**	**False**	**ABY1**	1.35e-05	0.00123%	0.0	1	0.00477	0.00121	0.000155	2.29e-05	0.000687%
**OTU_7952**	**True**	**ABY1**	1.35e-05	0.00123%	0.0	1	0.000662	0.0	7.1e-07	2.16e-05	0.000648%
**L1684**	**False**	**Mb-NB09**	1.35e-05	0.00123%	0.0	1	0.0025	0.00072	2.94e-05	2.86e-05	0.000859%
**OTU_994**	**True**	**[Acetothermia]**	1.34e-05	0.00122%	0.0	1	0.000346	0.0	0.0	3.44e-05	0.00103%
**L129**	**False**	**ABY1**	1.34e-05	0.00122%	0.0	1	0.00137	0.000162	1.89e-05	2.8e-05	0.00084%
**L737**	**False**	**PBS-25**	1.33e-05	0.00121%	0.0	1	0.0147	0.00141	0.00181	2.35e-05	0.000704%
**L1197**	**False**	**koll11**	1.33e-05	0.00121%	0.0	1	0.00197	0.0009	0.000184	4.21e-05	0.00126%
**OTU_8074**	**True**	**ABY1**	1.32e-05	0.0012%	0.0	1	0.000111	0.00236	0.00212	4.32e-05	0.0013%
**OTU_567**	**True**	**koll11**	1.32e-05	0.0012%	0.0	1	0.00102	2.62e-05	0.0	2.72e-05	0.000816%
**L444**	**False**	**OP11-4**	1.31e-05	0.0012%	0.0	1	0.00131	0.00017	0.00102	7.51e-05	0.00225%
**OTU_8250**	**True**	**ABY1**	1.31e-05	0.00119%	0.0	1	0.00151	0.000689	6.24e-05	5.02e-05	0.00151%
**L1857**	**False**	**OD1**	1.31e-05	0.00119%	0.0	1	0.000525	2.05e-05	1.85e-05	1.97e-05	0.00059%
**OTU_419**	**True**	**koll11**	1.3e-05	0.00119%	0.0	1	0.00112	1.5e-05	0.0	2.43e-05	0.000728%
**OTU_6177**	**True**	**OD1**	1.3e-05	0.00118%	0.0	1	0.000576	0.000145	0.0	2.67e-05	0.000801%
**OTU_7223**	**True**	**ABY1**	1.29e-05	0.00118%	0.0	1	0.000646	0.000495	7.6e-06	3.68e-05	0.0011%
**L79**	**False**	**OD1**	1.29e-05	0.00118%	0.0	1	0.0358	0.0521	0.0284	0.000156	0.00467%
**OTU_7045**	**True**	**ABY1**	1.29e-05	0.00117%	0.0	1	6.13e-05	0.0151	0.00517	3.26e-05	0.00098%
**L264**	**False**	**OD1**	1.29e-05	0.00117%	0.0	1	0.0037	0.00167	0.00137	0.000116	0.00349%
**L101**	**False**	**ABY1**	1.29e-05	0.00117%	0.0	1	0.00914	0.0014	0.000244	1.37e-05	0.000412%
**OTU_6084**	**True**	**ZB2**	1.27e-05	0.00116%	0.0	1	3.26e-05	0.0034	0.00132	3.45e-05	0.00104%
**OTU_6663**	**True**	**ZB2**	1.27e-05	0.00116%	0.0	1	0.00171	0.00144	7.1e-05	6.22e-05	0.00187%
**OTU_2490**	**True**	**koll11**	1.27e-05	0.00116%	0.0	1	0.00519	0.000408	0.00285	5.79e-05	0.00174%
**L802**	**False**	**OP3**	1.26e-05	0.00115%	0.0	1	0.0517	0.00638	0.00815	9.68e-06	0.00029%
**L370**	**False**	**OD1**	1.26e-05	0.00115%	0.0	1	0.00185	0.000261	0.000307	2.92e-05	0.000877%
**L995**	**False**	**koll11**	1.26e-05	0.00115%	0.0	1	0.00827	0.00157	0.00197	3.01e-05	0.000904%
**OTU_6502**	**True**	**WCHB1-64**	1.25e-05	0.00114%	0.0	1	3.29e-05	0.000317	0.000965	4.58e-05	0.00138%
**OTU_2352**	**True**	**SJA-4**	1.25e-05	0.00114%	0.0	1	0.0007	0.0	0.0	2.57e-05	0.000772%
**OTU_10764**	**True**	**OP11-4**	1.24e-05	0.00113%	0.0	1	0.0045	0.000969	0.000471	2.59e-05	0.000776%
**OTU_9054**	**True**	**ABY1**	1.24e-05	0.00113%	0.0	1	0.000195	0.0	0.000704	5.98e-05	0.0018%
**OTU_6086**	**True**	**ZB2**	1.24e-05	0.00112%	0.0	1	0.000591	8.86e-06	1.57e-05	2.71e-05	0.000814%
**OTU_6232**	**True**	**ZB2**	1.23e-05	0.00112%	0.0	1	6.96e-05	0.000973	0.00415	4.31e-05	0.00129%
**L1862**	**False**	**OD1**	1.23e-05	0.00112%	0.0	1	0.00258	0.0015	0.000205	3.16e-05	0.000948%
**OTU_5846**	**True**	**OD1**	1.23e-05	0.00112%	0.0	1	0.0	0.000868	0.0	3.72e-05	0.00112%
**L531**	**False**	**TM6**	1.22e-05	0.00111%	0.0	1	0.000164	0.00191	0.000134	6.72e-05	0.00202%

**OTU_8007**	**True**	**ABY1**	1.21e-05	0.0011%	0.0	1	0.000849	1.01e-05	0.0	2.65e-05	0.000794%
**L1019**	**False**	**koll11**	1.21e-05	0.0011%	0.0	1	0.00803	0.00111	0.000381	2.21e-05	0.000662%
**L136**	**False**	**OD1**	1.2e-05	0.00109%	0.0	1	0.00807	0.0303	0.0164	7.05e-05	0.00211%
**OTU_5425**	**True**	**OD1**	1.19e-05	0.00108%	0.0	1	0.00196	0.000254	2.48e-05	1.61e-05	0.000484%
**OTU_7967**	**True**	**ABY1**	1.19e-05	0.00108%	0.0	1	0.00247	0.00148	2.85e-05	3.99e-05	0.0012%
**OTU_7951**	**True**	**ABY1**	1.19e-05	0.00108%	0.0	1	0.00113	0.000779	0.0	5.33e-05	0.0016%
**OTU_1554**	**True**	**PBS-25**	1.18e-05	0.00108%	0.0	1	0.000837	0.0	0.0	1.55e-05	0.000465%
**L1696**	**False**	**Mb-NB09**	1.18e-05	0.00107%	0.0	1	0.000955	0.000151	4.65e-05	1.87e-05	0.000562%
**L991**	**False**	**koll11**	1.18e-05	0.00107%	0.0	1	0.0119	0.00216	0.0028	2.21e-05	0.000663%
**L1147**	**False**	**koll11**	1.18e-05	0.00107%	0.0	1	0.00754	0.000664	0.0009	2.81e-05	0.000843%
**L417**	**False**	**Bacteria**	1.17e-05	0.00107%	0.0	1	0.837	0.845	0.913	8.98e-05	0.00269%
**OTU_7990**	**True**	**ABY1**	1.17e-05	0.00107%	0.0	1	0.000735	9.02e-05	0.0	2.4e-05	0.000719%
**OTU_4950**	**True**	**[Acetothermia]**	1.17e-05	0.00107%	0.0	1	0.00035	2.53e-05	0.0	2.51e-05	0.000753%
**L1856**	**False**	**OD1**	1.17e-05	0.00106%	0.0	1	0.00311	0.00152	0.000224	2.37e-05	0.000711%
**L1824**	**False**	**OD1**	1.16e-05	0.00106%	0.0	1	0.00121	0.000196	0.000159	4.02e-05	0.00121%
**L1084**	**False**	**koll11**	1.15e-05	0.00105%	0.0	1	0.00813	0.000691	0.000283	2.08e-05	0.000625%
**L1629**	**False**	**ZB2**	1.15e-05	0.00104%	0.0	1	0.00444	0.00259	0.00139	0.000111	0.00332%
**L1892**	**False**	**OD1**	1.15e-05	0.00104%	0.0	1	0.00207	0.000738	0.00015	2.82e-05	0.000845%
**L1611**	**False**	**ZB2**	1.14e-05	0.00104%	0.0	1	0.00169	9.1e-05	8.5e-05	1.54e-05	0.000463%
**OTU_7373**	**True**	**OD1**	1.13e-05	0.00103%	0.0	1	0.000859	0.000297	0.000228	6.2e-05	0.00186%
**L380**	**False**	**OD1**	1.13e-05	0.00103%	0.0	1	0.000845	3.53e-05	7.74e-05	2.31e-05	0.000694%
**OTU_323**	**True**	**koll11**	1.11e-05	0.00101%	0.0	1	0.00147	0.000133	3.25e-05	2.02e-05	0.000606%
**L877**	**False**	**koll11**	1.11e-05	0.00101%	0.0	1	0.0198	0.00479	0.00566	2.05e-05	0.000616%
**L1502**	**False**	**ZB2**	1.09e-05	0.000994%	0.0	1	0.000183	0.00103	3.27e-05	4.76e-05	0.00143%
**OTU_292**	**True**	**PBS-25**	1.09e-05	0.000992%	0.0	1	0.00657	0.000653	0.000654	2.07e-05	0.00062%
**OTU_6667**	**True**	**ABY1**	1.09e-05	0.00099%	0.0	1	0.000245	0.0312	0.00884	2.24e-05	0.000671%
**L1894**	**False**	**OD1**	1.09e-05	0.000989%	0.0	1	0.00192	0.000688	0.000136	2.74e-05	0.000821%
**L1245**	**False**	**TM7-1**	1.08e-05	0.000979%	0.0	1	0.000896	0.0105	0.00755	3.69e-05	0.00111%
**L1012**	**False**	**koll11**	1.08e-05	0.000979%	0.0	1	0.0347	0.00644	0.00654	1.1e-05	0.00033%
**OTU_6170**	**True**	**ZB2**	1.07e-05	0.000977%	0.0	1	0.000641	5.1e-05	0.0	1.84e-05	0.000552%
**L714**	**False**	**PBS-25**	1.07e-05	0.00097%	0.0	1	0.0127	0.00187	0.00787	4.37e-05	0.00131%
**OTU_1087**	**True**	**OP3**	1.06e-05	0.000968%	0.0	1	0.000462	0.0	3.89e-06	2.05e-05	0.000615%
**L126**	**False**	**OD1**	1.06e-05	0.000967%	0.0	1	0.0124	0.0311	0.0167	0.000103	0.0031%
**OTU_9123**	**True**	**ABY1**	1.06e-05	0.000965%	0.0	1	0.00174	0.000229	1.96e-05	4.37e-05	0.00131%
**OTU_8179**	**True**	**EW055**	1.05e-05	0.000954%	0.0	1	0.000341	9.06e-07	0.0124	2.95e-05	0.000885%
**L589**	**False**	**PRR-12**	1.04e-05	0.000948%	0.0	1	0.01	0.00137	0.000637	1.32e-05	0.000397%
**L607**	**False**	**PRR-12**	1.04e-05	0.000944%	0.0	1	0.00298	0.000341	2.96e-06	1.74e-05	0.000523%
**L119**	**False**	**ABY1**	1.04e-05	0.000943%	0.0	1	0.00101	0.00948	0.00624	4.18e-05	0.00125%
**L1238**	**False**	**TM7**	1.03e-05	0.000934%	0.0	1	0.0131	0.0122	0.021	0.000412	0.0124%
**L1591**	**False**	**OD1**	1.02e-05	0.000932%	0.0	1	0.00167	0.00918	0.00397	4.86e-05	0.00146%
**L961**	**False**	**koll11**	1.02e-05	0.00093%	0.0	1	0.0244	0.00282	0.00183	1.01e-05	0.000304%
**OTU_205**	**True**	**koll11**	1.02e-05	0.000925%	0.0	1	0.00578	0.000965	0.000283	1.39e-05	0.000417%
**OTU_327**	**True**	**koll11**	1.01e-05	0.000923%	0.0	1	0.00116	5.37e-05	0.0	2e-05	0.000601%
**OTU_8135**	**True**	**EW055**	1.01e-05	0.000917%	0.0	1	0.00202	0.000186	0.0	3.1e-05	0.000929%
**OTU_259**	**True**	**PBS-25**	1e-05	0.000912%	0.0	1	0.00543	0.000569	0.000748	2.28e-05	0.000684%
**OTU_7164**	**True**	**ABY1**	1e-05	0.000912%	0.0	1	0.000251	0.0	0.0	2.56e-05	0.000769%
**OTU_8072**	**True**	**ABY1**	9.99e-06	0.00091%	0.0	1	0.000657	0.000534	0.000102	7.75e-05	0.00233%
**OTU_8948**	**True**	**TM7**	9.98e-06	0.000909%	0.0	1	0.000549	6.72e-05	2.84e-05	3.02e-05	0.000906%
**L1905**	**False**	**OD1**	9.95e-06	0.000906%	0.0	1	0.0012	9.83e-05	8.77e-06	1.27e-05	0.000382%
**L326**	**False**	**ABY1**	9.93e-06	0.000904%	0.0	1	0.000947	0.000362	8.68e-05	4.12e-05	0.00124%
**L1472**	**False**	**ZB2**	9.91e-06	0.000902%	0.0	1	0.00423	0.00172	0.000902	3.3e-05	0.00099%
**L274**	**False**	**ABY1**	9.89e-06	0.0009%	0.0	1	0.000183	0.0017	0.00103	4.86e-05	0.00146%
**L752**	**False**	**PBS-25**	9.85e-06	0.000897%	0.0	1	0.00663	0.000457	0.0025	2.68e-05	0.000803%
**OTU_201**	**True**	**OD1**	9.78e-06	0.00089%	0.0	1	0.00023	0.0	6.91e-06	2.25e-05	0.000674%
**OTU_6073**	**True**	**ZB2**	9.74e-06	0.000886%	0.0	1	5.9e-05	0.00487	0.00399	5.55e-05	0.00167%
**OTU_5635**	**True**	**OD1**	9.74e-06	0.000886%	0.0	1	0.000331	0.0	3.85e-06	2.08e-05	0.000624%
**OTU_409**	**True**	**koll11**	9.73e-06	0.000886%	0.0	1	0.00115	2.48e-05	0.0	2.3e-05	0.00069%
**L1352**	**False**	**OD1**	9.72e-06	0.000885%	0.0	1	0.00277	0.00146	0.000925	0.000105	0.00316%
**L437**	**False**	**OP11**	9.69e-06	0.000882%	0.0	1	0.0008	1.58e-05	0.0	1.08e-05	0.000324%
**L654**	**False**	**koll11**	9.64e-06	0.000877%	0.0	1	0.0207	0.00312	0.00473	9.87e-06	0.000296%
**L1838**	**False**	**OD1**	9.61e-06	0.000875%	0.0	1	0.00184	0.000545	0.000276	5.11e-05	0.00153%
**L1044**	**False**	**koll11**	9.61e-06	0.000874%	0.0	1	0.0131	0.00219	0.00636	3.03e-05	0.000908%
**L1608**	**False**	**ZB2**	9.6e-06	0.000874%	0.0	1	0.00232	0.000218	0.000194	1.36e-05	0.000408%
**OTU_437**	**True**	**PBS-25**	9.57e-06	0.000872%	0.0	1	0.000988	0.0	1.4e-05	1.98e-05	0.000593%

**OTU_10801**	**True**	**OP11-4**	9.52e-06	0.000866%	0.0	1	0.0014	0.00019	0.000181	1.67e-05	0.000502%
**L328**	**False**	**ABY1**	9.51e-06	0.000866%	0.0	1	0.000947	0.000133	8.13e-05	2.25e-05	0.000677%
**OTU_2622**	**True**	**SJA-4**	9.45e-06	0.00086%	0.0	1	0.0	0.0	0.000762	2.44e-05	0.000732%
**L979**	**False**	**koll11**	9.45e-06	0.00086%	0.0	1	0.00336	0.000681	0.000447	2.02e-05	0.000605%
**OTU_7941**	**True**	**ABY1**	9.42e-06	0.000857%	0.0	1	0.000849	1.11e-05	2.91e-05	1.41e-05	0.000423%
**L1037**	**False**	**koll11**	9.41e-06	0.000857%	0.0	1	0.00833	0.00134	0.00481	3e-05	0.0009%
**L862**	**False**	**koll11**	9.41e-06	0.000856%	0.0	1	0.0259	0.00645	0.00598	1.22e-05	0.000366%
**OTU_8016**	**True**	**TM7-3**	9.34e-06	0.00085%	0.0	1	0.0	2.57e-05	0.000628	2.81e-05	0.000844%
**OTU_7840**	**True**	**ABY1**	9.32e-06	0.000849%	0.0	1	0.00215	0.000177	0.000122	1.65e-05	0.000494%
**OTU_8182**	**True**	**EW055**	9.3e-06	0.000846%	0.0	1	0.0	0.0	0.00262	2.37e-05	0.000712%
**L1262**	**False**	**TM7-1**	9.28e-06	0.000845%	0.0	1	0.00204	0.000594	5.57e-05	1.96e-05	0.000589%
**L854**	**False**	**OP3**	9.25e-06	0.000842%	0.0	1	0.0223	0.00257	0.0019	4.93e-06	0.000148%
**OTU_12**	**True**	**ZB2**	9.24e-06	0.000841%	0.0	1	0.00132	0.000136	0.000109	1.16e-05	0.000347%
**OTU_276**	**True**	**koll11**	9.2e-06	0.000837%	0.0	1	0.00221	0.000101	7.04e-05	1.65e-05	0.000496%
**L627**	**False**	**PBS-25**	8.96e-06	0.000815%	0.0	1	0.0062	0.000215	7.94e-05	9.16e-06	0.000275%
**L1816**	**False**	**OD1**	8.94e-06	0.000813%	0.0	1	0.000408	0.000498	8.26e-06	5.54e-05	0.00166%
**L188**	**False**	**SM2F11**	8.93e-06	0.000813%	0.0	1	0.00494	0.0011	0.000185	1.31e-05	0.000392%
**L677**	**False**	**koll11**	8.93e-06	0.000813%	0.0	1	0.00673	0.000363	0.000709	2.12e-05	0.000636%
**L1610**	**False**	**ZB2**	8.93e-06	0.000813%	0.0	1	0.00218	0.000109	0.000131	1.17e-05	0.000352%
**L634**	**False**	**PBS-25**	8.81e-06	0.000801%	0.0	1	0.00267	0.000128	8.32e-06	1.44e-05	0.000432%
**OTU_7166**	**True**	**ABY1**	8.79e-06	0.0008%	0.0	1	0.000677	0.0	0.0	1.88e-05	0.000563%
**OTU_8910**	**True**	**TM7-1**	8.78e-06	0.000799%	0.0	1	0.000421	0.0	0.0	1.61e-05	0.000485%
**L1799**	**False**	**OD1**	8.76e-06	0.000797%	0.0	1	0.00121	1.94e-05	3e-05	8.32e-06	0.00025%
**OTU_217**	**True**	**koll11**	8.74e-06	0.000796%	0.0	1	0.0066	0.0013	0.00455	4.19e-05	0.00126%
**L1249**	**False**	**TM7-1**	8.69e-06	0.000791%	0.0	1	0.000872	6.32e-05	3.14e-06	9.08e-06	0.000272%
**L1711**	**False**	**OD1**	8.68e-06	0.00079%	0.0	1	0.00345	0.000807	0.00014	1.56e-05	0.00047%
**L1532**	**False**	**OD1**	8.67e-06	0.000789%	0.0	1	0.00393	0.000632	0.0015	2.51e-05	0.000752%
**L773**	**False**	**PBS-25**	8.66e-06	0.000788%	0.0	1	0.00257	0.000217	0.00032	2.66e-05	0.000797%
**OTU_7192**	**True**	**ZB2**	8.62e-06	0.000784%	0.0	1	0.0	0.0	0.00067	2.22e-05	0.000666%
**OTU_9132**	**True**	**TM7**	8.59e-06	0.000782%	0.0	1	0.00063	0.0	0.0	1.82e-05	0.000545%
**L1096**	**False**	**koll11**	8.59e-06	0.000782%	0.0	1	0.0111	0.00192	0.00312	1.37e-05	0.000411%
**L425**	**False**	**OP11**	8.57e-06	0.00078%	0.0	1	0.00231	0.000816	0.00336	6.22e-05	0.00187%
**OTU_247**	**True**	**koll11**	8.56e-06	0.000779%	0.0	1	0.0035	0.000891	0.00383	8.77e-05	0.00263%
**L84**	**False**	**ABY1**	8.55e-06	0.000778%	0.0	1	0.00144	0.000297	0.0	1.82e-05	0.000545%
**L1039**	**False**	**koll11**	8.52e-06	0.000775%	0.0	1	0.00708	0.00132	0.00481	3.67e-05	0.0011%
**OTU_9845**	**True**	**SC3**	8.46e-06	0.00077%	0.0	1	0.000424	0.0	0.0	1.61e-05	0.000484%
**L1144**	**False**	**koll11**	8.45e-06	0.000769%	0.0	1	0.00825	0.00106	0.00105	2.05e-05	0.000615%
**OTU_6586**	**True**	**OD1**	8.38e-06	0.000762%	0.0	1	0.0016	0.000493	0.000251	5.32e-05	0.0016%
**OTU_6179**	**True**	**ZB2**	8.36e-06	0.000761%	0.0	1	0.000454	1.18e-05	7.5e-06	1.08e-05	0.000323%
**L1722**	**False**	**Mb-NB09**	8.36e-06	0.000761%	0.0	1	0.00387	0.00161	0.00076	3.1e-05	0.000929%
**L1055**	**False**	**koll11**	8.36e-06	0.000761%	0.0	1	0.00403	0.000442	0.000611	1.37e-05	0.00041%
**OTU_8127**	**True**	**ABY1**	8.32e-06	0.000757%	0.0	1	0.0	3.32e-05	0.000588	1.5e-05	0.000449%
**OTU_2335**	**True**	**SBRH58**	8.28e-06	0.000753%	0.0	1	0.00108	0.000314	9.13e-05	4.73e-05	0.00142%
**L1559**	**False**	**ZB2**	8.27e-06	0.000753%	0.0	1	0.00821	0.000925	0.000379	1.22e-05	0.000367%
**L8**	**False**	**ABY1**	8.26e-06	0.000752%	0.0	1	0.00138	0.000585	7.6e-06	1.9e-05	0.000571%
**L113**	**False**	**ABY1**	8.26e-06	0.000752%	0.0	1	1.99e-05	0.00233	0.00126	1.93e-05	0.00058%
**OTU_8196**	**True**	**TM7-3**	8.21e-06	0.000748%	0.0	1	0.00636	0.0	1.4e-05	1.86e-05	0.000557%
**L1516**	**False**	**OD1**	8.21e-06	0.000747%	0.0	1	0.00222	0.000137	0.00102	3.75e-05	0.00113%
**OTU_7442**	**True**	**TM7-1**	8.17e-06	0.000743%	0.0	1	0.00092	0.000594	5.57e-05	3.32e-05	0.000998%
**L1016**	**False**	**koll11**	8.11e-06	0.000738%	0.0	1	0.0127	0.00218	0.00409	2.73e-05	0.000818%
**OTU_9129**	**True**	**Mb-NB09**	8.1e-06	0.000737%	0.0	1	0.00263	0.000103	5.5e-07	2.03e-05	0.000608%
**OTU_5341**	**True**	**OD1**	8.09e-06	0.000736%	0.0	1	0.0	0.000266	0.0	2.34e-05	0.000702%
**L128**	**False**	**OD1**	8.07e-06	0.000735%	0.0	1	0.0124	0.0309	0.0167	8e-05	0.0024%
**L1845**	**False**	**OD1**	8.05e-06	0.000733%	0.0	1	0.00469	0.00115	0.00044	1.76e-05	0.000529%
**L538**	**False**	**SJA-4**	8.03e-06	0.000731%	0.0	1	0.00489	0.00132	0.00255	7.95e-05	0.00238%
**OTU_6064**	**True**	**ZB2**	8.03e-06	0.000731%	0.0	1	0.000101	0.00173	0.00189	3.84e-05	0.00115%
**L745**	**False**	**PBS-25**	7.97e-06	0.000725%	0.0	1	0.00846	0.000636	0.00254	1.71e-05	0.000512%
**L1179**	**False**	**GIF10**	7.97e-06	0.000725%	0.0	1	0.00458	0.000574	0.00033	9.94e-06	0.000298%
**OTU_5362**	**True**	**ZB2**	7.92e-06	0.000721%	0.0	1	0.000655	1.67e-05	2.8e-06	1.35e-05	0.000405%
**OTU_9171**	**True**	**Mb-NB09**	7.91e-06	0.00072%	0.0	1	0.000782	5.31e-05	0.0	1.18e-05	0.000353%
**OTU_6914**	**True**	**ZB2**	7.91e-06	0.00072%	0.0	1	0.000842	0.000336	8.21e-05	3.58e-05	0.00107%
**L395**	**False**	**ABY1**	7.9e-06	0.000719%	0.0	1	0.00309	0.000133	0.000122	7.6e-06	0.000228%
**L1045**	**False**	**koll11**	7.88e-06	0.000717%	0.0	1	0.0118	0.00215	0.0062	2.98e-05	0.000893%
**L1702**	**False**	**OD1**	7.87e-06	0.000717%	0.0	1	0.00213	0.000165	3.61e-05	9e-06	0.00027%

**L492**	**False**	**OP11-4**	7.86e-06	0.000715%	0.0	1	0.00142	0.000368	7.14e-05	1.12e-05	0.000336%
**L1770**	**False**	**OD1**	7.85e-06	0.000715%	0.0	1	0.00196	0.000411	0.000104	1.94e-05	0.000581%
**L776**	**False**	**PBS-25**	7.85e-06	0.000715%	0.0	1	0.0018	0.00017	8.61e-05	2.22e-05	0.000666%
**L283**	**False**	**ABY1**	7.85e-06	0.000714%	0.0	1	0.00704	0.0195	0.0204	6.32e-05	0.0019%
**L14**	**False**	**OD1**	7.83e-06	0.000713%	0.0	1	0.00152	0.000813	5.45e-06	2.72e-05	0.000816%
**L82**	**False**	**ABY1**	7.82e-06	0.000712%	0.0	1	0.00174	0.000778	1.24e-05	2.22e-05	0.000665%
**L1190**	**False**	**koll11**	7.81e-06	0.000711%	0.0	1	0.00119	1.16e-05	3.38e-05	1.1e-05	0.00033%
**OTU_6987**	**True**	**OD1**	7.8e-06	0.00071%	0.0	1	0.00465	0.00161	0.000296	1.97e-05	0.00059%
**L1547**	**False**	**ZB2**	7.79e-06	0.000709%	0.0	1	0.00248	0.000283	0.000108	1e-05	0.000301%
**OTU_6039**	**True**	**OD1**	7.77e-06	0.000707%	0.0	1	0.000256	1.12e-05	3.27e-05	2.73e-05	0.00082%
**OTU_7940**	**True**	**ABY1**	7.75e-06	0.000705%	0.0	1	0.00239	0.000116	0.000107	8.64e-06	0.000259%
**OTU_8113**	**True**	**ABY1**	7.74e-06	0.000705%	0.0	1	0.000299	0.000482	1.24e-05	4.64e-05	0.00139%
**L978**	**False**	**koll11**	7.73e-06	0.000704%	0.0	1	0.00367	0.000869	0.000447	1.76e-05	0.000528%
**L1048**	**False**	**koll11**	7.73e-06	0.000703%	0.0	1	0.00725	0.00147	0.00386	4.4e-05	0.00132%
**OTU_2377**	**True**	**SJA-4**	7.7e-06	0.000701%	0.0	1	0.000551	0.0	0.0	1.58e-05	0.000475%
**L1886**	**False**	**OD1**	7.69e-06	0.0007%	0.0	1	0.00252	0.000827	0.000171	1.49e-05	0.000447%
**OTU_8003**	**True**	**d153**	7.67e-06	0.000698%	0.0	1	0.00027	5.04e-07	0.0	1.57e-05	0.000471%
**OTU_8764**	**True**	**ABY1**	7.66e-06	0.000698%	0.0	1	0.000595	7.5e-05	5.83e-08	1.46e-05	0.000439%
**L1694**	**False**	**OD1**	7.66e-06	0.000697%	0.0	1	0.00308	0.000401	8.26e-05	6.78e-06	0.000203%
**L65**	**False**	**ABY1**	7.63e-06	0.000695%	0.0	1	0.00215	0.000229	0.000101	2.8e-05	0.000839%
**L139**	**False**	**OD1**	7.61e-06	0.000693%	0.0	1	0.0079	0.0178	0.00593	7.1e-05	0.00213%
**OTU_10750**	**True**	**OP11-4**	7.58e-06	0.00069%	0.0	1	0.000364	0.0	0.0	1.61e-05	0.000484%
**OTU_5954**	**True**	**OD1**	7.58e-06	0.00069%	0.0	1	0.000561	0.0	1.48e-05	1.77e-05	0.00053%
**OTU_8147**	**True**	**ABY1**	7.57e-06	0.000689%	0.0	1	0.000453	3.78e-06	2.49e-05	1.02e-05	0.000307%
**OTU_8013**	**True**	**ABY1**	7.55e-06	0.000688%	0.0	1	4.9e-06	0.000477	0.00154	2.8e-05	0.000839%
**L1131**	**False**	**koll11**	7.54e-06	0.000687%	0.0	1	0.0018	0.000213	0.000127	1.66e-05	0.000497%
**OTU_4911**	**True**	**OD1**	7.51e-06	0.000684%	0.0	1	0.000751	0.000225	1.83e-06	2.43e-05	0.000729%
**L1551**	**False**	**ZB2**	7.47e-06	0.00068%	0.0	1	0.00541	0.00135	0.000553	1.51e-05	0.000453%
**OTU_7946**	**True**	**ABY1**	7.45e-06	0.000678%	0.0	1	0.000209	0.0	0.0	1.38e-05	0.000413%
**OTU_7982**	**True**	**ABY1**	7.41e-06	0.000674%	0.0	1	0.00274	0.00147	7.84e-05	2.16e-05	0.00065%
**L55**	**False**	**ABY1**	7.38e-06	0.000671%	0.0	1	0.00293	8.35e-05	0.0	1.54e-05	0.000462%
**OTU_8823**	**True**	**OD1**	7.32e-06	0.000667%	0.0	1	0.000755	0.0	0.0	1.51e-05	0.000454%
**OTU_4488**	**True**	**PRR-12**	7.3e-06	0.000664%	0.0	1	0.00021	0.0	0.0	1.54e-05	0.000462%
**OTU_2351**	**True**	**SJA-4**	7.26e-06	0.000661%	0.0	1	0.000375	0.0	0.0	1.49e-05	0.000446%
**L345**	**False**	**OD1**	7.25e-06	0.00066%	0.0	1	0.00648	0.00103	0.000697	1.1e-05	0.000329%
**OTU_9178**	**True**	**ABY1**	7.25e-06	0.00066%	0.0	1	0.000446	0.0	5.83e-08	8.88e-06	0.000266%
**OTU_8334**	**True**	**ABY1**	7.19e-06	0.000655%	0.0	1	0.000264	0.0	0.0	1.54e-05	0.000461%
**L356**	**False**	**ABY1**	7.16e-06	0.000652%	0.0	1	0.000481	2.32e-05	3.68e-06	9.43e-06	0.000283%
**OTU_2076**	**True**	**PRR-12**	7.12e-06	0.000648%	0.0	1	0.00064	0.0	5.83e-08	1.07e-05	0.000321%
**OTU_163**	**True**	**koll11**	7.06e-06	0.000642%	0.0	1	0.00704	0.00207	0.00296	4.72e-05	0.00142%
**OTU_895**	**True**	**PRR-12**	7.04e-06	0.000641%	0.0	1	0.00112	8.13e-05	1.83e-05	1.08e-05	0.000325%
**L41**	**False**	**OD1**	7.02e-06	0.000639%	0.0	1	0.00411	0.000409	0.000857	1.14e-05	0.000342%
**L334**	**False**	**OD1**	6.98e-06	0.000636%	0.0	1	0.000795	0.000287	4.55e-05	2.19e-05	0.000658%
**OTU_430**	**True**	**PBS-25**	6.98e-06	0.000635%	0.0	1	0.00526	0.00034	0.00074	1.42e-05	0.000428%
**L107**	**False**	**ABY1**	6.96e-06	0.000634%	0.0	1	0.00396	0.000674	0.000337	6.71e-06	0.000201%
**L1504**	**False**	**ZB2**	6.96e-06	0.000634%	0.0	1	0.000183	0.00077	0.0	3.5e-05	0.00105%
**L506**	**False**	**OP11-4**	6.91e-06	0.000629%	0.0	1	0.00571	0.000683	0.000561	8.54e-06	0.000256%
**OTU_6990**	**True**	**ZB2**	6.91e-06	0.000629%	0.0	1	0.00011	0.00209	0.000806	3.37e-05	0.00101%
**L876**	**False**	**koll11**	6.9e-06	0.000628%	0.0	1	0.0199	0.00483	0.00568	1.26e-05	0.000378%
**L824**	**False**	**PBS-25**	6.88e-06	0.000626%	0.0	1	0.0188	0.00191	0.00143	6.35e-06	0.00019%
**OTU_1149**	**True**	**koll11**	6.88e-06	0.000626%	0.0	1	0.000569	0.000317	5.49e-05	3.28e-05	0.000983%
**OTU_7850**	**True**	**ABY1**	6.86e-06	0.000625%	0.0	1	0.000172	0.0	1.1e-06	1.49e-05	0.000448%
**L718**	**False**	**PBS-25**	6.84e-06	0.000623%	0.0	1	0.00459	0.000868	0.000585	1.68e-05	0.000503%
**L1486**	**False**	**ZB2**	6.83e-06	0.000621%	0.0	1	0.00259	0.000893	0.000496	2.61e-05	0.000784%
**L858**	**False**	**OP3**	6.82e-06	0.000621%	0.0	1	0.0204	0.002	0.00172	3.66e-06	0.00011%
**L1606**	**False**	**OD1**	6.81e-06	0.00062%	0.0	1	0.00922	0.00523	0.00233	3.41e-05	0.00102%
**OTU_6178**	**True**	**ZB2**	6.8e-06	0.000619%	0.0	1	0.00332	0.000402	0.000145	1.48e-05	0.000445%
**OTU_8141**	**True**	**ABY1**	6.79e-06	0.000618%	0.0	1	2.35e-05	0.00586	0.00249	1.7e-05	0.000511%
**OTU_6986**	**True**	**ZB2**	6.78e-06	0.000617%	0.0	1	0.000561	0.000326	0.000126	3.97e-05	0.00119%
**L469**	**False**	**OP11-4**	6.73e-06	0.000613%	0.0	1	0.00232	0.000461	9.96e-05	6.04e-06	0.000181%
**OTU_325**	**True**	**koll11**	6.72e-06	0.000612%	0.0	1	0.00115	9.09e-06	0.0	1.17e-05	0.000351%
**OTU_415**	**True**	**koll11**	6.69e-06	0.000609%	0.0	1	0.000578	0.0	0.0	1.8e-05	0.000539%
**L1058**	**False**	**koll11**	6.69e-06	0.000609%	0.0	1	0.0026	0.000381	0.00059	1.84e-05	0.000553%
**OTU_8010**	**True**	**ABY1**	6.67e-06	0.000607%	0.0	1	0.000346	4.93e-05	1.59e-06	2.59e-05	0.000778%

**OTU_8008**	**True**	**TM7-1**	6.65e-06	0.000605%	0.0	1	0.000458	0.0	0.0	1.71e-05	0.000513%
**OTU_7143**	**True**	**OP11-4**	6.64e-06	0.000605%	0.0	1	0.000875	3.92e-05	0.000589	4.01e-05	0.0012%
**L470**	**False**	**OP11-4**	6.63e-06	0.000603%	0.0	1	0.00147	0.000282	6.03e-05	7.36e-06	0.000221%
**OTU_82**	**True**	**ZB2**	6.62e-06	0.000603%	0.0	1	0.000182	1.94e-05	0.000163	6.58e-05	0.00197%
**OTU_8768**	**True**	**Mb-NB09**	6.59e-06	0.0006%	0.0	1	0.00146	0.000159	5.41e-05	6.28e-06	0.000188%
**OTU_8318**	**True**	**OD1**	6.59e-06	0.0006%	0.0	1	0.00036	7e-05	0.0	2.2e-05	0.00066%
**L301**	**False**	**ABY1**	6.58e-06	0.000599%	0.0	1	0.000657	0.00594	0.00252	3.04e-05	0.000913%
**L599**	**False**	**PRR-12**	6.58e-06	0.000599%	0.0	1	0.00254	0.000161	7.3e-05	8.69e-06	0.000261%
**L1086**	**False**	**koll11**	6.58e-06	0.000599%	0.0	1	0.00813	0.000583	0.000283	1.15e-05	0.000346%
**OTU_8221**	**True**	**OD1**	6.58e-06	0.000599%	0.0	1	0.000119	0.0	0.0	1.17e-05	0.000352%
**OTU_6298**	**True**	**OD1**	6.57e-06	0.000598%	0.0	1	0.00212	0.000972	0.000524	5.33e-05	0.0016%
**OTU_10776**	**True**	**OP11-4**	6.5e-06	0.000592%	0.0	1	5.06e-05	2.91e-06	0.000445	2.39e-05	0.000717%
**L768**	**False**	**PBS-25**	6.5e-06	0.000591%	0.0	1	0.00357	0.00036	0.000348	1.38e-05	0.000414%
**L601**	**False**	**PRR-12**	6.46e-06	0.000588%	0.0	1	0.00166	0.000116	5.56e-05	1.19e-05	0.000356%
**OTU_455**	**True**	**PBS-25**	6.4e-06	0.000583%	0.0	1	0.00166	0.000152	1.77e-06	1.55e-05	0.000464%
**OTU_359**	**True**	**koll11**	6.39e-06	0.000582%	0.0	1	0.00056	0.0	0.0	1.19e-05	0.000357%
**OTU_604**	**True**	**koll11**	6.35e-06	0.000578%	0.0	1	0.00281	0.000234	5.35e-05	1.11e-05	0.000334%
**L209**	**False**	**ABY1**	6.35e-06	0.000578%	0.0	1	0.00116	3.45e-05	1.77e-05	9.17e-06	0.000275%
**L1199**	**False**	**koll11**	6.35e-06	0.000578%	0.0	1	0.00187	0.000834	0.000182	2.25e-05	0.000674%
**L1800**	**False**	**OD1**	6.34e-06	0.000577%	0.0	1	0.000592	1.94e-05	3e-05	9.39e-06	0.000282%
**L172**	**False**	**OD1**	6.33e-06	0.000577%	0.0	1	0.0278	0.00763	0.00347	7.2e-06	0.000216%
**L284**	**False**	**ABY1**	6.33e-06	0.000576%	0.0	1	0.00614	0.00952	0.0135	0.00011	0.00331%
**L827**	**False**	**PBS-25**	6.32e-06	0.000576%	0.0	1	0.00321	0.000546	0.000295	1.79e-05	0.000536%
**L120**	**False**	**ABY1**	6.31e-06	0.000574%	0.0	1	0.000948	1.76e-05	0.0	1.23e-05	0.000369%
**L499**	**False**	**OP11-4**	6.29e-06	0.000573%	0.0	1	0.000582	4.23e-05	0.0	7.24e-06	0.000217%
**L243**	**False**	**ABY1**	6.26e-06	0.00057%	0.0	1	0.000861	9.9e-05	0.00083	5.6e-05	0.00168%
**L427**	**False**	**OP11**	6.25e-06	0.000569%	0.0	1	0.00109	0.000357	0.000768	8.31e-05	0.00249%
**OTU_6669**	**True**	**ZB2**	6.23e-06	0.000567%	0.0	1	9.12e-06	0.0	0.000287	1.85e-05	0.000554%
**OTU_1019**	**True**	**PBS-25**	6.23e-06	0.000567%	0.0	1	0.000824	1.47e-05	1.05e-05	1.37e-05	0.000411%
**L147**	**False**	**OD1**	6.23e-06	0.000567%	0.0	1	0.0037	0.000906	0.000338	1.02e-05	0.000306%
**L1607**	**False**	**OD1**	6.23e-06	0.000567%	0.0	1	0.00429	0.00264	0.000931	3.3e-05	0.000991%
**L1107**	**False**	**koll11**	6.21e-06	0.000565%	0.0	1	0.00134	3.18e-05	3.72e-05	6.99e-06	0.00021%
**L336**	**False**	**OD1**	6.2e-06	0.000564%	0.0	1	0.00069	5.51e-05	4.52e-05	1.09e-05	0.000329%
**L1753**	**False**	**OD1**	6.18e-06	0.000562%	0.0	1	0.000465	0.0011	0.00151	0.000155	0.00465%
**L1291**	**False**	**TM7-3**	6.15e-06	0.00056%	0.0	1	0.00775	0.000105	0.00266	2.52e-05	0.000756%
**L1163**	**False**	**koll11**	6.12e-06	0.000557%	0.0	1	0.00144	3.75e-05	2.71e-05	1.24e-05	0.000374%
**L277**	**False**	**ABY1**	6.12e-06	0.000557%	0.0	1	0.0101	0.021	0.0205	9.49e-05	0.00285%
**OTU_7978**	**True**	**ABY1**	6.12e-06	0.000557%	0.0	1	0.000918	0.00041	2.41e-05	1.57e-05	0.000472%
**OTU_463**	**True**	**koll11**	6.11e-06	0.000556%	0.0	1	0.000377	0.000243	0.00173	4.5e-05	0.00135%
**L1908**	**False**	**OD1**	6.1e-06	0.000555%	0.0	1	0.00066	4.2e-05	5.97e-06	9.05e-06	0.000272%
**OTU_7174**	**True**	**OD1**	6.09e-06	0.000554%	0.0	1	0.00404	0.000574	0.000291	1.18e-05	0.000355%
**L363**	**False**	**OD1**	6.08e-06	0.000554%	0.0	1	0.000863	1.64e-05	7.1e-07	8.44e-06	0.000253%
**L1462**	**False**	**ZB2**	6.08e-06	0.000554%	0.0	1	0.000338	0.00439	0.00551	1.28e-05	0.000383%
**L1319**	**False**	**OD1**	6.08e-06	0.000553%	0.0	1	0.00033	0.0	1.29e-05	7.01e-06	0.00021%
**L726**	**False**	**PBS-25**	6.05e-06	0.000551%	0.0	1	0.0077	0.00109	0.00275	2.02e-05	0.000605%
**OTU_6494**	**True**	**ZB2**	6.02e-06	0.000548%	0.0	1	0.00137	0.000173	0.000211	1.41e-05	0.000423%
**L474**	**False**	**OP11-4**	6e-06	0.000546%	0.0	1	0.00129	0.00028	5.54e-05	7.28e-06	0.000218%
**L376**	**False**	**OD1**	5.98e-06	0.000544%	0.0	1	0.00117	3.53e-05	7.74e-05	7.83e-06	0.000235%
**L294**	**False**	**ABY1**	5.97e-06	0.000543%	0.0	1	0.00575	0.00619	0.0109	0.000131	0.00393%
**L631**	**False**	**PBS-25**	5.94e-06	0.00054%	0.0	1	0.00257	6.75e-05	7.1e-05	1.06e-05	0.000318%
**L1821**	**False**	**OD1**	5.92e-06	0.000539%	0.0	1	0.000816	0.000225	1.89e-06	1.71e-05	0.000514%
**OTU_7028**	**True**	**ABY1**	5.9e-06	0.000537%	0.0	1	0.0	0.00139	0.000904	1.76e-05	0.000527%
**OTU_8909**	**True**	**ABY1**	5.89e-06	0.000537%	0.0	1	0.000852	8.86e-06	0.0	1.17e-05	0.000352%
**OTU_6699**	**True**	**ZB2**	5.89e-06	0.000536%	0.0	1	0.000296	6.98e-05	0.0	2e-05	0.0006%
**L1688**	**False**	**OD1**	5.88e-06	0.000535%	0.0	1	0.0146	0.00308	0.00104	4.4e-06	0.000132%
**L1569**	**False**	**ZB2**	5.85e-06	0.000533%	0.0	1	0.003	0.000466	0.000589	8.67e-06	0.00026%
**L1175**	**False**	**koll11**	5.85e-06	0.000532%	0.0	1	0.00957	0.00328	0.00297	2.53e-05	0.00076%
**OTU_349**	**True**	**koll11**	5.81e-06	0.000529%	0.0	1	0.00184	0.000434	8.88e-05	1.6e-05	0.000481%
**OTU_4142**	**True**	**koll11**	5.81e-06	0.000529%	0.0	1	0.000543	0.0	0.0	1.26e-05	0.000377%
**L653**	**False**	**koll11**	5.81e-06	0.000529%	0.0	1	0.0214	0.00391	0.00486	6.93e-06	0.000208%
**L1333**	**False**	**ZB2**	5.8e-06	0.000528%	0.0	1	0.004	0.00115	0.000393	7.67e-06	0.00023%
**L1195**	**False**	**koll11**	5.8e-06	0.000528%	0.0	1	0.0029	0.00161	0.000647	3.41e-05	0.00102%
**OTU_8770**	**True**	**OD1**	5.79e-06	0.000527%	0.0	1	0.000156	7.74e-06	0.0	1.25e-05	0.000377%
**OTU_5589**	**True**	**OD1**	5.75e-06	0.000524%	0.0	1	0.000309	8.46e-06	2.69e-05	1.37e-05	0.000411%
**OTU_2334**	**True**	**SJA-4**	5.74e-06	0.000523%	0.0	1	2.79e-06	0.000181	0.000791	2.47e-05	0.000741%

**L1141**	**False**	**koll11**	5.72e-06	0.00052%	0.0	1	0.00777	0.00171	0.000598	1.18e-05	0.000353%
**OTU_6309**	**True**	**ZB2**	5.7e-06	0.000519%	0.0	1	0.000593	8.21e-07	3.52e-07	7.09e-06	0.000213%
**OTU_6584**	**True**	**OD1**	5.67e-06	0.000516%	0.0	1	0.0	0.00016	0.000689	2.27e-05	0.00068%
**L195**	**False**	**ABY1**	5.66e-06	0.000515%	0.0	1	0.00224	0.00068	3.3e-05	7.82e-06	0.000235%
**L1073**	**False**	**koll11**	5.59e-06	0.000509%	0.0	1	0.00216	0.000126	0.0	6.96e-06	0.000209%
**L1598**	**False**	**OD1**	5.59e-06	0.000508%	0.0	1	6e-05	0.00872	0.00274	9.28e-06	0.000278%
**L69**	**False**	**ABY1**	5.59e-06	0.000508%	0.0	1	0.00157	0.00658	0.00546	5.7e-05	0.00171%
**OTU_7350**	**True**	**ABY1**	5.58e-06	0.000508%	0.0	1	1.99e-05	0.000946	0.000354	1.62e-05	0.000485%
**L534**	**False**	**TM6**	5.57e-06	0.000507%	0.0	1	0.00627	0.00164	0.00264	4.05e-05	0.00122%
**OTU_8120**	**True**	**ABY1**	5.56e-06	0.000506%	0.0	1	9.17e-06	0.00164	0.00285	1.21e-05	0.000362%
**L1252**	**False**	**TM7-1**	5.55e-06	0.000505%	0.0	1	0.000602	7.3e-05	0.00729	1.85e-05	0.000554%
**OTU_361**	**True**	**koll11**	5.53e-06	0.000503%	0.0	1	0.00106	0.000212	5.2e-05	1.07e-05	0.00032%
**L269**	**False**	**OD1**	5.51e-06	0.000501%	0.0	1	0.00471	0.00804	0.00326	0.000109	0.00326%
**L285**	**False**	**ABY1**	5.51e-06	0.000501%	0.0	1	0.000397	0.00332	0.00264	2.3e-05	0.000691%
**L1583**	**False**	**ZB2**	5.5e-06	0.000501%	0.0	1	0.00138	0.000173	0.000392	1.66e-05	0.0005%
**OTU_6761**	**True**	**ZB2**	5.5e-06	0.000501%	0.0	1	0.0	2.16e-05	0.000524	1.72e-05	0.000518%
**L1272**	**False**	**TM7**	5.48e-06	0.000498%	0.0	1	0.000933	0.000223	1.63e-05	1.76e-05	0.000529%
**L109**	**False**	**ABY1**	5.45e-06	0.000496%	0.0	1	0.00362	0.000674	0.000322	5.78e-06	0.000173%
**L305**	**False**	**ABY1**	5.41e-06	0.000492%	0.0	1	0.000358	0.00593	0.00252	1.97e-05	0.000593%
**L1887**	**False**	**OD1**	5.4e-06	0.000492%	0.0	1	0.000447	8.81e-05	2.18e-05	1.47e-05	0.000442%
**OTU_268**	**True**	**koll11**	5.39e-06	0.000491%	0.0	1	0.000748	0.000221	0.00226	3.65e-05	0.0011%
**OTU_170**	**True**	**koll11**	5.36e-06	0.000488%	0.0	1	0.00214	0.000366	0.000113	1.05e-05	0.000316%
**L194**	**False**	**ABY1**	5.33e-06	0.000485%	0.0	1	0.00471	0.001	0.000639	8.3e-06	0.000249%
**OTU_428**	**True**	**koll11**	5.31e-06	0.000483%	0.0	1	9.36e-05	2.27e-05	0.000747	2.18e-05	0.000654%
**OTU_485**	**True**	**koll11**	5.29e-06	0.000482%	0.0	1	0.00258	0.0	7.79e-07	1.09e-05	0.000326%
**OTU_162**	**True**	**OD1**	5.29e-06	0.000481%	0.0	1	9.11e-05	0.0	0.0	1.31e-05	0.000392%
**L945**	**False**	**koll11**	5.27e-06	0.000479%	0.0	1	0.00404	0.00108	0.000843	9.67e-06	0.00029%
**OTU_6559**	**True**	**ZB2**	5.24e-06	0.000477%	0.0	1	0.0	4.54e-05	0.00116	1.34e-05	0.000402%
**L1059**	**False**	**koll11**	5.24e-06	0.000477%	0.0	1	0.000685	7.14e-05	5.73e-05	9.46e-06	0.000284%
**L1292**	**False**	**TM7-3**	5.23e-06	0.000476%	0.0	1	0.00138	0.000105	0.00264	3.35e-05	0.001%
**L330**	**False**	**ABY1**	5.23e-06	0.000476%	0.0	1	0.000947	1.11e-05	2.91e-05	6.54e-06	0.000196%
**OTU_6221**	**True**	**ZB2**	5.22e-06	0.000475%	0.0	1	0.000765	4.23e-07	0.0	9.88e-06	0.000296%
**OTU_7948**	**True**	**ABY1**	5.21e-06	0.000474%	0.0	1	0.000366	1.89e-05	2.55e-05	1.29e-05	0.000388%
**L906**	**False**	**koll11**	5.2e-06	0.000473%	0.0	1	0.0022	0.000236	0.000837	2.26e-05	0.000679%
**OTU_250**	**True**	**koll11**	5.2e-06	0.000473%	0.0	1	0.00121	0.000315	0.000335	2.66e-05	0.000799%
**OTU_1383**	**True**	**koll11**	5.19e-06	0.000473%	0.0	1	0.0	0.000832	3.04e-06	1.55e-05	0.000466%
**OTU_7837**	**True**	**ZB2**	5.19e-06	0.000473%	0.0	1	0.000273	0.0	0.0	9.7e-06	0.000291%
**OTU_8327**	**True**	**ZB2**	5.19e-06	0.000472%	0.0	1	0.0	2.59e-05	0.000406	1.55e-05	0.000465%
**OTU_2333**	**True**	**PBS-25**	5.13e-06	0.000467%	0.0	1	0.00117	3.2e-05	0.000141	1.34e-05	0.000403%
**L1022**	**False**	**koll11**	5.13e-06	0.000467%	0.0	1	0.00388	0.000855	0.00145	2.73e-05	0.00082%
**L674**	**False**	**koll11**	5.12e-06	0.000466%	0.0	1	0.0122	0.00199	0.00202	8.3e-06	0.000249%
**OTU_10120**	**True**	**OD1**	5.12e-06	0.000466%	0.0	1	0.000345	0.000107	0.0	1.61e-05	0.000484%
**OTU_60**	**True**	**OD1**	5.11e-06	0.000465%	0.0	1	9e-05	1.85e-05	0.000397	2.59e-05	0.000776%
**L1239**	**False**	**TM7**	5.11e-06	0.000465%	0.0	1	0.0121	0.012	0.021	0.000177	0.00532%
**OTU_6096**	**True**	**ZB2**	5.09e-06	0.000464%	0.0	1	0.00415	0.00131	0.000529	1.52e-05	0.000455%
**OTU_10907**	**True**	**OP11-4**	5.08e-06	0.000462%	0.0	1	0.000156	2.71e-06	0.0	1.09e-05	0.000327%
**L702**	**False**	**koll11**	5.07e-06	0.000461%	0.0	1	0.0012	4.96e-05	0.0	8.2e-06	0.000246%
**OTU_4604**	**True**	**ZB2**	5.06e-06	0.000461%	0.0	1	0.000141	0.0	0.0	9.46e-06	0.000284%
**OTU_6696**	**True**	**ZB2**	5.05e-06	0.00046%	0.0	1	6.26e-06	0.00151	0.00191	3.8e-05	0.00114%
**L701**	**False**	**koll11**	5.05e-06	0.000459%	0.0	1	0.00137	4.98e-05	0.0	6.92e-06	0.000208%
**OTU_166**	**True**	**OP3**	5.04e-06	0.000459%	0.0	1	0.000235	0.0	0.0	8.83e-06	0.000265%
**L1029**	**False**	**koll11**	5.02e-06	0.000457%	0.0	1	0.00923	0.00148	0.00558	1.41e-05	0.000424%
**OTU_6269**	**True**	**OD1**	5e-06	0.000455%	0.0	1	0.00014	0.0	0.000201	3.15e-05	0.000947%
**L1724**	**False**	**Mb-NB09**	4.98e-06	0.000453%	0.0	1	0.00342	0.00151	0.000738	2.19e-05	0.000657%
**OTU_8043**	**True**	**ABY1**	4.98e-06	0.000453%	0.0	1	0.0004	0.0	0.0	8.6e-06	0.000258%
**L717**	**False**	**PBS-25**	4.97e-06	0.000452%	0.0	1	0.0323	0.00436	0.00545	5.96e-06	0.000179%
**OTU_7298**	**True**	**ABY1**	4.94e-06	0.00045%	0.0	1	0.000115	0.00164	0.00102	2.07e-05	0.000621%
**OTU_240**	**True**	**koll11**	4.93e-06	0.000448%	0.0	1	0.00192	4.14e-05	1.08e-05	9.16e-06	0.000275%
**L433**	**False**	**OP11**	4.91e-06	0.000447%	0.0	1	0.00118	0.000142	0.00163	2.52e-05	0.000757%
**OTU_6393**	**True**	**ZB2**	4.88e-06	0.000444%	0.0	1	0.000519	7.94e-05	9.14e-05	1.47e-05	0.000441%
**L1554**	**False**	**ZB2**	4.87e-06	0.000444%	0.0	1	0.00432	0.00135	0.000553	1.42e-05	0.000426%
**OTU_4915**	**True**	**GIF10**	4.86e-06	0.000442%	0.0	1	0.000681	0.000144	5.51e-05	1.4e-05	0.00042%
**L287**	**False**	**ABY1**	4.83e-06	0.00044%	0.0	1	0.000397	0.00251	0.00213	2.72e-05	0.000816%
**OTU_5822**	**True**	**OD1**	4.83e-06	0.00044%	0.0	1	0.000201	1.64e-05	0.0	7.04e-06	0.000211%
**L675**	**False**	**koll11**	4.81e-06	0.000438%	0.0	1	0.00719	0.000585	0.000728	1.12e-05	0.000337%

**L159**	**False**	**ABY1**	4.81e-06	0.000438%	0.0	1	0.000598	0.000102	2.8e-05	1.54e-05	0.000461%
**L1474**	**False**	**ZB2**	4.81e-06	0.000438%	0.0	1	0.00411	0.00172	0.000902	1.68e-05	0.000504%
**L748**	**False**	**PBS-25**	4.8e-06	0.000437%	0.0	1	0.00714	0.000514	0.0025	1.21e-05	0.000363%
**OTU_8060**	**True**	**OD1**	4.79e-06	0.000436%	0.0	1	0.000136	8.97e-05	0.0	2.31e-05	0.000694%
**OTU_10794**	**True**	**OP11-3**	4.77e-06	0.000434%	0.0	1	0.000149	0.0	0.0	1e-05	0.000301%
**L234**	**False**	**ABY1**	4.76e-06	0.000434%	0.0	1	0.00358	0.00117	0.000221	1.45e-05	0.000436%
**L1813**	**False**	**OD1**	4.73e-06	0.00043%	0.0	1	0.00203	0.000949	0.000144	2.05e-05	0.000616%
**OTU_10803**	**True**	**OP11-4**	4.71e-06	0.000429%	0.0	1	0.000238	4.1e-06	0.0	9.64e-06	0.000289%
**OTU_2350**	**True**	**SJA-4**	4.71e-06	0.000429%	0.0	1	0.000587	0.0	0.0	9.64e-06	0.000289%
**L1565**	**False**	**ZB2**	4.69e-06	0.000427%	0.0	1	0.00495	0.00094	0.000738	5.67e-06	0.00017%
**OTU_4996**	**True**	**GIF10**	4.68e-06	0.000426%	0.0	1	1.3e-06	0.000511	0.0	1.33e-05	0.0004%
**OTU_8645**	**True**	**Mb-NB09**	4.68e-06	0.000426%	0.0	1	0.000416	4.49e-05	3.32e-06	6.56e-06	0.000197%
**OTU_472**	**True**	**koll11**	4.67e-06	0.000425%	0.0	1	0.000784	0.0	0.0	1.02e-05	0.000305%
**OTU_5239**	**True**	**OD1**	4.67e-06	0.000425%	0.0	1	0.000622	1.32e-05	5.97e-06	6.51e-06	0.000195%
**L1832**	**False**	**OD1**	4.65e-06	0.000423%	0.0	1	0.002	0.00222	0.000798	0.000108	0.00323%
**L956**	**False**	**koll11**	4.63e-06	0.000422%	0.0	1	0.00131	3.81e-05	5.4e-05	4.51e-06	0.000135%
**OTU_6051**	**True**	**OD1**	4.62e-06	0.00042%	0.0	1	0.000124	0.0	0.0	9.21e-06	0.000276%
**OTU_7480**	**True**	**ABY1**	4.59e-06	0.000418%	0.0	1	0.0	0.000811	0.000513	1.43e-05	0.000428%
**OTU_6306**	**True**	**ZB2**	4.59e-06	0.000418%	0.0	1	0.00145	8.02e-05	0.00045	1.6e-05	0.000481%
**OTU_7962**	**True**	**ABY1**	4.59e-06	0.000417%	0.0	1	0.000766	0.000279	0.000216	3.34e-05	0.001%
**L1181**	**False**	**GIF10**	4.58e-06	0.000417%	0.0	1	0.00197	0.000213	0.000219	7.71e-06	0.000231%
**L1874**	**False**	**OD1**	4.56e-06	0.000415%	0.0	1	0.00108	0.000482	5.3e-05	1.17e-05	0.000353%
**OTU_7046**	**True**	**ABY1**	4.54e-06	0.000413%	0.0	1	0.000861	5.62e-05	0.0	1.08e-05	0.000324%
**OTU_10859**	**True**	**OP11**	4.5e-06	0.000409%	0.0	1	0.000556	0.000101	4.16e-07	1.28e-05	0.000384%
**OTU_7050**	**True**	**ABY1**	4.48e-06	0.000408%	0.0	1	0.000654	0.000227	0.00013	2.75e-05	0.000826%
**OTU_8826**	**True**	**TM7-1**	4.48e-06	0.000407%	0.0	1	0.000454	0.0	0.0	9.08e-06	0.000273%
**OTU_6185**	**True**	**ABY1**	4.47e-06	0.000407%	0.0	1	0.000325	8.41e-05	1.14e-05	1.6e-05	0.00048%
**OTU_7217**	**True**	**ZB2**	4.47e-06	0.000407%	0.0	1	0.000241	0.0	0.0	8.27e-06	0.000248%
**L407**	**False**	**ABY1**	4.46e-06	0.000406%	0.0	1	0.000277	7.44e-06	1.02e-05	9.19e-06	0.000276%
**OTU_7379**	**True**	**ZB2**	4.45e-06	0.000405%	0.0	1	0.000267	0.0	0.0	8.29e-06	0.000249%
**OTU_348**	**True**	**koll11**	4.44e-06	0.000404%	0.0	1	0.000472	1.98e-05	0.000254	2.34e-05	0.000703%
**OTU_8139**	**True**	**EW055**	4.41e-06	0.000401%	0.0	1	0.00138	4.1e-07	2.4e-05	6.99e-06	0.00021%
**OTU_8036**	**True**	**TM7-1**	4.39e-06	0.0004%	0.0	1	0.000563	2.84e-05	3.14e-06	5.23e-06	0.000157%
**OTU_8913**	**True**	**Mb-NB09**	4.39e-06	0.000399%	0.0	1	0.000207	0.0	0.0	7.86e-06	0.000236%
**OTU_8793**	**True**	**OD1**	4.37e-06	0.000398%	0.0	1	3.42e-06	0.000341	0.000551	1.72e-05	0.000516%
**OTU_6343**	**True**	**ZB2**	4.33e-06	0.000394%	0.0	1	0.00215	0.000755	0.00088	4.9e-05	0.00147%
**OTU_9347**	**True**	**Mb-NB09**	4.31e-06	0.000392%	0.0	1	0.00039	4.88e-05	1.42e-07	8.59e-06	0.000258%
**OTU_1425**	**True**	**OP3**	4.31e-06	0.000392%	0.0	1	0.000339	6.85e-05	0.0	9.83e-06	0.000295%
**OTU_3671**	**True**	**kpj58rc**	4.29e-06	0.000391%	0.0	1	0.000387	2.78e-05	7.86e-05	7.84e-06	0.000235%
**OTU_8317**	**True**	**Mb-NB09**	4.29e-06	0.00039%	0.0	1	0.000531	0.000184	3.24e-05	1.79e-05	0.000536%
**OTU_6679**	**True**	**OD1**	4.29e-06	0.00039%	0.0	1	0.00106	0.00019	0.000123	1.73e-05	0.000519%
**OTU_6604**	**True**	**ZB2**	4.28e-06	0.00039%	0.0	1	0.0	6.05e-06	0.000522	1.41e-05	0.000423%
**OTU_165**	**True**	**koll11**	4.26e-06	0.000388%	0.0	1	0.0143	0.00232	0.00165	5.21e-06	0.000156%
**OTU_5847**	**True**	**OD1**	4.26e-06	0.000388%	0.0	1	0.000334	9.99e-06	2.9e-05	1.08e-05	0.000324%
**OTU_6108**	**True**	**ZB2**	4.25e-06	0.000387%	0.0	1	0.0	0.000262	1.77e-06	8.65e-06	0.00026%
**L299**	**False**	**ABY1**	4.25e-06	0.000387%	0.0	1	0.000697	0.00999	0.00618	1.15e-05	0.000344%
**OTU_7022**	**True**	**ABY1**	4.25e-06	0.000386%	0.0	1	0.00153	0.00654	0.00543	4.29e-05	0.00129%
**OTU_5424**	**True**	**ZB2**	4.24e-06	0.000386%	0.0	1	0.000473	7.55e-06	5.98e-05	8.11e-06	0.000243%
**OTU_5575**	**True**	**ZB2**	4.19e-06	0.000381%	0.0	1	0.00012	0.0	5.5e-07	8.71e-06	0.000261%
**OTU_6123**	**True**	**SM2F11**	4.19e-06	0.000381%	0.0	1	0.00023	0.0	0.0	7.3e-06	0.000219%
**OTU_1344**	**True**	**PBS-25**	4.18e-06	0.00038%	0.0	1	3.5e-05	0.0	0.0017	1.15e-05	0.000346%
**L1706**	**False**	**Mb-NB09**	4.18e-06	0.00038%	0.0	1	0.00124	0.000119	3.61e-05	5.36e-06	0.000161%
**L529**	**False**	**TM6**	4.15e-06	0.000378%	0.0	1	0.000164	0.00201	0.000134	2.13e-05	0.000639%
**L565**	**False**	**SJA-4**	4.15e-06	0.000378%	0.0	1	0.00132	3.47e-07	0.000456	1.71e-05	0.000512%
**OTU_3763**	**True**	**OP3**	4.14e-06	0.000377%	0.0	1	0.000192	0.0	0.0	1.01e-05	0.000302%
**OTU_7969**	**True**	**ABY1**	4.12e-06	0.000375%	0.0	1	0.000532	0.000194	8.78e-06	8.97e-06	0.000269%
**OTU_6349**	**True**	**WCHB1-64**	4.12e-06	0.000375%	0.0	1	0.000271	4.72e-05	0.00117	1.94e-05	0.000583%
**L1182**	**False**	**GIF10**	4.11e-06	0.000374%	0.0	1	0.001	6.93e-05	0.000164	1.4e-05	0.00042%
**L498**	**False**	**OP11-4**	4.11e-06	0.000374%	0.0	1	0.000582	4.23e-05	3.88e-05	6.82e-06	0.000205%
**OTU_4064**	**True**	**koll11**	4.08e-06	0.000371%	0.0	1	0.000317	3.03e-05	6.35e-06	1.28e-05	0.000384%
**OTU_7000**	**True**	**ZB2**	4.07e-06	0.000371%	0.0	1	1.39e-06	0.000603	0.000268	1.37e-05	0.000413%
**L1241**	**False**	**TM7**	4.07e-06	0.00037%	0.0	1	0.0117	0.012	0.021	0.000135	0.00404%
**OTU_10777**	**True**	**OP11-3**	4.03e-06	0.000367%	0.0	1	0.000119	0.000339	0.0	2.44e-05	0.000733%
**L1888**	**False**	**OD1**	4.03e-06	0.000367%	0.0	1	0.000325	5.87e-05	8.28e-06	1.13e-05	0.000341%

**OTU_6230**	**True**	**ZB2**	4.03e-06	0.000367%	0.0	1	0.000139	0.0131	0.0056	1.27e-05	0.000381%
**OTU_9264**	**True**	**ABY1**	4.02e-06	0.000366%	0.0	1	0.000249	1.93e-05	2.55e-06	1.01e-05	0.000302%
**L917**	**False**	**OP3**	4.02e-06	0.000366%	0.0	1	0.00053	6.85e-05	0.0	7.43e-06	0.000223%
**L207**	**False**	**ABY1**	4.01e-06	0.000365%	0.0	1	0.00143	3.45e-05	1.77e-05	4.74e-06	0.000142%
**OTU_499**	**True**	**PBS-25**	3.99e-06	0.000363%	0.0	1	0.000313	0.0	0.0	5.05e-06	0.000152%
**OTU_436**	**True**	**koll11**	3.99e-06	0.000363%	0.0	1	0.000386	0.0	1.77e-06	8.45e-06	0.000253%
**L132**	**False**	**OD1**	3.97e-06	0.000361%	0.0	1	0.011	0.0308	0.0167	3.3e-05	0.000991%
**L144**	**False**	**ABY1**	3.96e-06	0.00036%	0.0	1	0.00245	0.00149	0.000234	2.06e-05	0.000617%
**OTU_7670**	**True**	**ABY1**	3.95e-06	0.000359%	0.0	1	0.000341	1.56e-07	1.54e-05	7.78e-06	0.000234%
**L253**	**False**	**ABY1**	3.94e-06	0.000358%	0.0	1	0.000596	0.000139	1.93e-05	8.33e-06	0.00025%
**OTU_8067**	**True**	**OD1**	3.92e-06	0.000357%	0.0	1	0.000268	6.63e-05	0.0	1.23e-05	0.00037%
**OTU_571**	**True**	**koll11**	3.92e-06	0.000357%	0.0	1	0.000224	0.0	0.0	7.11e-06	0.000213%
**L1422**	**False**	**ZB2**	3.91e-06	0.000356%	0.0	1	0.000105	0.00214	0.0022	1.63e-05	0.00049%
**L311**	**False**	**ABY1**	3.91e-06	0.000356%	0.0	1	2.34e-05	0.00134	0.00102	9.92e-06	0.000298%
**L954**	**False**	**koll11**	3.9e-06	0.000355%	0.0	1	0.00135	0.000238	6.32e-05	6.63e-06	0.000199%
**OTU_3064**	**True**	**koll11**	3.89e-06	0.000354%	0.0	1	0.000847	8.98e-05	3.33e-05	5.7e-06	0.000171%
**L1553**	**False**	**ZB2**	3.86e-06	0.000351%	0.0	1	0.00465	0.00135	0.000553	9.47e-06	0.000284%
**L1900**	**False**	**OD1**	3.85e-06	0.000351%	0.0	1	0.00045	0.00016	5.8e-05	2e-05	0.000601%
**OTU_753**	**True**	**koll11**	3.85e-06	0.000351%	0.0	1	0.000426	4.23e-06	0.0	8.16e-06	0.000245%
**L544**	**False**	**SJA-4**	3.85e-06	0.00035%	0.0	1	0.000928	0.000181	0.00167	3.07e-05	0.000922%
**L1866**	**False**	**OD1**	3.85e-06	0.00035%	0.0	1	0.0013	0.000116	0.000142	6.09e-06	0.000183%
**OTU_7970**	**True**	**ABY1**	3.84e-06	0.00035%	0.0	1	1.38e-05	0.000658	0.000339	2.14e-05	0.000642%
**L1192**	**False**	**koll11**	3.82e-06	0.000348%	0.0	1	0.000499	1.13e-05	3.38e-05	6.21e-06	0.000186%
**OTU_1354**	**True**	**PBS-25**	3.81e-06	0.000347%	0.0	1	0.000726	0.0	0.0	8.52e-06	0.000256%
**OTU_4583**	**True**	**ZB2**	3.81e-06	0.000346%	0.0	1	0.000942	8.3e-05	8.82e-05	9.12e-06	0.000274%
**L477**	**False**	**OP11-4**	3.81e-06	0.000346%	0.0	1	0.000855	0.000179	3.94e-05	6.73e-06	0.000202%
**L426**	**False**	**OP11**	3.8e-06	0.000346%	0.0	1	0.00113	0.000674	0.00173	5.73e-05	0.00172%
**L1046**	**False**	**koll11**	3.77e-06	0.000343%	0.0	1	0.00741	0.00147	0.00386	2.07e-05	0.00062%
**L1864**	**False**	**OD1**	3.76e-06	0.000343%	0.0	1	0.00251	0.0015	0.000205	1e-05	0.0003%
**L663**	**False**	**koll11**	3.76e-06	0.000342%	0.0	1	0.00272	0.000121	0.000278	6.29e-06	0.000189%
**OTU_6055**	**True**	**ABY1**	3.75e-06	0.000342%	0.0	1	0.000227	2.32e-05	0.0	7.83e-06	0.000235%
**OTU_6013**	**True**	**ABY1**	3.74e-06	0.00034%	0.0	1	0.000254	0.0	3.68e-06	5.49e-06	0.000165%
**OTU_6**	**True**	**OD1**	3.73e-06	0.00034%	0.0	1	9.54e-05	0.0	0.0	8.95e-06	0.000269%
**OTU_1374**	**True**	**PBS-25**	3.73e-06	0.00034%	0.0	1	0.00176	4.88e-05	0.0	8.77e-06	0.000263%
**OTU_10862**	**True**	**OP11-4**	3.71e-06	0.000338%	0.0	1	0.000725	0.000179	3.94e-05	8.24e-06	0.000247%
**OTU_8782**	**True**	**ABY1**	3.69e-06	0.000336%	0.0	1	0.00207	7.47e-05	0.0	7.68e-06	0.00023%
**L1895**	**False**	**OD1**	3.69e-06	0.000336%	0.0	1	0.00166	0.000556	0.000116	9.65e-06	0.00029%
**L133**	**False**	**ABY1**	3.67e-06	0.000334%	0.0	1	0.00292	0.000456	0.000338	7.35e-06	0.000221%
**L658**	**False**	**koll11**	3.66e-06	0.000334%	0.0	1	0.00442	0.000827	0.00193	1.68e-05	0.000505%
**OTU_67**	**True**	**ZB2**	3.66e-06	0.000333%	0.0	1	9.44e-05	0.0125	0.0182	4.93e-06	0.000148%
**L826**	**False**	**PBS-25**	3.65e-06	0.000332%	0.0	1	0.00479	0.000638	0.000416	8.05e-06	0.000241%
**OTU_173**	**True**	**PBS-25**	3.64e-06	0.000331%	0.0	1	0.00101	9.46e-05	0.000662	3.02e-05	0.000907%
**OTU_861**	**True**	**PBS-25**	3.63e-06	0.000331%	0.0	1	0.000698	0.0	0.0	1.03e-05	0.000311%
**L377**	**False**	**ABY1**	3.62e-06	0.00033%	0.0	1	0.000324	0.0	5.83e-08	5.17e-06	0.000155%
**OTU_6094**	**True**	**ZB2**	3.61e-06	0.000329%	0.0	1	4.62e-05	0.00191	0.00148	1.24e-05	0.000373%
**OTU_6544**	**True**	**ZB2**	3.61e-06	0.000329%	0.0	1	0.0	0.0	0.00066	1.11e-05	0.000332%
**OTU_8108**	**True**	**ABY1**	3.61e-06	0.000329%	0.0	1	0.0002	1.12e-05	1.42e-07	6.35e-06	0.00019%
**OTU_4797**	**True**	**koll11**	3.6e-06	0.000328%	0.0	1	1.62e-06	9.62e-06	0.00031	1.09e-05	0.000326%
**OTU_318**	**True**	**koll11**	3.58e-06	0.000326%	0.0	1	0.00248	0.000327	0.00143	2.18e-05	0.000653%
**L412**	**False**	**ABY1**	3.57e-06	0.000325%	0.0	1	0.000295	0.000514	0.00154	3.13e-05	0.00094%
**OTU_7853**	**True**	**ABY1**	3.54e-06	0.000322%	0.0	1	0.000699	1.76e-05	1.52e-05	7.13e-06	0.000214%
**L770**	**False**	**PBS-25**	3.54e-06	0.000322%	0.0	1	0.00352	0.00035	0.000348	7.74e-06	0.000232%
**OTU_8815**	**True**	**ABY1**	3.53e-06	0.000321%	0.0	1	0.000346	9.92e-06	0.0	1.01e-05	0.000303%
**OTU_906**	**True**	**koll11**	3.52e-06	0.000321%	0.0	1	0.002	0.000388	5.94e-05	8.66e-06	0.00026%
**L1151**	**False**	**koll11**	3.52e-06	0.00032%	0.0	1	0.00187	0.000302	0.00013	8e-06	0.00024%
**OTU_7961**	**True**	**ZB2**	3.51e-06	0.000319%	0.0	1	0.000239	0.000144	8.45e-06	1.33e-05	0.0004%
**OTU_414**	**True**	**PRR-12**	3.5e-06	0.000319%	0.0	1	0.0023	0.000341	0.0	7.78e-06	0.000233%
**OTU_1247**	**True**	**PRR-12**	3.48e-06	0.000317%	0.0	1	0.000882	4.47e-05	1.74e-05	7.15e-06	0.000215%
**OTU_190**	**True**	**koll11**	3.47e-06	0.000316%	0.0	1	0.00338	0.00086	0.00464	2.67e-05	0.000802%
**OTU_2521**	**True**	**koll11**	3.46e-06	0.000315%	0.0	1	0.000242	1.42e-06	2.8e-06	8.07e-06	0.000242%
**OTU_1590**	**True**	**PBS-25**	3.45e-06	0.000314%	0.0	1	0.000323	9.86e-06	0.0	6.88e-06	0.000206%
**OTU_1088**	**True**	**koll11**	3.45e-06	0.000314%	0.0	1	0.000542	0.0	2.8e-06	6.42e-06	0.000193%
**L1540**	**False**	**ZB2**	3.45e-06	0.000314%	0.0	1	0.000377	0.0	0.0	5.36e-06	0.000161%
**L198**	**False**	**ABY1**	3.44e-06	0.000313%	0.0	1	0.00113	0.000269	8.84e-06	5.92e-06	0.000178%

**L1062**	**False**	**koll11**	3.44e-06	0.000313%	0.0	1	0.00191	0.00031	0.000533	1.32e-05	0.000396%
**L1121**	**False**	**koll11**	3.43e-06	0.000312%	0.0	1	0.00197	0.000118	9.12e-05	4.69e-06	0.000141%
**L1673**	**False**	**OD1**	3.42e-06	0.000312%	0.0	1	0.00373	0.00094	0.000595	9.86e-06	0.000296%
**L540**	**False**	**SJA-4**	3.42e-06	0.000312%	0.0	1	0.00345	0.00132	0.00255	6.61e-05	0.00198%
**OTU_7122**	**True**	**ZB2**	3.41e-06	0.00031%	0.0	1	0.000247	1.48e-05	1.57e-05	9.58e-06	0.000288%
**L1727**	**False**	**Mb-NB09**	3.4e-06	0.00031%	0.0	1	0.00305	0.00142	0.000608	1.65e-05	0.000495%
**L1263**	**False**	**TM7-1**	3.39e-06	0.000309%	0.0	1	0.000875	0.0	0.0	4.79e-06	0.000144%
**OTU_6328**	**True**	**ZB2**	3.38e-06	0.000308%	0.0	1	0.0	0.000798	0.000617	1.42e-05	0.000427%
**OTU_5893**	**True**	**OD1**	3.36e-06	0.000306%	0.0	1	0.000143	0.0	0.0	7.04e-06	0.000211%
**L1293**	**False**	**TM7-3**	3.36e-06	0.000306%	0.0	1	0.00138	0.000105	0.00264	2.15e-05	0.000644%
**OTU_4041**	**True**	**koll11**	3.35e-06	0.000305%	0.0	1	0.000474	0.0	0.0	6.81e-06	0.000204%
**OTU_449**	**True**	**koll11**	3.34e-06	0.000304%	0.0	1	0.000348	3.11e-05	0.000252	2.02e-05	0.000607%
**L1438**	**False**	**ZB2**	3.34e-06	0.000304%	0.0	1	0.00174	0.000798	0.000219	1.62e-05	0.000487%
**L1208**	**False**	**koll11**	3.34e-06	0.000304%	0.0	1	0.0005	0.000123	0.0	1.17e-05	0.000351%
**OTU_837**	**True**	**koll11**	3.33e-06	0.000303%	0.0	1	0.000787	0.000195	8.56e-05	1.64e-05	0.000492%
**L636**	**False**	**PBS-25**	3.33e-06	0.000303%	0.0	1	0.00091	7.9e-05	8.32e-06	5.92e-06	0.000178%
**OTU_1305**	**True**	**TM6**	3.32e-06	0.000302%	0.0	1	1.82e-06	0.00188	0.000131	1.37e-05	0.000411%
**OTU_8744**	**True**	**TM7-1**	3.29e-06	0.000299%	0.0	1	0.000115	0.0	0.0	7.82e-06	0.000235%
**OTU_10795**	**True**	**OP11-3**	3.28e-06	0.000298%	0.0	1	6.42e-05	0.0	0.0	8.43e-06	0.000253%
**OTU_6709**	**True**	**OD1**	3.27e-06	0.000298%	0.0	1	0.000193	0.0	0.0	4.52e-06	0.000136%
**OTU_8039**	**True**	**ABY1**	3.27e-06	0.000297%	0.0	1	0.000384	0.0	0.0	5.95e-06	0.000179%
**OTU_418**	**True**	**koll11**	3.26e-06	0.000297%	0.0	1	0.00241	2.94e-05	0.00121	1.27e-05	0.00038%
**OTU_8224**	**True**	**ABY1**	3.26e-06	0.000296%	0.0	1	9.77e-05	0.0	0.0	5.77e-06	0.000173%
**OTU_57**	**True**	**Mb-NB09**	3.25e-06	0.000296%	0.0	1	5.02e-05	5.25e-06	0.0	1.1e-05	0.00033%
**OTU_5443**	**True**	**ZB2**	3.25e-06	0.000296%	0.0	1	0.000663	0.000303	0.000145	2.03e-05	0.00061%
**L1133**	**False**	**koll11**	3.24e-06	0.000295%	0.0	1	0.00137	0.000213	3.12e-05	7.38e-06	0.000221%
**OTU_335**	**True**	**koll11**	3.21e-06	0.000292%	0.0	1	0.00264	0.000501	0.000108	7.22e-06	0.000217%
**OTU_5599**	**True**	**OD1**	3.21e-06	0.000292%	0.0	1	5.25e-05	0.000367	5.64e-06	1.16e-05	0.000347%
**L1340**	**False**	**ZB2**	3.2e-06	0.000292%	0.0	1	0.00109	0.000471	3.47e-05	8.67e-06	0.00026%
**L943**	**False**	**koll11**	3.2e-06	0.000291%	0.0	1	0.00999	0.00204	0.00113	3.34e-06	0.0001%
**L1667**	**False**	**OD1**	3.18e-06	0.00029%	0.0	1	0.000629	0.000145	0.0	5.8e-06	0.000174%
**L679**	**False**	**koll11**	3.18e-06	0.000289%	0.0	1	0.00588	0.000273	0.000676	8.95e-06	0.000269%
**L958**	**False**	**koll11**	3.18e-06	0.000289%	0.0	1	0.000725	2.19e-05	4.92e-05	5.55e-06	0.000166%
**L196**	**False**	**ABY1**	3.17e-06	0.000288%	0.0	1	0.00132	0.00027	8.84e-06	5.18e-06	0.000156%
**L1529**	**False**	**ZB2**	3.16e-06	0.000287%	0.0	1	0.000833	0.000343	0.000152	1.47e-05	0.000442%
**L1091**	**False**	**koll11**	3.15e-06	0.000287%	0.0	1	0.011	0.00187	0.00186	4.79e-06	0.000144%
**L118**	**False**	**OD1**	3.15e-06	0.000287%	0.0	1	0.0134	0.0406	0.023	1.73e-05	0.000519%
**OTU_8643**	**True**	**ABY1**	3.14e-06	0.000286%	0.0	1	0.000299	5.29e-06	5.5e-06	5.53e-06	0.000166%
**L353**	**False**	**OD1**	3.13e-06	0.000285%	0.0	1	0.00037	7.2e-05	1.2e-05	9.96e-06	0.000299%
**OTU_2705**	**True**	**SJA-4**	3.13e-06	0.000285%	0.0	1	0.000148	0.000373	1.69e-05	2.44e-05	0.000731%
**OTU_9026**	**True**	**ABY1**	3.12e-06	0.000284%	0.0	1	0.00041	4.5e-05	9.46e-05	7.75e-06	0.000233%
**OTU_8865**	**True**	**TM7-1**	3.12e-06	0.000284%	0.0	1	0.0	0.000752	0.000233	1.71e-05	0.000512%
**OTU_10899**	**True**	**OP11-4**	3.12e-06	0.000284%	0.0	1	0.000333	1.61e-06	0.0	6.74e-06	0.000202%
**OTU_8146**	**True**	**ABY1**	3.11e-06	0.000283%	0.0	1	0.000159	0.0	0.0	6.44e-06	0.000193%
**OTU_8122**	**True**	**ABY1**	3.11e-06	0.000283%	0.0	1	0.000215	1.23e-05	0.0	6.32e-06	0.00019%
**OTU_6088**	**True**	**ABY1**	3.1e-06	0.000283%	0.0	1	0.000537	7.22e-05	3.04e-05	9.74e-06	0.000292%
**OTU_1449**	**True**	**PBS-25**	3.1e-06	0.000282%	0.0	1	0.0151	0.00193	0.00367	5.37e-06	0.000161%
**OTU_6315**	**True**	**K2-4-19**	3.1e-06	0.000282%	0.0	1	0.000197	0.0	0.0	5.77e-06	0.000173%
**L86**	**False**	**ABY1**	3.1e-06	0.000282%	0.0	1	0.000949	5.62e-05	0.0	6.3e-06	0.000189%
**OTU_343**	**True**	**koll11**	3.09e-06	0.000281%	0.0	1	0.000738	7.6e-05	0.000316	1.52e-05	0.000455%
**OTU_1101**	**True**	**koll11**	3.09e-06	0.000281%	0.0	1	0.00135	0.000124	0.000325	1.13e-05	0.00034%
**L1274**	**False**	**TM7**	3.09e-06	0.000281%	0.0	1	0.00069	0.000223	1.63e-05	1.39e-05	0.000418%
**OTU_1020**	**True**	**PBS-25**	3.08e-06	0.00028%	0.0	1	0.000389	0.0	2.81e-05	7.18e-06	0.000216%
**L463**	**False**	**OP11-4**	3.07e-06	0.00028%	0.0	1	0.000307	8.11e-05	4.27e-06	9.62e-06	0.000289%
**OTU_8527**	**True**	**ABY1**	3.07e-06	0.00028%	0.0	1	4.32e-06	0.000125	0.000315	1.38e-05	0.000416%
**OTU_6095**	**True**	**ZB2**	3.07e-06	0.00028%	0.0	1	0.00143	0.000717	0.000127	1.73e-05	0.000518%
**OTU_5496**	**True**	**ZB2**	3.07e-06	0.00028%	0.0	1	0.00161	0.00107	0.000918	0.000104	0.00311%
**L500**	**False**	**OP11-4**	3.07e-06	0.000279%	0.0	1	0.000335	4.1e-06	0.0	4.63e-06	0.000139%
**OTU_6634**	**True**	**K2-4-19**	3.06e-06	0.000278%	0.0	1	0.000253	6.09e-06	0.0	3.67e-06	0.00011%
**L786**	**False**	**PBS-25**	3.03e-06	0.000276%	0.0	1	0.0012	8.65e-05	0.000295	8.78e-06	0.000264%
**L878**	**False**	**koll11**	3.03e-06	0.000276%	0.0	1	0.00924	0.00314	0.00235	8.99e-06	0.00027%
**OTU_8023**	**True**	**ABY1**	3.03e-06	0.000275%	0.0	1	1.12e-05	0.000249	0.0	9.28e-06	0.000279%
**L1564**	**False**	**ZB2**	3.02e-06	0.000275%	0.0	1	0.00511	0.0012	0.000741	4.04e-06	0.000121%
**OTU_6572**	**True**	**ZB2**	3.01e-06	0.000274%	0.0	1	0.00148	0.000459	4.01e-05	7.55e-06	0.000227%

**OTU_907**	**True**	**koll11**	3e-06	0.000273%	0.0	1	0.00316	0.000176	0.000751	5.43e-06	0.000163%
**OTU_4008**	**True**	**koll11**	2.99e-06	0.000272%	0.0	1	0.00331	0.000229	0.000764	6.99e-06	0.00021%
**L1186**	**False**	**GIF10**	2.98e-06	0.000272%	0.0	1	0.000294	0.0	0.0	4.32e-06	0.00013%
**OTU_8299**	**True**	**ABY1**	2.98e-06	0.000271%	0.0	1	1.42e-05	0.00124	0.00102	7.93e-06	0.000238%
**L1703**	**False**	**OD1**	2.96e-06	0.00027%	0.0	1	0.000893	4.63e-05	0.0	4.93e-06	0.000148%
**L720**	**False**	**PBS-25**	2.95e-06	0.000269%	0.0	1	0.00325	0.000787	0.000492	9.48e-06	0.000285%
**L1339**	**False**	**ZB2**	2.95e-06	0.000268%	0.0	1	0.00164	0.000696	0.000174	8.7e-06	0.000261%
**OTU_386**	**True**	**PBS-25**	2.93e-06	0.000267%	0.0	1	0.00143	0.0	3.33e-06	6.25e-06	0.000187%
**OTU_6041**	**True**	**ZB2**	2.93e-06	0.000267%	0.0	1	0.0015	0.000541	0.000738	3.39e-05	0.00102%
**OTU_315**	**True**	**PBS-25**	2.93e-06	0.000266%	0.0	1	0.0041	0.000778	7.66e-05	4.14e-06	0.000124%
**L586**	**False**	**PRR-12**	2.91e-06	0.000265%	0.0	1	0.00186	5.55e-05	1.25e-05	3.26e-06	9.79e-05%
**OTU_8916**	**True**	**MJK10**	2.91e-06	0.000265%	0.0	1	0.00074	0.00029	0.000562	4.95e-05	0.00148%
**OTU_4028**	**True**	**koll11**	2.9e-06	0.000264%	0.0	1	0.000652	4.96e-05	0.0	6.25e-06	0.000187%
**OTU_6437**	**True**	**ZB2**	2.89e-06	0.000263%	0.0	1	0.000406	8.12e-05	7.05e-06	6.86e-06	0.000206%
**OTU_4177**	**True**	**koll11**	2.89e-06	0.000263%	0.0	1	0.000676	6.15e-05	2.55e-05	7.52e-06	0.000226%
**OTU_477**	**True**	**PRR-12**	2.89e-06	0.000263%	0.0	1	0.000753	0.0	0.0	6.29e-06	0.000189%
**OTU_7009**	**True**	**ZB2**	2.89e-06	0.000263%	0.0	1	0.000359	0.0	0.0	5.84e-06	0.000175%
**OTU_9291**	**True**	**ABY1**	2.89e-06	0.000263%	0.0	1	0.000238	2.02e-05	0.0	7.66e-06	0.00023%
**L242**	**False**	**ABY1**	2.87e-06	0.000262%	0.0	1	0.0065	0.00147	0.000985	6.26e-06	0.000188%
**OTU_5449**	**True**	**OD1**	2.87e-06	0.000262%	0.0	1	0.000655	9.22e-05	3.41e-05	5.57e-06	0.000167%
**L1620**	**False**	**ZB2**	2.86e-06	0.000261%	0.0	1	0.00171	0.00224	0.000688	4.13e-05	0.00124%
**OTU_495**	**True**	**koll11**	2.86e-06	0.000261%	0.0	1	0.00077	0.00015	0.0	7.66e-06	0.00023%
**OTU_8164**	**True**	**Mb-NB09**	2.86e-06	0.00026%	0.0	1	0.000572	0.00104	4.89e-05	1.45e-05	0.000434%
**OTU_9271**	**True**	**ABY1**	2.84e-06	0.000258%	0.0	1	0.000108	7.06e-07	0.0	5.94e-06	0.000178%
**L102**	**False**	**ABY1**	2.84e-06	0.000258%	0.0	1	0.00131	0.000126	1.21e-05	4.67e-06	0.00014%
**OTU_8664**	**True**	**OD1**	2.82e-06	0.000256%	0.0	1	0.000101	1.06e-06	0.0	7.47e-06	0.000224%
**L747**	**False**	**PBS-25**	2.82e-06	0.000256%	0.0	1	0.00763	0.000621	0.00253	6.5e-06	0.000195%
**L848**	**False**	**koll11**	2.81e-06	0.000256%	0.0	1	0.00598	0.000602	0.00294	1.19e-05	0.000357%
**L550**	**False**	**SJA-4**	2.81e-06	0.000256%	0.0	1	0.000551	0.0	0.00012	1.03e-05	0.00031%
**OTU_2347**	**True**	**SJA-4**	2.8e-06	0.000255%	0.0	1	0.000595	5.49e-05	4.49e-05	9.34e-06	0.00028%
**OTU_4757**	**True**	**ZB2**	2.8e-06	0.000255%	0.0	1	0.00034	0.000274	1.98e-05	1.94e-05	0.000584%
**OTU_451**	**True**	**PRR-10**	2.8e-06	0.000255%	0.0	1	0.00827	0.00122	0.000468	3.82e-06	0.000115%
**OTU_8832**	**True**	**Mb-NB09**	2.79e-06	0.000254%	0.0	1	0.00048	6.4e-06	1.81e-05	7e-06	0.00021%
**L1219**	**False**	**koll11**	2.78e-06	0.000253%	0.0	1	0.000155	0.000275	0.0	1.85e-05	0.000554%
**L398**	**False**	**ABY1**	2.78e-06	0.000253%	0.0	1	0.001	0.000276	0.000132	1.14e-05	0.000342%
**OTU_5902**	**True**	**OD1**	2.77e-06	0.000252%	0.0	1	0.00038	1.77e-07	5.48e-06	4.04e-06	0.000121%
**OTU_4014**	**True**	**GIF10**	2.77e-06	0.000252%	0.0	1	0.0	9.68e-05	0.000368	1.7e-05	0.000511%
**OTU_996**	**True**	**PBS-25**	2.77e-06	0.000252%	0.0	1	0.000551	1.6e-05	0.0	5.43e-06	0.000163%
**OTU_552**	**True**	**koll11**	2.77e-06	0.000252%	0.0	1	0.00054	6.39e-05	1.61e-05	9.98e-06	0.000299%
**OTU_395**	**True**	**koll11**	2.76e-06	0.000252%	0.0	1	0.000933	2.89e-05	6.94e-05	4.13e-06	0.000124%
**OTU_9077**	**True**	**OD1**	2.76e-06	0.000251%	0.0	1	0.000346	0.0	2.8e-06	7.59e-06	0.000228%
**OTU_288**	**True**	**PBS-25**	2.75e-06	0.00025%	0.0	1	0.00182	0.000787	0.000488	1.83e-05	0.00055%
**L339**	**False**	**OD1**	2.74e-06	0.00025%	0.0	1	0.000674	5.8e-05	9.01e-06	6.34e-06	0.00019%
**OTU_7973**	**True**	**ABY1**	2.74e-06	0.00025%	0.0	1	0.0	0.000638	0.000458	1.45e-05	0.000436%
**OTU_600**	**True**	**PBS-25**	2.74e-06	0.000249%	0.0	1	0.000446	3.22e-06	2.47e-05	6.35e-06	0.00019%
**OTU_5236**	**True**	**OD1**	2.73e-06	0.000249%	0.0	1	0.000443	5.63e-05	2.8e-06	4.67e-06	0.00014%
**OTU_299**	**True**	**PBS-25**	2.72e-06	0.000247%	0.0	1	0.00119	0.000211	0.000225	7.6e-06	0.000228%
**L1484**	**False**	**ZB2**	2.72e-06	0.000247%	0.0	1	0.00344	0.00123	0.000578	9.09e-06	0.000273%
**OTU_371**	**True**	**koll11**	2.72e-06	0.000247%	0.0	1	0.000562	0.000121	0.000266	1.87e-05	0.000562%
**OTU_8249**	**True**	**ABY1**	2.7e-06	0.000245%	0.0	1	0.00169	0.000318	0.000188	9.09e-06	0.000273%
**OTU_7966**	**True**	**ZB2**	2.7e-06	0.000245%	0.0	1	0.0	0.000347	6.39e-05	7.97e-06	0.000239%
**OTU_35**	**True**	**GIF10**	2.69e-06	0.000245%	0.0	1	0.000728	0.0	1.17e-07	4.33e-06	0.00013%
**OTU_2207**	**True**	**PBS-25**	2.69e-06	0.000245%	0.0	1	0.00158	9.2e-05	0.000121	9.17e-06	0.000275%
**OTU_9016**	**True**	**ABY1**	2.68e-06	0.000244%	0.0	1	0.000206	1.52e-05	5.83e-08	5.59e-06	0.000168%
**OTU_833**	**True**	**koll11**	2.66e-06	0.000242%	0.0	1	0.00101	8.59e-06	1.77e-05	2.96e-06	8.88e-05%
**OTU_1263**	**True**	**PBS-25**	2.65e-06	0.000241%	0.0	1	0.000387	0.0	0.0	5.62e-06	0.000169%
**OTU_6568**	**True**	**ZB2**	2.65e-06	0.000241%	0.0	1	0.000209	0.0	0.0	4.86e-06	0.000146%
**OTU_7945**	**True**	**ABY1**	2.65e-06	0.000241%	0.0	1	0.000366	8.14e-06	1.77e-05	5.72e-06	0.000172%
**OTU_7944**	**True**	**ABY1**	2.64e-06	0.000241%	0.0	1	0.00043	0.0	2.43e-05	6.06e-06	0.000182%
**L387**	**False**	**Bacteria**	2.64e-06	0.000241%	0.0	1	0.848	0.853	0.92	2.04e-05	0.000613%
**OTU_2434**	**True**	**SBRH58**	2.64e-06	0.00024%	0.0	1	0.0	9.97e-05	0.0	6.23e-06	0.000187%
**L1730**	**False**	**Mb-NB09**	2.63e-06	0.00024%	0.0	1	0.00235	0.0013	0.000604	2.22e-05	0.000666%
**L1612**	**False**	**ZB2**	2.62e-06	0.000239%	0.0	1	0.00122	8.34e-05	2.51e-05	3.67e-06	0.00011%
**L252**	**False**	**ABY1**	2.62e-06	0.000238%	0.0	1	0.000596	0.000235	3.87e-05	8.92e-06	0.000268%
**OTU_5654**	**True**	**OD1**	2.61e-06	0.000237%	0.0	1	0.000102	0.0	0.0	5.52e-06	0.000166%

**OTU_9156**	**True**	**Mb-NB09**	2.61e-06	0.000237%	0.0	1	0.000215	2.78e-05	0.0	8.04e-06	0.000241%
**OTU_9728**	**True**	**ZB2**	2.6e-06	0.000237%	0.0	1	0.000136	8.63e-06	0.0	6.78e-06	0.000203%
**L206**	**False**	**ABY1**	2.59e-06	0.000236%	0.0	1	0.00165	0.000284	1.77e-05	4.73e-06	0.000142%
**L204**	**False**	**ABY1**	2.58e-06	0.000235%	0.0	1	0.00165	0.000317	0.000605	1.23e-05	0.000369%
**L1077**	**False**	**koll11**	2.57e-06	0.000234%	0.0	1	0.00178	0.000112	0.0	4.26e-06	0.000128%
**L1666**	**False**	**OD1**	2.57e-06	0.000234%	0.0	1	0.00295	0.00181	0.00056	1.23e-05	0.00037%
**OTU_7005**	**True**	**ZB2**	2.57e-06	0.000234%	0.0	1	0.000267	0.0	0.0	5.49e-06	0.000165%
**OTU_10779**	**True**	**OP11-4**	2.57e-06	0.000234%	0.0	1	0.000849	0.000174	4.02e-05	4.52e-06	0.000136%
**OTU_7212**	**True**	**ZB2**	2.56e-06	0.000233%	0.0	1	0.000148	3.99e-06	2.35e-07	5.72e-06	0.000172%
**OTU_5493**	**True**	**ZB2**	2.55e-06	0.000232%	0.0	1	0.000566	0.0	0.0	3.18e-06	9.54e-05%
**L1726**	**False**	**Mb-NB09**	2.55e-06	0.000232%	0.0	1	0.00321	0.00148	0.000738	1.25e-05	0.000374%
**OTU_5588**	**True**	**OD1**	2.54e-06	0.000232%	0.0	1	0.000619	1.32e-05	8.64e-06	4.58e-06	0.000138%
**OTU_5931**	**True**	**OD1**	2.54e-06	0.000231%	0.0	1	0.000801	0.000116	6.94e-05	6.28e-06	0.000188%
**L1695**	**False**	**Mb-NB09**	2.54e-06	0.000231%	0.0	1	0.000955	0.000236	4.65e-05	4.77e-06	0.000143%
**L1858**	**False**	**OD1**	2.53e-06	0.00023%	0.0	1	0.000259	8.2e-07	1.54e-05	4.48e-06	0.000134%
**OTU_7138**	**True**	**ABY1**	2.52e-06	0.00023%	0.0	1	5.73e-05	1.06e-05	0.000782	8.59e-06	0.000258%
**L1521**	**False**	**ZB2**	2.52e-06	0.000229%	0.0	1	0.00203	0.000663	8.04e-05	5.03e-06	0.000151%
**OTU_7383**	**True**	**ABY1**	2.52e-06	0.000229%	0.0	1	0.000239	1.9e-05	0.0	5.8e-06	0.000174%
**L15**	**False**	**ABY1**	2.51e-06	0.000229%	0.0	1	0.00125	0.00078	0.0	1.05e-05	0.000316%
**L554**	**False**	**SJA-4**	2.51e-06	0.000228%	0.0	1	0.000595	0.00027	4.59e-05	1.6e-05	0.00048%
**OTU_5495**	**True**	**ZB2**	2.51e-06	0.000228%	0.0	1	0.000589	0.000128	7.77e-06	6.89e-06	0.000207%
**L1001**	**False**	**koll11**	2.5e-06	0.000228%	0.0	1	0.00164	0.000538	0.000805	2.39e-05	0.000716%
**OTU_29**	**True**	**ZB2**	2.5e-06	0.000228%	0.0	1	0.00151	0.000322	0.000462	9.07e-06	0.000272%
**L1819**	**False**	**OD1**	2.5e-06	0.000228%	0.0	1	0.00112	0.000233	2.88e-05	6.57e-06	0.000197%
**OTU_6057**	**True**	**ABY1**	2.49e-06	0.000227%	0.0	1	0.000323	6.92e-05	0.0	1.13e-05	0.000338%
**L238**	**False**	**ABY1**	2.48e-06	0.000226%	0.0	1	0.000741	1.77e-06	2.25e-05	4.8e-06	0.000144%
**OTU_4894**	**True**	**GIF10**	2.48e-06	0.000226%	0.0	1	0.000191	0.0	0.0	5.23e-06	0.000157%
**L1850**	**False**	**OD1**	2.47e-06	0.000225%	0.0	1	0.000265	7.74e-06	6.45e-05	9.27e-06	0.000278%
**L886**	**False**	**koll11**	2.47e-06	0.000225%	0.0	1	0.00146	0.00145	0.000255	1.73e-05	0.000519%
**OTU_8159**	**True**	**ABY1**	2.46e-06	0.000224%	0.0	1	0.0	0.000228	5.45e-06	9.57e-06	0.000287%
**OTU_5515**	**True**	**ZB2**	2.44e-06	0.000222%	0.0	1	0.000305	1.6e-06	5.5e-07	5.37e-06	0.000161%
**L1896**	**False**	**OD1**	2.42e-06	0.00022%	0.0	1	0.000912	0.000219	2.74e-05	5.1e-06	0.000153%
**OTU_3125**	**True**	**koll11**	2.42e-06	0.00022%	0.0	1	0.000353	0.000403	1.57e-05	1.76e-05	0.000528%
**L1517**	**False**	**OD1**	2.42e-06	0.00022%	0.0	1	0.00053	0.000136	0.00101	1.65e-05	0.000495%
**OTU_2634**	**True**	**PBS-25**	2.41e-06	0.000219%	0.0	1	0.00284	0.000459	0.000428	6.55e-06	0.000196%
**OTU_5995**	**True**	**SM2F11**	2.41e-06	0.000219%	0.0	1	0.000209	3.15e-05	0.000161	1.65e-05	0.000496%
**OTU_6591**	**True**	**ZB2**	2.4e-06	0.000219%	0.0	1	0.000285	0.0	0.0	4.86e-06	0.000146%
**OTU_4491**	**True**	**koll11**	2.4e-06	0.000218%	0.0	1	0.000225	0.0	0.0	5.06e-06	0.000152%
**OTU_6662**	**True**	**ZB2**	2.38e-06	0.000217%	0.0	1	6.32e-07	0.000395	0.00104	9.35e-06	0.000281%
**L1803**	**False**	**OD1**	2.38e-06	0.000216%	0.0	1	0.000614	0.0	0.0	3.19e-06	9.56e-05%
**OTU_5240**	**True**	**ZB2**	2.38e-06	0.000216%	0.0	1	0.000351	0.000183	7.2e-06	8.53e-06	0.000256%
**OTU_8868**	**True**	**ABY1**	2.37e-06	0.000216%	0.0	1	0.00013	2.44e-05	6.59e-07	6.19e-06	0.000186%
**OTU_6835**	**True**	**OD1**	2.37e-06	0.000216%	0.0	1	0.000132	0.00055	1.29e-05	1.37e-05	0.000411%
**OTU_6231**	**True**	**ZB2**	2.37e-06	0.000216%	0.0	1	3.34e-06	0.000318	1.02e-06	6.56e-06	0.000197%
**OTU_608**	**True**	**koll11**	2.37e-06	0.000215%	0.0	1	0.00323	0.000533	0.00079	6.21e-06	0.000186%
**L1196**	**False**	**koll11**	2.36e-06	0.000215%	0.0	1	0.00269	0.00159	0.000608	1.57e-05	0.000472%
**OTU_5881**	**True**	**OD1**	2.35e-06	0.000214%	0.0	1	0.000605	0.000171	0.000202	1.12e-05	0.000335%
**OTU_6966**	**True**	**OD1**	2.34e-06	0.000213%	0.0	1	0.000234	0.0	0.0	4.7e-06	0.000141%
**OTU_52**	**True**	**ZB2**	2.34e-06	0.000213%	0.0	1	0.000143	7.1e-06	0.0	5.6e-06	0.000168%
**L935**	**False**	**koll11**	2.33e-06	0.000212%	0.0	1	0.000865	0.000154	0.0	5.37e-06	0.000161%
**OTU_7327**	**True**	**ABY1**	2.32e-06	0.000212%	0.0	1	4.06e-07	0.000516	7.37e-05	5.26e-06	0.000158%
**OTU_8012**	**True**	**ZB2**	2.32e-06	0.000211%	0.0	1	0.00148	0.00085	0.000156	1.63e-05	0.000488%
**OTU_5343**	**True**	**ZB2**	2.31e-06	0.00021%	0.0	1	0.000561	6.67e-05	2.23e-05	4.98e-06	0.000149%
**L1590**	**False**	**OD1**	2.31e-06	0.00021%	0.0	1	0.0109	0.0144	0.00629	2.71e-05	0.000813%
**L1616**	**False**	**ZB2**	2.3e-06	0.000209%	0.0	1	0.000492	1.85e-05	4.63e-05	5.75e-06	0.000172%
**OTU_7843**	**True**	**ABY1**	2.29e-06	0.000208%	0.0	1	0.00278	0.00169	0.00016	9.61e-06	0.000288%
**L460**	**False**	**OP11-4**	2.28e-06	0.000208%	0.0	1	0.000689	9.41e-05	0.00053	1.4e-05	0.000419%
**OTU_328**	**True**	**koll11**	2.28e-06	0.000207%	0.0	1	0.000341	0.0	0.0	4.68e-06	0.00014%
**OTU_8163**	**True**	**ABY1**	2.27e-06	0.000207%	0.0	1	0.000198	0.000152	0.0	1.28e-05	0.000385%
**L1075**	**False**	**koll11**	2.26e-06	0.000206%	0.0	1	0.00199	0.000112	0.0	3.25e-06	9.74e-05%
**L558**	**False**	**SJA-4**	2.26e-06	0.000206%	0.0	1	0.00186	0.000761	0.0006	2.66e-05	0.000799%
**OTU_1583**	**True**	**koll11**	2.26e-06	0.000206%	0.0	1	0.000223	3.19e-06	1.23e-05	5.2e-06	0.000156%
**OTU_8180**	**True**	**EW055**	2.26e-06	0.000205%	0.0	1	0.0	0.0	0.000806	5.78e-06	0.000174%
**OTU_7231**	**True**	**ABY1**	2.25e-06	0.000205%	0.0	1	0.000235	0.0	0.0	3.46e-06	0.000104%
**OTU_5490**	**True**	**Mb-NB09**	2.24e-06	0.000204%	0.0	1	0.000622	4.53e-05	0.0	5.29e-06	0.000159%

**L655**	**False**	**koll11**	2.23e-06	0.000203%	0.0	1	0.00854	0.00113	0.00272	4.24e-06	0.000127%
**OTU_2809**	**True**	**SJA-4**	2.21e-06	0.000201%	0.0	1	0.0	0.000186	0.0	6.09e-06	0.000183%
**OTU_7052**	**True**	**ABY1**	2.21e-06	0.000201%	0.0	1	0.0009	7.76e-05	0.0	5.6e-06	0.000168%
**L1631**	**False**	**ZB2**	2.2e-06	0.0002%	0.0	1	0.00296	0.00174	0.00124	3.38e-05	0.00101%
**OTU_2801**	**True**	**PBS-25**	2.2e-06	0.0002%	0.0	1	1.37e-05	0.000307	5.83e-08	6.69e-06	0.000201%
**L734**	**False**	**PBS-25**	2.2e-06	0.0002%	0.0	1	0.0053	0.000989	0.000302	2.87e-06	8.63e-05%
**L1344**	**False**	**ZB2**	2.2e-06	0.0002%	0.0	1	0.000483	0.000277	1.98e-05	9.79e-06	0.000294%
**L1185**	**False**	**GIF10**	2.19e-06	0.000199%	0.0	1	0.000975	0.000144	5.51e-05	3.81e-06	0.000114%
**OTU_5528**	**True**	**ZB2**	2.18e-06	0.000199%	0.0	1	0.00017	4.08e-05	6.68e-06	6.54e-06	0.000196%
**L1628**	**False**	**ZB2**	2.18e-06	0.000199%	0.0	1	0.00493	0.00259	0.00139	1.62e-05	0.000486%
**OTU_1282**	**True**	**PRR-12**	2.18e-06	0.000199%	0.0	1	0.00057	3.65e-05	1.57e-06	4.71e-06	0.000141%
**L1351**	**False**	**OD1**	2.17e-06	0.000198%	0.0	1	0.00302	0.00146	0.00121	2.65e-05	0.000795%
**L1648**	**False**	**ZB2**	2.17e-06	0.000198%	0.0	1	0.000375	0.000153	8.45e-06	6.21e-06	0.000187%
**L1087**	**False**	**koll11**	2.16e-06	0.000197%	0.0	1	0.000572	0.000143	6.83e-05	8.15e-06	0.000245%
**OTU_6469**	**True**	**OD1**	2.16e-06	0.000196%	0.0	1	0.00276	0.000486	0.000254	5.36e-06	0.000161%
**OTU_7975**	**True**	**ABY1**	2.16e-06	0.000196%	0.0	1	0.00093	0.000584	5.62e-06	8.13e-06	0.000244%
**OTU_5531**	**True**	**OD1**	2.15e-06	0.000196%	0.0	1	0.000271	1.02e-06	0.0	3.85e-06	0.000115%
**OTU_6282**	**True**	**OD1**	2.15e-06	0.000196%	0.0	1	0.0	0.000119	0.0	5.87e-06	0.000176%
**OTU_310**	**True**	**koll11**	2.15e-06	0.000196%	0.0	1	0.000763	0.000213	3.12e-05	9.59e-06	0.000288%
**L1525**	**False**	**ZB2**	2.15e-06	0.000196%	0.0	1	0.000436	0.000204	3.97e-05	9.34e-06	0.00028%
**OTU_227**	**True**	**koll11**	2.14e-06	0.000195%	0.0	1	0.00157	0.000279	0.000281	8.24e-06	0.000247%
**OTU_7983**	**True**	**ABY1**	2.14e-06	0.000195%	0.0	1	0.0	0.000356	0.000348	1.3e-05	0.000392%
**L662**	**False**	**koll11**	2.14e-06	0.000195%	0.0	1	0.0034	0.000712	0.000405	5.6e-06	0.000168%
**OTU_4887**	**True**	**koll11**	2.14e-06	0.000195%	0.0	1	0.000407	3.7e-06	3.38e-05	4.14e-06	0.000124%
**OTU_8786**	**True**	**ABY1**	2.14e-06	0.000194%	0.0	1	6.31e-05	0.000248	4.6e-06	1.22e-05	0.000367%
**OTU_588**	**True**	**koll11**	2.13e-06	0.000194%	0.0	1	0.000186	8.07e-06	0.0	4.51e-06	0.000135%
**L740**	**False**	**PBS-25**	2.13e-06	0.000194%	0.0	1	0.00118	0.000103	0.000935	1.59e-05	0.000476%
**L1643**	**False**	**ZB2**	2.13e-06	0.000194%	0.0	1	0.001	0.000309	0.000815	2.04e-05	0.000613%
**OTU_6779**	**True**	**ZB2**	2.13e-06	0.000194%	0.0	1	4.01e-06	0.0	0.00018	5.84e-06	0.000175%
**L772**	**False**	**PBS-25**	2.13e-06	0.000194%	0.0	1	0.00313	0.00035	0.00032	5.12e-06	0.000154%
**L1067**	**False**	**koll11**	2.13e-06	0.000193%	0.0	1	0.00064	3.78e-05	0.000154	6.62e-06	0.000199%
**L1394**	**False**	**ZB2**	2.12e-06	0.000193%	0.0	1	0.00388	0.00283	0.00171	2.93e-05	0.00088%
**OTU_8932**	**True**	**Mb-NB09**	2.11e-06	0.000192%	0.0	1	0.000444	0.000101	2.21e-05	6.51e-06	0.000195%
**OTU_6089**	**True**	**SM2F11**	2.11e-06	0.000192%	0.0	1	0.000253	9.43e-05	0.0	1.03e-05	0.000311%
**L684**	**False**	**koll11**	2.11e-06	0.000192%	0.0	1	0.000839	2.9e-05	2.42e-05	4.79e-06	0.000144%
**OTU_7592**	**True**	**ZB2**	2.11e-06	0.000192%	0.0	1	0.0	0.0	0.000241	5.33e-06	0.00016%
**OTU_9808**	**True**	**TM7-1**	2.11e-06	0.000192%	0.0	1	0.000243	0.0	0.0	4.28e-06	0.000128%
**OTU_7092**	**True**	**OD1**	2.09e-06	0.00019%	0.0	1	3.38e-05	0.000282	8.26e-06	8.67e-06	0.00026%
**OTU_4959**	**True**	**ZB2**	2.09e-06	0.00019%	0.0	1	0.000259	1.11e-05	7.7e-06	7.34e-06	0.00022%
**OTU_7233**	**True**	**ABY1**	2.08e-06	0.000189%	0.0	1	0.00315	0.000925	0.000203	7.29e-06	0.000219%
**OTU_8222**	**True**	**ABY1**	2.07e-06	0.000188%	0.0	1	8.92e-05	0.0	5.83e-08	3.75e-06	0.000113%
**OTU_998**	**True**	**PBS-25**	2.07e-06	0.000188%	0.0	1	0.000369	0.0	0.0	4.29e-06	0.000129%
**OTU_8928**	**True**	**TM7-1**	2.06e-06	0.000188%	0.0	1	0.000295	6.96e-05	1.81e-05	8.35e-06	0.000251%
**L754**	**False**	**PBS-25**	2.06e-06	0.000187%	0.0	1	0.00138	0.000117	0.00176	1.29e-05	0.000388%
**L1670**	**False**	**OD1**	2.04e-06	0.000186%	0.0	1	0.00232	0.00167	0.00056	1.73e-05	0.000518%
**OTU_2571**	**True**	**PBS-25**	2.03e-06	0.000185%	0.0	1	0.000213	1.2e-05	4.1e-06	5.15e-06	0.000154%
**OTU_5423**	**True**	**OD1**	2.03e-06	0.000185%	0.0	1	7.85e-05	0.0	0.0	4.1e-06	0.000123%
**L462**	**False**	**OP11-4**	2.03e-06	0.000185%	0.0	1	0.000639	9.12e-05	8.46e-05	5.15e-06	0.000155%
**OTU_178**	**True**	**koll11**	2.02e-06	0.000184%	0.0	1	0.00102	0.000171	0.000229	8.56e-06	0.000257%
**OTU_1094**	**True**	**PBS-25**	2.02e-06	0.000184%	0.0	1	0.00115	0.000117	5.54e-05	4.34e-06	0.00013%
**OTU_6542**	**True**	**OD1**	2.02e-06	0.000184%	0.0	1	0.000204	0.000695	3.52e-05	9.01e-06	0.00027%
**OTU_262**	**True**	**koll11**	2.02e-06	0.000184%	0.0	1	0.00746	0.00112	0.00168	4.88e-06	0.000147%
**L1734**	**False**	**Mb-NB09**	2.01e-06	0.000183%	0.0	1	0.000779	0.00104	4.89e-05	9.34e-06	0.00028%
**OTU_3998**	**True**	**koll11**	2.01e-06	0.000183%	0.0	1	0.000215	1.8e-05	3.95e-05	6.03e-06	0.000181%
**OTU_10765**	**True**	**OP11-4**	2.01e-06	0.000183%	0.0	1	0.000593	3.84e-05	6.05e-05	2.82e-06	8.46e-05%
**OTU_7343**	**True**	**ABY1**	2e-06	0.000182%	0.0	1	4.06e-07	0.00062	0.00089	1.02e-05	0.000307%
**L657**	**False**	**koll11**	2e-06	0.000182%	0.0	1	0.00523	0.000899	0.00195	7.32e-06	0.00022%
**OTU_307**	**True**	**koll11**	1.99e-06	0.000181%	0.0	1	0.0034	0.000292	0.000849	4.62e-06	0.000139%
**L1070**	**False**	**koll11**	1.98e-06	0.00018%	0.0	1	0.000671	5.59e-06	2.98e-06	4.67e-06	0.00014%
**OTU_5951**	**True**	**OD1**	1.97e-06	0.00018%	0.0	1	0.000143	1.43e-05	5.49e-06	5.4e-06	0.000162%
**OTU_6598**	**True**	**ZB2**	1.97e-06	0.000179%	0.0	1	0.0	0.000186	0.000621	6.91e-06	0.000207%
**L1134**	**False**	**koll11**	1.97e-06	0.000179%	0.0	1	0.000988	0.000213	3.12e-05	6.34e-06	0.00019%
**L1100**	**False**	**koll11**	1.97e-06	0.000179%	0.0	1	0.00147	0.000202	0.000565	9.17e-06	0.000275%
**OTU_2626**	**True**	**SJA-4**	1.96e-06	0.000179%	0.0	1	2.99e-05	3.47e-07	0.000456	6.45e-06	0.000194%
**OTU_6672**	**True**	**ABY1**	1.96e-06	0.000179%	0.0	1	6.06e-05	0.000521	0.000177	1.36e-05	0.000408%

**L719**	**False**	**PBS-25**	1.96e-06	0.000178%	0.0	1	0.0036	0.000868	0.000571	5.67e-06	0.00017%
**L1481**	**False**	**ZB2**	1.96e-06	0.000178%	0.0	1	0.000406	0.0	0.0	3.57e-06	0.000107%
**L351**	**False**	**OD1**	1.93e-06	0.000176%	0.0	1	0.0005	9.64e-05	1.26e-05	4.44e-06	0.000133%
**OTU_858**	**True**	**koll11**	1.93e-06	0.000176%	0.0	1	0.000338	2.09e-05	0.0	6.63e-06	0.000199%
**OTU_429**	**True**	**PBS-25**	1.92e-06	0.000175%	0.0	1	0.000511	7.06e-06	0.000266	1.01e-05	0.000303%
**OTU_891**	**True**	**PRR-12**	1.92e-06	0.000174%	0.0	1	0.001	0.000115	5.56e-05	5.24e-06	0.000157%
**OTU_8867**	**True**	**Mb-NB09**	1.91e-06	0.000174%	0.0	1	0.000725	0.000254	0.000535	2.85e-05	0.000855%
**OTU_8198**	**True**	**OD1**	1.91e-06	0.000174%	0.0	1	0.000134	3.14e-05	2.67e-06	7.07e-06	0.000212%
**L1127**	**False**	**koll11**	1.9e-06	0.000173%	0.0	1	0.000779	8.2e-05	2.84e-05	5.64e-06	0.000169%
**OTU_811**	**True**	**koll11**	1.9e-06	0.000173%	0.0	1	0.000345	0.0	1.12e-05	2.79e-06	8.37e-05%
**L915**	**False**	**OP3**	1.9e-06	0.000173%	0.0	1	0.000992	6.85e-05	3.89e-06	2.54e-06	7.61e-05%
**OTU_6922**	**True**	**OD1**	1.86e-06	0.00017%	0.0	1	0.000233	5.13e-05	2.54e-05	7.84e-06	0.000235%
**OTU_6567**	**True**	**ZB2**	1.86e-06	0.000169%	0.0	1	0.0015	0.000661	0.000385	1.6e-05	0.00048%
**L1492**	**False**	**ZB2**	1.86e-06	0.000169%	0.0	1	0.000719	0.000129	2.05e-05	2.79e-06	8.38e-05%
**L1201**	**False**	**koll11**	1.85e-06	0.000169%	0.0	1	0.00113	0.000403	8.74e-05	6.64e-06	0.000199%
**L609**	**False**	**PRR-12**	1.85e-06	0.000169%	0.0	1	0.000683	0.0	2.96e-06	2.66e-06	7.99e-05%
**OTU_6161**	**True**	**ABY1**	1.85e-06	0.000168%	0.0	1	0.000125	0.0	0.0	3.93e-06	0.000118%
**OTU_5514**	**True**	**OD1**	1.84e-06	0.000168%	0.0	1	0.000117	1.47e-05	0.0	4.99e-06	0.00015%
**OTU_4389**	**True**	**koll11**	1.84e-06	0.000168%	0.0	1	0.00579	0.000271	0.000676	5.37e-06	0.000161%
**OTU_9070**	**True**	**ABY1**	1.84e-06	0.000168%	0.0	1	4.37e-06	0.000222	0.00022	1.14e-05	0.000341%
**L340**	**False**	**OD1**	1.84e-06	0.000168%	0.0	1	0.000447	3.18e-05	4.53e-06	4.54e-06	0.000136%
**OTU_549**	**True**	**koll11**	1.84e-06	0.000167%	0.0	1	0.000197	0.0	2.14e-05	4.44e-06	0.000133%
**OTU_7207**	**True**	**ZB2**	1.83e-06	0.000167%	0.0	1	8.43e-05	1.73e-06	1.68e-06	3.96e-06	0.000119%
**L1707**	**False**	**Mb-NB09**	1.83e-06	0.000167%	0.0	1	0.000848	6.97e-05	3.59e-05	3.04e-06	9.13e-05%
**L1118**	**False**	**koll11**	1.83e-06	0.000167%	0.0	1	0.00347	0.000292	0.000849	4.1e-06	0.000123%
**OTU_10850**	**True**	**OP11-4**	1.83e-06	0.000166%	0.0	1	9.67e-05	0.0	0.0	3.29e-06	9.87e-05%
**L280**	**False**	**ABY1**	1.82e-06	0.000166%	0.0	1	0.000339	2.02e-05	0.0	3.55e-06	0.000107%
**OTU_904**	**True**	**koll11**	1.82e-06	0.000166%	0.0	1	0.000371	7.9e-06	0.0	3.01e-06	9.04e-05%
**OTU_7938**	**True**	**ABY1**	1.82e-06	0.000165%	0.0	1	0.00024	1.77e-06	2.25e-05	3.87e-06	0.000116%
**OTU_10809**	**True**	**OP11-4**	1.82e-06	0.000165%	0.0	1	0.000247	3.82e-05	0.0	3.82e-06	0.000115%
**OTU_2412**	**True**	**SJA-4**	1.82e-06	0.000165%	0.0	1	0.000143	6.91e-06	0.0	4.32e-06	0.00013%
**OTU_8172**	**True**	**EW055**	1.81e-06	0.000165%	0.0	1	3.64e-06	0.0	0.000373	4.96e-06	0.000149%
**OTU_6976**	**True**	**ZB2**	1.81e-06	0.000165%	0.0	1	0.000258	2.17e-05	1.91e-05	6.68e-06	0.0002%
**L1168**	**False**	**GIF10**	1.81e-06	0.000165%	0.0	1	0.00107	0.000803	3.73e-05	7.43e-06	0.000223%
**OTU_6953**	**True**	**ZB2**	1.8e-06	0.000164%	0.0	1	0.0	0.000331	0.0	2.72e-06	8.17e-05%
**L515**	**False**	**OP11-3**	1.8e-06	0.000164%	0.0	1	0.000692	0.000407	5.68e-05	1.25e-05	0.000376%
**OTU_6680**	**True**	**OD1**	1.8e-06	0.000164%	0.0	1	0.00022	0.0	1.29e-05	2.84e-06	8.51e-05%
**OTU_2194**	**True**	**koll11**	1.8e-06	0.000164%	0.0	1	0.00102	1.04e-05	0.000807	6.9e-06	0.000207%
**L34**	**False**	**ABY1**	1.8e-06	0.000164%	0.0	1	0.000354	1.52e-05	1.5e-05	3.13e-06	9.38e-05%
**L1880**	**False**	**OD1**	1.8e-06	0.000164%	0.0	1	0.000266	3.77e-05	1.32e-05	4.41e-06	0.000132%
**OTU_8239**	**True**	**ABY1**	1.79e-06	0.000163%	0.0	1	0.000195	1.3e-06	0.0	3.5e-06	0.000105%
**OTU_2529**	**True**	**GIF10**	1.77e-06	0.000162%	0.0	1	0.000434	2.01e-05	1.11e-05	3.99e-06	0.00012%
**L1200**	**False**	**koll11**	1.77e-06	0.000161%	0.0	1	0.00149	0.000807	0.000103	7.64e-06	0.000229%
**OTU_892**	**True**	**BD4-9**	1.76e-06	0.000161%	0.0	1	0.000251	0.0	0.0	4.29e-06	0.000129%
**L1172**	**False**	**GIF10**	1.76e-06	0.00016%	0.0	1	0.000926	0.000293	3.73e-05	4.7e-06	0.000141%
**OTU_7929**	**True**	**OD1**	1.76e-06	0.00016%	0.0	1	8.22e-05	0.0	6.52e-07	3.67e-06	0.00011%
**OTU_672**	**True**	**koll11**	1.76e-06	0.00016%	0.0	1	0.000709	0.000292	0.000149	1.39e-05	0.000417%
**L1773**	**False**	**OD1**	1.76e-06	0.00016%	0.0	1	0.000573	0.000285	5.7e-05	8.05e-06	0.000242%
**OTU_8267**	**True**	**ABY1**	1.75e-06	0.000159%	0.0	1	7.59e-06	0.00236	0.00209	5.46e-06	0.000164%
**OTU_8201**	**True**	**ABY1**	1.75e-06	0.000159%	0.0	1	0.000125	2.63e-05	5.83e-08	4.46e-06	0.000134%
**L944**	**False**	**koll11**	1.75e-06	0.000159%	0.0	1	0.00421	0.00108	0.000843	3.11e-06	9.33e-05%
**OTU_7219**	**True**	**ZB2**	1.74e-06	0.000159%	0.0	1	3.3e-05	0.000269	0.0	5.98e-06	0.00018%
**OTU_293**	**True**	**koll11**	1.74e-06	0.000159%	0.0	1	0.000396	0.000168	9.72e-05	1.91e-05	0.000573%
**OTU_4010**	**True**	**koll11**	1.73e-06	0.000158%	0.0	1	0.000467	0.000332	5.77e-06	6.7e-06	0.000201%
**L1354**	**False**	**ZB2**	1.73e-06	0.000157%	0.0	1	0.00277	0.00119	0.000925	1.56e-05	0.000468%
**OTU_6069**	**True**	**ABY1**	1.72e-06	0.000156%	0.0	1	0.000275	3.27e-05	2.8e-05	5.3e-06	0.000159%
**OTU_821**	**True**	**koll11**	1.72e-06	0.000156%	0.0	1	0.000159	0.0	0.0	3.62e-06	0.000109%
**L1341**	**False**	**ZB2**	1.72e-06	0.000156%	0.0	1	0.00061	0.000194	1.49e-05	5.23e-06	0.000157%
**OTU_817**	**True**	**BD4-9**	1.72e-06	0.000156%	0.0	1	0.000102	0.0	0.0	3.1e-06	9.32e-05%
**OTU_559**	**True**	**koll11**	1.7e-06	0.000155%	0.0	1	0.00029	1.26e-05	2.71e-05	4.26e-06	0.000128%
**OTU_345**	**True**	**koll11**	1.69e-06	0.000154%	0.0	1	3.12e-05	4.72e-05	0.000358	1.13e-05	0.000338%
**OTU_8006**	**True**	**TM7-1**	1.69e-06	0.000154%	0.0	1	0.000309	3.49e-05	0.0	4.47e-06	0.000134%
**L1623**	**False**	**OD1**	1.69e-06	0.000154%	0.0	1	0.00026	0.00018	4.84e-05	1.58e-05	0.000474%
**OTU_1539**	**True**	**PBS-25**	1.68e-06	0.000153%	0.0	1	0.00012	0.0	0.0	3.54e-06	0.000106%
**OTU_2714**	**True**	**koll11**	1.68e-06	0.000153%	0.0	1	0.00102	0.000191	0.000647	1.23e-05	0.00037%

**OTU_6183**	**True**	**WCHB1**	1.68e-06	0.000153%	0.0	1	0.000643	0.000351	0.000768	5.69e-05	0.00171%
**L225**	**False**	**ABY1**	1.68e-06	0.000153%	0.0	1	0.00105	0.000715	0.000458	4.13e-05	0.00124%
**OTU_6569**	**True**	**ZB2**	1.67e-06	0.000152%	0.0	1	0.000284	2.37e-05	1.29e-05	3.88e-06	0.000117%
**OTU_8050**	**True**	**ABY1**	1.67e-06	0.000152%	0.0	1	0.000138	3.5e-05	1.42e-07	5.17e-06	0.000155%
**L911**	**False**	**koll11**	1.66e-06	0.000151%	0.0	1	0.000862	0.000289	0.000276	1.08e-05	0.000325%
**L202**	**False**	**ABY1**	1.66e-06	0.000151%	0.0	1	0.00247	0.000323	0.000606	4.5e-06	0.000135%
**L1570**	**False**	**ZB2**	1.65e-06	0.00015%	0.0	1	0.00168	0.00033	0.00048	5.16e-06	0.000155%
**L761**	**False**	**PBS-25**	1.64e-06	0.000149%	0.0	1	0.00213	0.00025	0.000115	6.16e-06	0.000185%
**OTU_8219**	**True**	**ABY1**	1.64e-06	0.000149%	0.0	1	0.000118	7.44e-06	1.02e-05	5.69e-06	0.000171%
**OTU_2577**	**True**	**PBS-25**	1.63e-06	0.000148%	0.0	1	0.000715	7.86e-05	2.68e-05	4.59e-06	0.000138%
**OTU_9137**	**True**	**ABY1**	1.63e-06	0.000148%	0.0	1	0.0	0.000409	0.0	5.13e-06	0.000154%
**OTU_10770**	**True**	**OP11-4**	1.62e-06	0.000148%	0.0	1	0.000211	6.89e-05	2.4e-06	4.74e-06	0.000142%
**OTU_509**	**True**	**PBS-25**	1.62e-06	0.000147%	0.0	1	0.000976	7.84e-05	4.2e-05	4.35e-06	0.00013%
**OTU_8227**	**True**	**OD1**	1.62e-06	0.000147%	0.0	1	0.000101	5.67e-06	1.2e-05	4.85e-06	0.000145%
**L1170**	**False**	**GIF10**	1.62e-06	0.000147%	0.0	1	0.000927	0.000803	3.73e-05	7.37e-06	0.000221%
**L237**	**False**	**ABY1**	1.61e-06	0.000146%	0.0	1	0.00129	0.000118	2.5e-05	2.98e-06	8.94e-05%
**OTU_367**	**True**	**koll11**	1.6e-06	0.000146%	0.0	1	0.000442	3.78e-05	0.000149	6.19e-06	0.000186%
**OTU_445**	**True**	**koll11**	1.6e-06	0.000145%	0.0	1	0.000373	7.14e-05	5.6e-05	5.89e-06	0.000177%
**L1303**	**False**	**TM7**	1.59e-06	0.000145%	0.0	1	0.000674	0.000218	0.000139	9.81e-06	0.000294%
**OTU_4122**	**True**	**koll11**	1.59e-06	0.000144%	0.0	1	0.000706	0.000793	0.000128	1.92e-05	0.000577%
**L665**	**False**	**koll11**	1.58e-06	0.000144%	0.0	1	0.00261	0.000106	0.000278	2.67e-06	8.01e-05%
**OTU_9341**	**True**	**TM7-1**	1.57e-06	0.000143%	0.0	1	0.000192	3.44e-06	2.9e-05	4.12e-06	0.000124%
**OTU_2279**	**True**	**OP3**	1.57e-06	0.000143%	0.0	1	0.00107	3.14e-05	0.0	2.14e-06	6.44e-05%
**OTU_6219**	**True**	**ZB2**	1.57e-06	0.000143%	0.0	1	0.000254	1.89e-05	0.0	4.2e-06	0.000126%
**L1651**	**False**	**ZB2**	1.56e-06	0.000142%	0.0	1	0.000152	6.7e-05	0.000643	9.11e-06	0.000274%
**L739**	**False**	**PBS-25**	1.56e-06	0.000142%	0.0	1	0.00215	0.000181	0.000977	7.48e-06	0.000224%
**OTU_3356**	**True**	**SBRH58**	1.56e-06	0.000142%	0.0	1	0.0003	0.0	5.83e-08	2.55e-06	7.67e-05%
**OTU_8749**	**True**	**OD1**	1.56e-06	0.000142%	0.0	1	0.00037	0.000264	3.57e-05	9.1e-06	0.000273%
**OTU_3027**	**True**	**SJA-4**	1.56e-06	0.000142%	0.0	1	2.96e-05	0.0	0.000222	7.4e-06	0.000222%
**OTU_468**	**True**	**koll11**	1.54e-06	0.000141%	0.0	1	4.74e-05	0.0002	9.23e-06	1.06e-05	0.000318%
**OTU_140**	**True**	**OD1**	1.54e-06	0.00014%	0.0	1	3.83e-05	0.000211	0.000291	3.06e-05	0.000918%
**OTU_8990**	**True**	**ABY1**	1.54e-06	0.00014%	0.0	1	0.0	0.000266	0.000132	8.35e-06	0.000251%
**OTU_2202**	**True**	**PBS-25**	1.54e-06	0.00014%	0.0	1	0.000189	0.0	0.0	3.66e-06	0.00011%
**OTU_278**	**True**	**koll11**	1.54e-06	0.00014%	0.0	1	0.000722	0.000235	8.63e-05	7.02e-06	0.000211%
**OTU_860**	**True**	**PBS-25**	1.53e-06	0.000139%	0.0	1	0.000186	0.0	0.0	4.37e-06	0.000131%
**OTU_1432**	**True**	**koll11**	1.53e-06	0.000139%	0.0	1	0.000414	4.04e-06	0.0	1.75e-06	5.26e-05%
**OTU_574**	**True**	**koll11**	1.52e-06	0.000139%	0.0	1	0.000217	1.35e-06	3.02e-05	4.12e-06	0.000124%
**L1875**	**False**	**OD1**	1.5e-06	0.000136%	0.0	1	0.000817	0.000445	3.98e-05	5.47e-06	0.000164%
**L445**	**False**	**OP11-4**	1.5e-06	0.000136%	0.0	1	0.00119	0.000126	0.000843	8.38e-06	0.000252%
**OTU_9374**	**True**	**TM7-1**	1.5e-06	0.000136%	0.0	1	0.000249	0.0	0.0	2.84e-06	8.54e-05%
**L696**	**False**	**koll11**	1.48e-06	0.000135%	0.0	1	0.00165	0.000863	4.44e-05	3.5e-06	0.000105%
**L671**	**False**	**koll11**	1.48e-06	0.000134%	0.0	1	0.000817	7.27e-05	2.55e-05	3.12e-06	9.37e-05%
**OTU_6189**	**True**	**ZB2**	1.48e-06	0.000134%	0.0	1	0.0	0.000321	6.86e-05	3.17e-06	9.5e-05%
**OTU_2009**	**True**	**PRR-12**	1.47e-06	0.000134%	0.0	1	0.00118	0.000115	0.000168	3.05e-06	9.16e-05%
**L245**	**False**	**ABY1**	1.47e-06	0.000134%	0.0	1	8.94e-05	9.81e-05	0.000806	7.83e-06	0.000235%
**OTU_1389**	**True**	**GIF10**	1.47e-06	0.000133%	0.0	1	0.000891	0.00109	0.00196	7.44e-05	0.00223%
**L1114**	**False**	**koll11**	1.46e-06	0.000133%	0.0	1	0.00342	0.000356	0.0015	5.39e-06	0.000162%
**OTU_8936**	**True**	**Mb-NB09**	1.46e-06	0.000133%	0.0	1	0.0	0.000157	9.73e-06	4.34e-06	0.00013%
**OTU_554**	**True**	**PRR-12**	1.46e-06	0.000133%	0.0	1	0.00038	0.0	2.96e-06	2.99e-06	8.98e-05%
**L471**	**False**	**OP11-4**	1.45e-06	0.000132%	0.0	1	0.000174	2.4e-06	4.83e-06	2.63e-06	7.89e-05%
**L1877**	**False**	**OD1**	1.44e-06	0.000131%	0.0	1	0.000648	0.000329	3.18e-05	5.27e-06	0.000158%
**L214**	**False**	**ABY1**	1.44e-06	0.000131%	0.0	1	0.00022	0.000249	0.0	8.03e-06	0.000241%
**L178**	**False**	**SM2F11**	1.44e-06	0.000131%	0.0	1	0.000499	0.000227	0.000185	1.4e-05	0.000421%
**L1202**	**False**	**koll11**	1.43e-06	0.00013%	0.0	1	0.000961	0.000394	7.3e-05	6.2e-06	0.000186%
**L1508**	**False**	**OD1**	1.41e-06	0.000128%	0.0	1	0.0134	0.00343	0.00626	3.6e-06	0.000108%
**OTU_5075**	**True**	**GIF10**	1.4e-06	0.000128%	0.0	1	0.000104	0.0	0.0	2.27e-06	6.8e-05%
**OTU_8213**	**True**	**ABY1**	1.4e-06	0.000127%	0.0	1	0.000122	7.4e-07	0.0	2.15e-06	6.44e-05%
**OTU_981**	**True**	**PRR-12**	1.4e-06	0.000127%	0.0	1	0.000651	7.56e-07	0.0	2.87e-06	8.62e-05%
**OTU_5837**	**True**	**OD1**	1.39e-06	0.000127%	0.0	1	0.000154	5.95e-06	3.64e-05	5.49e-06	0.000165%
**OTU_3769**	**True**	**koll11**	1.39e-06	0.000127%	0.0	1	0.00119	0.000531	3.86e-05	4.1e-06	0.000123%
**OTU_7211**	**True**	**ABY1**	1.38e-06	0.000126%	0.0	1	7.01e-05	0.0	0.0	3.28e-06	9.85e-05%
**L1398**	**False**	**ZB2**	1.38e-06	0.000126%	0.0	1	0.00152	0.00208	0.000834	3.09e-05	0.000927%
**OTU_8021**	**True**	**ABY1**	1.38e-06	0.000125%	0.0	1	0.000546	0.000116	2.51e-06	4.3e-06	0.000129%
**OTU_6995**	**True**	**ABY1**	1.38e-06	0.000125%	0.0	1	0.000861	0.000256	7.46e-05	3.45e-06	0.000103%
**OTU_7959**	**True**	**ABY1**	1.37e-06	0.000125%	0.0	1	0.000492	0.00024	0.0	6.01e-06	0.00018%

**L1266**	**False**	**TM7-1**	1.37e-06	0.000125%	0.0	1	0.00117	0.000594	5.57e-05	5.07e-06	0.000152%
**OTU_454**	**True**	**koll11**	1.37e-06	0.000125%	0.0	1	0.000415	3.42e-05	2.11e-05	2.57e-06	7.71e-05%
**OTU_1232**	**True**	**PBS-25**	1.36e-06	0.000124%	0.0	1	0.00185	6.75e-05	7.1e-05	3.16e-06	9.48e-05%
**OTU_498**	**True**	**wb1_H11**	1.36e-06	0.000124%	0.0	1	0.000303	0.0	0.0	2.51e-06	7.54e-05%
**OTU_384**	**True**	**PBS-25**	1.36e-06	0.000123%	0.0	1	0.00015	0.000262	5.83e-08	6.68e-06	0.0002%
**OTU_440**	**True**	**PBS-25**	1.35e-06	0.000123%	0.0	1	0.000687	7.95e-05	2.89e-05	2.62e-06	7.88e-05%
**OTU_9815**	**True**	**ABY1**	1.35e-06	0.000123%	0.0	1	0.000101	0.0	0.0	2.68e-06	8.05e-05%
**OTU_6708**	**True**	**ZB2**	1.35e-06	0.000122%	0.0	1	9.12e-05	0.0	0.0	2.43e-06	7.3e-05%
**OTU_8252**	**True**	**ABY1**	1.33e-06	0.000121%	0.0	1	0.00109	3.87e-05	4.66e-06	1.91e-06	5.74e-05%
**OTU_7958**	**True**	**ABY1**	1.32e-06	0.00012%	0.0	1	9.96e-05	7.49e-06	0.0	3.39e-06	0.000102%
**L1469**	**False**	**ZB2**	1.31e-06	0.00012%	0.0	1	0.000406	0.000224	1.24e-05	5.69e-06	0.000171%
**L688**	**False**	**koll11**	1.3e-06	0.000119%	0.0	1	0.00417	0.00138	0.00116	4.12e-06	0.000124%
**OTU_7943**	**True**	**ABY1**	1.3e-06	0.000118%	0.0	1	8.59e-05	0.0	0.0	2.99e-06	8.97e-05%
**OTU_501**	**True**	**koll11**	1.29e-06	0.000118%	0.0	1	0.000209	0.0	0.0	1.86e-06	5.58e-05%
**L451**	**False**	**OP11-4**	1.29e-06	0.000118%	0.0	1	0.001	0.000103	0.000609	8.7e-06	0.000261%
**OTU_7029**	**True**	**OD1**	1.29e-06	0.000117%	0.0	1	0.000188	0.000196	5.77e-05	1.82e-05	0.000547%
**OTU_659**	**True**	**koll11**	1.28e-06	0.000117%	0.0	1	0.000124	0.0	0.0	2.33e-06	7e-05%
**OTU_3274**	**True**	**PBS-25**	1.28e-06	0.000117%	0.0	1	0.000276	1.85e-05	0.0	2.72e-06	8.16e-05%
**L48**	**False**	**OD1**	1.28e-06	0.000116%	0.0	1	0.000423	0.000318	4.6e-06	6.51e-06	0.000195%
**L1899**	**False**	**OD1**	1.27e-06	0.000116%	0.0	1	0.000752	0.000337	8.89e-05	5.89e-06	0.000177%
**OTU_562**	**True**	**koll11**	1.27e-06	0.000116%	0.0	1	0.000338	2.19e-05	4.75e-05	3.23e-06	9.68e-05%
**OTU_6515**	**True**	**SM2F11**	1.26e-06	0.000114%	0.0	1	0.000229	0.0	2.53e-05	3.21e-06	9.65e-05%
**OTU_1042**	**True**	**OP3**	1.25e-06	0.000114%	0.0	1	0.00027	3.52e-05	4.48e-06	2.28e-06	6.83e-05%
**OTU_2685**	**True**	**PBS-25**	1.25e-06	0.000114%	0.0	1	0.0	0.0	0.000288	3.29e-06	9.87e-05%
**OTU_5377**	**True**	**ZB2**	1.25e-06	0.000114%	0.0	1	0.000187	1.69e-05	4.58e-05	6.2e-06	0.000186%
**OTU_10819**	**True**	**OP11-4**	1.25e-06	0.000114%	0.0	1	0.0	5.74e-05	0.0	4.58e-06	0.000137%
**L517**	**False**	**OP11-3**	1.25e-06	0.000114%	0.0	1	0.00028	0.0004	5.63e-05	1.39e-05	0.000418%
**OTU_6305**	**True**	**OD1**	1.24e-06	0.000113%	0.0	1	5.3e-05	0.0	0.0	2.94e-06	8.82e-05%
**L546**	**False**	**SJA-4**	1.24e-06	0.000113%	0.0	1	0.000377	0.000181	0.000791	1.81e-05	0.000543%
**OTU_6184**	**True**	**SM2F11**	1.24e-06	0.000112%	0.0	1	0.0	0.000167	1.18e-05	3.56e-06	0.000107%
**OTU_8757**	**True**	**OD1**	1.23e-06	0.000112%	0.0	1	0.000109	0.0	6.45e-05	5.34e-06	0.00016%
**L382**	**False**	**OD1**	1.22e-06	0.000111%	0.0	1	0.000769	3.53e-05	7.74e-05	2.87e-06	8.6e-05%
**OTU_5491**	**True**	**OD1**	1.22e-06	0.000111%	0.0	1	0.000379	0.0	0.0	2.13e-06	6.39e-05%
**L1487**	**False**	**ZB2**	1.21e-06	0.000111%	0.0	1	0.00188	0.000763	0.000476	8.14e-06	0.000244%
**OTU_6930**	**True**	**ZB2**	1.21e-06	0.00011%	0.0	1	0.000145	0.0	0.0	2.54e-06	7.63e-05%
**OTU_295**	**True**	**koll11**	1.21e-06	0.00011%	0.0	1	1.99e-05	0.000172	0.0	5.09e-06	0.000153%
**OTU_985**	**True**	**PBS-25**	1.2e-06	0.000109%	0.0	1	0.000444	0.000133	5.83e-08	3.55e-06	0.000106%
**OTU_550**	**True**	**koll11**	1.19e-06	0.000108%	0.0	1	0.000198	0.0	4.47e-06	3.01e-06	9.02e-05%
**OTU_1244**	**True**	**koll11**	1.19e-06	0.000108%	0.0	1	0.000536	0.000691	0.000171	1.83e-05	0.00055%
**OTU_10934**	**True**	**GIF10**	1.18e-06	0.000108%	0.0	1	0.000138	0.0	0.0	2.17e-06	6.51e-05%
**OTU_5392**	**True**	**ZB2**	1.18e-06	0.000107%	0.0	1	5.19e-05	0.0	5.42e-06	3.57e-06	0.000107%
**OTU_7215**	**True**	**ABY1**	1.17e-06	0.000106%	0.0	1	0.000506	0.000115	1.93e-05	2.86e-06	8.59e-05%
**OTU_1825**	**True**	**koll11**	1.17e-06	0.000106%	0.0	1	0.000647	0.000268	0.000276	1.37e-05	0.000412%
**OTU_8373**	**True**	**ABY1**	1.16e-06	0.000106%	0.0	1	0.000129	1.77e-07	1.37e-05	2.58e-06	7.73e-05%
**OTU_1140**	**True**	**PBS-25**	1.16e-06	0.000106%	0.0	1	0.000383	4.73e-05	0.000234	1.01e-05	0.000303%
**L571**	**False**	**SJA-4**	1.16e-06	0.000105%	0.0	1	0.000143	0.000193	0.0	7.02e-06	0.000211%
**OTU_4802**	**True**	**GIF10**	1.15e-06	0.000104%	0.0	1	0.000195	3.05e-05	0.0	3.61e-06	0.000108%
**L1639**	**False**	**ZB2**	1.14e-06	0.000104%	0.0	1	0.00101	0.00211	0.00281	3.53e-05	0.00106%
**OTU_6222**	**True**	**ZB2**	1.14e-06	0.000104%	0.0	1	0.000208	6.11e-05	4.3e-05	6.39e-06	0.000192%
**L1359**	**False**	**ZB2**	1.13e-06	0.000103%	0.0	1	0.000251	0.0	0.000287	6.13e-06	0.000184%
**OTU_6933**	**True**	**ZB2**	1.13e-06	0.000103%	0.0	1	0.000233	4.77e-05	0.0	3.46e-06	0.000104%
**OTU_4247**	**True**	**koll11**	1.13e-06	0.000103%	0.0	1	0.000422	8.14e-06	2.42e-05	2.52e-06	7.55e-05%
**OTU_1218**	**True**	**koll11**	1.12e-06	0.000102%	0.0	1	0.00023	8.73e-05	2.06e-05	4.92e-06	0.000148%
**OTU_8126**	**True**	**ABY1**	1.12e-06	0.000102%	0.0	1	0.000772	8.39e-07	2.36e-05	2.95e-06	8.84e-05%
**L1646**	**False**	**ZB2**	1.12e-06	0.000102%	0.0	1	0.000557	0.000172	0.000172	7.57e-06	0.000227%
**OTU_8208**	**True**	**ABY1**	1.12e-06	0.000102%	0.0	1	6.49e-05	5.77e-05	6.78e-06	1.15e-05	0.000346%
**OTU_7930**	**True**	**ZB2**	1.12e-06	0.000102%	0.0	1	0.000115	0.0	0.0	2.21e-06	6.64e-05%
**OTU_8247**	**True**	**ZB2**	1.11e-06	0.000101%	0.0	1	0.000354	0.000315	2.34e-06	7e-06	0.00021%
**OTU_5887**	**True**	**OD1**	1.1e-06	0.0001%	0.0	1	0.000266	1.97e-05	3.14e-06	2.01e-06	6.03e-05%
**L693**	**False**	**koll11**	1.1e-06	0.0001%	0.0	1	0.00139	0.000278	0.000471	4.43e-06	0.000133%
**L1645**	**False**	**ZB2**	1.09e-06	9.97e-05%	0.0	1	0.000709	0.000239	0.000815	1.22e-05	0.000366%
**OTU_4833**	**True**	**OD1**	1.09e-06	9.91e-05%	0.0	1	6.43e-05	0.0	5.83e-08	2.51e-06	7.54e-05%
**L629**	**False**	**PBS-25**	1.08e-06	9.86e-05%	0.0	1	0.00269	8.75e-05	7.1e-05	1.85e-06	5.55e-05%
**L429**	**False**	**OP11**	1.08e-06	9.84e-05%	0.0	1	0.00084	0.000351	0.000768	2.39e-05	0.000717%

**OTU_8762**	**True**	**OD1**	1.07e-06	9.77e-05%	0.0	1	0.000203	2.06e-05	2.13e-05	2.91e-06	8.74e-05%
**OTU_3804**	**True**	**PBS-25**	1.07e-06	9.73e-05%	0.0	1	0.000108	0.0	0.0	2.24e-06	6.72e-05%
**OTU_643**	**True**	**koll11**	1.07e-06	9.72e-05%	0.0	1	0.00118	0.000226	3.02e-05	3.22e-06	9.66e-05%
**OTU_5794**	**True**	**OD1**	1.07e-06	9.7e-05%	0.0	1	0.000261	1.94e-05	2.62e-05	1.89e-06	5.67e-05%
**OTU_6117**	**True**	**OD1**	1.06e-06	9.64e-05%	0.0	1	0.000307	4.44e-05	0.000305	9.76e-06	0.000293%
**OTU_1133**	**True**	**GIF10**	1.06e-06	9.63e-05%	0.0	1	0.000492	0.000272	2.62e-05	4.84e-06	0.000145%
**OTU_8009**	**True**	**ABY1**	1.06e-06	9.62e-05%	0.0	1	0.000673	0.0	0.0	2.2e-06	6.6e-05%
**L302**	**False**	**ABY1**	1.05e-06	9.6e-05%	0.0	1	0.000299	1.08e-05	5.5e-06	1.97e-06	5.91e-05%
**OTU_6758**	**True**	**ZB2**	1.05e-06	9.57e-05%	0.0	1	0.000183	7.15e-07	0.0	2.76e-06	8.28e-05%
**OTU_7059**	**True**	**ZB2**	1.05e-06	9.54e-05%	0.0	1	6.19e-05	0.000532	5.45e-06	3.3e-06	9.91e-05%
**OTU_5878**	**True**	**OD1**	1.05e-06	9.52e-05%	0.0	1	0.000151	5.03e-05	1.32e-05	4.78e-06	0.000143%
**OTU_8903**	**True**	**ABY1**	1.05e-06	9.52e-05%	0.0	1	0.000412	0.0	8.1e-05	3.23e-06	9.7e-05%
**OTU_2868**	**True**	**SJA-4**	1.04e-06	9.51e-05%	0.0	1	0.0	0.000215	1.05e-06	3.33e-06	9.99e-05%
**OTU_6579**	**True**	**ZB2**	1.04e-06	9.47e-05%	0.0	1	1.58e-06	0.0001	0.000417	5.16e-06	0.000155%
**L1578**	**False**	**ZB2**	1.04e-06	9.42e-05%	0.0	1	0.000445	2.46e-05	5.46e-05	2.86e-06	8.58e-05%
**OTU_580**	**True**	**PBS-25**	1.03e-06	9.38e-05%	0.0	1	0.00125	0.000219	0.000127	2.65e-06	7.94e-05%
**L1154**	**False**	**koll11**	1.03e-06	9.35e-05%	0.0	1	0.00615	0.00134	0.00466	7.49e-06	0.000225%
**OTU_4482**	**True**	**koll11**	1.02e-06	9.3e-05%	0.0	1	0.00015	0.0	0.0	2.41e-06	7.23e-05%
**L615**	**False**	**BD4-9**	1.02e-06	9.26e-05%	0.0	1	0.000353	0.000132	0.0	4.3e-06	0.000129%
**OTU_893**	**True**	**PBS-25**	1.01e-06	9.2e-05%	0.0	1	0.000499	0.000218	5.83e-08	4.14e-06	0.000124%
**L728**	**False**	**PBS-25**	1.01e-06	9.2e-05%	0.0	1	0.00722	0.00103	0.00263	3.73e-06	0.000112%
**OTU_10835**	**True**	**OP11-4**	1.01e-06	9.18e-05%	0.0	1	6.03e-05	2.22e-06	4.83e-06	3.83e-06	0.000115%
**L562**	**False**	**SJA-4**	1.01e-06	9.16e-05%	0.0	1	0.000248	0.000158	2.35e-07	3.67e-06	0.00011%
**OTU_6561**	**True**	**OD1**	1e-06	9.13e-05%	0.0	1	9.09e-05	1.77e-07	0.0	2.01e-06	6.03e-05%
**OTU_500**	**True**	**PBS-25**	1e-06	9.11e-05%	0.0	1	0.000141	0.0	4.22e-06	2.18e-06	6.55e-05%
**OTU_6989**	**True**	**ABY1**	9.87e-07	8.99e-05%	0.0	1	8.59e-05	4.87e-05	1.2e-05	7.62e-06	0.000229%
**OTU_966**	**True**	**PRR-12**	9.69e-07	8.82e-05%	0.0	1	0.0	3.54e-05	9.86e-05	6.09e-06	0.000183%
**L1577**	**False**	**ZB2**	9.68e-07	8.81e-05%	0.0	1	0.000778	0.000332	7.69e-05	3.78e-06	0.000114%
**OTU_1117**	**True**	**BD4-9**	9.65e-07	8.79e-05%	0.0	1	0.0	0.000132	0.0	3.12e-06	9.38e-05%
**OTU_10774**	**True**	**OP11-4**	9.6e-07	8.74e-05%	0.0	1	0.00013	0.0	0.0	2.01e-06	6.03e-05%
**L1159**	**False**	**koll11**	9.56e-07	8.7e-05%	0.0	1	0.00268	1.25e-05	1.93e-05	1.96e-06	5.88e-05%
**OTU_266**	**True**	**koll11**	9.5e-07	8.65e-05%	0.0	1	0.000293	5.42e-06	1.77e-05	2.04e-06	6.12e-05%
**OTU_7041**	**True**	**ZB2**	9.46e-07	8.61e-05%	0.0	1	1.29e-06	0.000387	0.000302	4.05e-06	0.000121%
**OTU_6312**	**True**	**ZB2**	9.45e-07	8.6e-05%	0.0	1	0.00103	0.000135	7.25e-05	2.34e-06	7.02e-05%
**OTU_4223**	**True**	**koll11**	9.44e-07	8.59e-05%	0.0	1	0.0	0.0	0.000102	3.1e-06	9.31e-05%
**L691**	**False**	**koll11**	9.39e-07	8.55e-05%	0.0	1	0.00241	0.000469	0.00112	3.44e-06	0.000103%
**L1757**	**False**	**OD1**	9.36e-07	8.52e-05%	0.0	1	0.000465	0.00055	0.000214	3.25e-05	0.000974%
**OTU_8150**	**True**	**OP11-4**	9.36e-07	8.52e-05%	0.0	1	9.44e-05	0.0	7.21e-06	2.61e-06	7.84e-05%
**L817**	**False**	**PBS-25**	9.33e-07	8.5e-05%	0.0	1	0.00112	0.00028	5.83e-08	2.86e-06	8.59e-05%
**L316**	**False**	**ABY1**	9.33e-07	8.49e-05%	0.0	1	0.000161	0.000632	0.000213	9.71e-06	0.000292%
**OTU_8644**	**True**	**ABY1**	9.3e-07	8.46e-05%	0.0	1	0.000432	0.000248	1.78e-05	3.52e-06	0.000106%
**OTU_5265**	**True**	**OD1**	9.26e-07	8.43e-05%	0.0	1	0.000552	0.00014	1.58e-05	2.45e-06	7.35e-05%
**OTU_738**	**True**	**koll11**	9.26e-07	8.43e-05%	0.0	1	0.000147	4.73e-05	2.83e-06	4.46e-06	0.000134%
**OTU_7113**	**True**	**ABY1**	9.2e-07	8.37e-05%	0.0	1	3.23e-05	0.000126	0.0	3.53e-06	0.000106%
**OTU_8032**	**True**	**ABY1**	9.2e-07	8.37e-05%	0.0	1	0.000199	1.14e-05	0.0	2.47e-06	7.4e-05%
**L882**	**False**	**koll11**	9.12e-07	8.3e-05%	0.0	1	0.00711	0.00268	0.00229	3.34e-06	0.0001%
**L453**	**False**	**OP11-4**	9.11e-07	8.29e-05%	0.0	1	0.00097	3.92e-05	0.000597	4.92e-06	0.000148%
**OTU_5804**	**True**	**OD1**	9.08e-07	8.26e-05%	0.0	1	0.000385	1.28e-05	2.26e-06	2.25e-06	6.74e-05%
**OTU_388**	**True**	**koll11**	9.04e-07	8.23e-05%	0.0	1	0.000269	4.3e-05	0.0	1.71e-06	5.13e-05%
**OTU_306**	**True**	**koll11**	9.02e-07	8.21e-05%	0.0	1	0.000926	0.000762	0.000222	8.58e-06	0.000257%
**L446**	**False**	**OP11-4**	9.02e-07	8.21e-05%	0.0	1	0.000186	2.34e-05	0.000234	5.27e-06	0.000158%
**OTU_4170**	**True**	**koll11**	9e-07	8.19e-05%	0.0	1	2.61e-05	0.000123	0.0	4.51e-06	0.000135%
**OTU_5249**	**True**	**OD1**	8.99e-07	8.18e-05%	0.0	1	0.000361	7.91e-05	1.16e-05	2.57e-06	7.71e-05%
**L1395**	**False**	**ZB2**	8.96e-07	8.15e-05%	0.0	1	0.00236	0.000755	0.00088	7.97e-06	0.000239%
**OTU_433**	**True**	**PBS-25**	8.92e-07	8.12e-05%	0.0	1	0.000355	8.15e-05	7.91e-05	3.6e-06	0.000108%
**L683**	**False**	**koll11**	8.91e-07	8.11e-05%	0.0	1	0.000839	2.9e-05	0.000126	2.86e-06	8.58e-05%
**OTU_300**	**True**	**koll11**	8.88e-07	8.09e-05%	0.0	1	0.0	0.000107	0.0	2.57e-06	7.71e-05%
**OTU_6070**	**True**	**ZB2**	8.88e-07	8.08e-05%	0.0	1	1.09e-05	0.000568	0.0005	6.14e-06	0.000184%
**L1475**	**False**	**ZB2**	8.84e-07	8.04e-05%	0.0	1	0.000675	0.000493	0.000323	2.46e-05	0.000739%
**OTU_4019**	**True**	**koll11**	8.8e-07	8.01e-05%	0.0	1	0.0	0.0	0.000103	2.32e-06	6.96e-05%
**OTU_6988**	**True**	**ZB2**	8.79e-07	8e-05%	0.0	1	0.0015	0.000448	0.000737	8.53e-06	0.000256%
**OTU_1795**	**True**	**koll11**	8.75e-07	7.96e-05%	0.0	1	0.000253	3.13e-05	1.69e-05	2.7e-06	8.09e-05%
**L1868**	**False**	**OD1**	8.73e-07	7.95e-05%	0.0	1	0.00116	0.000116	0.000142	1.68e-06	5.03e-05%
**OTU_6180**	**True**	**OD1**	8.73e-07	7.95e-05%	0.0	1	0.000314	0.000319	2.8e-06	3.88e-06	0.000116%

**OTU_926**	**True**	**koll11**	8.72e-07	7.94e-05%	0.0	1	0.000882	0.000758	8.33e-05	4.51e-06	0.000135%
**OTU_4056**	**True**	**koll11**	8.67e-07	7.89e-05%	0.0	1	0.000418	2.09e-05	0.0	1.89e-06	5.68e-05%
**OTU_8324**	**True**	**ZB2**	8.64e-07	7.87e-05%	0.0	1	0.0	3.26e-06	6.84e-05	2.41e-06	7.23e-05%
**L880**	**False**	**koll11**	8.62e-07	7.85e-05%	0.0	1	0.00723	0.00275	0.00229	3.13e-06	9.4e-05%
**OTU_2694**	**True**	**SJA-4**	8.61e-07	7.84e-05%	0.0	1	0.0	0.0	0.00012	2.85e-06	8.56e-05%
**OTU_354**	**True**	**koll11**	8.57e-07	7.81e-05%	0.0	1	0.000312	0.0	1.3e-06	1.2e-06	3.62e-05%
**OTU_363**	**True**	**koll11**	8.56e-07	7.79e-05%	0.0	1	0.000317	0.000188	0.0	3.91e-06	0.000117%
**OTU_1608**	**True**	**koll11**	8.55e-07	7.79e-05%	0.0	1	0.000215	2.11e-05	0.0	2.1e-06	6.32e-05%
**OTU_3117**	**True**	**koll11**	8.53e-07	7.76e-05%	0.0	1	0.000262	6.54e-05	0.000139	8.16e-06	0.000245%
**OTU_827**	**True**	**koll11**	8.51e-07	7.75e-05%	0.0	1	0.000174	0.0	0.0	2.22e-06	6.65e-05%
**L44**	**False**	**OD1**	8.51e-07	7.75e-05%	0.0	1	0.104	0.137	0.0762	9.72e-06	0.000292%
**L1203**	**False**	**koll11**	8.5e-07	7.74e-05%	0.0	1	0.000461	0.000271	7.3e-05	6.6e-06	0.000198%
**L888**	**False**	**koll11**	8.48e-07	7.72e-05%	0.0	1	0.000579	0.000691	0.000171	1.21e-05	0.000362%
**OTU_5840**	**True**	**OD1**	8.46e-07	7.7e-05%	0.0	1	0.00017	0.000116	7.96e-06	5e-06	0.00015%
**OTU_965**	**True**	**PBS-25**	8.45e-07	7.69e-05%	0.0	1	0.0	3.2e-05	0.000115	4.19e-06	0.000126%
**OTU_1558**	**True**	**PBS-25**	8.45e-07	7.69e-05%	0.0	1	0.000118	2e-05	0.0	2.66e-06	7.99e-05%
**L1033**	**False**	**koll11**	8.43e-07	7.68e-05%	0.0	1	0.000434	2.27e-05	0.000747	5.14e-06	0.000154%
**OTU_4832**	**True**	**ZB2**	8.4e-07	7.65e-05%	0.0	1	0.000143	3.2e-06	0.0	1.17e-06	3.52e-05%
**L1465**	**False**	**ZB2**	8.39e-07	7.64e-05%	0.0	1	0.000254	0.00211	0.000806	5.81e-06	0.000174%
**OTU_464**	**True**	**koll11**	8.33e-07	7.58e-05%	0.0	1	4.58e-06	0.000113	0.0	2.68e-06	8.06e-05%
**OTU_365**	**True**	**koll11**	8.26e-07	7.51e-05%	0.0	1	0.000426	0.0	9.58e-05	2.92e-06	8.78e-05%
**OTU_9710**	**True**	**ABY1**	8.25e-07	7.51e-05%	0.0	1	0.000337	0.000105	4.81e-05	3.87e-06	0.000116%
**OTU_3007**	**True**	**koll11**	8.23e-07	7.49e-05%	0.0	1	0.000592	0.000591	8.98e-05	1.01e-05	0.000305%
**OTU_3762**	**True**	**SJA-4**	8.18e-07	7.45e-05%	0.0	1	0.000184	0.0	0.0	1.51e-06	4.52e-05%
**OTU_4166**	**True**	**koll11**	8.16e-07	7.43e-05%	0.0	1	4.66e-06	0.000275	0.0	2.57e-06	7.71e-05%
**OTU_510**	**True**	**koll11**	8.15e-07	7.42e-05%	0.0	1	0.000337	2.32e-05	1.96e-05	1.84e-06	5.53e-05%
**OTU_8143**	**True**	**OP11-4**	8.14e-07	7.41e-05%	0.0	1	8.52e-06	7.23e-06	8.56e-05	4.42e-06	0.000133%
**OTU_6005**	**True**	**SM2F11**	8.13e-07	7.4e-05%	0.0	1	0.000206	0.000183	2.38e-05	6.54e-06	0.000196%
**OTU_8125**	**True**	**ABY1**	8.12e-07	7.39e-05%	0.0	1	0.000508	0.000258	5.66e-06	2.5e-06	7.5e-05%
**L1881**	**False**	**OD1**	8.07e-07	7.35e-05%	0.0	1	0.000193	1.77e-07	0.0	1.25e-06	3.76e-05%
**OTU_7225**	**True**	**ZB2**	8.01e-07	7.29e-05%	0.0	1	0.00247	0.00102	0.00106	8.69e-06	0.000261%
**OTU_1444**	**True**	**koll11**	8e-07	7.28e-05%	0.0	1	0.000284	6.39e-06	0.000259	3.35e-06	0.000101%
**OTU_7949**	**True**	**ABY1**	8e-07	7.28e-05%	0.0	1	0.000654	0.000519	6.07e-05	5.18e-06	0.000155%
**L819**	**False**	**PBS-25**	7.96e-07	7.25e-05%	0.0	1	0.000427	0.00028	5.83e-08	2.87e-06	8.61e-05%
**OTU_10753**	**True**	**OP11-3**	7.96e-07	7.25e-05%	0.0	1	0.000161	6.14e-05	5.63e-05	9.88e-06	0.000297%
**OTU_675**	**True**	**PBS-25**	7.95e-07	7.24e-05%	0.0	1	0.000532	0.000219	0.00198	5.4e-06	0.000162%
**L1635**	**False**	**ZB2**	7.93e-07	7.22e-05%	0.0	1	0.000354	0.000398	2.34e-06	5.33e-06	0.00016%
**L1494**	**False**	**ZB2**	7.93e-07	7.22e-05%	0.0	1	0.00055	0.000107	1.69e-05	1.54e-06	4.62e-05%
**OTU_8743**	**True**	**OD1**	7.85e-07	7.15e-05%	0.0	1	0.000207	0.0	0.0	1.45e-06	4.35e-05%
**OTU_7980**	**True**	**OP11-4**	7.84e-07	7.14e-05%	0.0	1	0.000124	4.38e-05	0.000176	1.07e-05	0.000321%
**L1737**	**False**	**Mb-NB09**	7.84e-07	7.14e-05%	0.0	1	0.000281	7.56e-05	0.0	2.2e-06	6.61e-05%
**L1138**	**False**	**koll11**	7.83e-07	7.12e-05%	0.0	1	0.000398	5.6e-06	1.77e-05	1.26e-06	3.79e-05%
**OTU_8112**	**True**	**ABY1**	7.82e-07	7.12e-05%	0.0	1	0.00179	0.0007	9.49e-05	1.79e-06	5.37e-05%
**OTU_3602**	**True**	**PBS-25**	7.8e-07	7.1e-05%	0.0	1	0.000263	1.66e-05	2.77e-05	1.91e-06	5.74e-05%
**OTU_2636**	**True**	**koll11**	7.79e-07	7.09e-05%	0.0	1	8.96e-05	0.0	3.72e-05	2.96e-06	8.87e-05%
**OTU_10854**	**True**	**OP11-4**	7.77e-07	7.07e-05%	0.0	1	0.000153	0.0	0.0	1.85e-06	5.54e-05%
**OTU_152**	**True**	**ZB2**	7.76e-07	7.06e-05%	0.0	1	0.000191	5.68e-06	5.46e-05	3.14e-06	9.42e-05%
**OTU_7216**	**True**	**ZB2**	7.74e-07	7.04e-05%	0.0	1	0.000206	0.0	0.0	1.44e-06	4.31e-05%
**OTU_339**	**True**	**koll11**	7.7e-07	7.01e-05%	0.0	1	0.000593	7.39e-05	2.84e-05	3.12e-06	9.35e-05%
**OTU_6585**	**True**	**ZB2**	7.69e-07	7e-05%	0.0	1	0.000152	4.54e-05	0.000119	1.13e-05	0.000338%
**L22**	**False**	**OD1**	7.67e-07	6.99e-05%	0.0	1	0.000222	0.000138	1.2e-05	3.4e-06	0.000102%
**OTU_427**	**True**	**koll11**	7.67e-07	6.98e-05%	0.0	1	8.28e-05	0.000351	0.0	3.43e-06	0.000103%
**L560**	**False**	**SJA-4**	7.52e-07	6.85e-05%	0.0	1	0.000396	0.000531	1.71e-05	4.05e-06	0.000121%
**L1156**	**False**	**koll11**	7.52e-07	6.85e-05%	0.0	1	0.00606	0.000985	0.00466	4.51e-06	0.000135%
**OTU_6575**	**True**	**ZB2**	7.49e-07	6.82e-05%	0.0	1	0.000232	2.05e-05	4.27e-05	1.64e-06	4.91e-05%
**L870**	**False**	**koll11**	7.49e-07	6.81e-05%	0.0	1	0.000327	3.15e-05	1.23e-05	1.61e-06	4.84e-05%
**OTU_6217**	**True**	**ZB2**	7.45e-07	6.78e-05%	0.0	1	0.0	1.85e-05	0.000148	2.46e-06	7.39e-05%
**L1436**	**False**	**ZB2**	7.44e-07	6.77e-05%	0.0	1	0.00198	0.000812	0.000235	2.89e-06	8.67e-05%
**OTU_8162**	**True**	**ABY1**	7.42e-07	6.75e-05%	0.0	1	0.0	9.66e-05	1.94e-05	4.75e-06	0.000142%
**L865**	**False**	**koll11**	7.37e-07	6.71e-05%	0.0	1	0.000595	4.9e-05	0.000143	1.84e-06	5.53e-05%
**OTU_6190**	**True**	**ZB2**	7.37e-07	6.71e-05%	0.0	1	0.000333	0.000307	2.23e-05	4.25e-06	0.000128%
**L749**	**False**	**PBS-25**	7.34e-07	6.68e-05%	0.0	1	0.000501	5.72e-05	0.0	1.74e-06	5.23e-05%
**OTU_8246**	**True**	**OD1**	7.28e-07	6.62e-05%	0.0	1	0.00206	0.00022	7.3e-05	1.11e-06	3.33e-05%
**OTU_8761**	**True**	**OD1**	7.25e-07	6.6e-05%	0.0	1	0.000128	2.3e-05	5.25e-07	1.74e-06	5.22e-05%
**OTU_9088**	**True**	**ABY1**	7.24e-07	6.59e-05%	0.0	1	8.4e-06	0.000141	0.0	2.75e-06	8.24e-05%

**L1024**	**False**	**koll11**	7.23e-07	6.58e-05%	0.0	1	0.00334	0.000791	0.00143	4.6e-06	0.000138%
**OTU_2885**	**True**	**PBS-25**	7.22e-07	6.57e-05%	0.0	1	0.000405	0.000102	3.5e-06	1.72e-06	5.16e-05%
**OTU_16**	**True**	**ZB2**	7.21e-07	6.56e-05%	0.0	1	5.12e-05	0.0	0.0	1.22e-06	3.66e-05%
**OTU_1456**	**True**	**kpj58rc**	7.2e-07	6.56e-05%	0.0	1	8.21e-05	4.94e-06	0.0	1.57e-06	4.72e-05%
**L1366**	**False**	**ZB2**	7.17e-07	6.52e-05%	0.0	1	1.39e-06	0.000934	0.000268	1.7e-06	5.11e-05%
**L1066**	**False**	**koll11**	7.15e-07	6.51e-05%	0.0	1	0.00131	4.34e-05	0.000157	1.52e-06	4.57e-05%
**OTU_619**	**True**	**koll11**	7.13e-07	6.49e-05%	0.0	1	0.000167	1.35e-05	0.0	1.6e-06	4.81e-05%
**OTU_7937**	**True**	**ABY1**	7.12e-07	6.48e-05%	0.0	1	0.0005	0.0	0.0	1.37e-06	4.1e-05%
**OTU_10851**	**True**	**OP11-4**	7.09e-07	6.45e-05%	0.0	1	0.000114	1.77e-07	0.0	1.18e-06	3.53e-05%
**L797**	**False**	**PBS-25**	7.09e-07	6.45e-05%	0.0	1	0.000513	0.000102	3.5e-06	1.55e-06	4.65e-05%
**OTU_4507**	**True**	**koll11**	7.02e-07	6.39e-05%	0.0	1	9.46e-05	1.73e-06	0.0	1.6e-06	4.81e-05%
**L1434**	**False**	**ZB2**	7e-07	6.37e-05%	0.0	1	0.00198	0.000812	0.000905	7.92e-06	0.000238%
**L265**	**False**	**ABY1**	6.96e-07	6.34e-05%	0.0	1	0.00226	0.00151	0.000986	1.26e-05	0.000379%
**L1907**	**False**	**OD1**	6.94e-07	6.32e-05%	0.0	1	0.000755	4.2e-05	5.97e-06	9.8e-07	2.94e-05%
**OTU_602**	**True**	**koll11**	6.85e-07	6.24e-05%	0.0	1	0.000197	7.6e-05	1.97e-05	3.76e-06	0.000113%
**L18**	**False**	**OD1**	6.85e-07	6.23e-05%	0.0	1	0.00027	3.27e-05	5.45e-06	1.22e-06	3.67e-05%
**OTU_5241**	**True**	**Mb-NB09**	6.81e-07	6.2e-05%	0.0	1	0.0	8.58e-05	0.0	1.98e-06	5.93e-05%
**OTU_9084**	**True**	**TM7-1**	6.78e-07	6.17e-05%	0.0	1	6.01e-05	0.000223	1.63e-05	5.41e-06	0.000162%
**L414**	**False**	**ABY1**	6.74e-07	6.14e-05%	0.0	1	0.000204	0.000488	0.00154	4.86e-06	0.000146%
**OTU_3345**	**True**	**PBS-25**	6.72e-07	6.11e-05%	0.0	1	0.000175	0.0	2.35e-07	1.23e-06	3.7e-05%
**OTU_421**	**True**	**koll11**	6.69e-07	6.09e-05%	0.0	1	0.000582	1.62e-05	4.75e-06	8.13e-07	2.44e-05%
**OTU_1428**	**True**	**GIF10**	6.67e-07	6.07e-05%	0.0	1	8.93e-05	4.59e-06	1.44e-05	2.31e-06	6.95e-05%
**L997**	**False**	**koll11**	6.64e-07	6.04e-05%	0.0	1	0.000809	0.000448	0.00029	8.2e-06	0.000246%
**OTU_10757**	**True**	**OP11-4**	6.62e-07	6.03e-05%	0.0	1	0.000293	0.000164	5.7e-05	4.65e-06	0.00014%
**OTU_8390**	**True**	**ABY1**	6.62e-07	6.02e-05%	0.0	1	0.000369	2.22e-05	4.17e-06	1.66e-06	4.98e-05%
**OTU_282**	**True**	**koll11**	6.54e-07	5.96e-05%	0.0	1	9.22e-05	5.59e-06	2.98e-06	1.38e-06	4.15e-05%
**OTU_1061**	**True**	**PBS-25**	6.54e-07	5.96e-05%	0.0	1	8.38e-05	2.26e-05	0.0	3.05e-06	9.16e-05%
**OTU_6574**	**True**	**ZB2**	6.54e-07	5.95e-05%	0.0	1	0.000317	5.95e-05	1.69e-05	1.59e-06	4.76e-05%
**OTU_5626**	**True**	**OD1**	6.52e-07	5.94e-05%	0.0	1	0.000103	0.0	0.0	1.18e-06	3.55e-05%
**L780**	**False**	**PBS-25**	6.51e-07	5.93e-05%	0.0	1	0.000564	0.000133	5.83e-08	1.51e-06	4.53e-05%
**OTU_3013**	**True**	**PBS-25**	6.48e-07	5.9e-05%	0.0	1	1.43e-05	0.000255	0.0	2.75e-06	8.25e-05%
**OTU_9434**	**True**	**ABY1**	6.44e-07	5.86e-05%	0.0	1	9.47e-05	0.0	0.0	9.93e-07	2.98e-05%
**OTU_9703**	**True**	**Mb-NB09**	6.39e-07	5.82e-05%	0.0	1	0.000127	0.0	0.0	1.5e-06	4.5e-05%
**L401**	**False**	**ABY1**	6.39e-07	5.81e-05%	0.0	1	0.0069	0.00337	0.000848	2.06e-06	6.18e-05%
**OTU_10816**	**True**	**OP11-4**	6.36e-07	5.79e-05%	0.0	1	0.000154	8.11e-05	4.27e-06	3.12e-06	9.36e-05%
**L543**	**False**	**SJA-4**	6.35e-07	5.78e-05%	0.0	1	0.00155	0.000451	0.00194	7.73e-06	0.000232%
**L567**	**False**	**SJA-4**	6.34e-07	5.77e-05%	0.0	1	0.00073	3.47e-07	0.000456	3.19e-06	9.57e-05%
**OTU_8350**	**True**	**ABY1**	6.3e-07	5.73e-05%	0.0	1	8.75e-05	0.0	0.0	1.02e-06	3.07e-05%
**OTU_1550**	**True**	**PRR-12**	6.3e-07	5.73e-05%	0.0	1	0.000183	1.62e-05	0.0	1.46e-06	4.39e-05%
**OTU_6533**	**True**	**OD1**	6.28e-07	5.71e-05%	0.0	1	0.0	0.000389	0.000607	3.26e-06	9.77e-05%
**OTU_4039**	**True**	**koll11**	6.2e-07	5.65e-05%	0.0	1	0.000109	4.33e-05	0.0	2.73e-06	8.2e-05%
**OTU_6076**	**True**	**SM2F11**	6.18e-07	5.62e-05%	0.0	1	8.43e-05	1.27e-05	0.0	1.72e-06	5.16e-05%
**L1347**	**False**	**ZB2**	6.15e-07	5.6e-05%	0.0	1	0.000542	0.000225	0.000139	3.85e-06	0.000116%
**OTU_6160**	**True**	**ZB2**	6.07e-07	5.53e-05%	0.0	1	4.51e-05	0.000696	0.000548	2.26e-06	6.78e-05%
**L25**	**False**	**Bacteria**	6.07e-07	5.52e-05%	0.0	1	0.994	0.997	1.0	-3.93486222055e-07	-1.18085856031e-05%
**L591**	**False**	**PRR-12**	6.06e-07	5.52e-05%	0.0	1	0.00175	0.000151	0.000169	9.33e-07	2.8e-05%
**OTU_6617**	**True**	**ZB2**	6.05e-07	5.51e-05%	0.0	1	0.0	0.000265	3.27e-05	2.19e-06	6.57e-05%
**OTU_466**	**True**	**koll11**	6.03e-07	5.49e-05%	0.0	1	0.000173	0.00027	8.15e-05	1.82e-05	0.000547%
**L650**	**False**	**koll11**	5.96e-07	5.42e-05%	0.0	1	0.000397	3.92e-05	3.54e-05	1.76e-06	5.27e-05%
**OTU_211**	**True**	**koll11**	5.94e-07	5.41e-05%	0.0	1	5.27e-05	7.58e-05	0.0	4.35e-06	0.000131%
**OTU_19**	**True**	**ZB2**	5.92e-07	5.39e-05%	0.0	1	0.000127	7.61e-06	1.8e-05	1.82e-06	5.46e-05%
**L1814**	**False**	**OD1**	5.88e-07	5.35e-05%	0.0	1	0.000905	0.000715	0.000115	4.44e-06	0.000133%
**L545**	**False**	**SJA-4**	5.85e-07	5.33e-05%	0.0	1	0.000377	0.000181	0.00155	3.39e-06	0.000102%
**OTU_1538**	**True**	**PBS-25**	5.8e-07	5.28e-05%	0.0	1	0.000133	5.72e-05	0.0	2.72e-06	8.17e-05%
**OTU_1067**	**True**	**koll11**	5.8e-07	5.28e-05%	0.0	1	0.000251	4.9e-05	0.000132	4.52e-06	0.000136%
**L668**	**False**	**koll11**	5.77e-07	5.26e-05%	0.0	1	0.000681	0.000591	0.000127	7.47e-06	0.000224%
**OTU_8168**	**True**	**ZB2**	5.68e-07	5.17e-05%	0.0	1	0.0	8.29e-05	0.0	2.07e-06	6.2e-05%
**OTU_6199**	**True**	**ZB2**	5.68e-07	5.17e-05%	0.0	1	0.000325	0.0	0.0	1.14e-06	3.43e-05%
**OTU_8787**	**True**	**ABY1**	5.66e-07	5.15e-05%	0.0	1	6.2e-05	0.000303	0.000198	9.19e-06	0.000276%
**L1418**	**False**	**ZB2**	5.53e-07	5.04e-05%	0.0	1	0.000462	6.77e-05	0.000689	4.75e-06	0.000142%
**OTU_2442**	**True**	**koll11**	5.49e-07	5e-05%	0.0	1	0.000105	1.5e-05	0.0	1.97e-06	5.92e-05%
**OTU_4179**	**True**	**koll11**	5.48e-07	4.99e-05%	0.0	1	0.000746	4.73e-05	0.00145	3.18e-06	9.54e-05%
**OTU_171**	**True**	**koll11**	5.44e-07	4.96e-05%	0.0	1	0.00109	0.000538	0.000802	1.12e-05	0.000337%
**OTU_4272**	**True**	**koll11**	5.44e-07	4.95e-05%	0.0	1	0.000464	0.000222	1.88e-05	2.03e-06	6.09e-05%

**OTU_2664**	**True**	**koll11**	5.43e-07	4.94e-05%	0.0	1	9.29e-05	6.58e-05	2.8e-06	3.18e-06	9.56e-05%
**L883**	**False**	**koll11**	5.4e-07	4.91e-05%	0.0	1	0.00332	0.00169	0.000588	2.44e-06	7.33e-05%
**OTU_4071**	**True**	**koll11**	5.38e-07	4.9e-05%	0.0	1	0.000341	4.28e-05	1.11e-05	1.28e-06	3.84e-05%
**OTU_7173**	**True**	**OD1**	5.35e-07	4.87e-05%	0.0	1	0.00011	0.000149	2.88e-06	4.15e-06	0.000125%
**OTU_4770**	**True**	**GIF10**	5.35e-07	4.87e-05%	0.0	1	0.000272	6.93e-05	0.000164	6.16e-06	0.000185%
**OTU_129**	**True**	**OD1**	5.32e-07	4.84e-05%	0.0	1	8.14e-06	6.62e-05	3.45e-05	9.08e-06	0.000272%
**OTU_470**	**True**	**koll11**	5.28e-07	4.8e-05%	0.0	1	0.0	0.000107	0.0	1.53e-06	4.58e-05%
**OTU_912**	**True**	**BD4-9**	5.28e-07	4.8e-05%	0.0	1	0.00029	0.0	0.0	9.82e-07	2.95e-05%
**L1243**	**False**	**TM7-1**	5.26e-07	4.79e-05%	0.0	1	0.0117	0.012	0.0151	0.000122	0.00368%
**OTU_7016**	**True**	**ZB2**	5.25e-07	4.78e-05%	0.0	1	0.0	0.000107	2.18e-05	1.85e-06	5.54e-05%
**OTU_4334**	**True**	**koll11**	5.25e-07	4.77e-05%	0.0	1	0.00105	0.000235	0.00046	2.9e-06	8.69e-05%
**OTU_8638**	**True**	**ABY1**	5.19e-07	4.72e-05%	0.0	1	0.000149	0.0	0.0	9.62e-07	2.89e-05%
**OTU_5076**	**True**	**ZB2**	5.08e-07	4.62e-05%	0.0	1	0.000213	5.21e-05	8.16e-05	3e-06	9.02e-05%
**OTU_2628**	**True**	**SJA-4**	5.07e-07	4.61e-05%	0.0	1	0.0	3.74e-05	0.000127	3.34e-06	0.0001%
**L559**	**False**	**SJA-4**	5.06e-07	4.6e-05%	0.0	1	0.00171	0.000531	0.000474	4.15e-06	0.000125%
**L373**	**False**	**ABY1**	4.99e-07	4.54e-05%	0.0	1	0.000429	0.000226	0.000229	1.19e-05	0.000357%
**OTU_8156**	**True**	**OP11-4**	4.98e-07	4.53e-05%	0.0	1	3.18e-05	6.35e-05	1.24e-05	8.69e-06	0.000261%
**OTU_1982**	**True**	**koll11**	4.95e-07	4.5e-05%	0.0	1	0.000131	4.24e-05	1.41e-05	2.67e-06	8.01e-05%
**L1081**	**False**	**koll11**	4.95e-07	4.5e-05%	0.0	1	0.000364	4.86e-05	3.3e-05	1.55e-06	4.65e-05%
**OTU_10815**	**True**	**OP11-4**	4.94e-07	4.49e-05%	0.0	1	0.0	0.0	3.88e-05	1.64e-06	4.92e-05%
**OTU_9148**	**True**	**ABY1**	4.92e-07	4.48e-05%	0.0	1	9.16e-06	9.61e-05	0.0	2.03e-06	6.1e-05%
**L1762**	**False**	**OD1**	4.89e-07	4.45e-05%	0.0	1	0.000359	0.000211	0.000298	2.92e-05	0.000875%
**OTU_6693**	**True**	**ZB2**	4.83e-07	4.39e-05%	0.0	1	0.0	0.000286	8.01e-05	3.05e-06	9.17e-05%
**OTU_8045**	**True**	**OD1**	4.77e-07	4.35e-05%	0.0	1	0.000653	0.000575	0.000149	4.36e-06	0.000131%
**OTU_402**	**True**	**PBS-25**	4.76e-07	4.33e-05%	0.0	1	0.0	0.0	0.000175	1.24e-06	3.72e-05%
**OTU_1319**	**True**	**koll11**	4.73e-07	4.3e-05%	0.0	1	0.000186	0.000377	2.69e-05	4.02e-06	0.000121%
**OTU_8181**	**True**	**OP11-4**	4.72e-07	4.3e-05%	0.0	1	0.000105	6.11e-06	0.000132	2.74e-06	8.22e-05%
**OTU_9183**	**True**	**ABY1**	4.72e-07	4.29e-05%	0.0	1	0.000335	6.63e-05	2.45e-05	1.68e-06	5.05e-05%
**L903**	**False**	**koll11**	4.7e-07	4.28e-05%	0.0	1	0.000788	0.000352	0.000582	8.35e-06	0.000251%
**OTU_7089**	**True**	**ABY1**	4.54e-07	4.13e-05%	0.0	1	9.12e-05	2.6e-05	7.06e-07	1.54e-06	4.61e-05%
**OTU_305**	**True**	**PBS-25**	4.5e-07	4.1e-05%	0.0	1	0.000166	8.15e-06	0.000272	2.42e-06	7.27e-05%
**L1477**	**False**	**ZB2**	4.5e-07	4.09e-05%	0.0	1	0.000675	0.000387	0.000302	9.46e-06	0.000284%
**OTU_1725**	**True**	**koll11**	4.48e-07	4.08e-05%	0.0	1	4.28e-05	0.0	0.0	9.63e-07	2.89e-05%
**OTU_2558**	**True**	**SJA-4**	4.47e-07	4.07e-05%	0.0	1	3.61e-05	0.00011	1.2e-05	4.26e-06	0.000128%
**OTU_1496**	**True**	**koll11**	4.45e-07	4.05e-05%	0.0	1	0.000121	6.93e-05	0.0	1.94e-06	5.81e-05%
**OTU_5311**	**True**	**OD1**	4.45e-07	4.05e-05%	0.0	1	0.000259	0.000132	2.02e-05	2.11e-06	6.34e-05%
**OTU_7976**	**True**	**ABY1**	4.41e-07	4.01e-05%	0.0	1	2.27e-05	0.00014	2.45e-05	3.63e-06	0.000109%
**OTU_880**	**True**	**koll11**	4.31e-07	3.92e-05%	0.0	1	7.26e-05	0.0	0.0	1.09e-06	3.26e-05%
**L151**	**False**	**ABY1**	4.31e-07	3.92e-05%	0.0	1	0.000657	0.000332	0.000201	4.82e-06	0.000145%
**L885**	**False**	**koll11**	4.27e-07	3.89e-05%	0.0	1	0.00183	0.00155	0.000255	2.33e-06	7e-05%
**L21**	**False**	**Bacteria**	4.25e-07	3.87e-05%	0.0	1	0.994	0.997	1.0	-2.8405084632e-07	-8.52441215576e-06%
**OTU_5955**	**True**	**OD1**	4.25e-07	3.87e-05%	0.0	1	0.000121	3.75e-05	1.03e-05	1.92e-06	5.77e-05%
**OTU_10860**	**True**	**OP11**	4.23e-07	3.85e-05%	0.0	1	0.000271	0.000315	8.21e-06	2.32e-06	6.97e-05%
**OTU_8732**	**True**	**ABY1**	4.21e-07	3.83e-05%	0.0	1	0.000215	8.77e-05	7.49e-06	1.43e-06	4.29e-05%
**OTU_6316**	**True**	**ZB2**	4.18e-07	3.8e-05%	0.0	1	0.000462	6.77e-05	2.87e-05	1e-06	3e-05%
**OTU_9149**	**True**	**TM7-1**	4.14e-07	3.77e-05%	0.0	1	0.0	0.000175	0.0	1.2e-06	3.6e-05%
**OTU_718**	**True**	**PBS-25**	4.14e-07	3.77e-05%	0.0	1	0.000496	0.000107	2.73e-05	1.11e-06	3.34e-05%
**OTU_6059**	**True**	**ABY1**	4.14e-07	3.76e-05%	0.0	1	0.0	3.35e-05	6.21e-06	2.62e-06	7.86e-05%
**OTU_603**	**True**	**koll11**	4.11e-07	3.74e-05%	0.0	1	9.45e-05	4.06e-06	0.0	9.55e-07	2.87e-05%
**OTU_5444**	**True**	**OD1**	4.07e-07	3.71e-05%	0.0	1	6.17e-05	1.9e-05	2.28e-06	1.73e-06	5.19e-05%
**L897**	**False**	**koll11**	4.06e-07	3.7e-05%	0.0	1	0.0016	0.000756	0.000867	5.47e-06	0.000164%
**OTU_8169**	**True**	**TM7-3**	4.05e-07	3.68e-05%	0.0	1	3.44e-06	0.000104	0.0	1.68e-06	5.05e-05%
**OTU_3798**	**True**	**PBS-25**	4.03e-07	3.67e-05%	0.0	1	0.000108	0.0	0.0	8.6e-07	2.58e-05%
**OTU_5818**	**True**	**OD1**	3.98e-07	3.62e-05%	0.0	1	0.00145	0.000193	0.000385	1.11e-06	3.34e-05%
**OTU_7954**	**True**	**OD1**	3.94e-07	3.59e-05%	0.0	1	0.000628	0.000126	4.67e-05	1.35e-06	4.05e-05%
**L755**	**False**	**PBS-25**	3.91e-07	3.56e-05%	0.0	1	0.00119	0.000117	0.00176	2.59e-06	7.78e-05%
**L179**	**False**	**SM2F11**	3.9e-07	3.55e-05%	0.0	1	0.00029	0.000195	2.38e-05	2.11e-06	6.33e-05%
**L290**	**False**	**ABY1**	3.89e-07	3.54e-05%	0.0	1	7.06e-05	0.000141	1.34e-05	4.32e-06	0.00013%
**L891**	**False**	**koll11**	3.85e-07	3.5e-05%	0.0	1	0.000371	0.0001	0.0	1.22e-06	3.65e-05%
**OTU_10759**	**True**	**OP11-4**	3.83e-07	3.48e-05%	0.0	1	0.000442	0.000106	1.52e-05	6.84e-07	2.05e-05%
**OTU_353**	**True**	**koll11**	3.8e-07	3.46e-05%	0.0	1	0.000496	0.000445	0.000195	9.5e-06	0.000285%
**OTU_7964**	**True**	**ABY1**	3.78e-07	3.44e-05%	0.0	1	7.56e-05	0.0	0.0	8.68e-07	2.61e-05%
**L1759**	**False**	**OD1**	3.76e-07	3.42e-05%	0.0	1	0.000272	0.00055	0.000214	1.66e-05	0.000498%
**OTU_875**	**True**	**koll11**	3.74e-07	3.4e-05%	0.0	1	0.000105	1.77e-07	0.0	7.45e-07	2.24e-05%

**L553**	**False**	**SJA-4**	3.72e-07	3.38e-05%	0.0	1	0.000624	0.00027	0.000268	6.89e-06	0.000207%
**L36**	**False**	**ABY1**	3.72e-07	3.38e-05%	0.0	1	0.0003	1.52e-05	5.83e-08	5.43e-07	1.63e-05%
**OTU_6694**	**True**	**ZB2**	3.68e-07	3.35e-05%	0.0	1	0.0	0.000769	0.0	9.97e-07	2.99e-05%
**OTU_397**	**True**	**koll11**	3.64e-07	3.31e-05%	0.0	1	0.000194	0.000191	2.7e-05	2.9e-06	8.71e-05%
**OTU_287**	**True**	**koll11**	3.62e-07	3.3e-05%	0.0	1	0.000233	4.07e-05	1.26e-05	1.01e-06	3.02e-05%
**OTU_9588**	**True**	**ABY1**	3.61e-07	3.29e-05%	0.0	1	6.22e-05	0.0	1.34e-05	1.23e-06	3.69e-05%
**OTU_4088**	**True**	**koll11**	3.58e-07	3.26e-05%	0.0	1	0.000172	3.92e-05	3.54e-05	2.36e-06	7.07e-05%
**L1625**	**False**	**OD1**	3.56e-07	3.24e-05%	0.0	1	0.000208	0.00018	4.3e-05	3.94e-06	0.000118%
**L3**	**False**	**Bacteria**	3.53e-07	3.22e-05%	0.0	1	0.998	0.999	1.0	-6.56418777537e-08	-1.96992344117e-06%
**OTU_6157**	**True**	**ZB2**	3.52e-07	3.2e-05%	0.0	1	0.000317	7.78e-05	8.4e-05	1.69e-06	5.08e-05%
**OTU_5953**	**True**	**OD1**	3.49e-07	3.18e-05%	0.0	1	0.000358	0.0	7.26e-05	8.4e-07	2.52e-05%
**OTU_277**	**True**	**koll11**	3.45e-07	3.14e-05%	0.0	1	0.000312	2.56e-06	9.54e-05	8.21e-07	2.47e-05%
**L901**	**False**	**koll11**	3.41e-07	3.11e-05%	0.0	1	0.00107	0.000358	0.00084	3.21e-06	9.63e-05%
**OTU_456**	**True**	**koll11**	3.39e-07	3.09e-05%	0.0	1	0.000234	0.000138	3.12e-05	2.45e-06	7.34e-05%
**L152**	**False**	**ABY1**	3.38e-07	3.08e-05%	0.0	1	0.000408	0.000313	0.000198	1.14e-05	0.000341%
**L1205**	**False**	**koll11**	3.31e-07	3.02e-05%	0.0	1	0.000144	0.00024	6.66e-05	6.67e-06	0.0002%
**OTU_6099**	**True**	**OD1**	3.31e-07	3.01e-05%	0.0	1	0.000227	2.62e-05	4.48e-06	1.16e-06	3.48e-05%
**OTU_424**	**True**	**PBS-25**	3.21e-07	2.93e-05%	0.0	1	0.000117	0.00016	1.17e-07	1.8e-06	5.39e-05%
**OTU_5435**	**True**	**OD1**	3.2e-07	2.91e-05%	0.0	1	0.000176	0.000146	4.02e-05	3.93e-06	0.000118%
**OTU_6251**	**True**	**ZB2**	3.2e-07	2.91e-05%	0.0	1	0.000172	3.57e-05	2.48e-05	1.5e-06	4.51e-05%
**OTU_3150**	**True**	**TM6**	3.18e-07	2.89e-05%	0.0	1	0.000162	3.09e-05	3.13e-06	9.08e-07	2.72e-05%
**OTU_8738**	**True**	**OD1**	3.14e-07	2.86e-05%	0.0	1	0.0	1.31e-05	7.32e-05	1.57e-06	4.72e-05%
**L1053**	**False**	**koll11**	3.1e-07	2.82e-05%	0.0	1	0.00441	0.000685	0.00235	1.31e-06	3.93e-05%
**OTU_8089**	**True**	**ABY1**	3.08e-07	2.81e-05%	0.0	1	0.0	0.000122	5.22e-05	2.03e-06	6.08e-05%
**OTU_8669**	**True**	**EW055**	3.08e-07	2.81e-05%	0.0	1	0.00013	0.0	4.94e-05	1.34e-06	4.01e-05%
**OTU_7939**	**True**	**OD1**	3.08e-07	2.8e-05%	0.0	1	6.92e-05	2.15e-05	5.31e-06	1.29e-06	3.88e-05%
**OTU_2708**	**True**	**koll11**	3.07e-07	2.79e-05%	0.0	1	0.000273	6.73e-05	7.31e-05	1.86e-06	5.58e-05%
**OTU_4986**	**True**	**ZB2**	3.06e-07	2.79e-05%	0.0	1	0.000329	0.000173	5.73e-05	2.53e-06	7.58e-05%
**L541**	**False**	**SJA-4**	3.03e-07	2.76e-05%	0.0	1	0.00159	0.000561	0.00195	4.49e-06	0.000135%
**L637**	**False**	**PBS-25**	2.99e-07	2.72e-05%	0.0	1	0.000218	6.3e-05	4.1e-06	1.24e-06	3.73e-05%
**OTU_528**	**True**	**koll11**	2.97e-07	2.7e-05%	0.0	1	9.69e-05	1.25e-05	1.85e-05	1.37e-06	4.12e-05%
**L448**	**False**	**OP11-4**	2.96e-07	2.7e-05%	0.0	1	0.000114	1.33e-05	0.000217	1.57e-06	4.71e-05%
**OTU_5883**	**True**	**OD1**	2.95e-07	2.69e-05%	0.0	1	0.000116	8.2e-07	1.54e-05	7.5e-07	2.25e-05%
**L617**	**False**	**BD4-9**	2.94e-07	2.67e-05%	0.0	1	0.000102	0.000132	0.0	1.95e-06	5.86e-05%
**OTU_7974**	**True**	**ABY1**	2.86e-07	2.6e-05%	0.0	1	0.000104	0.000141	0.000218	2.6e-05	0.000779%
**OTU_9719**	**True**	**Mb-NB09**	2.83e-07	2.58e-05%	0.0	1	8.78e-05	0.0	5.89e-06	9.04e-07	2.71e-05%
**OTU_6920**	**True**	**OD1**	2.81e-07	2.56e-05%	0.0	1	0.00011	0.0	0.0	5.08e-07	1.53e-05%
**L219**	**False**	**ABY1**	2.8e-07	2.54e-05%	0.0	1	0.00143	0.00175	0.00117	5.5e-05	0.00165%
**L1748**	**False**	**OD1**	2.78e-07	2.53e-05%	0.0	1	0.00134	0.00135	0.00184	5.4e-05	0.00162%
**OTU_6645**	**True**	**ZB2**	2.78e-07	2.53e-05%	0.0	1	0.000157	0.00016	3.64e-05	3.45e-06	0.000104%
**OTU_6633**	**True**	**ZB2**	2.71e-07	2.47e-05%	0.0	1	0.000141	0.000108	6.31e-05	8.38e-06	0.000252%
**L1489**	**False**	**ZB2**	2.68e-07	2.44e-05%	0.0	1	0.000377	0.000102	9.1e-05	1.19e-06	3.57e-05%
**OTU_7852**	**True**	**ZB2**	2.57e-07	2.34e-05%	0.0	1	4.74e-05	0.0	0.0	6.74e-07	2.02e-05%
**OTU_6019**	**True**	**ABY1**	2.54e-07	2.31e-05%	0.0	1	0.0001	0.000111	3.62e-05	5.46e-06	0.000164%
**OTU_5997**	**True**	**OD1**	2.53e-07	2.3e-05%	0.0	1	5.39e-05	0.000156	0.000125	2.34e-05	0.000703%
**OTU_928**	**True**	**koll11**	2.52e-07	2.29e-05%	0.0	1	0.0	9.22e-05	0.0	9.06e-07	2.72e-05%
**OTU_2288**	**True**	**PBS-25**	2.51e-07	2.29e-05%	0.0	1	4.93e-05	1.02e-05	0.0	7.32e-07	2.2e-05%
**OTU_460**	**True**	**koll11**	2.51e-07	2.28e-05%	0.0	1	1.91e-05	0.000118	0.0	1.18e-06	3.54e-05%
**OTU_6066**	**True**	**OD1**	2.48e-07	2.26e-05%	0.0	1	1.89e-05	0.000107	1.58e-05	2.41e-06	7.22e-05%
**OTU_2611**	**True**	**SJA-4**	2.43e-07	2.21e-05%	0.0	1	6.36e-05	0.000158	2.35e-07	1e-06	3e-05%
**L1211**	**False**	**GIF10**	2.43e-07	2.21e-05%	0.0	1	0.000171	9.52e-06	1.44e-05	5.53e-07	1.66e-05%
**L869**	**False**	**koll11**	2.42e-07	2.21e-05%	0.0	1	0.00074	3.55e-05	1.23e-05	3.66e-07	1.1e-05%
**L27**	**False**	**Bacteria**	2.37e-07	2.16e-05%	0.0	1	0.992	0.997	1.0	-2.34642430207e-07	-7.04165754203e-06%
**OTU_5486**	**True**	**OD1**	2.36e-07	2.15e-05%	0.0	1	0.000142	0.0	0.0	4.76e-07	1.43e-05%
**L288**	**False**	**ABY1**	2.31e-07	2.11e-05%	0.0	1	0.000286	0.000153	1.34e-05	1.18e-06	3.56e-05%
**OTU_8795**	**True**	**Mb-NB09**	2.29e-07	2.09e-05%	0.0	1	0.000158	5.77e-05	0.00013	2.98e-06	8.95e-05%
**OTU_8328**	**True**	**OD1**	2.29e-07	2.09e-05%	0.0	1	0.000114	2.44e-05	5.39e-05	1.79e-06	5.38e-05%
**L1763**	**False**	**OD1**	2.12e-07	1.93e-05%	0.0	1	0.000268	0.000211	0.000298	3.82e-05	0.00115%
**L11**	**False**	**Bacteria**	2.12e-07	1.93e-05%	0.0	1	0.995	0.998	1.0	-9.17483848654e-08	-2.75338397104e-06%
**L221**	**False**	**ABY1**	2.1e-07	1.91e-05%	0.0	1	0.00106	0.00173	0.00114	4.28e-05	0.00128%
**OTU_6516**	**True**	**OD1**	2.07e-07	1.88e-05%	0.0	1	7.23e-05	3.76e-05	1.32e-05	2.11e-06	6.35e-05%
**L1030**	**False**	**koll11**	2.05e-07	1.86e-05%	0.0	1	0.000901	0.000142	0.000767	1.56e-06	4.67e-05%
**OTU_1326**	**True**	**PBS-25**	2.03e-07	1.85e-05%	0.0	1	5.3e-06	5.1e-05	0.0	1.03e-06	3.08e-05%

**OTU_1514**	**True**	**koll11**	1.99e-07	1.82e-05%	0.0	1	7.85e-05	1.96e-05	0.0	6.95e-07	2.09e-05%
**L217**	**False**	**ABY1**	1.99e-07	1.81e-05%	0.0	1	0.00157	0.00178	0.00117	2.36e-05	0.000707%
**OTU_2845**	**True**	**PBS-25**	1.98e-07	1.81e-05%	0.0	1	0.000365	0.000382	0.000106	2.34e-06	7.02e-05%
**OTU_5270**	**True**	**OD1**	1.96e-07	1.78e-05%	0.0	1	0.000122	2.94e-05	1.35e-05	1.13e-06	3.38e-05%
**OTU_6642**	**True**	**ZB2**	1.94e-07	1.77e-05%	0.0	1	0.000169	2.2e-05	3.64e-06	5.05e-07	1.52e-05%
**L1224**	**False**	**koll11**	1.91e-07	1.74e-05%	0.0	1	0.000395	0.000283	0.00043	1.53e-05	0.000458%
**OTU_1439**	**True**	**koll11**	1.9e-07	1.73e-05%	0.0	1	0.000104	2.83e-05	0.0	6.39e-07	1.92e-05%
**OTU_9194**	**True**	**ABY1**	1.89e-07	1.72e-05%	0.0	1	5.37e-05	0.0	1.49e-05	6.89e-07	2.07e-05%
**OTU_6682**	**True**	**ZB2**	1.85e-07	1.69e-05%	0.0	1	0.0	0.000142	5.39e-06	6.29e-07	1.89e-05%
**OTU_8641**	**True**	**ABY1**	1.83e-07	1.66e-05%	0.0	1	9e-05	2.41e-05	0.0	7.32e-07	2.2e-05%
**OTU_9063**	**True**	**Mb-NB09**	1.82e-07	1.66e-05%	0.0	1	0.000231	7.03e-05	0.0	6.01e-07	1.8e-05%
**OTU_1331**	**True**	**koll11**	1.82e-07	1.65e-05%	0.0	1	3.62e-05	0.000187	4.63e-05	2.28e-06	6.84e-05%
**OTU_1058**	**True**	**koll11**	1.82e-07	1.65e-05%	0.0	1	0.000535	0.000321	0.000565	6.85e-06	0.000206%
**L1439**	**False**	**ZB2**	1.79e-07	1.63e-05%	0.0	1	0.000302	8.04e-05	9.29e-05	1.21e-06	3.62e-05%
**OTU_8041**	**True**	**TM7-3**	1.78e-07	1.62e-05%	0.0	1	5.72e-05	0.0	0.0	4.01e-07	1.2e-05%
**OTU_3297**	**True**	**PBS-25**	1.77e-07	1.61e-05%	0.0	1	0.000332	0.000102	0.00022	1.45e-06	4.35e-05%
**OTU_1626**	**True**	**PBS-25**	1.76e-07	1.6e-05%	0.0	1	0.000196	0.0	0.0	2.94e-07	8.83e-06%
**OTU_6085**	**True**	**ZB2**	1.73e-07	1.57e-05%	0.0	1	0.000161	0.000261	3.14e-06	1.2e-06	3.6e-05%
**OTU_5924**	**True**	**OD1**	1.63e-07	1.49e-05%	0.0	1	0.000105	0.000232	2.91e-07	7.99e-07	2.4e-05%
**OTU_1096**	**True**	**koll11**	1.63e-07	1.48e-05%	0.0	1	0.000108	5.37e-05	2.03e-05	1.16e-06	3.47e-05%
**L1226**	**False**	**koll11**	1.61e-07	1.47e-05%	0.0	1	0.000201	0.000253	0.00043	9.2e-06	0.000276%
**OTU_10804**	**True**	**OP11-4**	1.59e-07	1.45e-05%	0.0	1	0.000332	1.01e-05	8.03e-05	4.42e-07	1.32e-05%
**L965**	**False**	**koll11**	1.58e-07	1.43e-05%	0.0	1	0.00052	0.000565	7.77e-05	1.13e-06	3.39e-05%
**OTU_136**	**True**	**OD1**	1.57e-07	1.43e-05%	0.0	1	3.78e-05	2.88e-05	0.0	7.93e-07	2.38e-05%
**OTU_458**	**True**	**koll11**	1.52e-07	1.38e-05%	0.0	1	2.76e-05	0.000147	3.41e-05	1.9e-06	5.7e-05%
**L1749**	**False**	**OD1**	1.49e-07	1.35e-05%	0.0	1	0.000979	0.00114	0.00154	2.43e-05	0.00073%
**OTU_878**	**True**	**koll11**	1.45e-07	1.32e-05%	0.0	1	8.95e-05	6.29e-06	1.29e-05	5.13e-07	1.54e-05%
**L804**	**False**	**PBS-25**	1.3e-07	1.18e-05%	0.0	1	1.43e-05	0.000255	0.000288	1.71e-06	5.13e-05%
**L1216**	**False**	**koll11**	1.27e-07	1.16e-05%	0.0	1	0.000724	0.000688	0.000423	7.97e-06	0.000239%
**L223**	**False**	**ABY1**	1.24e-07	1.13e-05%	0.0	1	0.00105	0.00107	0.000805	2.77e-05	0.000832%
**OTU_459**	**True**	**koll11**	1.23e-07	1.12e-05%	0.0	1	0.000467	0.000489	7.77e-05	9.97e-07	2.99e-05%
**OTU_5359**	**True**	**Mb-NB09**	1.22e-07	1.11e-05%	0.0	1	6.95e-05	7.63e-07	2.83e-06	3.23e-07	9.68e-06%
**L1619**	**False**	**OD1**	1.2e-07	1.1e-05%	0.0	1	0.00197	0.00242	0.000736	1.58e-06	4.73e-05%
**L1755**	**False**	**OD1**	1.14e-07	1.04e-05%	0.0	1	0.000465	0.000938	0.000821	1.57e-05	0.000472%
**OTU_6580**	**True**	**ZB2**	1.14e-07	1.04e-05%	0.0	1	1.8e-05	5.67e-05	8.01e-05	2.96e-06	8.9e-05%
**OTU_9018**	**True**	**Mb-NB09**	1.1e-07	9.97e-06%	0.0	1	6.6e-05	1.66e-05	3.59e-05	1.29e-06	3.88e-05%
**OTU_1646**	**True**	**PBS-25**	1.07e-07	9.73e-06%	0.0	1	0.000135	1.77e-05	8.44e-05	9.44e-07	2.83e-05%
**OTU_4502**	**True**	**koll11**	1.04e-07	9.44e-06%	0.0	1	0.00017	1.77e-07	0.0	2.2e-07	6.6e-06%
**OTU_8165**	**True**	**ABY1**	1.02e-07	9.33e-06%	0.0	1	3.21e-05	8.76e-05	2.47e-05	2.57e-06	7.71e-05%
**OTU_8234**	**True**	**ABY1**	1.01e-07	9.16e-06%	0.0	1	6.8e-05	5.99e-05	1.81e-05	1.67e-06	5.02e-05%
**L1032**	**False**	**koll11**	9.5e-08	8.65e-06%	0.0	1	0.000632	9.87e-05	0.000767	7.77e-07	2.33e-05%
**L1217**	**False**	**koll11**	9.24e-08	8.41e-06%	0.0	1	0.000155	0.000372	0.000368	1.01e-05	0.000303%
**OTU_4774**	**True**	**koll11**	7.41e-08	6.74e-06%	0.0	1	0.000199	0.000243	0.00012	4.97e-06	0.000149%
**OTU_6684**	**True**	**ZB2**	7.31e-08	6.65e-06%	0.0	1	0.000145	8.18e-05	4.83e-05	1.01e-06	3.04e-05%
**OTU_8154**	**True**	**OP11-4**	7.23e-08	6.58e-06%	0.0	1	7.26e-05	1e-05	1.62e-05	3.76e-07	1.13e-05%
**OTU_8890**	**True**	**ABY1**	6.56e-08	5.97e-06%	0.0	1	0.0	5.47e-06	0.0	1.92e-07	5.75e-06%
**L898**	**False**	**koll11**	6.28e-08	5.72e-06%	0.0	1	0.000524	0.000398	2.69e-05	4.43e-07	1.33e-05%
**OTU_4497**	**True**	**koll11**	5.28e-08	4.8e-06%	0.0	1	0.000141	1.12e-05	0.0	1.41e-07	4.24e-06%
**OTU_1883**	**True**	**koll11**	5.25e-08	4.78e-06%	0.0	1	0.000139	1.48e-05	8.44e-06	1.6e-07	4.8e-06%
**OTU_831**	**True**	**koll11**	4.95e-08	4.5e-06%	0.0	1	0.000295	2.88e-05	0.0	1.04e-07	3.13e-06%
**L1362**	**False**	**OD1**	4.55e-08	4.14e-06%	0.0	1	0.077	0.696	0.738	1.55e-08	4.66e-07%
**OTU_4864**	**True**	**GIF10**	4.54e-08	4.13e-06%	0.0	1	9.21e-05	7.59e-06	0.0	1.06e-07	3.18e-06%
**L1377**	**False**	**OD1**	4.48e-08	4.08e-06%	0.0	1	0.0159	0.564	0.479	2.69e-08	8.07e-07%
**OTU_8167**	**True**	**ZB2**	3.9e-08	3.55e-06%	0.0	1	0.000141	0.000328	0.000177	2.66e-06	7.98e-05%
**L1364**	**False**	**OD1**	3.87e-08	3.53e-06%	0.0	1	0.0654	0.642	0.579	2.01e-08	6.03e-07%
**OTU_8084**	**True**	**ABY1**	3.49e-08	3.18e-06%	0.0	1	0.000122	8.61e-05	0.000166	1.63e-06	4.89e-05%
**L570**	**False**	**SJA-4**	2.29e-08	2.08e-06%	0.0	1	0.000143	0.00023	0.000127	4.1e-06	0.000123%
**OTU_8017**	**True**	**ABY1**	2.04e-08	1.85e-06%	0.001	0	4.16e-05	4.62e-05	2.12e-05	7.85e-07	2.36e-05%
**OTU_5992**	**True**	**ZB2**	1.61e-08	1.47e-06%	0.0	1	0.00244	0.208	0.13	2.53e-08	7.59e-07%
**OTU_1177**	**True**	**OP3**	1.18e-08	1.07e-06%	0.014	0	0.000117	0.000135	8.85e-05	2.41e-06	7.22e-05%
**L970**	**False**	**koll11**	1.17e-08	1.07e-06%	0.0	1	0.187	0.028	0.0363	4.18e-09	1.26e-07%
**L1007**	**False**	**koll11**	5.93e-09	5.4e-07%	0.0	1	0.0996	0.0149	0.0241	3.41e-09	1.02e-07%
**L1433**	**False**	**ZB2**	3.57e-09	3.25e-07%	0.0	1	0.00486	0.0436	0.064	5.04e-09	1.51e-07%
**L808**	**False**	**OP3**	3.37e-09	3.07e-07%	0.0	1	0.0451	0.00553	0.00698	2.73e-09	8.2e-08%
**L953**	**False**	**koll11**	2.32e-09	2.11e-07%	0.0	1	0.0257	0.00305	0.00189	2.1e-09	6.31e-08%

**L1280**	**False**	**TM7-3**	2.22e-09	2.02e-07%	0.0	1	0.0103	0.000291	0.0265	1.1e-08	3.32e-07%
**L1498**	**False**	**ZB2**	2.01e-09	1.83e-07%	0.0	1	0.000497	0.024	0.0253	6.63e-09	1.99e-07%
**L468**	**False**	**OP11-4**	1.83e-09	1.67e-07%	0.0	1	0.0254	0.00548	0.00257	2.01e-09	6.03e-08%
**L319**	**False**	**OD1**	1.59e-09	1.44e-07%	0.0	1	0.018	0.00287	0.00106	1.22e-09	3.65e-08%
**L170**	**False**	**OD1**	1.55e-09	1.42e-07%	0.0	1	0.0416	0.0211	0.00948	3.2e-09	9.59e-08%
**L1042**	**False**	**koll11**	1.5e-09	1.36e-07%	0.0	1	0.0238	0.00306	0.00668	2.74e-09	8.22e-08%
**L1682**	**False**	**OD1**	1.39e-09	1.26e-07%	0.0	1	0.0173	0.0039	0.00109	1.03e-09	3.1e-08%
**L1103**	**False**	**koll11**	1.09e-09	9.9e-08%	0.0	1	0.00857	0.000108	0.000353	1.89e-09	5.66e-08%
**L1043**	**False**	**koll11**	8.18e-10	7.44e-08%	0.0	1	0.0153	0.00232	0.00636	2.11e-09	6.33e-08%
**L230**	**False**	**ABY1**	8.1e-10	7.37e-08%	0.0	1	0.0115	0.00276	0.00123	1.28e-09	3.86e-08%
**OTU_7230**	**True**	**ABY1**	7.69e-10	7e-08%	0.0	1	0.00783	0.00128	0.000231	8.86e-10	2.66e-08%
**L164**	**False**	**ABY1**	6.17e-10	5.62e-08%	0.0	1	0.00524	0.000484	4.5e-05	1.1e-09	3.31e-08%
**OTU_6022**	**True**	**OD1**	5.75e-10	5.23e-08%	0.0	1	0.00616	0.00142	0.000151	8.21e-10	2.47e-08%
**L1124**	**False**	**koll11**	4.58e-10	4.17e-08%	0.0	1	0.00444	0.0003	0.000347	6.49e-10	1.95e-08%
**L249**	**False**	**ABY1**	4.37e-10	3.98e-08%	0.0	1	0.00497	0.00137	0.000155	7.23e-10	2.17e-08%
**L169**	**False**	**OD1**	4.29e-10	3.9e-08%	0.0	1	0.0518	0.0421	0.03	5.11e-09	1.53e-07%
**L1421**	**False**	**ZB2**	4.16e-10	3.79e-08%	0.0	1	0.000175	0.00311	0.00635	1.1e-09	3.29e-08%
**OTU_8209**	**True**	**ABY1**	3.7e-10	3.37e-08%	0.0	1	5.71e-05	0.00538	0.00272	1.81e-09	5.43e-08%
**L324**	**False**	**ABY1**	3.42e-10	3.11e-08%	0.0	1	0.00332	0.000489	8.77e-05	5e-10	1.5e-08%
**L1455**	**False**	**ZB2**	3.4e-10	3.09e-08%	0.0	1	0.000744	0.00485	0.00737	9.15e-10	2.75e-08%
**L1566**	**False**	**ZB2**	3.16e-10	2.88e-08%	0.0	1	0.00418	0.000608	0.000661	4.21e-10	1.26e-08%
**L1788**	**False**	**OD1**	3.15e-10	2.87e-08%	0.0	1	0.00347	0.000443	0.000295	3.13e-10	9.41e-09%
**L1295**	**False**	**TM7-3**	3.06e-10	2.79e-08%	0.0	1	3.44e-06	0.000104	0.00262	9.45e-10	2.84e-08%
**L763**	**False**	**PBS-25**	2.62e-10	2.39e-08%	0.0	1	0.00213	0.000218	5.83e-08	7.63e-10	2.29e-08%
**L369**	**False**	**Bacteria**	2.58e-10	2.35e-08%	0.0	1	0.85	0.854	0.92	2.05e-09	6.16e-08%
**L1300**	**False**	**EW055**	2.48e-10	2.26e-08%	0.0	1	0.00215	0.000186	4.94e-05	7.8e-10	2.34e-08%
**L682**	**False**	**koll11**	2.46e-10	2.24e-08%	0.0	1	0.00501	0.0014	0.00129	5.97e-10	1.79e-08%
**OTU_5548**	**True**	**OD1**	2.29e-10	2.09e-08%	0.0	1	0.00266	0.000215	0.000472	3.23e-10	9.69e-09%
**L774**	**False**	**PBS-25**	1.88e-10	1.71e-08%	0.0	1	0.00218	0.000217	0.00032	6.17e-10	1.85e-08%
**OTU_7396**	**True**	**ABY1**	1.79e-10	1.63e-08%	0.0	1	0.00245	0.000976	0.00016	7.96e-10	2.39e-08%
**L922**	**False**	**koll11**	1.71e-10	1.56e-08%	0.0	1	0.00113	3.48e-06	3.29e-06	2.78e-10	8.35e-09%
**L896**	**False**	**koll11**	1.53e-10	1.39e-08%	0.0	1	0.00379	0.000992	0.0017	7.71e-10	2.31e-08%
**OTU_851**	**True**	**koll11**	1.49e-10	1.35e-08%	0.0	1	0.00141	0.000118	9.12e-05	2.9e-10	8.69e-09%
**L884**	**False**	**koll11**	1.48e-10	1.35e-08%	0.0	1	0.00318	0.00167	0.000579	7.25e-10	2.18e-08%
**L1732**	**False**	**Mb-NB09**	1.25e-10	1.14e-08%	0.0	1	0.00163	0.00105	6.99e-05	5.87e-10	1.76e-08%
**L689**	**False**	**koll11**	1.21e-10	1.1e-08%	0.0	1	0.00252	0.000512	0.00112	4.24e-10	1.27e-08%
**OTU_4847**	**True**	**ZB2**	1.13e-10	1.03e-08%	0.0	1	0.000864	4.88e-05	5.03e-06	1.58e-10	4.73e-09%
**OTU_7190**	**True**	**ABY1**	1.1e-10	1e-08%	0.0	1	0.0	0.0013	0.000245	3.27e-10	9.82e-09%
**L384**	**False**	**ABY1**	1.07e-10	9.77e-09%	0.0	1	0.000769	2.22e-05	4.17e-06	1.65e-10	4.95e-09%
**L1729**	**False**	**Mb-NB09**	1.07e-10	9.71e-09%	0.0	1	0.00263	0.00138	0.000604	7.13e-10	2.14e-08%
**L640**	**False**	**PBS-25**	9.79e-11	8.91e-09%	0.0	1	0.000692	1.6e-05	4.22e-06	1.64e-10	4.93e-09%
**L388**	**False**	**ABY1**	8.78e-11	7.99e-09%	0.0	1	0.0117	0.00848	0.00762	1.62e-09	4.86e-08%
**L1633**	**False**	**ZB2**	7.77e-11	7.07e-09%	0.0	1	0.00282	0.00141	0.00106	9.35e-10	2.81e-08%
**OTU_6407**	**True**	**ZB2**	7.34e-11	6.68e-09%	0.0	1	1.06e-05	0.00111	0.000529	1.32e-10	3.97e-09%
**OTU_7956**	**True**	**OD1**	6.33e-11	5.77e-09%	0.0	1	0.000521	7.01e-05	0.000979	3.33e-10	9.98e-09%
**OTU_876**	**True**	**koll11**	6.04e-11	5.5e-09%	0.0	1	0.000382	0.0	0.0	1.22e-10	3.66e-09%
**OTU_5946**	**True**	**OD1**	5.89e-11	5.36e-09%	0.0	1	0.000548	4.09e-05	3.98e-05	1.36e-10	4.07e-09%
**L795**	**False**	**PBS-25**	5e-11	4.55e-09%	0.0	1	0.000527	0.000409	3.56e-06	2.12e-10	6.35e-09%
**OTU_309**	**True**	**PBS-25**	3.5e-11	3.19e-09%	0.0	1	0.000478	6.21e-05	0.000119	1.32e-10	3.97e-09%
**OTU_5803**	**True**	**OD1**	3.19e-11	2.9e-09%	0.0	1	0.000274	1.4e-05	1.78e-05	1.03e-10	3.08e-09%
**OTU_6715**	**True**	**ZB2**	3.15e-11	2.87e-09%	0.0	1	0.000279	4.37e-05	3.3e-06	8.65e-11	2.6e-09%
**L371**	**False**	**ABY1**	2.86e-11	2.6e-09%	0.0	1	0.00068	0.000226	0.000229	2.61e-10	7.84e-09%
**OTU_411**	**True**	**koll11**	2.78e-11	2.53e-09%	0.0	1	0.000342	5.56e-05	4.77e-05	1.21e-10	3.64e-09%
**OTU_6109**	**True**	**ZB2**	2.75e-11	2.51e-09%	0.0	1	4.14e-06	0.000414	0.000314	7.86e-11	2.36e-09%
**OTU_6578**	**True**	**WCHB1-64**	2.32e-11	2.11e-09%	0.0	1	0.000112	7.92e-05	0.000464	1.6e-10	4.81e-09%
**OTU_5244**	**True**	**OD1**	2.07e-11	1.88e-09%	0.0	1	0.000222	5.87e-05	8.28e-06	9.03e-11	2.71e-09%
**OTU_1017**	**True**	**PRR-12**	2.07e-11	1.88e-09%	0.0	1	0.000166	0.0	2.43e-05	7.59e-11	2.28e-09%
**OTU_5805**	**True**	**OD1**	1.91e-11	1.74e-09%	0.0	1	0.000302	0.000177	3.09e-05	9.9e-11	2.97e-09%
**OTU_6594**	**True**	**ZB2**	1.73e-11	1.57e-09%	0.0	1	0.000144	2.44e-05	0.0	5.95e-11	1.79e-09%
**L1751**	**False**	**OD1**	1.11e-11	1.01e-09%	0.0	1	0.000721	0.00111	0.00154	6.46e-10	1.94e-08%
**OTU_9186**	**True**	**Mb-NB09**	6.2e-12	5.64e-10%	0.0	1	0.00013	9.95e-05	2.45e-05	6.17e-11	1.85e-09%
**L0**	**False**	**Bacteria**	4.19e-17	3.81e-15%	0.0731	0	1.0	1.0	1.0	1.26e-16	3.77e-15%
**L1**	**False**	**Bacteria**	1.92e-17	1.75e-15%	0.0731	0	1.0	1.0	1.0	5.78e-17	1.73e-15%
**L1960**	**False**	**Archaea**	0.0	0.0%	1.0	0	0.0	0.0	0.0	0.0	0.0%
**L1975**	**False**	**Archaea**	0.0	0.0%	1.0	0	0.0	0.0	0.0	0.0	0.0%

**OTU_10936**	**True**	**Archaea**	0.0	0.0%	1.0	0	0.0	0.0	0.0	0.0	0.0%
**OTU_10954**	**True**	**Archaea**	0.0	0.0%	1.0	0	0.0	0.0	0.0	0.0	0.0%
**L1984**	**False**	**Archaea**	0.0	0.0%	1.0	0	0.0	0.0	0.0	0.0	0.0%
**L1983**	**False**	**Archaea**	0.0	0.0%	1.0	0	0.0	0.0	0.0	0.0	0.0%
**OTU_10977**	**True**	**Archaea**	0.0	0.0%	1.0	0	0.0	0.0	0.0	0.0	0.0%
**L1981**	**False**	**Archaea**	0.0	0.0%	1.0	0	0.0	0.0	0.0	0.0	0.0%
**OTU_13**	**True**	**Archaea**	0.0	0.0%	1.0	0	0.0	0.0	0.0	0.0	0.0%
**OTU_10991**	**True**	**Archaea**	0.0	0.0%	1.0	0	0.0	0.0	0.0	0.0	0.0%
**OTU_11097**	**True**	**Archaea**	0.0	0.0%	1.0	0	0.0	0.0	0.0	0.0	0.0%
**L1977**	**False**	**Archaea**	0.0	0.0%	1.0	0	0.0	0.0	0.0	0.0	0.0%
**OTU_11020**	**True**	**Archaea**	0.0	0.0%	1.0	0	0.0	0.0	0.0	0.0	0.0%
**L1974**	**False**	**Archaea**	0.0	0.0%	1.0	0	0.0	0.0	0.0	0.0	0.0%
**L1988**	**False**	**Archaea**	0.0	0.0%	1.0	0	0.0	0.0	0.0	0.0	0.0%
**L1973**	**False**	**Archaea**	0.0	0.0%	1.0	0	0.0	0.0	0.0	0.0	0.0%
**L1972**	**False**	**Archaea**	0.0	0.0%	1.0	0	0.0	0.0	0.0	0.0	0.0%
**OTU_11044**	**True**	**Archaea**	0.0	0.0%	1.0	0	0.0	0.0	0.0	0.0	0.0%
**OTU_11073**	**True**	**Archaea**	0.0	0.0%	1.0	0	0.0	0.0	0.0	0.0	0.0%
**L1969**	**False**	**Archaea**	0.0	0.0%	1.0	0	0.0	0.0	0.0	0.0	0.0%
**L1968**	**False**	**Archaea**	0.0	0.0%	1.0	0	0.0	0.0	0.0	0.0	0.0%
**OTU_11306**	**True**	**Archaea**	0.0	0.0%	1.0	0	0.0	0.0	0.0	0.0	0.0%
**OTU_11297**	**True**	**Archaea**	0.0	0.0%	1.0	0	0.0	0.0	0.0	0.0	0.0%
**L1965**	**False**	**Archaea**	0.0	0.0%	1.0	0	0.0	0.0	0.0	0.0	0.0%
**OTU_11207**	**True**	**Archaea**	0.0	0.0%	1.0	0	0.0	0.0	0.0	0.0	0.0%
**OTU_10884**	**True**	**Archaea**	0.0	0.0%	1.0	0	0.0	0.0	0.0	0.0	0.0%
**L1987**	**False**	**Archaea**	0.0	0.0%	1.0	0	0.0	0.0	0.0	0.0	0.0%
**OTU_10976**	**True**	**Archaea**	0.0	0.0%	1.0	0	0.0	0.0	0.0	0.0	0.0%
**OTU_11266**	**True**	**Archaea**	0.0	0.0%	1.0	0	0.0	0.0	0.0	0.0	0.0%
**OTU_10957**	**True**	**Archaea**	0.0	0.0%	1.0	0	0.0	0.0	0.0	0.0	0.0%
**OTU_21**	**True**	**Archaea**	0.0	0.0%	1.0	0	0.0	0.0	0.0	0.0	0.0%
**L2012**	**False**	**Archaea**	0.0	0.0%	1.0	0	0.0	0.0	0.0	0.0	0.0%
**OTU_20**	**True**	**Archaea**	0.0	0.0%	1.0	0	0.0	0.0	0.0	0.0	0.0%
**L2010**	**False**	**Archaea**	0.0	0.0%	1.0	0	0.0	0.0	0.0	0.0	0.0%
**OTU_10959**	**True**	**Archaea**	0.0	0.0%	1.0	0	0.0	0.0	0.0	0.0	0.0%
**L2008**	**False**	**Archaea**	0.0	0.0%	1.0	0	0.0	0.0	0.0	0.0	0.0%
**OTU_11285**	**True**	**Archaea**	0.0	0.0%	1.0	0	0.0	0.0	0.0	0.0	0.0%
**OTU_11271**	**True**	**Archaea**	0.0	0.0%	1.0	0	0.0	0.0	0.0	0.0	0.0%
**OTU_11308**	**True**	**Archaea**	0.0	0.0%	1.0	0	0.0	0.0	0.0	0.0	0.0%
**L2004**	**False**	**Archaea**	0.0	0.0%	1.0	0	0.0	0.0	0.0	0.0	0.0%
**OTU_11284**	**True**	**Archaea**	0.0	0.0%	1.0	0	0.0	0.0	0.0	0.0	0.0%
**L2002**	**False**	**Archaea**	0.0	0.0%	1.0	0	0.0	0.0	0.0	0.0	0.0%
**OTU_22**	**True**	**Archaea**	0.0	0.0%	1.0	0	0.0	0.0	0.0	0.0	0.0%
**L2000**	**False**	**Archaea**	0.0	0.0%	1.0	0	0.0	0.0	0.0	0.0	0.0%
**OTU_11336**	**True**	**Archaea**	0.0	0.0%	1.0	0	0.0	0.0	0.0	0.0	0.0%
**L1998**	**False**	**Archaea**	0.0	0.0%	1.0	0	0.0	0.0	0.0	0.0	0.0%
**OTU_17**	**True**	**Archaea**	0.0	0.0%	1.0	0	0.0	0.0	0.0	0.0	0.0%
**L1996**	**False**	**Archaea**	0.0	0.0%	1.0	0	0.0	0.0	0.0	0.0	0.0%
**L1995**	**False**	**Archaea**	0.0	0.0%	1.0	0	0.0	0.0	0.0	0.0	0.0%
**OTU_11180**	**True**	**Archaea**	0.0	0.0%	1.0	0	0.0	0.0	0.0	0.0	0.0%
**L1993**	**False**	**Archaea**	0.0	0.0%	1.0	0	0.0	0.0	0.0	0.0	0.0%
**L1992**	**False**	**Archaea**	0.0	0.0%	1.0	0	0.0	0.0	0.0	0.0	0.0%
**OTU_11000**	**True**	**Archaea**	0.0	0.0%	1.0	0	0.0	0.0	0.0	0.0	0.0%
**L1962**	**False**	**Archaea**	0.0	0.0%	1.0	0	0.0	0.0	0.0	0.0	0.0%
**OTU_11312**	**True**	**Archaea**	0.0	0.0%	1.0	0	0.0	0.0	0.0	0.0	0.0%
**OTU_11167**	**True**	**Archaea**	0.0	0.0%	1.0	0	0.0	0.0	0.0	0.0	0.0%
**L1921**	**False**	**Archaea**	0.0	0.0%	1.0	0	0.0	0.0	0.0	0.0	0.0%
**OTU_11194**	**True**	**Archaea**	0.0	0.0%	1.0	0	0.0	0.0	0.0	0.0	0.0%
**L1930**	**False**	**Archaea**	0.0	0.0%	1.0	0	0.0	0.0	0.0	0.0	0.0%
**OTU_11276**	**True**	**Archaea**	0.0	0.0%	1.0	0	0.0	0.0	0.0	0.0	0.0%
**OTU_10942**	**True**	**Archaea**	0.0	0.0%	1.0	0	0.0	0.0	0.0	0.0	0.0%
**OTU_11120**	**True**	**Archaea**	0.0	0.0%	1.0	0	0.0	0.0	0.0	0.0	0.0%
**L1926**	**False**	**Archaea**	0.0	0.0%	1.0	0	0.0	0.0	0.0	0.0	0.0%
**L1925**	**False**	**Archaea**	0.0	0.0%	1.0	0	0.0	0.0	0.0	0.0	0.0%
**OTU_10973**	**True**	**Archaea**	0.0	0.0%	1.0	0	0.0	0.0	0.0	0.0	0.0%
**L1923**	**False**	**Archaea**	0.0	0.0%	1.0	0	0.0	0.0	0.0	0.0	0.0%
**L1922**	**False**	**Archaea**	0.0	0.0%	1.0	0	0.0	0.0	0.0	0.0	0.0%

**OTU_10967**	**True**	**Archaea**	0.0	0.0%	1.0	0	0.0	0.0	0.0	0.0	0.0%
**L1959**	**False**	**Archaea**	0.0	0.0%	1.0	0	0.0	0.0	0.0	0.0	0.0%
**L1919**	**False**	**Archaea**	0.0	0.0%	1.0	0	0.0	0.0	0.0	0.0	0.0%
**L1918**	**False**	**Archaea**	0.0	0.0%	1.0	0	0.0	0.0	0.0	0.0	0.0%
**OTU_11012**	**True**	**Archaea**	0.0	0.0%	1.0	0	0.0	0.0	0.0	0.0	0.0%
**OTU_11188**	**True**	**Archaea**	0.0	0.0%	1.0	0	0.0	0.0	0.0	0.0	0.0%
**L1915**	**False**	**Archaea**	0.0	0.0%	1.0	0	0.0	0.0	0.0	0.0	0.0%
**L1914**	**False**	**Archaea**	0.0	0.0%	1.0	0	0.0	0.0	0.0	0.0	0.0%
**L1913**	**False**	**Archaea**	0.0	0.0%	1.0	0	0.0	0.0	0.0	0.0	0.0%
**L1912**	**False**	**Archaea**	0.0	0.0%	1.0	0	0.0	0.0	0.0	0.0	0.0%
**OTU_5181**	**True**	**BB36**	0.0	0.0%	1.0	0	0.0	0.0	0.0	0.0	0.0%
**OTU_8171**	**True**	**TM7-3**	0.0	0.0%	1.0	0	0.0	0.0	0.0	0.0	0.0%
**L1932**	**False**	**Archaea**	0.0	0.0%	1.0	0	0.0	0.0	0.0	0.0	0.0%
**OTU_11121**	**True**	**Archaea**	0.0	0.0%	1.0	0	0.0	0.0	0.0	0.0	0.0%
**OTU_11281**	**True**	**Archaea**	0.0	0.0%	1.0	0	0.0	0.0	0.0	0.0	0.0%
**OTU_11341**	**True**	**Archaea**	0.0	0.0%	1.0	0	0.0	0.0	0.0	0.0	0.0%
**OTU_102**	**True**	**Archaea**	0.0	0.0%	1.0	0	0.0	0.0	0.0	0.0	0.0%
**L1955**	**False**	**Archaea**	0.0	0.0%	1.0	0	0.0	0.0	0.0	0.0	0.0%
**OTU_3999**	**True**	**Archaea**	0.0	0.0%	1.0	0	0.0	0.0	0.0	0.0	0.0%
**L1953**	**False**	**Archaea**	0.0	0.0%	1.0	0	0.0	0.0	0.0	0.0	0.0%
**OTU_144**	**True**	**Archaea**	0.0	0.0%	1.0	0	0.0	0.0	0.0	0.0	0.0%
**L1951**	**False**	**Archaea**	0.0	0.0%	1.0	0	0.0	0.0	0.0	0.0	0.0%
**L1950**	**False**	**Archaea**	0.0	0.0%	1.0	0	0.0	0.0	0.0	0.0	0.0%
**L1949**	**False**	**Archaea**	0.0	0.0%	1.0	0	0.0	0.0	0.0	0.0	0.0%
**OTU_11287**	**True**	**Archaea**	0.0	0.0%	1.0	0	0.0	0.0	0.0	0.0	0.0%
**L1935**	**False**	**Archaea**	0.0	0.0%	1.0	0	0.0	0.0	0.0	0.0	0.0%
**L1946**	**False**	**Archaea**	0.0	0.0%	1.0	0	0.0	0.0	0.0	0.0	0.0%
**L1945**	**False**	**Archaea**	0.0	0.0%	1.0	0	0.0	0.0	0.0	0.0	0.0%
**OTU_10979**	**True**	**Archaea**	0.0	0.0%	1.0	0	0.0	0.0	0.0	0.0	0.0%
**OTU_11015**	**True**	**Archaea**	0.0	0.0%	1.0	0	0.0	0.0	0.0	0.0	0.0%
**OTU_11153**	**True**	**Archaea**	0.0	0.0%	1.0	0	0.0	0.0	0.0	0.0	0.0%
**L1941**	**False**	**Archaea**	0.0	0.0%	1.0	0	0.0	0.0	0.0	0.0	0.0%
**L1940**	**False**	**Archaea**	0.0	0.0%	1.0	0	0.0	0.0	0.0	0.0	0.0%
**OTU_10982**	**True**	**Archaea**	0.0	0.0%	1.0	0	0.0	0.0	0.0	0.0	0.0%
**L1938**	**False**	**Archaea**	0.0	0.0%	1.0	0	0.0	0.0	0.0	0.0	0.0%
**OTU_10944**	**True**	**Archaea**	0.0	0.0%	1.0	0	0.0	0.0	0.0	0.0	0.0%
**OTU_10946**	**True**	**Archaea**	0.0	0.0%	1.0	0	0.0	0.0	0.0	0.0	0.0%
**OTU_112**	**True**	**Archaea**	0.0	0.0%	1.0	0	0.0	0.0	0.0	0.0	0.0%
**OTU_8811**	**True**	**TM7-1**	-4.12666858936e-09	-3.75625562533e-07%	1.0	0	0.000125	0.00015	0.000111	1.31e-05	0.000392%
**L1478**	**False**	**ZB2**	-2.61521032376e-08	-2.38046702256e-06%	1.0	0	0.000269	0.000387	0.000302	1.01e-05	0.000303%
**L1674**	**False**	**OD1**	-2.91522705834e-08	-2.65355402302e-06%	1.0	0	0.00031	0.000362	0.000306	1.51e-05	0.000453%
**L1746**	**False**	**OD1**	-9.75070249994e-08	-8.87547190262e-06%	1.0	0	0.00139	0.00172	0.00185	5.03e-05	0.00151%
